# 4‐Methyltetrahydropyran (4‐MeTHP): Application as an Organic Reaction Solvent

**DOI:** 10.1002/asia.201901169

**Published:** 2019-10-16

**Authors:** Shoji Kobayashi, Tomoki Tamura, Saki Yoshimoto, Takashi Kawakami, Araki Masuyama

**Affiliations:** ^1^ Department of Applied Chemistry, Faculty of Engineering Osaka Institute of Technology 5-16-1 Ohmiya, Asahi-ku Osaka 535-8585 Japan

**Keywords:** 2-methyltetrahydrofuran, 4-methyltetrahydropyran, green solvent, organic reaction, radical

## Abstract

4‐Methyltetrahydropyran (4‐MeTHP) is a hydrophobic cyclic ether with potential for industrial applications. We herein report, for the first time, a comprehensive study on the performance of 4‐MeTHP as an organic reaction solvent. Its broad application to organic reactions includes radical, Grignard, Wittig, organometallic, halogen‐metal exchange, reduction, oxidation, epoxidation, amidation, esterification, metathesis, and other miscellaneous organic reactions. This breadth suggests 4‐MeTHP can serve as a substitute for conventional ethers and harmful halogenated solvents. However, 4‐MeTHP was found incompatible with strong Lewis acids, and the C−O bond was readily cleaved by treatment with BBr_3_. Moreover, the radical‐based degradation pathways of 4‐MeTHP, THP and 2‐MeTHF were elucidated on the basis of GC‐MS analyses. The data reported herein is anticipated to be useful for a broad range of synthetic chemists, especially industrial process chemists, when selecting the reaction solvent with green chemistry perspectives.

## Graphical abstract

**Organic pastures greener**: A comprehensive study on the performance of 4‐methyltetrahydropyran (4‐MeTHP) as a reaction solvent is described. The broad applications and higher stability under free‐radical conditions make this solvent a promising alternative to common organic solvents. This study provides key data for process chemists in choosing a reaction solvent under green chemistry perspectives.

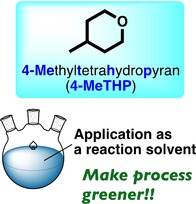

## Introduction

The selection of a reaction solvent is important in a myriad of synthetic processes to make chemical and pharmaceutical products.^1^, ^2^ From an industrial point of view, safety, health and environmental aspects are seriously taken into account in addition to the general requirements of solubility, stability, removability (volatility), and compatibility of reactants.^3^, ^4^, ^5^, ^6^, ^7^ The traditional ethereal solvents, such as diethyl ether (Et_2_O), tetrahydrofuran (THF) and 1,4‐dioxane, which are still used in many laboratory experiments today, are highly recommended to be replaced by safer and greener solvents in pilot‐scale synthesis, in order to avoid the risks of inflammability, explosion from peroxide build‐up, and toxic exposure. In this regard, less risky, recyclable ethereal solvents such as 2‐methyltetrahydrofuran (2‐MeTHF)^8^, ^9^, ^10^ and cyclopentyl methyl ether (CPME)^11^, ^12^, ^13^ have been developed as alternatives to conventional ethers, which are nowadays widely used in process chemistry.^14^ *tert*Butyl methyl ether (TBME), developed as an antiknock agent for gasoline, is also used occasionally; however, its flammable nature and volatile organic compound emissions are matters of concern.^6^, ^7^ Other related ethers such as *tert‐*butyl ethyl ether (TBEE) and *tert‐*amyl methyl ether (TAME), the possible replacements for TBME, are still less common and more expensive, and their use in organic synthesis has yet to be explored.^6^ More recently, 2,2,5,5‐tetramethyltetrahydrofuran (TMTHF) has been reported as a non‐polar, non‐peroxide forming ether and the solvent properties were found to be similar to those of toluene rather than traditional ethers.^15^

During recent years, we have extensively explored the applicability of newly‐developed, less common organic solvents to reactions in order to make existing synthetic processes more environmentally benign. In particular, we have demonstrated the usefulness of tetrahydropyran (THP) and CPME as the solvents for classical organic reactions involving radical reactions, palladium‐catalyzed coupling and Grignard reactions.^16^, ^17^, ^18^ Our achievements have led to improvements of the synthetic process of widely‐used pharmaceuticals such as tamoxifen and tramadol hydrochloride.^18^ It is notable that the strong hydrophobicity and low heat of vaporization of THP and CPME enabled easy recovery and reuse of the solvents, making the whole synthetic processes greener. Overall, readily available CPME appears a good alternative to conventional solvents; however, we sometimes encountered difficulties in carrying out reactions in CPME. For instance, highly labile Grignard reagents such as propargyl, 2‐chlorophenyl, and 2‐fluorophenylmagnesium bromide barely formed in CPME.^18^ In addition, neither the Grignard formation of less reactive aromatic chlorides nor halogen‐metal exchange of aromatic bromides were successful when using CPME as a single solvent.^18^ These negative observations prompted us to search other commercially available, yet‐to‐be‐explored, low‐molecular weight volatile ethers as the backup solvents.

**Scheme 1:**
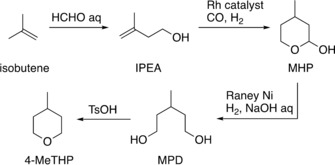
Manufacturing process of 4‐MeTHP. IPEA=isopentyl alcohol, MHP=2‐hydroxy‐4‐methyltetrahydropyran, MPD=3‐methylpentan‐1,5‐diol.

Among the candidates, 4‐methyltetrahydropyran (4‐MeTHP)^19^ attracted us, since it has physical properties similar to CPME (Table 1) and is available at a reasonable price, competitive with traditional solvents.^20^ Industrial 4‐MeTHP is supplied from isobutene via a four‐step reaction sequence, including a carbonyl‐ene reaction (Prins reaction), hydroformylation, hydrogenation, and intramolecular dehydrative etherification (Scheme 1).^19^, ^20^, ^21^, ^22^ Although it originates from petroleum, the established manufacturing process involving three addition reactions and one dehydration contributes to the minimization of chemical waste as much as possible. Moreover, it is advantageous that 4‐MeTHP is recyclable because of its immiscibility with water and its ability to be recovered by liquid‐liquid extraction and evaporation. The published safety data including LD_50_ and NOEL (no observed effect level) for 4‐MeTHP are comparable to those for THF, 2‐MeTHF and CPME.^23^ As there are only a few reports on the application of 4‐MeTHP in organic synthesis,^24^, ^25^ we investigated the performance of 4‐MeTHP in a broad range of organic reactions. We herein report the systematic application of 4‐MeTHP in a variety of organic reactions, which demonstrated 4‐MeTHP to be a promising alternative to not only conventional ethers but also harmful halogenated solvents in a wide range of reaction types.

**Table 1:** Selected physical data of 4‐MeTHP, THP, CPME, 2‐MeTHF and THF.

Solvent property	4‐MeTHP	THP	CPME	2‐MeTHF	THF
Boiling point [°C]	105^[a]^	88^[a]^	106^[a]^	80^[a]^	65^[a]^
Melting point [°C]	−92^[c]^	−45^[g]^	<−140^[b]^	−136^[b]^	−108.5^[b]^
Density [g cm^−3^]	0.857^[c]^	0.88^[g]^	0.86^[b]^	0.85^[b]^	0.89^[b]^
Solubility in water [g/100 g, 23 °C]	1.5^[a]^	8^[a]^	1.1^[a]^	14^[a]^	miscible^[a]^
Dielectric constant [25 °C]	4.8^[c]^	5.7^[a]^	4.8^[a]^	7.0^[a]^	7.6^[a]^
Dipole moment [*D*]	1.86 (calcd)^[c]^	1.63^[f]^	1.27 (calcd)^[b]^	1.38^[e]^	1.7^[b]^
Log Pow	1.9^[c]^	unknown	1.59^[b]^	0.77^[d]^	0.47^[b]^
Flash point [°C]	6.5^[c]^	−16^[g]^	−1^[b]^	−11^[b]^	−14.5^[b]^

[a] Patent;^19^ [b] Ref. 11; [c] Published data;^23^ [d] Ref. 15; [e] Ref. 8; [f] Symposium abstract;^26^ [g] Safety data sheet (Sigma–Aldrich).^27^

## Results and Discussion

### Applications to radical reactions and radical‐based degradation pathways

**Scheme 2:**
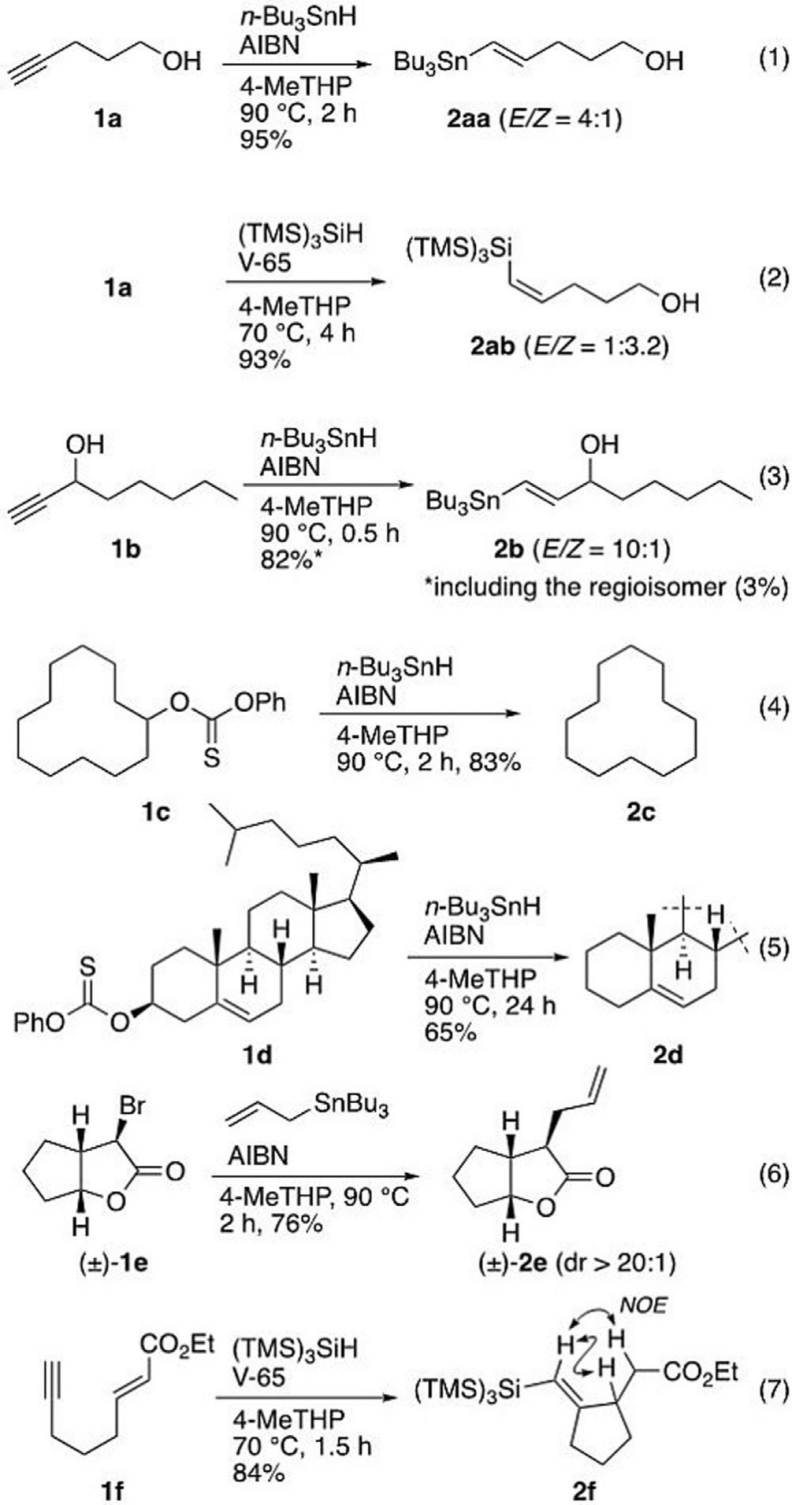
Radical reactions in 4‐MeTHP. V‐65=2,2′‐azobis(2,4‐dimethylvaleronitrile)

Initially we set out to test radical reaction compatibilities, as it is expected the six‐membered THP derivatives would be more stable to autooxidation as compared to five‐membered THF derivatives.^16^, ^28^ Indeed, a selection of radical reactions in laboratory experiments are still occasionally carried out in carcinogenic benzene,^29^ a solvent in great need of replacement in view of the health hazards. As representative examples, tin or silicon radical‐mediated additions,^30^, ^31^ reductions,^32^ and cyclizations were carried out in 4‐MeTHP (Scheme 2). Overall, the reactions progressed smoothly with almost the same yield and selectivity as with the conventional solvents.^17^ The success of the allylation and cyclization reactions indicates that hydrogen abstraction from the solvent was not a matter of concern.

After demonstrating that 4‐MeTHP can be selected for representative radical reactions, we turned our attention to recycling the solvent and evaluating its recovered quality. In a typical radical experiment between alkyne **1 b** and *n*Bu_3_SnH [Scheme 2, Eq. (3)], the solvent was recovered by distillation under reduced pressure and analyzed by GC and GC‐MS. The recovered solvent was again subjected to the same radical reaction and recovered to reevaluate its quality. As comparisons, THP and 2‐MeTHF were also tested in the same reaction system and the purity of the recovered solvents was systematically analyzed. We confirmed that each reaction progressed in more than 60 % yield with an *E/Z* ratio ranging from 16:1 to 9:1, irrespective of the solvent used. In addition, each solvent was recovered in 80–90 % yield by standard distillation techniques. Table 2 shows the purity of the recovered solvents. Notably, the purity of 4‐MeTHP and THP remained almost unchanged (99.99 %→99.88 % for 4‐MeTHP, 99.63 %→99.77 % for THP), while that of 2‐MeTHF clearly decreased after two recycles (99.93 %→99.59 %).

**Table 2:** Purity of the recovered solvents after the reactions with **1 b** and *n*Bu_3_SnH.^[a]^

run	4‐MeTHP [%]	THP [%]	2‐MeTHF [%]
initial	99.99	99.63	99.93
1	99.94	99.75	99.96
2	99.88	99.77	99.59

[a] In a typical experiment, a solution of **1 b** (1 mmol), *n*Bu_3_SnH (1.3 mmol), and an initiator (0.2 mmol) in the solvent (20 mL) was refluxed for 30 min, after which the solvent was recovered by distillation under reduced pressure and analyzed by GC and GC‐MS to determine the purity. For reactions in 4‐MeTHP, AIBN was used as an initiator, while V‐65 was used for reactions in THP and 2‐MeTHF.

**Figure 1:**
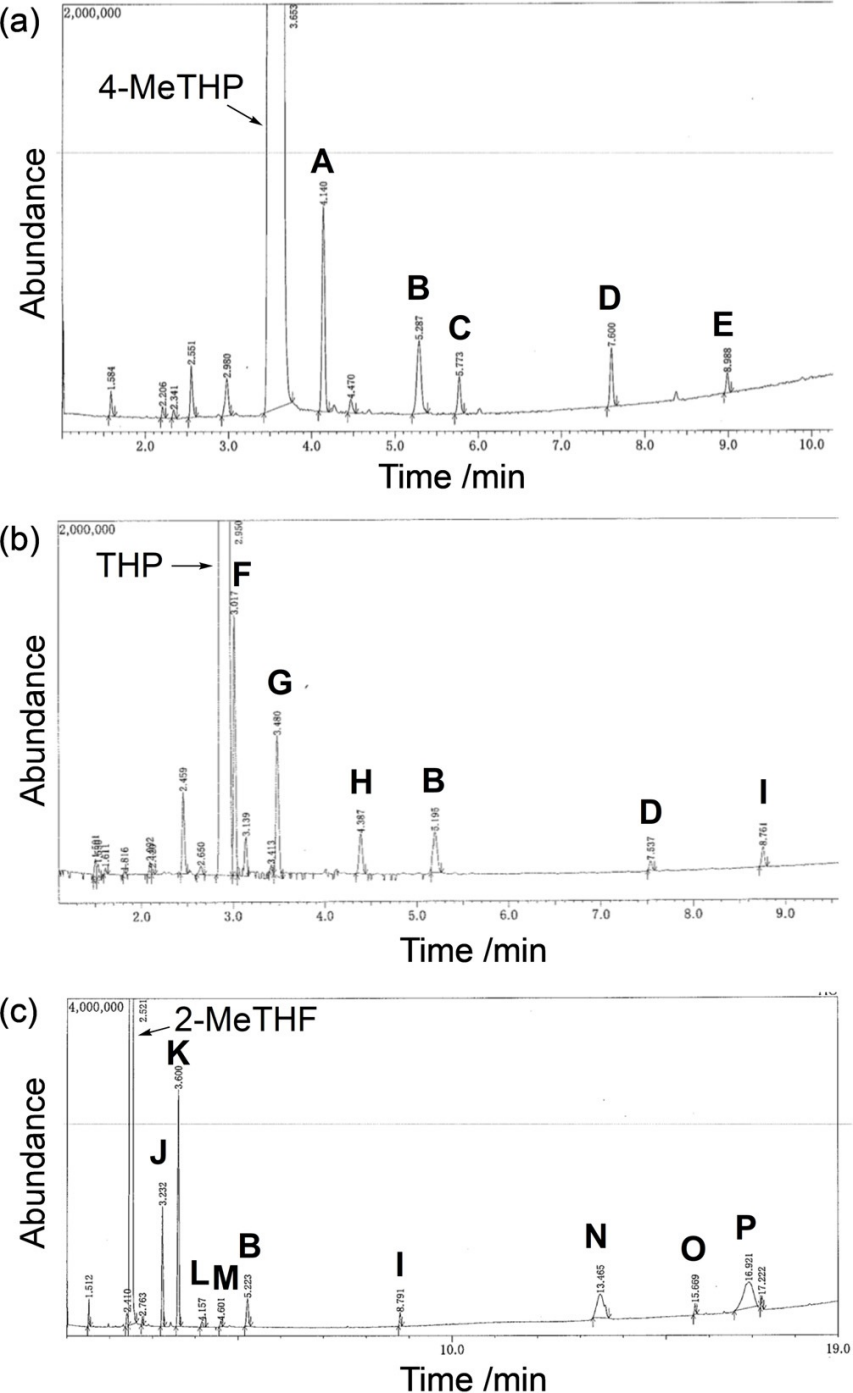
GC‐MS chromatograms of (a) 4‐MeTHP, (b) THP and (c) 2‐MeTHF after two recycles. Peak assignments were made by comparison with the reference data (NIST 14 mass spectral library) or the retention time of authentic samples. **A**=isobutyronitrile; **B**=H_2_O; **C**=isopentyl formate; **D**=1‐butanol; **E**=isopentanol; **F**=2‐methyltetrahydropyran; **G**=3‐methyltetrahydropyran; **H**=*n‐butyl* formate; **I**=2,4‐dimethylpentanenitrile; **J**=*sec*‐butyl formate; **K**=*n*‐propyl acetate; **L**=unidentifiable; **M**=2‐butanol; **N**=5‐methyltetrahydrofuran‐2‐ol (This peak may contain 5‐methyl‐2,3‐dihydrofuran derived from degradation of 5‐hydroxypentan‐2‐one); **O**=γ‐valerolactone; **P**=5‐hydroxypentan‐2‐one.

The GC and the GC‐MS spectra of the recovered solvents showed trace amounts of degradation side‐products, some of which were clearly derived from the solvent (Figure 1). In the case of 4‐MeTHP, isopentyl formate (**C**) and isopentanol (**E**) were identified as degradation products, and their content ratios as determined by GC were 0.009 % and 0.004 %, respectively (chart a). Similarly, *n‐*butyl formate (**H**) was identified as a degradation product of THP with a content ratio of 0.003 % (chart b). It is thought that 1‐butanol (**D**) is also a possible degradation product of THP, however, this possibility is ambiguous since degradation of *n*Bu_3_SnH may produce 1‐butanol as well. This assumption is supported by the observation of peak **D** in the recovered 4‐MeTHP (chart a). It is noteworthy that more peaks appeared in the recovered 2‐MeTHF after two recycles, which were identified as *sec*‐butyl formate (**J**), *n*‐propyl acetate (**K**), 2‐butanol (**M**), 5‐methyltetrahydrofuran‐2‐ol (**N**), γ‐valerolactone (**O**) and 5‐hydroxypentan‐2‐one (**P**) (chart c).^33^ The GC analyses indicated that the content ratios of peaks **J** and **K** were 0.09 % and 0.16 %, respectively, which were more than ten times larger than in the case of 4‐MeTHP and THP.

**Scheme 3:**
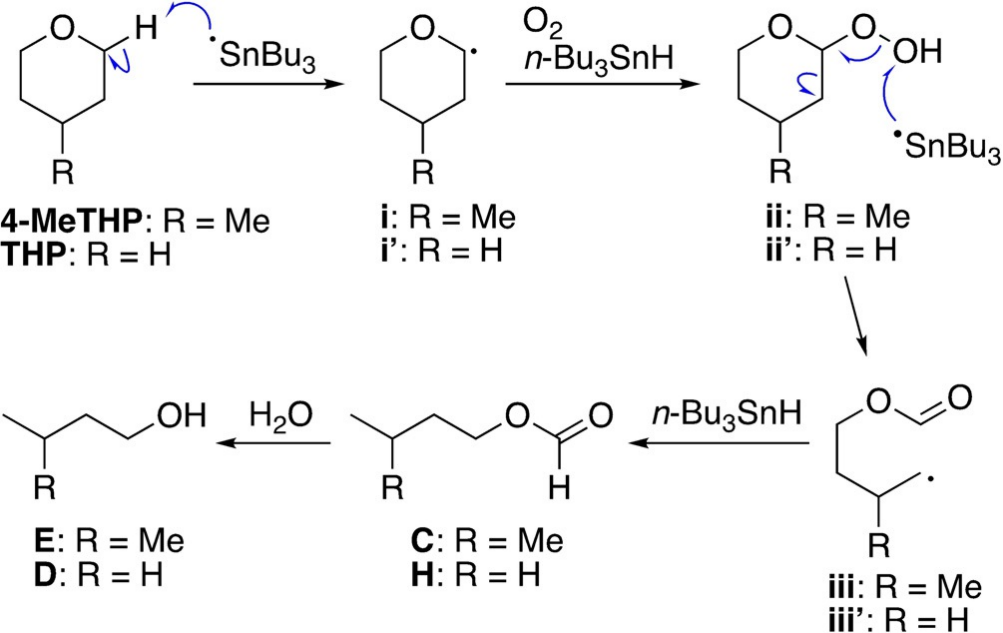
Possible degradation pathways of 4‐MeTHP and THP under radical addition conditions.

With this experimental evidence in hand, the radical‐based degradation pathway of each solvent was deduced. In the case of 4‐MeTHP and THP, C2‐H is initially abstracted by tributyltin radicals to provide carbon radicals (**i** and **i′**), which are trapped by dissolved oxygen to provide peroxides (**ii** and **ii′**) after hydrogen abstraction from *n*Bu_3_SnH (Scheme 3). Next, a tin radical‐induced C−C bond scission takes place and the resulting carbon radicals (**iii** and **iii′**) abstract hydrogen from *n*Bu_3_SnH to provide formyl esters (**C** and **H**), which are hydrolyzed by trace amounts of water during reactions or under analytical conditions to afford isopentanol (**E**) and 1‐butanol (**D**). Unlike CPME, degradation leading to hemiacetal (i.e. 4‐methyl‐2‐hydroxytetrahydropyran from 4‐MeTHP and 2‐hydroxytetrahydropyran from THP) via direct reduction of peroxides (**ii** and **ii′**) was not observed under the experimental conditions.^17^

**Scheme 4:**
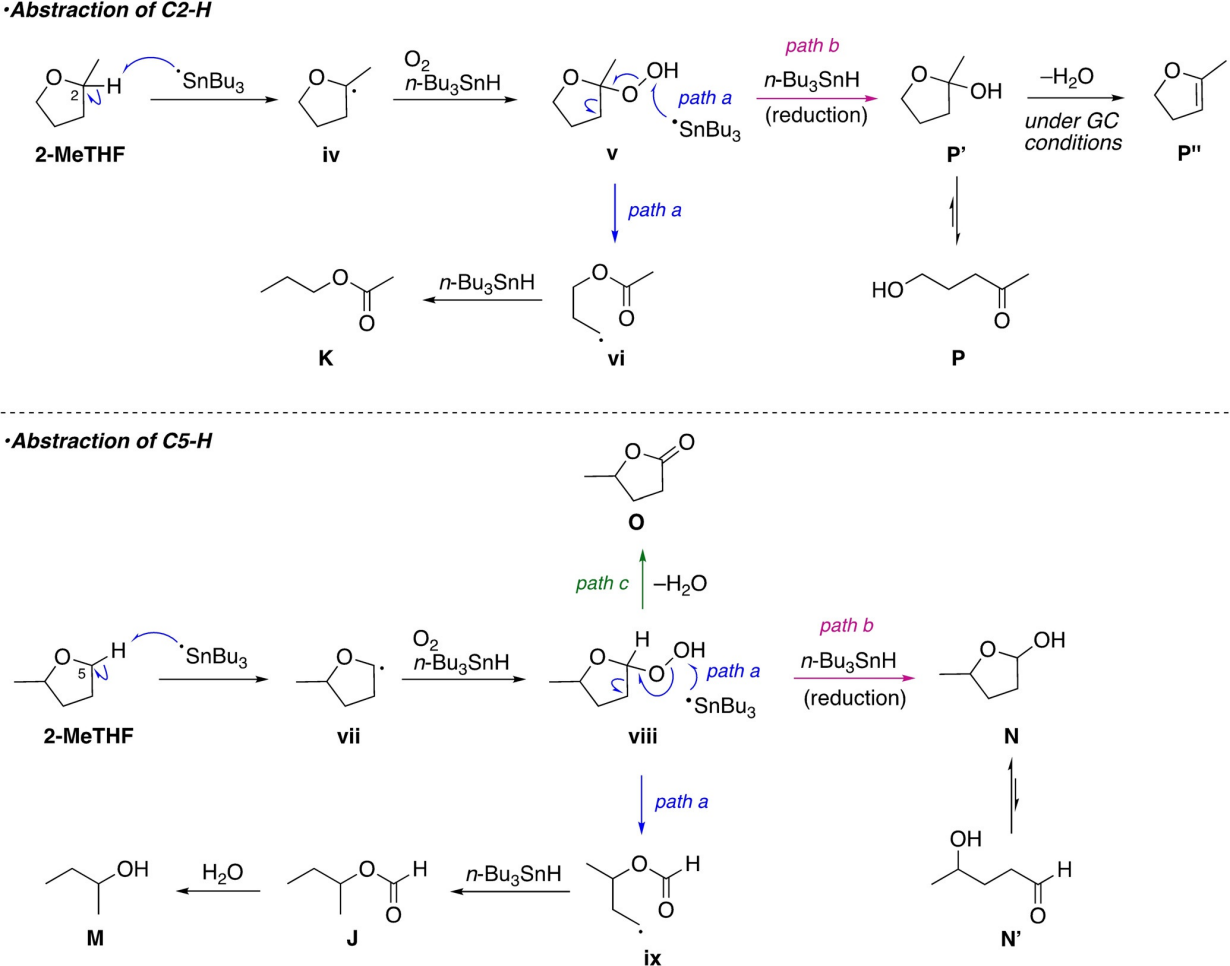
Possible degradation pathways of 2‐MeTHF under the radical addition conditions.

Scheme 4 represents plausible degradation pathways of 2‐MeTHF. Importantly, there are two positions, namely C2 and C5, where hydrogen abstraction can occur. The major degradation path begins with abstraction of C2‐H to produce the relatively stable tertiary radicals (**iv**), which react with dissolved oxygen to afford peroxide (**v**) after hydrogen abstraction. The following pathway is the same as that for 4‐MeTHP and THP, which results in the formation of *n*‐propyl acetate (**K**) through the C−C bond cleavage (path a). As a minor pathway, reduction of peroxide (**v**) with *n*Bu_3_SnH provides the observed hydroxyketone (**P**) that is in equilibrium with hemiacetal (**P′**) (path b).^34^ Along with C2‐H abstraction, degradation arising from C5‐H abstraction is competitive. Thus, the peroxide intermediate (**viii**) is produced via C5‐H abstraction and can degrade to a formyl ester (**J**) and 2‐butanol (**M**) through C−C bond cleavage and hydrolysis (path a), or through hydride reduction to a hemiacetal (**N**) (path b).^35^ Additionally, γ‐valerolactone (**O**) can form by elimination of water of peroxide (**viii**). Although the lactone formation was slight under the experimental conditions, the presence of such a degradation pathway is supported by contamination of *δ*‐lactone in the prolonged storage of THP.^36^ Despite a pronounced reactivity of 2‐MeTHF relative to 4‐MeTHP under free‐radical conditions, it should be noted that degradation could be substantially suppressed by the presence of a radical scavenger, 2,6‐di‐*tert*‐butyl‐4‐hydroxytoluene (BHT), as evidenced by a sustained purity of the solvent after the first reaction (Table 2).

**Scheme 5:**
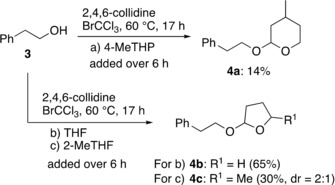
Solvent reactions with primary alcohol under free‐radical conditions.

A higher stability of 4‐MeTHP over THF and 2‐MeTHF under the free‐radical conditions was further confirmed by the reaction of 2‐phenylethanol (**3**) with BrCCl_3_ under heating conditions in which a radical chain mechanism is involved (Scheme 5).^37^ In contrast to higher yields of a THF and a 2‐MeTHF adducts (**4 b**: 65 %, **4 c**: 30 %), a THP adduct **4 a** was obtained in only 14 % yield, reflecting the slower rate of autooxidation of 4‐MeTHP relative to THF and 2‐MeTHF.^28^, ^37^ It should be pointed out that many unidentifiable side‐products appeared when using 2‐MeTHF, which probably resulted from degradation via C2‐H abstraction. Although the simple alcohol adduct at C2 was not identified, one of the minor components was found to involve a singlet peak (*δ*=1.42) corresponding to C2‐Me (ca. 5 % yield). Mass spectroscopic analysis suggested that some minor products contained bromine(s) in the molecule.

In view of these experimental results, it can be concluded that 4‐MeTHP is more stable to free‐radical conditions than THF and 2‐MeTHF, and can serve as a radical reaction solvent, although there is another option to carry out radical reactions in 2‐MeTHF in the presence of an appropriate amount of a radical scavenger.

### Applications to Grignard reactions

**Scheme 6:**
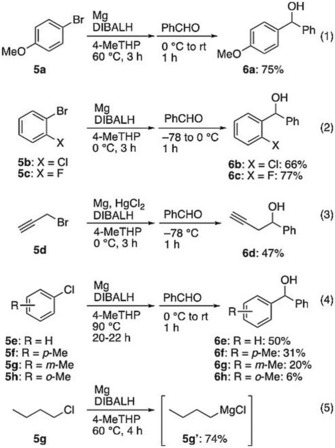
Grignard reactions in 4‐MeTHP. Each reaction was carried out with aryl or alkyl halide (10 mmol), magnesium (15 mmol), DIBALH (0.0075 mmol) and benzaldehyde (10 mmol). For the formation of propargylmagnesium bromide, HgCl_2_ (0.0050 mmol) was added [Eq. (3)]. For the formation of arylmagnesium chloride, ten times amount of DIBALH (0.075 mmol) was used [Eq. 4)].

Next, we turned our attention to Grignard reactions in order to evaluate solvent compatibility with organometallic reagents. As mentioned in the introduction, we have previously surveyed Grignard reactions using CPME as an alternative solvent for Et_2_O and THF.^18^ As a follow up to our previous study, we chose the substrates that formed Grignard reagents with difficulty in CPME. We applied the reaction conditions optimized in the previous study, where DIBALH was used as the activator of magnesium.^18^, ^38^, ^39^ After successful demonstration of the representative Grignard reaction with 4‐bromoanisole (**5 a**) and benzaldehyde [Scheme 6, Eq. (1)], we embarked on the preparation of highly labile 2‐chloro and 2‐fluorophenylmagnesium bromide [Eq. (2)].^40^ To suppress spontaneous decomposition of Grignard reagents, preparation of Grignard reagents and addition of the electrophile were carried out at 0 °C and −78 °C, respectively. Under these conditions, the desired alcohols **6 b** and **6 c** were obtained in 66 % and 77 % yields, respectively, demonstrating the advantage of 4‐MeTHP over CPME.^18^ The next challenge is the preparation of propargyl Grignard reagent that was also found incompatible with CPME.^18^ In practice, Grignard formation occurred smoothly at 0 °C and, upon addition of aldehyde at −78 °C, alcohol **6 d** was formed in 47 % yield [Eq. (3)]. Although the yield was lower than when using Et_2_O (71 % yield) in our technique, it was comparable to that with 2‐MeTHF (49 % yield).

We next turned our attention to the preparation of Grignard reagents from the less reactive chlorobenzene (**5 e**), whereby a co‐solvent (THF) was necessary when using CPME.^18^ The use of ten times amount of DIBALH with prolonged heating at 90 °C led to the formation of a gray suspension of phenylmagnesium chloride, to which benzaldehyde was added after cooling the Grignard solution at 0 °C. The desired alcohol **6 e** was obtained in a maximum yield of 50 % after chromatography [Eq. (4)]. With this positive result in hand, we briefly checked the substrate scope with various tolyl chlorides (**5 f**–**5 h**). In practice, **6 f**–**6 h** were obtained in modest to low yields and biaryl side‐products, resulting from Wurtz coupling of aryl chlorides, were detected, which could not be suppressed by modification of the conditions [Eq. (4)]. When the Grignard formation of **5 f** was carried out at 60 °C for 20 h, only the biaryl side‐product was detected without forming the desired product. To our knowledge, commercially available tolylmagnesium chloride are usually prepared as a THF solution, probably due to the difficulty in preparing in other solvent.

On the whole, 4‐MeTHP showed better results than CPME. It is likely that effective coordination of the less hindered oxygen of 4‐MeTHP (relative to the oxygen of CPME) to the magnesium contributes to the stability and the reactivity of Grignard reagents. From the physical data associated with solvent polarity, there is a small difference in dipole moment, while dielectric constant, solubility in water, and log Pow are nearly the same between 4‐MeTHP and CPME (Table 1). It is recommended that more detailed physicochemical study is performed to understand these experimental results.

Finally, we checked the stability of the representative Grignard reagent, *n‐*butylmagnesium chloride (**5 g′**) in 4‐MeTHP, that was prepared from 4‐chlorobutane (**5 g**) in 74 % yield [Eq. (5)]. The solution was stored in a sealed brown glass bottle at a concentration of 0.66 m at room temperature. After three months, no decrease in concentration was observed as checked by titration. Such practicalities bode well for other industrial‐scale applications.

### Applications to coupling reactions

**Scheme 7:**
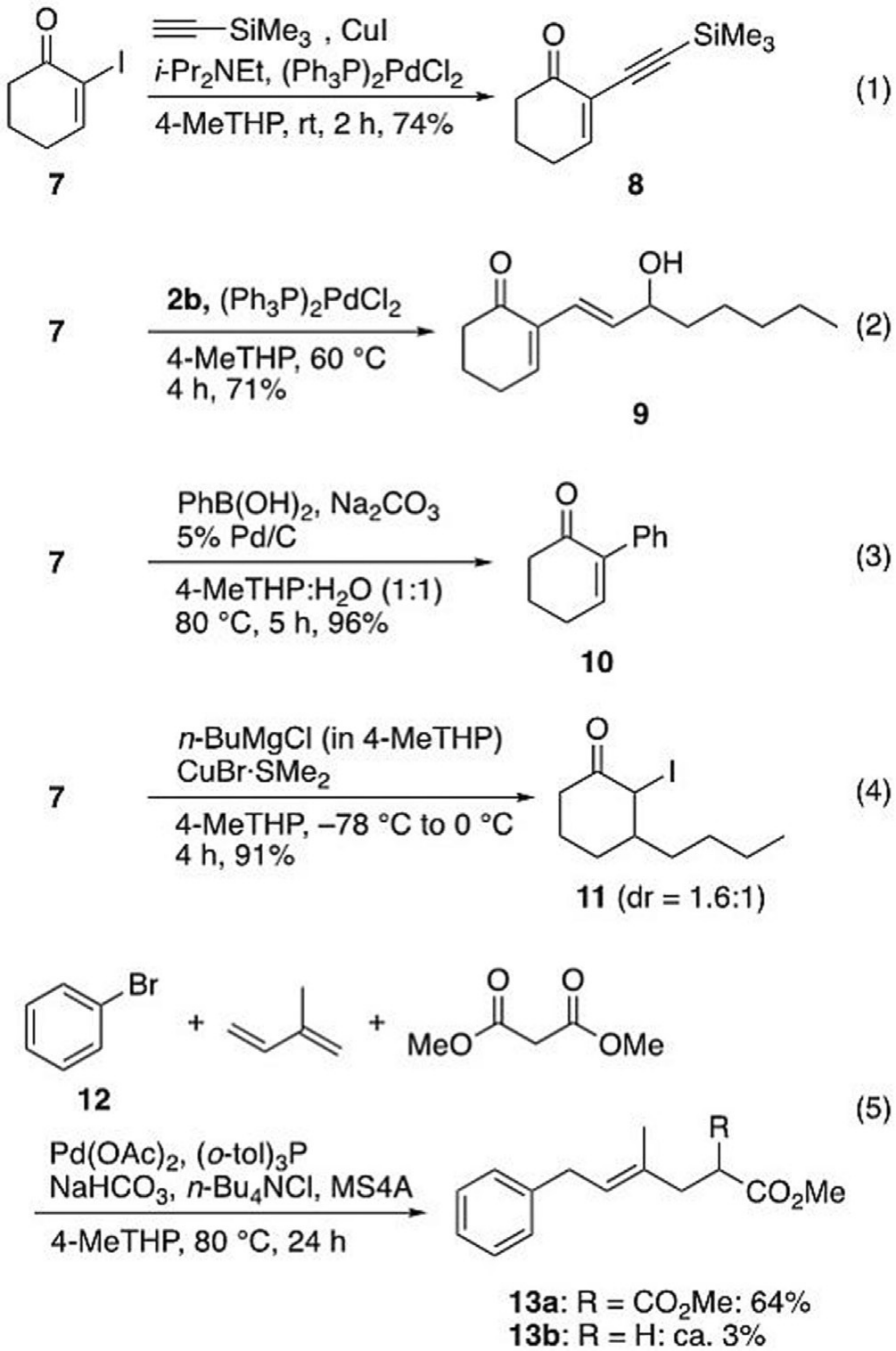
Coupling reactions in 4‐MeTHP.

After evaluating the potential of 4‐MeTHP in Grignard reactions, we undertook its utility in transition metal‐catalyzed coupling reactions with organometallic reagents. As shown in Scheme 7, conventional coupling reactions such as Sonogashira,^41^, ^42^ Stille,^17^, ^43^ and Suzuki coupling^44^, ^45^ of α‐iodoenone **7** progressed in 71–96 % yields in 4‐MeTHP [Scheme 7, Eq. (1)–(3)]. Moreover, the conjugate addition of *n*BuMgCl (prepared in 4‐MeTHP) in the presence of a catalytic amount of CuBr⋅SMe_2_ afforded β‐butylated ketone **11** in 91 % yield, demonstrating the utility of the stable Grignard reagent in 4‐MeTHP [Eq. (4)]. A remarkable example is the three component coupling reaction of bromobenzene (**12**), isoprene and methyl malonate, which was originally reported by Shimizu et al. in their synthetic study of mycophenolic acid.^46^ The original conditions were only found applicable to aryl iodides (not aryl bromides) using DMSO as a reaction solvent, and we therefore searched for alternative conditions. After extensive investigation of palladium catalysts, ligands, bases, phase transfer catalysts, and solvents, we found that the conditions shown in Equation (5) using 4‐MeTHP as the solvent gave rise to the coupling product **13 a** in 64 % yield together with a small amount of the decarboxylated product **13 b** (ca. 3 % yield). When DMF was used instead of 4‐MeTHP, a significant decrease in yield of **13 a** was observed (ca. 10 % yield). Addition of molecular sieves was found effective under our conditions [Eq. (5)].^47^

### Scope and limitation of 4‐MeTHP in miscellaneous organic reactions

**Scheme 8:**
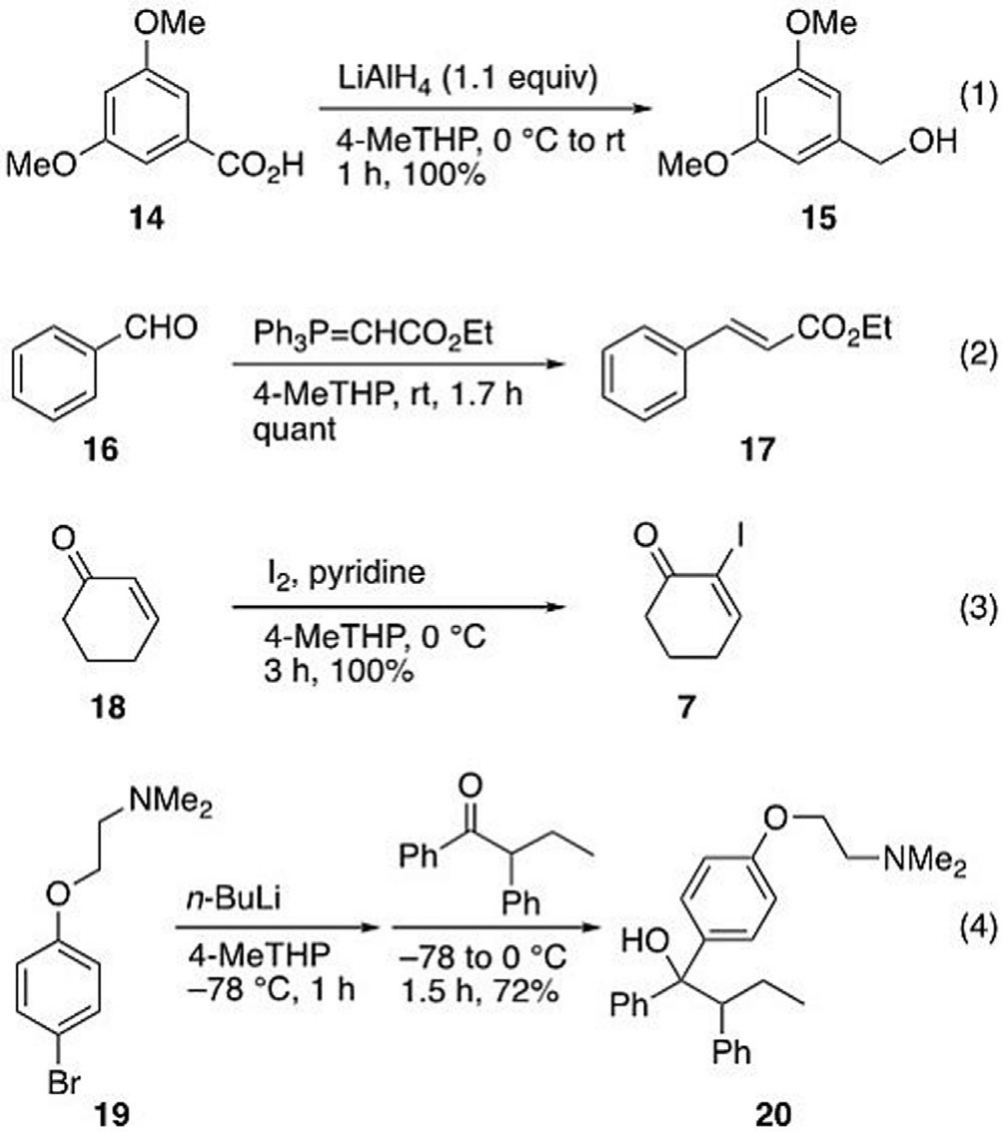
Use of 4‐MeTHP as a substitute for THF.

As mentioned thus far, 4‐MeTHP was found to be a good substitute for conventional solvents in a variety of organic reactions. Scheme 8 represents a set of reactions with 4‐MeTHP where THF is traditionally employed. A remarkable example is the synthesis of a key intermediate of tamoxifen (**20**), a pioneering medicine for estrogen‐receptor‐positive breast cancer, through organolithium addition [Eq. (4)].^48^, ^49^ In this reaction, bromine‐lithium exchange between aryl bromide **19** and *n*BuLi progressed smoothly at −78 °C and gave tertiary alcohol **20**, upon addition of ketone, in 72 % isolated yield after recrystallization. It is notable that a co‐solvent, for example, THF, was unnecessary for the complete halogen‐lithium exchange, which represents the clear advantage of 4‐MeTHP over using CPME/THF mixtures.^18^

**Scheme 9:**
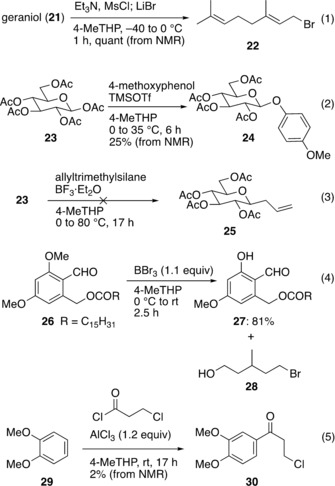
Inappropriate use of 4‐MeTHP.

During our investigations into the adoption of 4‐MeTHP as a solvent, several difficulties were encountered; these are listed in Scheme 9. For the bromination of geraniol (**21**), although the crude NMR spectrum (after extraction and evaporation) indicated full conversion to geranyl bromide (**22**), the complete removal of the solvent without loss of the volatile product was found difficult due to the relatively high boiling point of 4‐MeTHP (105 °C). Not surprisingly, 4‐MeTHP should not be used for reactions and purification of volatile materials such as in the flavor and fragrance industries. Another unsatisfactory example is glycosylation reactions with Lewis acid promoters. For instance, the anomeric *O*‐arylation of pentaacetyl glucose (**23**) with 4‐methoxyphenol^50^ using TMSOTf as an activator resulted in incomplete formation of the glycoside **24** (25 % yield) and the starting material **23** largely remained unreacted (60 % recovery) [Eq. (2)]. Likewise, the allylation of **23** with allyltrimethylsilane and BF⋅Et_2_O^51^ did not furnish the allylation product **25** [Eq. (3)]. In contrast, **24** or **25** could be obtained in high yields when using dichloromethane (DCM) or CH_3_CN, respectively. It is likely that the strong coordination of the oxygen lone pairs in 4‐MeTHP to the silicon or boron results in deactivation of the Lewis acids. The β‐selectivity observed in the *O*‐arylation of **23** was the same as DCM. Another unfavorable result occurred in the regioselective cleavage of phenolic methyl ether **26** by using BBr_3_ [Eq. (4)]. In this reaction, degradation of 4‐MeTHP competed and 5‐bromo‐3‐methylpentan‐1‐ol (**28**) was detected in considerable amounts.^52^ A quantitative formation of **28** was further confirmed by treating 4‐MeTHP and BBr_3_ for 24 h at room temperature in the absence of **26**. Furthermore, we surveyed Friedel–Crafts acylation of 1,2‐dimethoxybenzene (**29**) and 3‐chloropropanoyl chloride using AlCl_3_. The reaction was quite slow and gave the product **30** in only 2 % yield after 17 h (as detected by crude NMR), while the corresponding reaction in DCM completed within 1 h at room temperature with an isolated yield of 95 %. When the reaction mixture was heated at 60 °C, the product **30** disappeared with the formation of unknown byproducts. These examples clearly suggest that 4‐MeTHP is incompatible with strong Lewis acids, in spite of a report that it is stable to the exposure of AlCl_3_.^23^

### Applications of 4‐MeTHP as a substitute for halogenated solvents

**Scheme 10:**
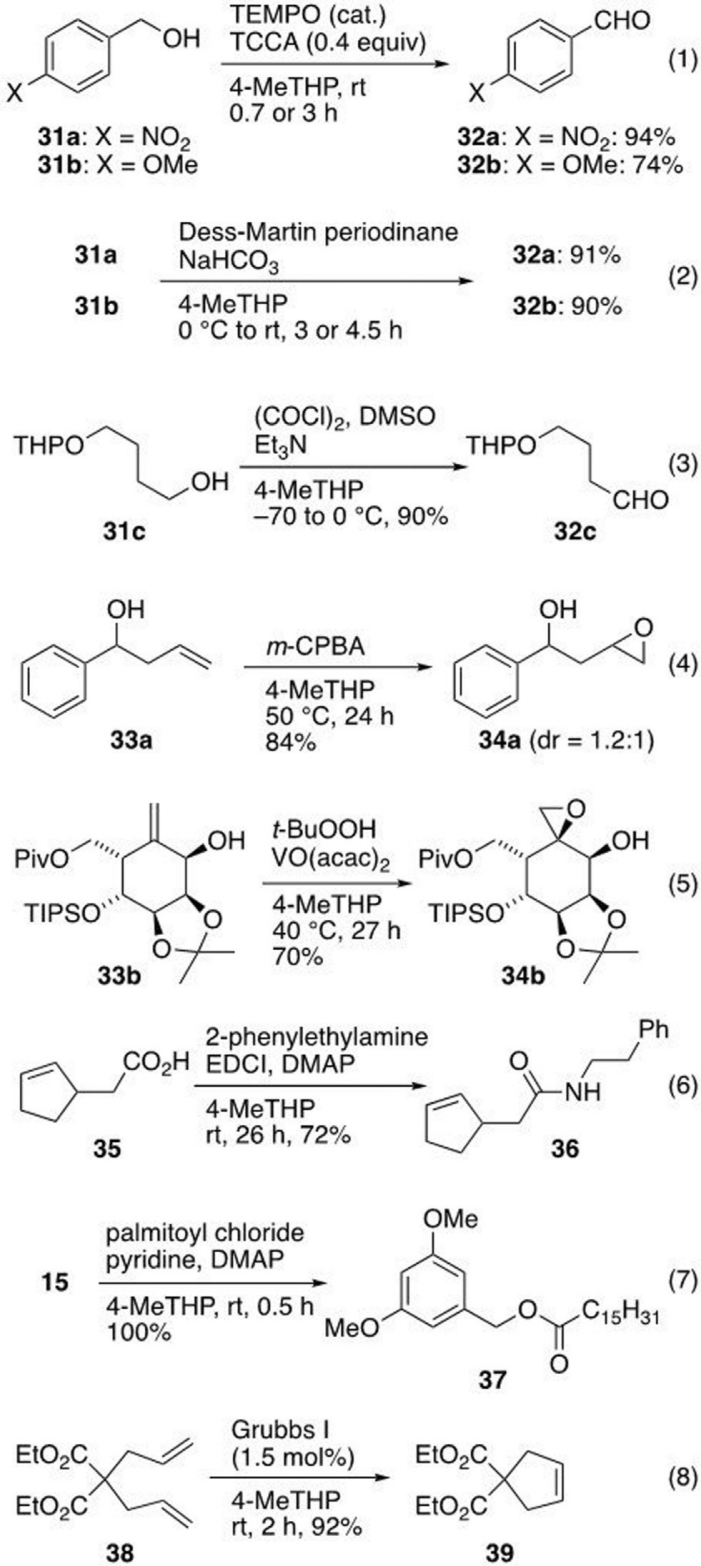
Use of 4‐MeTHP as a substitute for DCM.

The replacement of halogen‐based solvents with greener solvents is highly desirable due to their inherent harmful natures.^3^, ^4^, ^6^, ^7^ However, these solvents are still commonly and widely used in many laboratories because there are no alternatives effective enough for certain reaction cases. Even in recent industrial processes to make pharmaceuticals, DCM is still occasionally used.^53^, ^54^ We therefore studied the application of 4‐MeTHP in reactions where DCM is typically adopted, specifically, in oxidation, epoxidation, condensation, and olefin metathesis reactions (Scheme 10).

As anticipated, trichloroisocyanuric acid (TCCA)‐mediated oxidation,^55^ Dess–Martin oxidation,^56^ and Swern oxidation^57^ of alcohols (**31 a**‐**c**) progressed in more than 74 % yield [Eq. (1)–(3)]. Moreover, the epoxidation of allylic alcohol **33 a** with *m‐*CPBA^58^ afforded epoxide **34 a** in 84 % yield, although the reaction was slower than when using DCM and required 50 °C to complete [Eq. (4)]. It is notable that diastereoselective epoxidation was achieved with the congested allylic alcohol **33 b**, providing β‐epoxyalcohol **34 b** in 70 % yield [Eq. (5)].^59^ In the case of the Dess–Martin oxidation and the two epoxidations, trace amounts of solvent‐derived by‐products were detected by ^1^H NMR,^60^ while only 1.2 equivalents of oxidants were sufficient to convert all starting materials. Further applications to the condensation reactions, such as amidation^61^ of acid **35** with 2‐phenylethylamine and esterification of alcohol **15** with palmitoyl chloride, demonstrated the usefulness of 4‐MeTHP in polar reactions [Eqs. (6) and (7)]. To our knowledge, these condensation reactions are usually carried out in halogenated or aprotic polar solvent probably due to high solubility of organic salt intermediates. The last example is the ring‐closing metathesis (RCM)^62^ of diene **38**, giving the cyclized product **39** in 92 % yield. RCM has now become one of the most applicable methods for providing various carbocycles and heterocycles, and it is widely used in pharmaceutical development,^63^ but normally in DCM or toluene. Although the generality remains to be confirmed, the example shown in Equation (8) indicates the applicability of 4‐MeTHP in RCM chemistry. Overall, 4‐MeTHP appears to be a prime solvent to consider as an alternative for DCM in process chemistry.

## Conclusions

In conclusion, we surveyed the fundamental properties of 4‐MeTHP as a green organic reaction solvent. For radical reactions, 4‐MeTHP showed better stability than 2‐MeTHF and was efficiently recycled without loss of purity. For the Grignard reactions, 4‐MeTHP exhibited superior performance than CPME, which led to the successful formation of both unstable and more challenging Grignard reagents. Further applications to couplings, organometallics, reductions, Wittigs, and halogen‐metal exchanges demonstrated the potential of 4‐MeTHP as a substitute for conventional ethers. Notably, 4‐MeTHP can be used for oxidation, epoxidation, amidation, esterification, and ring‐closing metathesis reactions, and showed high potential to replace harmful halogenated solvents. Nevertheless, 4‐MeTHP was found incompatible with Lewis acids, and the C−O bond can be readily cleaved by treatment with strong Lewis acids like BBr_3_. Besides its synthetic utility, the radical‐based degradation pathways of 4‐MeTHP, THP and 2‐MeTHF were elucidated on the basis of GC and GC‐MS analyses. To the best of our knowledge, this is the first experimental evidence on the mechanisms of radical‐based degradations of above cyclic ether solvents, although it is well known that the corresponding peroxides tend to form after prolonged storage of cyclic ethers^16^ These comprehensive studies are anticipated to find wide use for a broad range of synthetic chemists, especially process chemists in industrial settings, where the selection of a reaction solvent with green chemistry perspectives is of high importance and consequence.

## Experimental Section

### General Techniques

All reactions utilizing air‐ or moisture‐sensitive reagents were performed under an atmosphere of argon. Commercially available dry solvents were used for THF and DCM. 4‐MeTHP, 2‐MeTHF, MeCN and DMSO were dried over molecular sieves prior to use. Pyridine and Et_3_N were distilled from CaH_2_ and dried over molecular sieves. *n*Bu_3_SnH was distilled under reduced pressure. Other chemicals were used as received. Reactions were monitored by thin‐layer chromatography (TLC) carried out on 0.25 mm silica gel plates (60‐F254) that were analyzed by fluorescence upon 254 nm irradiation or by staining with *p‐*anisaldeyde/AcOH/H_2_SO_4_/EtOH, 12 MoO_3_⋅H_3_PO_4_/EtOH, or (NH_4_)_6_Mo_7_O_24_⋅4 H_2_O/H_2_SO_4_. The products were purified by either open chromatography on silica gel (spherical, neutral, 70–230 μm) or flash chromatography on silica gel (spherical, neutral, 40–50 μm) and, if necessary, HPLC equipped with a pre‐packed column with an eluent of *n*‐hexane/EtOAc. NMR spectra were recorded with a 300 MHz (^1^H: 300 MHz, ^13^C: 75 MHz) or a 400 MHz (^1^H: 400 MHz, ^13^C: 100 MHz) spectrometer and referenced to the solvent peak at 7.26 ppm (^1^H) and 77.16 ppm (^13^C) for CDCl_3_. Splitting patterns are indicated as follows: br, broad; s, singlet; d, doublet; t, triplet; q, quartet; qui, quintet; m, multiplet. Infrared spectra were recorded with a FT/IR spectrometer and reported as wavenumber (cm^−1^). High‐resolution atmospheric pressure chemical ionization (APCI) and electron spray ionization (ESI) mass spectra were recorded with an Orbitrap analyzer in positive or negative ion mode.

### Radical addition, reduction, and cyclization

**5‐(Tributylstannyl)pent‐4‐en‐1‐ol (2 aa).^[17]^** A solution of 4‐pentyn‐1‐ol (**1 a**) (168 mg, 2.00 mmol), AIBN (65.6 mg, 0.400 mmol), and *n*Bu_3_SnH (756 mg, 2.60 mmol) in 4‐MeTHP (6.6 mL) was stirred at 90 °C for 2 h. The reaction mixture was concentrated, and the residue was purified by open chromatography on silica gel (*n*‐hexane/EtOAc/Et_3_N, 30:1:0.3→10:1:0.1 v/v) to give stannane **2 aa** (710 mg, 1.89 mmol, 95 %) as an inseparable 4:1 *E/Z* mixture. Pale yellow oil; The following assignments were made with an inseparable mixture of stereoisomers. Data for *E*‐isomer: ^1^H NMR (300 MHz, CDCl_3_): *δ=*6.02–5.89 (m, 2 H), 3.66 (dd, *J=*6.5, 5.6 Hz, 2 H), 2.26–2.19 (m, 2 H), 1.73–1.64 (m, 2 H), 1.55–1.43 (m, 6 H), 1.36–1.22 (m, 6 H), 0.97–0.77 ppm (m, 15 H); ^13^C NMR (75 MHz, CDCl_3_): *δ=*148.8, 128.3, 62.6, 34.2, 32.0, 29.2, 27.4, 13.8, 9.5 ppm. Data for *Z*‐isomer: ^1^H NMR (300 MHz, CDCl_3_): *δ=*6.53 (dt, *J=*12.3, 7.0 Hz, 1 H), 5.83 (dt, *J=*12.3, 1.1 Hz, 1 H), 3.67 (dd, *J=*6.5, 5.6 Hz, 2 H), 2.15–2.07 (m, 2 H), 1.73–1.64 (m, 2 H), 1.55–1.43 (m, 6 H), 1.36–1.22 (m, 6 H), 0.97–0.77 ppm (m, 15 H); ^13^C NMR (75 MHz, CDCl_3_): *δ=*148,4, 128.9, 62.8, 33.5, 33.0, 29.2, 27.4, 13.8, 10.4 ppm.

**5‐(1,1,1,3,3,3‐Hexamethyl‐2‐(trimethylsilyl)trisilan‐2‐yl)pent‐4‐en‐1‐ol (2 ab).^[17]^** A solution of alkyne **1 a** (83.9 mg, 1.00 mmol), (TMS)_3_SiH (323 mg, 1.30 mmol), and V‐65 (49.7 mg, 0.200 mmol) in 4‐MeTHP (3.3 mL) was stirred at 70 °C for 4 h. The reaction mixture was concentrated, and the residue was purified by flash chromatography on silica gel (*n*‐hexane/EtOAc/Et_3_N, 30:1:0.3→10:1:0.1 v/v) to give silane **2 ab** (309 mg, 0.929 mmol, 93 %) as an inseparable 1:3.2 *E/Z* mixture. Pale yellow oil; The following assignments were made with an inseparable mixture of stereoisomers. Data for *E*‐isomer: ^1^H NMR (400 MHz, CDCl_3_): *δ=*6.00 (dt, *J=*18, 6.4 Hz, 1 H), 5.56 (dt, *J=*18, 1.2 Hz, 1 H), 3.66 (t, *J=*6.4 Hz, 2 H), 2.23–2.13 (m, 2 H), 1.71–1.63 (m, 2 H), 0.16 ppm (s, 27 H); ^13^C NMR (75 MHz, CDCl_3_): *δ=*148.3, 121.9, 62.6, 34.0, 32.3, 1.0 ppm. Data for *Z*‐isomer: ^1^H NMR (400 MHz, CDCl_3_): *δ=*6.39 (dt, *J=*13, 7.0 Hz, 1 H), 5.54 (dt, *J=*13, 1.2 Hz, 1 H), 3.69 (t, *J=*6.8 Hz, 2 H), 2.23–2.13 (m, 2 H), 1.71–1.63 (m, 2 H), 0.18 ppm (s, 27 H); ^13^C NMR (75 MHz, CDCl_3_): *δ=*148.3, 121.1, 62.9, 32.9, 31.9, 1.3 ppm.

**1‐(Tributylstannyl)oct‐1‐en‐3‐ol (2 b).^[64]^** A solution of oct‐1‐yn‐3‐ol (**1 b)** (126 mg, 1.00 mmol), AIBN (33.0 mg, 0.201 mmol), and *n*Bu_3_SnH (379 mg, 1.30 mmol) in 4‐MeTHP (3.3 mL) was stirred at 90 °C for 30 min. The reaction mixture was concentrated, and the residue was purified by flash chromatography on silica gel (*n*‐hexane/Et_3_N, 100:1→*n*‐hexane/EtOAc/Et_3_N, 50:1:0.5 v/v) to give stannane **2 b** (340 mg, 0.816 mmol, 82 %) as an inseparable 10:1 *E/Z* mixture. The ^1^H NMR indicated contamination of a regioisomer (ca. 3 %). Pale yellow oil; The following assignments were made with an inseparable mixture of stereoisomers. Data for *E*‐isomer: ^1^H NMR (400 MHz, CDCl_3_): *δ=*6.12 (dd, *J=*19, 0.8 Hz, 1 H), 5.99 (dd, *J=*19, 5.6 Hz, 1 H), 4.06 (m, 1 H), 1.68–1.26 (m, 20 H), 0.97–0.80 ppm (m, 18 H); ^13^C NMR (75 MHz, CDCl_3_): *δ=*151.4, 127.7, 75.8, 37.1, 32.0, 29.2, 27.4, 25.2, 22.8, 14.1, 13.8, 9.6 ppm. Data for *Z*‐isomer: ^1^H NMR (400 MHz, CDCl_3_): *δ=*6.49 (dd, *J=*13, 7.6 Hz, 1 H), 6.00 (dd, *J=*13, 0.9 Hz, 1 H), 3.89 (m, 1 H), 1.68–1.26 (m, 20 H), 0.97–0.80 ppm (m, 18 H).

**Cyclododecane (2 c)**. A solution of *n*Bu_3_SnH (189 mg, 0.650 mmol) and AIBN (16.7 mg, 0.102 mmol) in 4‐MeTHP (1.5 mL) was added to a solution of thiocarbonate **1 c** 17 (160 mg, 0.499 mmol) in 4‐MeTHP (6 mL) at 90 °C. The resultant mixture was stirred at 90 °C for 2 h and concentrated. The residue was purified by flash column chromatography on 10 % w/w K_2_CO_3_‐silica gel (*n*‐hexane) to give cyclododecane (**2 c**) (69.4 mg, 0.412 mmol, 83 %). Colorless waxy solid; ^1^H NMR (400 MHz, CDCl_3_): *δ=*1.33 ppm (m, 24 H); ^13^C NMR (75 MHz, CDCl_3_): *δ=*23.8 ppm. This compound is commercially available. (CAS: 294‐62‐2)

**Cholest‐5‐ene (2 d)**.^16^ A solution of *n*Bu_3_SnH (189 mg, 0.650 mmol) and AIBN (16.7 mg, 0.102 mmol) in 4‐MeTHP (1.5 mL) was added to a solution of thiocarbonate **1 c** 17 (160 mg, 0.499 mmol) in 4‐MeTHP (6 mL) at 90 °C. The reaction mixture was stirred at 90 °C for 24 h and concentrated. The residue was purified by flash column chromatography on 10 % w/w K_2_CO_3_‐silica gel (*n*‐hexane) to give cyclododecane (**2 c**) (69.4 mg, 0.412 mmol, 83 %). Colorless waxy solid; ^1^H NMR (400 MHz, CDCl_3_): *δ=*1.33 ppm (m, 24 H); ^13^C NMR (75 MHz, CDCl_3_): *δ=*23.8 ppm. This compound is commercially available. (CAS: 294‐62‐2)

**(±)‐(3*R*,3a*R*,6a*R*)‐3‐Allylhexahydro‐2*H*‐cyclopenta[*b*]furan‐2‐one (2 e)**. Bromolactone **1 e** 65 (121 mg, 0.590 mmol), allyltributyltin (277 mg, 0.835 mmol), and AIBN (9.3 mg, 0.057 mmol) were placed in a 30 mL round bottom flask, which was dissolved in 4‐MeTHP (5.5 mL). The solution was degassed by three freeze‐pump‐thaw cycles and heated at 90 °C under argon (balloon). After 2 h, the reaction mixture was diluted with 4‐MeTHP (3 mL) and quenched by the addition of saturated aqueous KF (3 mL) at room temperature. The resulting mixture was vigorously stirred for 2 h and extracted Et_2_O (5 mL). The organic layer was washed with brine, dried over anhydrous MgSO_4_, filtered and concentrated. The residue was purified by flash chromatography on 10 % w/w KF‐silica gel (*n*‐hexane/EtOAc, 10:1 v/v) to give lactone **2 e** (74.9 mg, 0.451 mmol, 76 %). Colorless oil; ^1^H NMR (300 MHz, CDCl_3_): *δ=*5.78 (m, 1 H), 5.19–5.11 (m, 2 H), 4.92 (m, 1 H), 2.62 (m, 1 H), 2.53 (m, 1 H), 2.44–2.29 (m, 2 H), 2.01 (m, 1 H), 1.89–1.52 ppm (m, 5 H); ^13^C NMR (75 MHz, CDCl_3_): *δ=*179.6, 134.5, 118.2, 84.8, 47.7, 43.8, 36.3, 33.7, 33.4, 23.6 ppm; IR (film on ZnSe): $\tilde{\nu }$ =3078, 2961, 2872, 1771, 1641, 1439, 1362, 1323, 1182 cm^−1^; HRMS (APCI+): *m*/*z*: calcd for C_10_H_15_O_2_: 167.1067 [*M*+H]^+^; found 167.1066.

**Ethyl 2‐(2‐((1,1,1,3,3,3‐hexamethyl‐2‐(trimethylsilyl)trisilan‐2‐yl)methylene)cyclopentyl)acetate (2 f)**. A solution of alkyne **1 f** (166 mg, 0.998 mmol), (TMS)_3_SiH (359 mg, purity >90.0 %, >1.30 mmol), and V‐65 (49.8 mg, 0.201 mmol) in 4‐MeTHP (3.3 mL) was heated at 70 °C. After 1.5 h, the reaction mixture was concentrated, and the residue was purified by the repeated flash chromatography on silica gel (*n*‐hexane/EtOAc, 50:1 v/v, then *n*‐hexane, *n*‐hexane/EtOAc, 10:1 v/v) to give a cyclized product **2 f** (346 mg, 0.929 mmol, 84 %). Colorless oil; ^1^H NMR (300 MHz, CDCl_3_): *δ=*5.27 (q, *J=*2.2 Hz, 1 H), 4.13 (q, *J=*7.1 Hz, 2 H), 2.81 (m, 1 H), 2.55 (dd, *J=*15, 5.3 Hz, 1 H), 2.33–2.17 (m, 2 H), 2.20 (dd, *J=*15, 9.5 Hz, 1 H), 1.97 (m, 1 H), 1.75 (m, 1 H), 1.59 (m, 1 H), 1.34 (m, 1 H), 1.26 (t, *J=*7.1 Hz, 3 H), 0.16 ppm (s, 27 H); ^13^C NMR (75 MHz, CDCl_3_): *δ=*173.3, 164.6, 109.5, 60.4, 43.5, 40.0, 34.9, 32.9, 24.4, 14.4, 1.3 ppm; IR (film on ZnSe): $\tilde{\nu }$ =2949, 2893, 1738, 1612, 1244, 1163, 1032 cm^−1^; HRMS (APCI+): *m*/*z*: calcd for C_19_H_43_O_2_Si_4_: 415.2335 [*M*+H]^+^; found: 415.2334.

### General Procedure for Recycling the Solvent in the Radical Reaction with 1 b and *n*Bu_3_SnH

Commercially available 4‐MeTHP (stabilized with BHT) was stored with molecular sieves 4Å and used to the reaction. Oct‐1‐yn‐3‐ol (**1 b**) (126 mg, 0.996 mmol), *n*Bu_3_SnH (378 mg, 1.30 mmol), and AIBN (32.8 mg, 0.200 mmol) were placed in a 100 mL two‐necked round bottom flask equipped with a reflux condenser. The flask was flushed with argon and 4‐MeTHP (20 mL) was added. After complete dissolution of AIBN at room temperature, the solution was heated at reflux for 30 min. The solution was then cooled to room temperature and, after attachment of the distillation apparatus, the solvent was distillated under reduced pressure (bp 38–40 °C/8–10 kPa). The residual oil was purified by flash chromatography on silica gel (*n*‐hexane/EtOAc/Et_3_N, 50:1:0.5 v/v) to give stannane **2 b** (288 mg, 0.691 mmol, 69 %, *E/Z=*16:1) as a pale yellow oil. The purity of the recovered 4‐MeTHP was determined to be 99.94 % by the GC analysis. The same experiment was performed with the recovered 4‐MeTHP, providing the product **2 b** (64 % yield, *E/Z=*8:1) with a recovery of 4‐MeTHP (purity: 99.88 %).

### Gas Chromatography Analysis of the Recovered Solvent

Gas‐phase chromatography was performed on a GC‐2014 instrument using a DB‐WAX column (30 m×0.25 mm×0.25 μm film thickness). A temperature of FID detector was adjusted to 250 °C. The oven was heated at 50 °C for 5 min followed by a temperature gradient of 10 °C min^−1^ to 200 °C and, finally, at 200 °C for 5 min. Inlet temperature and pressure were 200 °C and 83 kPa, respectively, with a split ratio of 100:1. Helium was used as the carrier gas at a flow rate of 1.0 mL min^−1^.

### Gas Chromatography‐Mass Spectrometry Analysis of the Recovered Solvent

The gas chromatography‐mass spectrometry model GCMS‐QP2010 Ultra (SHIMADZU CORPORATON, Japan), equipped with DB‐WAX capillary column with dimensions 30 m×0.25 mm×0.25 μm, was used. The oven was maintained at 50 °C for 5 min followed by a temperature gradient of 10 °C min^−1^ to 200 °C and, finally, at 200 °C for 5 min. The injection volume of the sample was 0.2 μL with a split ratio of 100:1, using helium as the carrier gas at a flow rate of 1.0 mL min^−1^. Injector temperature was maintained at 200 °C. Detector temperature was maintained at 230 °C. The percentage composition was calculated using peak normalization method assuming equal detector response. The samples were analyzed with an electron impact ionization at 70 eV. The compounds separated were characterized from their mass spectral data using the NIST14 mass spectral library.

### General Procedure for the Radical Reaction with 2‐Phenylethanol and BrCCl_3_

Commercially available 4‐MeTHP, THF and 2‐MeTHF were distilled from sodium/benzophenone just prior to the reaction. 2‐Phenylethanol (600 μL, 5.00 mmol), BrCCl_3_ (1.35 mL, 15.0 mmol) and 2,4,6‐collidine (650 μL, 5.00 mmol) were placed in a two‐necked round bottom flask equipped with a reflux condenser. The distilled 4‐MeTHP (15 mL) was added over 6 h using a syringe pump at 60 °C, and the resulting mixture was additionally stirred at 60 °C for 11 h. The reaction mixture was quenched by the addition of saturated aqueous NH_4_Cl and extracted with *n*‐hexane (2×15 mL). The combined organic layer was washed with water, brine, dried over anhydrous MgSO_4_, filtered and concentrated. The residue was purified by flash chromatography on silica gel (*n*‐hexane/EtOAc, 100:1 v/v) to give fractions containing **4 a** and diphenethyl carbonate. The calculated yield of **4 a** based on the NMR analysis was 14 % (155 mg, 0.704 mmol). The analytical sample was obtained by normal‐phase HPLC equipped with a prepacked column [Mightysil, Si 60 250‐20 (5 μm)] using *n*‐hexane/EtOAc (87:13 v/v) as an eluent.

**4‐Methyl‐2‐phenethoxytetrahydro‐2*H*‐pyran (4 a)**. Colorless oil; ^1^H NMR (400 MHz, CDCl_3_): *δ=*7.13–7.18 (m, 5 H), 4.80 (br d, *J=*3.0 Hz, 1 H), 3.87 (dt, *J=*9.7, 7.2 Hz, 1 H), 3.64–3.56 (m, 2 H), 3.51 (ddd, *J=*11, 5.1, 1.8 Hz, 1 H), 2.90 (t, *J=*7.2 Hz, 2 H), 1.93 (m, 1 H), 1.70 (m, 1 H), 1.49 (m, 1 H), 1.30–1.18 (m, 2 H), 0.88 ppm (d, *J=*6.6 Hz, 3 H); ^13^C NMR (100 MHz, CDCl_3_): *δ=*139.4, 129.1, 128.4, 126.3, 97.1, 67.8, 59.8, 38.8, 36.5, 34.3, 24.3, 22.4 ppm; IR (film on ZnSe): $\tilde{\nu }$ =3063, 3028, 2951, 2926, 2870, 1497, 1456, 1387, 1341, 1258, 1184, 1165 cm^−1^; HRMS (APCI+): *m*/*z*: calcd for C_14_H_20_O_2_Na: 243.1356 [*M*+Na]^+^; found: 243.1359.

**2‐Phenethoxytetrahydrofuran (4 b)**. This compound was obtained in 65 % yield by using THF as a reactant. The analytical sample was obtained by normal‐phase HPLC equipped with a prepacked column [Mightysil, Si 60 250‐20 (5 μm)] using *n*‐hexane/EtOAc (87:13 v/v) as an eluent. Colorless oil; ^1^H NMR (400 MHz, CDCl_3_): *δ=*7.30–7.18 (m, 5 H), 5.12 (dd, *J=*3.9, 2.1 Hz, 1 H), 3.88 (dt, *J=*10, 7.2 Hz, 1 H), 3.86–3.81 (m, 2 H), 3.61 (dt, *J=*10, 7.2 Hz, 1 H), 2.88 (t, *J=*7.2 Hz, 2 H), 2.02–1.77 ppm (m, 4 H); ^13^C NMR (100 MHz, CDCl_3_): *δ=*139.2, 129.1, 128.4, 126.2, 103.9, 68.0, 67.0, 36.5, 32.5, 23.6 ppm; IR (film on ZnSe): $\tilde{\nu }$ =3086, 3063, 3028, 2949, 2909, 2880, 1605, 1497, 1454, 1348, 1184 cm^−1^; HRMS (APCI+): *m*/*z*: calcd for C_12_H_17_O_2_: 193.1223 [*M*+H]^+^; found: 193.1228.

**2‐Methyl‐5‐phenethoxytetrahydrofuran (4 c)**. This compound was obtained in 30 % yield with a 2:1 diastereoselectivity by using 2‐MeTHF as a reactant. Two diastereomers were separated by normal‐phase HPLC equipped with a prepacked column [Mightysil, Si 60 250‐20 (5 μm)] using *n*‐hexane/EtOAc (89:11 v/v) as an eluent. Data for major isomer: Colorless oil; ^1^H NMR (400 MHz, CDCl_3_): *δ=*7.30–7.17 (m, 5 H), 5.12 (dd, *J=*4.9, 1.9 Hz, 1 H), 4.12 (m, 1 H), 3.90 (dt, *J=*9.8, 7.2 Hz, 1 H), 3.61 (dt, *J=*9.8, 7.2 Hz, 1 H), 2.88 (t, *J=*7.2 Hz, 2 H), 2.11–2.02 (m, 2 H), 1.84 (m, 1 H), 1.36 (m, 1 H), 1.21 ppm (d, *J=*6.4 Hz, 3 H); ^13^C NMR (100 MHz, CDCl_3_): *δ=*139.2, 129.1, 128.4, 126.2, 104.2, 74.0, 68.2, 36.5, 32.6, 31.3, 21.0 ppm; IR (film on ZnSe): $\tilde{\nu }$ =3063, 3028, 2968, 2907, 2868, 1497, 1454, 1383, 1339, 1190 cm^−1^; HRMS (APCI+): *m*/*z*: calcd for C_13_H_18_O_2_Na: 229.1199 [*M*+Na]^+^; found: 229.1201. Data for minor isomer: Colorless oil; ^1^H NMR (400 MHz, CDCl_3_): *δ=*7.30–7.17 (m, 5 H), 5.04 (d, *J=*4.6 Hz, 1 H), 4.15 (m, 1 H), 3.90 (dt, *J=*9.6, 7.2 Hz, 1 H), 3.58 (dt, *J=*9.6, 7.2 Hz), 2.88 (t, *J=*7.2 Hz, 2 H), 2.00–1.84 (m, 3 H), 1.63 (m, 1 H), 1.22 ppm (d, *J=*6.0 Hz, 3 H); ^13^C NMR (100 MHz, CDCl_3_): *δ=*139.3, 129.1, 128.4, 126.2, 103.9, 76.7, 67.7, 36.5, 33.8, 31.2, 23.0 ppm; IR (film on ZnSe): $\tilde{\nu }$ =3063, 3028, 2968, 2928, 1497, 1454, 1377, 1346, 1198 cm^−1^; HRMS (APCI+): *m*/*z*: calcd for C_13_H_18_O_2_Na: 229.1199 [*M*+Na]^+^; found: 229.1204.

### Grignard Reactions in 4‐MeTHP

**(4‐Methoxyphenyl)(phenyl)methanol (6 a).^[18]^** Well‐ground magnesium turnings (365 mg, 15.0 mmol) were placed in a well‐dried three‐necked round bottom flask equipped with a reflux condenser and a dropping funnel. The flask was further dried under reduced pressure with a heat gun and then flushed with argon, after which 4‐MeTHP (2 mL) and DIBALH (1.0 M solution in *n*‐hexane, 75 μL, 75 μmol) were added. The suspension was stirred for 30 min at room temperature and warmed to 60 °C. A solution of 4‐bromoanisole (**5 a**) (1.87 g, 9.99 mmol) in 4‐MeTHP (3 mL+3 mL for rinse) was added through the dropping funnel over 50 min. The reaction mixture was stirred at 60 °C for 3 h in total and cooled with an ice bath. A solution of benzaldehyde (1.06 g, 10.0 mmol) in 4‐MeTHP (15 mL) was added and the mixture was stirred for 1 h at room temperature. The reaction mixture was quenched by the addition of saturated aqueous NH_4_Cl at 0 °C and extracted with Et_2_O (3×15 mL). The combined organic layer was washed with brine, dried over anhydrous MgSO_4_, filtered and concentrated. The residue was purified by open chromatography on silica gel (*n*‐hexane/EtOAc=100:1→5:1 v/v) to give alcohol **6 a** (1.60 g, 7.47 mmol, 75 %). Colorless solid; ^1^H NMR (400 MHz, CDCl_3_): *δ=*7.40–7.26 (m, 7 H), 6.87 (d, *J=*8.8 Hz, 2 H), 5.81 (s, 1 H), 3.79 ppm (s, 3 H); ^13^C NMR (100 MHz, CDCl_3_): *δ=*159.2, 144.1, 136.3, 128.6, 128.0, 127.6, 126.5, 114.0, 75.9, 55.4 ppm.

**(2‐Chlorophenyl)(phenyl)methanol (6 b).^[66]^** Well‐ground magnesium turnings (365 mg, 15.0 mmol) were placed in a well‐dried 100 mL three‐necked round bottom flask equipped with a reflux condenser and a dropping funnel. The flask was further dried under reduced pressure with a heat gun and then flushed with argon, after which 4‐MeTHP (2 mL) and DIBALH (1.0 M solution in *n*‐hexane, 75 μL, 75 μmol) were added. The suspension was stirred for 30 min at room temperature and cooled with an ice bath. A solution of 1‐bromo‐2‐chlorobenzene (**5 b**) (1.91 g, 10.0 mmol) in 4‐MeTHP (6 mL+2 mL for rinse) was added through a dropping funnel over 30 min and the reaction mixture was further stirred at 0 °C for 2 h. The solution was then cooled to −78 °C followed by the addition of benzaldehyde (1.06 g, 9.98 mmol) in 4‐MeTHP (10 mL+4 mL for rinse). The reaction mixture was stirred at −78 °C for 1.2 h and warmed to 0 °C. After 5 min, the reaction mixture was quenched by the addition of saturated aqueous NH_4_Cl and extracted with EtOAc (3×15 mL). The combined organic layer was washed with brine, dried over anhydrous MgSO_4_, filtered and concentrated. The residue was purified by open chromatography on silica gel (*n*‐hexane/EtOAc, 200:1→5:1 v/v) to give alcohol **6 b** (1.43 g, 6.54 mmol, 66 %). Colorless solid; ^1^H NMR (400 MHz, CDCl_3_): *δ=*7.61 (dd, *J=*7.6, 1.8 Hz, 1 H), 7.42–7.21 (m, 8 H), 6.24 ppm (s, 1 H); ^13^C NMR (100 MHz, CDCl_3_): *δ=*142.3, 141.1, 132.6, 129.6, 128.9, 128.6, 128.1, 127.9, 127.2, 127.0, 72.7 ppm.

**(2‐Fluorophenyl)(phenyl)methanol (6 c).^[66]^** Well‐ground magnesium turnings (365 mg, 15.0 mmol) were placed in a well‐dried 100 mL three‐necked round bottom flask equipped with a reflux condenser and a dropping funnel. The flask was further dried under reduced pressure with a heat gun and flushed with argon, after which 4‐MeTHP (2 mL) and DIBALH (1.0 m solution in *n*‐hexane, 75 μL, 75 μmol) were added. The suspension was stirred for 30 min at room temperature and then cooled with an ice bath. A solution of 1‐bromo‐2‐fluorobenzene (**5 c**) (1.75 g, 10.0 mmol) in 4‐MeTHP (5 mL+2 mL for rinse) was added through a dropping funnel over 30 min and the resulting mixture was stirred for 2 h at 0 °C. The reaction mixture was then cooled to −78 °C followed by the addition of benzaldehyde (1.07 g, 10.0 mmol) in 4‐MeTHP (5 mL+3 mL for rinse). The resulting mixture was stirred at −78 °C for 1 h and warmed to 0 °C. After 3 min, the reaction mixture was quenched by the addition of saturated aqueous NH_4_Cl and saturated aqueous Rochelle salt, and extracted with EtOAc (3×15 mL). The combined organic layer was washed with brine, dried over anhydrous MgSO_4_, filtered and concentrated. The residue was purified by open chromatography on silica gel (*n*‐hexane/EtOAc, 100:1→50:1→5:1 v/v) to give alcohol **6 c** (1.43 g, 7.67 mmol, 77 %). Colorless oil; ^1^H NMR (300 MHz, CDCl_3_): *δ=*7.52 (td, *J=*7.5, 1.8 Hz, 1 H), 7.43–7.23 (m, 6 H), 7.16 (td, *J=*7.5, 1.2 Hz, 1 H), 7.03 (m, 1 H), 6.15 (d, *J=*6.3 Hz, 1 H), 2.42 ppm (br s, 1 H, OH); ^13^C NMR (75 MHz, CDCl_3_): *δ=*160.1 (d, *J=*246 Hz), 142.9, 131.1 (d, *J=*12 Hz), 129.3 (d, *J=*8.3 Hz), 128.6, 127.9, 126.5, 124.4 (d, *J=*3.5 Hz), 115.5 (d, *J=*21 Hz), 70.3 ppm.

**1‐Phenylbut‐3‐yn‐1‐ol (6 d).^[67]^** Well‐ground magnesium turnings (365 mg, 15.0 mmol) were placed in a well‐dried 300 mL three‐necked round bottom flask equipped with a reflux condenser and a dropping funnel. The flask was further dried under reduced pressure with a heat gun followed by the addition of HgCl_2_ (13.5 mg, 0.0497 mmol). The flask was flushed with argon followed by the addition of 4‐MeTHP (2 mL) and DIBALH (1.0 M solution in *n*‐hexane, 75 μL, 75 μmol). The suspension was stirred for 30 min at room temperature and cooled to 0 °C. A solution of 3‐bromoprop‐1‐yne (**5 d**) (1.19 g, 10.0 mmol) in 4‐MeTHP (3 mL+3 mL for rinse) was added through a dropping funnel over 1 h and the resulting mixture was additionally stirred for 2 h at 0 °C. The reaction mixture was then cooled to −78 °C followed by the addition of benzaldehyde (1.06 g, 9.98 mmol) in 4‐MeTHP (15 mL). The resulting mixture was stirred for 1 h at −78 °C and quenched by the addition of saturated aqueous NH_4_Cl. The resulting mixture was extracted with EtOAc (3×15 mL), and the combined organic layer was washed with brine, dried over anhydrous MgSO_4_, filtered and concentrated. The residue was purified by open chromatography on silica gel (*n*‐hexane/EtOAc, 150:1→5:1 v/v) to give alcohol **6 d** (680 mg, 4.65 mmol, 47 %). Yellow oil; ^1^H NMR (400 MHz, CDCl_3_): *δ=*7.40–7.29 (m, 5 H), 4.83 (t, *J=*6.4 Hz, 1 H), 2.88 (br s, 1 H), 2.62 (dd, *J=*6.4, 2.6 Hz, 2 H), 2.07 ppm (t, *J=*2.6 Hz, 1 H); ^13^C NMR (100 MHz, CDCl_3_): *δ=*142.5, 128.4, 127.9, 125.8, 80.8, 72.3, 71.0, 29.3 ppm.

**Diphenylmethanol (6 e).^[18]^** Well‐ground magnesium turnings (365 mg, 15.0 mmol) were placed in a well‐dried 100 mL three‐necked round bottom flask equipped with a reflux condenser and a dropping funnel. The flask was further dried under reduced pressure with a heat gun and flushed with argon, after which 4‐MeTHP (2 mL) and DIBALH (1.0 m solution in *n*‐hexane, 750 μL, 750 μmol) were added. The suspension was stirred for 30 min at room temperature and warmed to 90 °C. A solution of chlorobenzene (**5 e**) (1.13 g, 10.0 mmol) in 4‐MeTHP (3 mL+3 mL for rinse) was added through a dropping funnel over 20 min and the mixture was stirred at 90 °C for 20 h in total. The mixture was cooled to 0 °C followed by the addition of benzaldehyde (1.06 g, 10.0 mmol) in 4‐MeTHP (15 mL). The resultant mixture was stirred for 1 h at room temperature and quenched at 0 °C by the addition of saturated aqueous NH_4_Cl and saturated aqueous Rochelle salt. The resulting mixture was extracted with Et_2_O (3×15 mL), and the combined organic layer was washed with brine, dried over anhydrous MgSO_4_, filtered and concentrated. The residue was purified by open chromatography on silica gel (*n*‐hexane/EtOAc, 300:1→100:1→50:1→5:1 v/v) to give alcohol **6 e** (917 mg, 4.98 mmol, 50 %). Colorless solid; ^1^H NMR (400 MHz, CDCl_3_): *δ=*7.41–7.25 (m, 10 H), 5.86 (s, 1 H), 1.62 ppm (br s, 1 H, OH); ^13^C NMR (100 MHz, CDCl_3_): *δ=*143.9, 128.6, 127.7, 126.6, 76.3 ppm.

**Phenyl(*p‐*tolyl)methanol (6 f).^[18]^** Well‐ground magnesium turnings (365 mg, 15.0 mmol) were placed in a well‐dried 100 mL three‐necked round bottom flask equipped with a reflux condenser and a dropping funnel. The flask was further dried under reduced pressure with a heat gun and flushed with argon, after which 4‐MeTHP (2 mL) and DIBALH (1.0 m solution in *n*‐hexane, 750 μL, 750 μmol) were added. The suspension was stirred for 30 min at room temperature and warmed to 90 °C. A solution of 1‐chloro‐4‐methylbenzene (**5 f**) (1.27 g, 10.0 mmol) in 4‐MeTHP (3 mL+3 mL for rinse) was added through a dropping funnel over 20 min and the mixture was stirred at 90 °C for 20 h in total. The reaction mixture was cooled to 0 °C followed by the addition of benzaldehyde (535 mg, 5.04 mmol) in 4‐MeTHP (10 mL). The resultant mixture was stirred for 1 h at room temperature and quenched at 0 °C by the addition of saturated aqueous NH_4_Cl and saturated aqueous Rochelle salt. The resulting mixture was extracted with Et_2_O (3×10 mL), and the combined organic layer was washed with brine, dried over anhydrous MgSO_4_, filtered and concentrated. The residue was purified by flash chromatography on silica gel (*n*‐hexane/EtOAc, 70:1→10:1 v/v) to give alcohol **6 f** (616 mg, 3.11 mmol, 31 %). Colorless needles; ^1^H NMR (300 MHz, CDCl_3_): *δ=*7.41–7.23 (m, 7 H), 7.15 (br d, *J=*7.8 Hz, 2 H), 5.82 (d, *J=*3.6 Hz, 1 H), 2.33 (s, 3 H), 2.18 ppm (d, *J=*3.6 Hz, 1 H, OH); ^13^C NMR (75 MHz, CDCl_3_): *δ=*144.2, 141.2, 137.4, 129.3, 128.6, 127.6, 126.7, 126.6, 76.3, 21.2 ppm.

**Phenyl(*m‐*tolyl)methanol (6 g).^[18]^** Well‐ground magnesium turnings (366 mg, 15.1 mmol) were placed in a well‐dried 100 mL three‐necked round bottom flask equipped with a reflux condenser and a dropping funnel. The flask was further dried under reduced pressure with a heat gun and flushed with argon, after which 4‐MeTHP (2 mL) and DIBALH (1.0 m solution in *n*‐hexane, 750 μL, 750 μmol) were added. The suspension was stirred for 30 min at room temperature and warmed to 90 °C. A solution of 1‐chloro‐3‐methylbenzene (**5 g**) (1.27 g, 10.0 mmol) in 4‐MeTHP (3 mL+3 mL for rinse) was added through a dropping funnel over 50 min and the mixture was stirred at 90 °C for 22 h in total. The reaction mixture was cooled to 0 °C followed by the addition of benzaldehyde (534 mg, 5.03 mmol) in 4‐MeTHP (10 mL). The resultant mixture was stirred for 1 h at room temperature and quenched at 0 °C by the addition of saturated aqueous NH_4_Cl and saturated aqueous Rochelle salt. The resulting mixture was extracted with Et_2_O (3×10 mL), and the combined organic layer was washed with brine, dried over anhydrous MgSO_4_, filtered and concentrated. The residue was purified by flash chromatography on silica gel (*n*‐hexane/EtOAc, 300:1→100:1→50:1 v/v) to give alcohol **6 g** (392 mg, 1.98 mmol, 20 %). Colorless solid; ^1^H NMR (300 MHz, CDCl_3_): *δ=*7.41–7.16 (m, 8 H), 7.08 (d, *J=*7.3 Hz, 1 H), 5.82 (d, *J=*3.4 Hz, 1 H), 2.34 (s, 3 H), 2.21 ppm (br s, 1 H, OH); ^13^C NMR (75 MHz, CDCl_3_): *δ=*144.0, 143.9, 138.3, 128.6, 128.54, 128.49, 127.6, 127.3, 126.7, 123.8, 76.4, 21.6 ppm.

**Phenyl(*o‐*tolyl)methanol (6 h).^[18]^** Well‐ground magnesium turnings (366 mg, 15.1 mmol) were placed in a well‐dried 50 mL three‐necked round bottom flask equipped with a reflux condenser and a dropping funnel. The flask was further dried under reduced pressure with a heat gun and flushed with argon, after which 4‐MeTHP (2 mL) and DIBALH (1.0 m solution in *n*‐hexane, 750 μL, 750 μmol) were added. The suspension was stirred for 30 min at room temperature and warmed to 90 °C. A solution of 1‐chloro‐2‐methylbenzene (**5 h**) (1.27 g, 10.0 mmol) in 4‐MeTHP (3 mL+3 mL for rinse) was added through a dropping funnel over 20 min and the mixture was stirred at 90 °C for 21 h in total. The reaction mixture was cooled to 0 °C followed by the addition of benzaldehyde (532 mg, 5.00 mmol) in 4‐MeTHP (10 mL). The resulting mixture was stirred for 1 h at room temperature and quenched at 0 °C by the addition of saturated aqueous NH_4_Cl and saturated aqueous Rochelle salt. The resulting mixture was extracted with Et_2_O (3×10 mL), and the combined organic layer was washed with brine, dried over anhydrous MgSO_4_, filtered and concentrated. The residue was purified by flash chromatography on silica gel (*n*‐hexane/EtOAc, 50:1→10:1 v/v) to give alcohol **6 h** (108 mg, 0.545 mmol, 6 %). Colorless solid; ^1^H NMR (300 MHz, CDCl_3_): *δ=*7.46 (dd, 1 H, *J=*7.3, 1.8 Hz), 7.28–7.07 (m, 8 H), 5.94 (d, *J=*3.7 Hz, 1 H), 2.19 (s, 3 H), 2.11 ppm (d, *J=*3.7 Hz, 1 H, OH); ^13^C NMR (100 MHz, CDCl_3_): *δ=*142.9, 141.5, 135.5, 130.7, 128.6, 127.71, 127.65, 127.2, 126.34, 126.25, 73.5, 19.5 ppm.

***n*** **Butylmagnesium chloride (5 g′)**. Well‐ground magnesium turnings (2.19 g, 90.1 mmol) were placed in a well‐dried 300 mL three‐necked round bottom flask equipped with a reflux condenser and a dropping funnel. The flask was further dried under reduced pressure with a heat gun and flushed with argon, after which 4‐MeTHP (12 mL) and DIBALH (1.0 m solution in *n*‐hexane, 450 μL, 450 μmol) were added. The suspension was stirred for 30 min at room temperature and warmed to 60 °C. A solution of 1‐chlorobutane (**5 g**) (6.3 mL, 60 mmol) in 4‐MeTHP (36 mL) was added through a dropping funnel over 2 h and the mixture was additionally stirred for 2 h at 60 °C. The solution was left at room temperature until two phases were clearly separated. After addition of 4‐MeTHP (20 mL), the supernatant was transferred into a sealed brown glass bottle and stored at room temperature in the dark. Titration of the Grignard reagent^18^ determined the concentration of **5 g“** to be 0.66 m (74 % yield).

### Coupling Reactions in 4‐MeTHP

**2‐((Trimethylsilyl)ethynyl)cyclohex‐2‐en‐1‐one (8).^[68]^** Iodoenone **7** (188 mg, 0.845 mmol) and CuI (16.2 mg, 0.0851 mmol) were placed in a well‐dried 200 mL one‐necked round bottom flask, which was flushed with argon. 4‐MeTHP (8.4 mL), *i*Pr_2_NEt (653 μL, 2.52 mmol), ethynyltrimethylsilane (174 μL, 1.26 mmol), and (Ph_3_P)_2_PdCl_2_ were sequentially added, and the mixture was stirred at room temperature for 2 h. The reaction mixture was diluted with *n*‐hexane and quenched by the addition of saturated aqueous NH_4_Cl. The resulting mixture was extracted with *n*‐hexane (3×15 mL), and the combined organic layer was washed with 10 % aqueous ammonium, brine, dried over anhydrous MgSO_4_, filtered and concentrated. The residue was purified by flash chromatography on silica gel (*n*‐hexane/EtOAc, 50:1→10:1→5:1 v/v) to give enyne **8** (120 mg, 0.624 mmol, 74 %). Yellow solid; ^1^H NMR (400 MHz, CDCl_3_): *δ=*7.33 (t, *J=*4.4 Hz, 1 H), 2.49–2.42 (m, 4 H), 2.01 (m, 2 H), 0.21 ppm (s, 9 H); ^13^C NMR (100 MHz, CDCl_3_): *δ=*195.5, 155.5, 125.4, 99.3, 97.5, 38.1, 26.5, 22.4, 0.00 ppm.

**(*E*)‐2‐(3‐Hydroxyoct‐1‐en‐1‐yl)cyclohex‐2‐en‐1‐one (9)**. Iodoenone **7** (251 mg, 0.601 mmol), stannane **2 b** (111 mg, 0.500 mmol) and (Ph_3_P)_2_PdCl_2_ (34.9 mg, 0.0497 mmol) were placed in a well‐dried 30 mL two‐necked round bottom flask, which was flushed with argon. 4‐MeTHP (5 mL) was added, and the mixture was stirred at 60 °C for 4 h. The reaction mixture was quenched by the addition of saturated aqueous NH_4_Cl and extracted with EtOAc (2×5 mL). The combined organic layer was washed with brine, dried over anhydrous MgSO_4_, filtered and concentrated. The residue was purified by flash chromatography on silica gel (*n*‐hexane/EtOAc, 30:1→10:1→5:1→1:1 v/v) to give dienone **9** (72.0 mg, 0.356 mmol, 71 %). Yellow oil; ^1^H NMR (400 MHz, CDCl_3_): *δ=*7.01 (t, *J=*4.6 Hz, 1 H), 6.38 (dt, *J=*16, 1.0 Hz, 1 H), 6.18 (dd, *J=*16, 6.8 Hz, 1 H), 4.17 (q, *J=*6.8 Hz, 1 H), 2.49–2.43 (m, 4 H), 2.00 (m, 2 H), 1.62–1.48 (m, 2 H), 1.42–1.24 (m, 6 H), 0.88 ppm (t, *J=*6.4 Hz, 3 H); ^13^C NMR (100 MHz, CDCl_3_): *δ=*198.7, 145.7, 135.8, 134.6, 124.5, 73.3, 38.9, 37.3, 31.9, 26.5, 25.2, 22.724, 22.715, 14.2 ppm; IR (film on ZnSe): $\tilde{\nu }$ =3420, 2955, 2930, 2860, 1682, 1456, 1379, 1136 cm^−1^; HRMS (APCI+): *m*/*z*: calcd for C_14_H_21_O: 205.1587 [*M*‐OH]^+^; found: 205.1589.

**4,5‐Dihydro‐[1,1′‐biphenyl]‐2(3*H*)‐one (10).^[45]^** Iodoenone **7** (111 mg, 0.501 mmol), Na_2_CO_3_ (106 mg, 1.00 mmol), PhB(OH)_2_ (122 mg, 1.00 mmol) and 5 wt % Pd on carbon (25.3 mg) were placed in a well‐dried 30 mL two‐necked round bottom flask, which was flushed with argon. 4‐MeTHP (1.5 mL) and water (1.5 mL) were added, and the mixture was stirred at 80 °C for 5 h. The reaction mixture was filtered through a pad of Celite and the filtrate was extracted, after the addition of water (10 mL), with EtOAc (3×5 mL). The combined organic layer was washed with brine, dried over anhydrous MgSO_4_, filtered and concentrated. The residue was purified by flash chromatography on silica gel (*n*‐hexane/EtOAc, 30:1 v/v) to give enone **10** (82.8 mg, 0.481 mmol, 96 %) as a colorless solid. ^1^H NMR (400 MHz, CDCl_3_): *δ=*7.37–7.27 (m, 5 H), 7.04 (t, *J=*4.2 Hz, 1 H), 2.62–2.58 (m, 2 H), 2.55 (t, *J=*6.0 Hz, 1 H), 2.54 (t, *J=*6.0 Hz, 1 H), 2.15–2.08 ppm (m, 2 H); ^13^C NMR (100 MHz, CDCl_3_): *δ=*198.1, 148.2, 140.5, 136.7, 128.7, 128.1, 127.7, 39.2, 26.7, 23.1 ppm.

**3‐Butyl‐2‐iodocyclohexan‐1‐one (11)**. CuBr⋅SMe_2_ (49.1 mg, 0.239 mmol) was placed in a well‐dried 20 mL two‐necked round bottom flask equipped with a dropping funnel, which was flushed with argon. 4‐MeTHP (1 mL) was added, and the solution was cooled to −78 °C. *n*Butylmagnesium bromide (0.66 m in 4‐MeTHP, 1.8 mL, 1.2 mmol) was added, and the suspension was stirred at −78 °C for 1 h. A solution of iodoenone **7** (178 mg, 0.799 mmol) in 4‐MeTHP (7 mL) was added through a dropping funnel over 15 min and the mixture was stirred at −78 °C for 2.5 h. The reaction mixture was then warmed to 0 °C and, after 5 min, quenched by the addition of saturated aqueous NH_4_Cl. The resulting mixture was extracted with *n*‐hexane (2×10 mL). The combined organic layer was washed with water, brine, dried over anhydrous MgSO_4_, filtered and concentrated to give iodoketone **11** (204 mg, 0.728 mmol, 91 %) as an inseparable 1.6:1 diastereomeric mixture. Yellow oil; ^1^H NMR (400 MHz, CDCl_3_): *δ=*4.51 (dt, *J=*4.8, 2.4 Hz, 0.38 H), 4.45 (dt, *J=*6.4, 1.6 Hz, 0.62 H); 3.37 (m, 0.38 H), 3.18 (ddd, *J=*15, 10, 6.8 Hz, 0.62 H), 2.34–1.16 (m, 12 H), 0.90 (t, *J=*8.4 Hz, 1.15 H), 0.89 ppm (t, *J=*7.4 Hz, 1.85 H);^13^C NMR (100 MHz, CDCl_3_): *δ=*205.7, 205.3, 46.0, 43.6, 42.2, 38.3, 36.6, 36.5, 34.9, 33.8, 29.3, 28.3, 27.8, 26.1, 25.3, 22.69, 22.66, 22.5, 14.12, 14.05 ppm; IR (film on ZnSe): $\tilde{\nu }$ =2955, 2930, 2859, 1715, 1456, 1317, 1250, 1219, 1146, 1123 cm^−1^; HRMS (ESI+): *m*/*z*: calcd for C_10_H_18_OI: 281.0397 [*M*+H]^+^; found: 281.0409.

**Dimethyl (*E*)‐2‐(2‐methyl‐4‐phenylbut‐2‐en−1‐yl)malonate (13 a)**. Bromobenzene (**12**) (79.6 mg, 0.507 mmol), methyl malonate (334 mg, 2.53 mmol), (*o‐*tol)_3_P (14.9 mg, 0.0489 mmol), NaHCO_3_ (126 mg, 1.50 mmol), molecular sieves 4A (202 mg, activated by a heat gun under reduced pressure), isoprene (103 mg, 1.52 mmol), and *n*Bu_4_NCl (150 mg, 0.541 mmol) were placed in a well‐dried Pyrex tube with a screw cap. The mixture was dissolved in 4‐MeTHP (1 mL), which was followed by the addition of Pd(OAc)_2_ (6.4 mg, 0.029 mmol). The resulting mixture was stirred at 80 °C for 24 h and filtered through a cotton plug using EtOAc as washings. Water (10 mL) was added to the filtrate, which was extracted with EtOAc (2×5 mL). The combined organic layer was washed with brine, dried over anhydrous MgSO_4_, filtered and concentrated. The residue was purified by flash chromatography on silica gel (*n*‐hexane/EtOAc=50:1→20:1 v/v) to give the coupling product **13 a** (97.7 mg, containing 9 % impurity). The calculated yield of **13 a** based on the NMR analysis was 64 % (89.1 mg, 0.322 mmol). The analytical sample was obtained by normal‐phase HPLC equipped with a prepacked column [Mightysil, Si 60 250‐20 (5 μm)] using *n*‐hexane/EtOAc (86:14 v/v) as an eluent. Colorless oil; ^1^H NMR (400 MHz, CDCl_3_): *δ=*7.28–7.24 (m, 2 H), 7.18–7.11 (m, 3 H), 5.41 (m, 1 H), 3.65 (s, 6 H), 3.61 (t, *J=*7.8 Hz, 1 H), 3.32 (d, *J=*7.3 Hz, 2 H), 2.64 (d, *J=*7.8 Hz), 1.73 ppm (s, 3 H); ^13^C NMR (100 MHz, CDCl_3_): *δ=*169.6, 141.1, 131.9, 128.5, 128.4, 126.4, 126.0, 52.6, 50.6, 38.8, 34.3, 16.0 ppm; IR (film on ZnSe): $\tilde{\nu }$ =3061, 3026, 2953, 2847, 1748, 1732, 1603, 1495, 1454, 1435, 1339, 1281, 1238 cm^−1^; HRMS (APCI+): *m*/*z*: calcd for C_16_H_21_O_4_: 277.1434 [*M*+H]^+^; found: 277.1431.

### Miscellaneous reactions in 4‐MeTHP

**(3,5‐Dimethoxyphenyl)methanol (15)**. 3,5‐Dimethoxybenzoic acid (**14**) (4.48 g, 24.6 mmol) was placed in a well‐dried 300 mL two‐necked round bottom flask, which was flushed with argon. 4‐MeTHP (62 mL) was added and the suspension was cooled to 0 °C. LiAlH_4_ (1.03 g, 27.2 mmol) was added in portions and the mixture was stirred at room temperature for 1 h. The reaction mixture was quenched by the addition of saturated aqueous Rochelle salt (20 mL) and water (20 mL), and the precipitate was filtered through a pad of Celite. The filtrate was extracted with EtOAc (3×30 mL). The combined organic layer was washed with brine, dried over anhydrous MgSO_4_, filtered and concentrated to give alcohol **15** (4.14 g, 24.6 mmol, 100 %). Colorless solid; ^1^H NMR (400 MHz, CDCl_3_): *δ=*6.53 (br d, *J=*2.4 Hz, 2 H), 6.39 (t, *J=*2.4 Hz, 1 H), 4.65 (s, 2 H), 3.80 ppm (s, 3 H); ^13^C NMR (75 MHz, CDCl_3_): *δ=*161.2, 143.5, 104.8, 99.9, 65.5, 55.5 ppm. This compound is commercially available. (CAS: 705‐76‐0)

**Ethyl cinnamate (17)**. Ethyl (triphenylphosphoranylidene)acetate (419 mg, 1.20 mmol) was added to a solution of benzaldehyde (**16**) (106 mg, 1.00 mmol) in 4‐MeTHP (2 mL). The mixture was stirred at room temperature for 40 min. Additional ethyl (triphenylphosphoranylidene)acetate (104 mg, 0.297 mmol) was added and the mixture was stirred for 1 h. The solution was directly purified by flash chromatography on silica gel (*n*‐hexane/EtOAc, 40:1 v/v) to give ester **17** (180 mg, quant, *E*/*Z=*10:1). Colorless oil; The following assignments were made with a mixture of stereoisomers. Data for *E*‐isomer: ^1^H NMR (300 MHz, CDCl_3_): *δ=*7.69 (d, *J=*16 Hz, 1 H), 7.54–7.52 (m, 2 H), 7.40–7.33 (m, 3 H), 6.44 (d, *J=*16 Hz, 1 H), 4.27 (q, *J=*7.2 Hz, 2 H), 1.34 ppm (t, *J=*7.2 Hz, 3 H); ^13^C NMR (75 MHz, CDCl_3_): *δ=*167.0, 144.6, 134.6, 130.3, 129.0, 128.1, 118.4, 60.6, 14.4 ppm. Data for *Z*‐isomer: ^1^H NMR (300 MHz, CDCl_3_): *δ=*7.60–7.54 (m, 2 H), 7.40–7.33 (m, 3 H), 6.94 (d, *J=*13 Hz, 1 H), 5.95 (d, *J=*16 Hz, 1 H), 4.17 (q, *J=*7.2 Hz, 2 H), 1.24 ppm (t, *J=*7.2 Hz, 3 H). This compound is commercially available. (CAS: 4192‐77‐2)

**2‐Iodocyclohex‐2‐en‐1‐one (7).^[69]^** A solution of iodine (6.35 g, 25.0 mmol) in 4‐MeTHP (26 mL) was added to a solution of cyclohex‐2‐en‐1‐one (**18**) (970 μL, 10.1 mmol) and pyridine (2.8 mL, 35 mmol) in 4‐MeTHP (14 mL) through a dropping funnel over 1 h. After 2 h, the solution was diluted with *n*‐hexane (40 mL) and quenched by the addition of 1 m aqueous NaHCO_3_ (20 mL). The resulting mixture was extracted with *n*‐hexane (3×15 mL). The combined organic layer was washed with 1 m aqueous HCl, water, brine, dried over anhydrous MgSO_4_, filtered and concentrated. The residue was purified by open chromatography on silica gel (*n*‐hexane/EtOAc, 30:1 v/v) to give iodoenone **7** (2.25 g, 10.1 mmol, 100 %). Colorless solid; ^1^H NMR (400 MHz, CDCl_3_): *δ=*7.77 (t, *J=*4.4 Hz, 1 H), 2.67 (m, 2 H), 2.44 (td, *J=*6.0, 4.4 Hz, 2 H), 2.12–2.06 ppm (m, 2 H); ^13^C NMR (100 MHz, CDCl_3_): *δ=*192.3, 159.8, 103.9, 37.4, 30.1, 22.9 ppm.

**1‐(4‐(2‐(Dimethylamino)ethoxy)phenyl)‐1,2‐diphenylbutan‐1‐ol (20)**. *n*BuLi (1.6 M solution in hexane, 1.15 mL, 1.84 mmol) was added to a solution of aryl bromide **19** 18 (416 mg, 1.70 mmol) in 4‐MeTHP (13 mL) at −78 °C. After 1 h, a solution of 1,2‐diphenylbutan‐1‐one^18^ (456 mg, 1.99 mmol) in 4‐MeTHP (2 mL and 2 mL for rinse) was added and the mixture was gradually warmed to 0 °C. After 1.5 h, the reaction mixture was quenched by the addition of water (10 mL) and extracted with EtOAc (2×10 mL). The combined organic layer was washed with brine, dried over anhydrous MgSO_4_, filtered and concentrated. The residue was purified by flash chromatography on silica gel (DCM→DCM/MeOH, 10:1 v/v) followed by recrystallization from EtOH to give alcohol **20** (478 mg, 1.23 mmol, 72 %). Colorless solid; M.p. 126–128 °C; ^1^H NMR (400 MHz, CDCl_3_): *δ=*7.48 (d, *J=*9.0 Hz, 2 H), 7.20–6.99 (m, 10 H), 6.89 (d, *J=*9.0 Hz, 2 H), 4.34 (br s, 2 H), 3.56 (t, *J=*7.2 Hz, 1 H), 3.16 (br s, 2 H), 2.69 (br s, 6 H), 2.38 (s, 1 H, OH), 1.80 (quint, *J=*7.2 Hz, 2 H), 0.75 ppm (t, *J=*7.2 Hz, 3 H); ^13^C NMR (100 MHz, CDCl_3_): *δ=*157.5, 146.8, 140.0, 138.5, 130.3, 127.7, 127.6, 126.4, 126.1, 125.8, 114.1, 80.8, 65.9, 58.4, 56.5, 45.9, 23.5, 12.7 ppm; IR (film on ZnSe): $\tilde{\nu }$ =3154, 3061, 3024, 2955, 2870, 2830, 2781, 1607, 1506, 1447, 1287, 1246, 1172, 1053, 1032 cm^−1^; HRMS (APCI+): *m*/*z*: calcd for C_26_H_32_O_2_N: 390.2428 [*M*+H]^+^; found: 390.2424.

**Geranyl bromide (22).^[70]^** MsCl (402 μL, 5.20 mmol) was added to a solution of geraniol (**21**) (620 mg, 4.02 mmol) and Et_3_N (836 μL, 6.00 mmol) in 4‐MeTHP (10 mL) at −40 °C. After 15 min at −40 °C, a solution of LiBr (1.39 g, 16.0 mmol) in 4‐MeTHP (10 mL) was added, and the reaction mixture was warmed to 0 °C. After 30 min, the reaction mixture was diluted with *n*‐hexane (10 mL) and quenched by the addition of water (10 mL). The resulting mixture was extracted with *n*‐hexane (2×8 mL). The combined organic layer was washed with brine, dried over anhydrous MgSO_4_, filtered and carefully concentrated to give a 6.5:1 mixture of geranyl bromide (**22**) and 4‐MeTHP (946 mg, quant). The analytical sample was obtained by further concentration of the mixture. Yellow oil; ^1^H NMR (300 MHz, CDCl_3_): *δ=*5.53 (m, 1 H), 5.07 (m, 1 H), 4.03 (d, 2 H, *J=*8.4 Hz), 2.15–2.03 (m, 4 H), 1.73 (d, *J=*1.2 Hz, 3 H), 1.68 (s, 3 H), 1.60 ppm (s, 3 H); ^13^C NMR (75 MHz, CDCl_3_): *δ=*143.7, 132.1, 123.7, 120.8, 39.7, 29.7, 26.4, 25.8, 17.8, 16.1 ppm. This compound is commercially available. (CAS: 6138‐90‐5)

**Arylation of 23.^[50]^** Penta‐*O*‐acetyl‐β‐d‐glucopyranose (**23**) (807 mg, 2.07 mmol) and 4‐methoxyphenol (387 mg, 3.12 mmol) were placed in a well‐dried 30 mL two‐necked round bottom flask, which was flushed with argon. 4‐MeTHP (6.5 mL) was added, and the mixture was cooled to 0 °C. TMSOTf (40 μL, 0.22 mmol) was added, and the reaction mixture was stirred at room temperature for 4 h. Additional 4‐MeTHP (3.5 mL) was added and the mixture was warmed to 35 °C. After 1 h, TMSOTf (40 μL, 0.22 mmol) was further added. After 1 h at 35 °C, the reaction mixture was diluted with EtOAc (8 mL) and quenched by the addition of saturated aqueous NaHCO_3_ (5 mL). The organic layer was separated, washed with water, brine, dried over anhydrous MgSO_4_, filtered and concentrated. The residue was purified by flash chromatography on silica gel (*n*‐hexane/EtOAc, 3:1→2:1→1:1 v/v) to give a 2.3:1 inseparable mixture of **23** and **24** (729 mg) as a colorless solid. The yield of **24** based on the NMR analysis was 25 % (235 mg, 0.518 mmol). The authentic sample of **24** 71 was obtained by using DCM as a solvent. Colorless solid; ^1^H NMR (400 MHz, CDCl_3_): *δ=*6.95 (d, *J=*9.2 Hz, 2 H), 6.82 (d, *J=*9.2 Hz, 2 H), 5.30–5.10 (m, 3 H), 4.95 (d, *J=*7.6 Hz, 1 H), 4.29 (dd, *J=*12, 5.2 Hz, 1 H), 4.17 (dd, *J=*12, 2.6 Hz, 1 H), 4.11 (t, *J=*7.2 Hz, 1 H), 3.79 (m, 1 H), 3.78 (s, 3 H), 2.084 (s, 3 H), 2.076 (s, 3 H), 2.04 (s, 3 H), 2.03 ppm (s, 3 H); ^13^C NMR (75 MHz, CDCl_3_): *δ=*170.6, 170.3, 169.5, 169.3, 156.0, 151.1, 118.9, 114.7, 100.5, 72.9, 72.1, 71.4, 68.5, 62.1, 55.8, 20.77, 20.73, 20.69, 20.66 ppm.

**Attempted allylation of 23.^[51]^** Allyltrimethylsilane (0.40 mL, 2.5 mmol) and BF_3_⋅Et_2_O (0.30 mL, 2.4 mmol) were added to a solution of **23** (192 mg, 0.492 mmol) in 4‐MeTHP (3.5 mL) at 0 °C. The mixture was stirred at room temperature for 15 min and at 80 °C for 17 h. TLC indicated that most of the starting material remained and deacetylation slightly occurred.

**2‐Formyl‐3,5‐dimethoxybenzyl palmitate (26)**. POCl_3_ (4.20 mL, 46.3 mmol) was added to DMF (2.95 mL, 38.3 mmol) at 0 °C. The mixture was warmed to room temperature and stirred for 40 min. Alcohol **15** (9.12 g, 22.4 mmol) was added and the resulting mixture was stirred at 80 °C for 4 h. The reaction mixture was diluted with DCM and washed with saturated aqueous NaHCO_3_. The aqueous layer was extracted with DCM (3×50 mL), and the combined organic layer was washed with brine, dried over anhydrous MgSO_4_, filtered and concentrated to give aldehyde **26** (9.61 g, 22.1 mmol, 98 %). The analytical sample was obtained by flash chromatography on silica gel (*n‐*hexane/EtOAc, 10:1 v/v). Flesh‐colored solid; ^1^H NMR (300 MHz, CDCl_3_): *δ=*0.44 (d, *J=*0.6 Hz, 1 H), 6.60 (dd, *J=*2.4, 0.6 Hz, 1 H), 6.40 (d, *J=*2.4 Hz), 5.51 (s, 2 H), 3.89 (s, 3 H), 3.87 (s, 3 H), 2.41 (t, *J=*7.5 Hz, 2 H), 1.68 (qui, *J=*7.5 Hz, 2 H), 1.31–1.18 (m, 24 H), 0.87 ppm (t, *J=*6.7 Hz, 3 H); ^13^C NMR (75 MHz, CDCl_3_): *δ=*189.9, 173.3, 165.4, 165.3, 142.7, 115.9, 104.5, 96.6, 64.5, 56.0, 55.6, 34.5, 32.0, 29.82, 29.80, 29.78, 29.73, 29.6, 29.5, 29.4, 29.3, 25.2, 22.8, 14.2 ppm; IR (film on ZnSe): $\tilde{\nu }$ =2999, 2924, 2853, 1738, 1599, 1464, 1429, 1350, 1321, 1298 cm^−1^; HRMS (ESI+): *m*/*z*: calcd for C_26_H_43_O_5_: 435.3105 [*M*+H]^+^; found 435.3089.

**2‐Formyl‐3‐hydroxy‐5‐methoxybenzyl palmitate (27)**. BBr_3_ (1.0 M solution in CH_2_Cl_2_, 2.6 mL, 2.6 mmol) was added to a solution of aldehyde **26** (1.00 g, 2.30 mmol) in 4‐MeTHP (40 mL) at 0 °C. After 1.5 h, the reaction mixture was warmed to room temperature and stirred for additional 1 h. The reaction mixture was quenched by the addition of saturated aqueous NaHCO_3_ (30 mL) and H_2_O (20 mL), and extracted with EtOAc (2×20 mL). The precipitates in the aqueous layer was removed by filtration though a pad of Celite and the filtrate was extracted with EtOAc (2×20 mL). The combined organic layer was dried over anhydrous MgSO_4_, filtered and concentrated. The residual solid was recrystallized from *n*‐hexane/EtOAc (10:1 v/v) to give phenol **27** (573 mg, 1.37 mmol, 59 %) as a brownish solid. The mother liquid was further purified by flash chromatography on silica gel (*n‐*hexane → *n‐*hexane/EtOAc, 20:1 v/v) to give **27** (216 mg, 0.517 mmol, 22 %) as a colorless solid. ^1^H NMR (400 MHz, CDCl_3_): *δ=*12.46 (s, 1 H), 10.06 (s, 1 H), 6.51 (d, *J=*2.4 Hz, 1 H), 6.40 (d, *J=*2.4 Hz, 1 H), 5.30 (s, 2 H), 3.85 (s, 3 H), 2.34 (t, *J=*7.4 Hz, 2 H), 1.62 (m, 2 H), 1.31–1.21 (m, 24 H), 0.88 ppm (t, *J=*6.8 Hz, 3 H); ^13^C NMR (75 MHz, CDCl_3_): *δ=*192.7, 173.2, 166.9, 166.6, 140.8, 112.5, 110.8, 100.9, 62.7, 55.9, 34.4, 32.1, 29.84, 29.81, 29.78, 29.72, 29.6, 29.5, 29.4, 29.3, 25.0, 22.8, 14.2 ppm; IR (film on ZnSe): $\tilde{\nu }$ =2918, 2851, 1732, 1622, 1327, 1281, 1206, 1155 cm^−1^; HRMS (APCI+): *m*/*z*: calcd for C_25_H_41_O_5_: 421.2949 [*M*+H]^+^; found: 421.2943.

**5‐Bromo‐3‐methylpentan‐1‐ol (28)**. BBr_3_ (1.0 m solution in DCM, 271 μL, 0.271 mmol) was added to 4‐MeTHP (2.4 mL) at 0 °C. The mixture was warmed to room temperature and stirred for 24 h. The reaction mixture was quenched by the addition of saturated aqueous NaHCO_3_ (2 mL) and extracted with EtOAc (2×5 mL). The combined organic layer was washed with brine, dried over anhydrous MgSO_4_, filtered and concentrated to give **28** (147 mg, 0.812 mmol). Pale yellow oil; ^1^H NMR (400 MHz, CDCl_3_): *δ=*3.76–3.65 (m, 2 H), 3.51–3.39 (m, 2 H), 1.95–1.78 (m, 2 H), 1.76–1.58 (m, 2 H), 1.47–1.39 (m, 1 H), 0.94 ppm (d, *J=*6.4 Hz, 3 H); ^13^C NMR (75 MHz, CDCl_3_): *δ=*60.8, 40.1, 39.2, 31.9, 28.6, 19.0 ppm; IR (film on ZnSe): $\tilde{\nu }$ =3335, 2961, 2930, 2874, 1462, 1381, 1256, 1221 cm^−1^; HRMS (APCI+): *m*/*z*: calcd for C_6_H_12_Br: 163.0117 [*M*+H]^+^; found: 163.0118.

**3‐Chloro‐1‐(3,4‐dimethoxyphenyl)propan‐1‐one (30)**.^72^ 3‐Chloropropanoyl chloride (303 μL, 3.15 mmol) and 1,2‐dimethoxybenzene (**29**) (384 μL, 3.00 mmol) were added to a suspension of AlCl_3_ (479 mg, 3.60 mmol) in 4‐MeTHP (6 mL) at room temperature. After 17 h, the reaction mixture was quenched by the addition of water and extracted with DCM (2×5 mL). The combined organic layer was washed with brine, dried over anhydrous MgSO_4_, filtered and concentrated. The crude NMR indicated that a ratio of **29** and **30** was 51:1 (2 % yield). The analytical sample was obtained from the reaction in DCM. Colorless solid; ^1^H NMR (300 MHz, CDCl_3_): *δ=*7.58 (dd, *J=*8.4, 2.1 Hz, 1 H), 7.53 (d, *J=*2.1 Hz, 1 H), 6.90 (d, *J=*8.4 Hz, 1 H), 3.96 (s, 3 H), 3.94 (s, 3 H), 3.93 (t, *J=*6.9 Hz, 2 H), 3.43 ppm (t, *J=*6.9 Hz, 2 H); ^13^C NMR (75 MHz, CDCl_3_): *δ=*195.3, 153.9, 149.4, 129.9, 122.9, 110.3 (2), 56.20, 56.16, 40.9, 39.1 ppm.

**Synthesis of 4‐nitrobenzaldehyde (32 a) by TCCA oxidation**. TCCA (94.9 mg, 0.408 mmol) was added to a solution of 4‐nitrobenzyl alcohol (**31 a**) (154 mg, 1.01 mmol) and TEMPO (6.5 mg, 0.042 mmol) in 4‐MeTHP (5 mL). After 3 h at room temperature, the reaction mixture was quenched by the addition of 1 M aqueous NaOH and extracted with 4‐MeTHP (2×5 mL). The combined organic layer was washed with brine, dried over anhydrous MgSO_4_, filtered and concentrated. The residue was purified by flash chromatography on silica gel (*n*‐hexane/EtOAc, 20:1 v/v) to give aldehyde **32 a** (143 mg, 0.946 mmol, 94 %). Colorless solid; ^1^H NMR (400 MHz, CDCl_3_): *δ=*10.16 (s, 1 H), 8.40 (d, *J=*8.8 Hz, 2 H), 8.08 ppm (d, *J=*8.8 Hz, 2 H); ^13^C NMR (75 MHz, CDCl_3_): *δ=*190.3, 151.3, 140.2, 130.6, 124.4 ppm. This compound is commercially available. (CAS: 555‐16‐8)

**Synthesis of 4‐methoxybenzaldehyde (32 b) by TCCA oxidation**. TCCA (95.3 mg, 0.410 mmol) was added to a solution of 4‐methoxybenzyl alcohol (**31 b**) (139 mg, 1.01 mmol) and TEMPO (7.2 mg, 0.046 mmol) in 4‐MeTHP (3 mL). After 40 min at room temperature, the reaction mixture was quenched by the addition of 1 m aqueous NaOH and extracted with 4‐MeTHP (2×5 mL). The combined organic layer was washed with brine, dried over anhydrous MgSO_4_, filtered and concentrated. The residue was purified by flash chromatography on silica gel (*n*‐hexane/EtOAc, 20:1 v/v) to give aldehyde **32 b** (102 mg, 0.751 mmol, 74 %). Colorless oil; ^1^H NMR (400 MHz, CDCl_3_): *δ=*9.89 (s, 1 H), 7.84 (d, *J=*8.4 Hz, 2 H), 7.01 (d, *J=*8.4 Hz, 2 H), 3.89 ppm (s, 3 H); ^13^C NMR (100 MHz, CDCl_3_): *δ=*190.8, 164.6, 132.0, 129.9, 114.3, 55.6 ppm. This compound is commercially available. (CAS: 123‐11‐5)

**Synthesis of 32 a by Dess–Martin oxidation**. Dess–Martin periodinane (97 % purity, 262 mg, 0.599 mmol) was added to a solution of **31 a** (76.5 mg, 0.500 mmol) and NaHCO_3_ (126 mg, 1.50 mmol) in 4‐MeTHP (2.5 mL) at 0 °C. The mixture was warmed to room temperature and stirred for 4.5 h. The reaction mixture was quenched successively by the addition of saturated aqueous Na_2_S_2_O_3_ (2 mL) and saturated aqueous NaHCO_3_ (2 mL) and extracted with EtOAc (10 mL). The organic layer was washed with brine, dried over anhydrous MgSO_4_, filtered and concentrated. The residue was purified by flash chromatography on silica gel (*n*‐hexane/EtOAc, 40:1→20:1 v/v) to give aldehyde **32 a** (69.0 mg, 0.457 mmol, 91 %) as a colorless solid.

**Synthesis of 32 b by Dess–Martin oxidation**. Dess–Martin periodinane (97 % purity, 262 mg, 0.599 mmol) was added to a solution of **31 b** (97 % purity, 71.2 mg, 0.500 mmol) and NaHCO_3_ (126 mg, 1.50 mmol) in 4‐MeTHP (2.5 mL) at 0 °C. The mixture was warmed to room temperature and stirred for 3 h. The reaction mixture was quenched successively by the addition of saturated aqueous Na_2_S_2_O_3_ (2 mL) and saturated aqueous NaHCO_3_ (2 mL) and extracted with EtOAc (2×5 mL). The organic layer was washed with brine, dried over anhydrous MgSO_4_, filtered and concentrated. The residue was purified by flash chromatography on silica gel (*n*‐hexane/EtOAc, 40:1→20:1 v/v) to give aldehyde **32 b** (61.3 mg, 0.450 mmol, 90 %) as a colorless oil.

**4‐((Tetrahydro‐2*H*‐pyran‐2‐yl)oxy)butan‐1‐ol (31 c)**. 1,4‐Butanediol (50.0 g, 554 mmol) and three drops of concentrated HCl were placed in a well‐dried 300 mL two‐necked round bottom flask equipped a dropping funnel. 3,4‐Dihydro‐2*H*‐pyran (29 mL, 0.32 mol) was slowly added through the dropping funnel over 2.5 h. After 30 min, the reaction mixture was cooled to 0 °C and additional 3,4‐dihydro‐2*H*‐pyran (10 mL, 0.11 mol) was added over 45 min. After 1.5 h at room temperature, the reaction mixture was diluted with *n*‐hexane/EtOAc (1:1 v/v) and quenched by the addition of saturated aqueous NaHCO_3_. The resulting mixture was extracted with *n*‐hexane/EtOAc (1:1 v/v) (3×30 mL). The combined organic layer was washed with brine, dried over anhydrous MgSO_4_, filtered and concentrated. The residual oil was distilled under reduced pressure (bp: 110–115 °C/20 Pa) to give alcohol **31 c** (52.0 g, 298 mmol, 67 %) as a colorless oil. ^1^H NMR (300 MHz, CDCl_3_): *δ=*4.56 (m, 1 H), 3.87–3.72 (m, 2 H), 3.66–3.61 (m, 2 H), 3.52–3.37 (m, 2 H), 2.77 (br s, 1 H, OH), 1.84–1.45 ppm (m, 10 H); ^13^C NMR (75 MHz, CDCl_3_): *δ=*99.0, 67.6, 62.7, 62.5, 30.7, 30.1, 26.6, 25.5, 19.6 ppm; IR (film on ZnSe): $\tilde{\nu }$ =3434, 2952, 2867, 1454, 1441, 1353, 1261 cm^−1^; MS (EI, 70 eV): *m*/*z* (%) 175 ([*M*+H]^+^, 5), 101 (12), 85 (100), 73 (21); HRMS (ESI+): *m*/*z*: calcd for C_9_H_19_O_3_: 175.1334 [*M*+H]^+^; found: 175.1326.

**4‐((Tetrahydro‐2*H*‐pyran‐2‐yl)oxy)butanal (32 c).^[73]^** A solution of DMSO (450 μL, 6.34 mmol) in 4‐MeTHP (5 mL) was added to a solution of oxalyl chloride (390 μL, 4.54 mmol) in 4‐MeTHP (6 mL) at −70 °C. After 30 min, a solution of alcohol **31 c** (261 mg, 1.50 mmol) in 4‐MeTHP (9 mL) was added at −70 °C. After 1 h, Et_3_N (2.1 mL, 15 mmol) was added and the reaction mixture was warmed to 0 °C. After 1 h, the reaction mixture was quenched by the addition of water (10 mL) and extracted with EtOAc (2×10 mL). The combined organic layer was washed with brine, dried over anhydrous MgSO_4_, filtered and concentrated. The residue was purified by open chromatography on Florisil (*n*‐hexane/EtOAc, 10:1 v/v) to give aldehyde **32 c** (232 mg, 1.35 mmol, 90 %). Pale yellow oil; ^1^H NMR (400 MHz, CDCl_3_): *δ=*9.78 (t, *J=*1.7 Hz, 1 H), 4.56 (t, *J=*3.6 Hz, 1 H), 3.80 (m, 1 H), 3.62 (dt, *J=*9.9, 6.0 Hz, 1 H), 3.48 (m, 1 H), 3.41 (dt, *J=*9.9, 6.0 Hz, 1 H), 2.53 (tt, *J=*6.9, 1.5 Hz, 2 H), 1.94 (quint, *J=*6.6 Hz, 2 H), 1.85–1.46 ppm (m, 6 H); ^13^C NMR (75 MHz, CDCl_3_): *δ=*202.2, 98.8, 66.4, 62.2, 41.1, 30.6, 25.4, 22.7, 19.5 ppm; IR (film on ZnSe): $\tilde{\nu }$ =2943, 2854, 1725, 1453, 1441, 1353, 1201, 1138, 1122 cm^−1^; MS (EI, 70 eV): *m*/*z* (%) 173 ([*M*+H]^+^, 14), 149 (37), 101 (85), 85 (100), 71 (100); HRMS (ESI+): *m*/*z*: calcd for C_9_H_17_O_3_: 173.1178 [*M*+H]^+^; found: 173.1215.

**2‐(Oxiran‐2‐yl)‐1‐phenylethan‐1‐ol (34 a).^[58]^** *m‐*CPBA (69 % purity, 301 mg, 1.20 mmol) was added to a solution of alcohol **33 a** (148 mg, 0.999 mmol) in 4‐MeTHP (5 mL). The mixture was stirred at 50 °C for 24 h and quenched successively by the addition of saturated aqueous Na_2_S_2_O_3_ (4 mL) and saturated aqueous NaHCO_3_ (4 mL) at 0 °C. The resulting mixture was extracted with EtOAc (2×10 mL). The combined organic layer was washed with brine, dried over anhydrous MgSO_4_, filtered and concentrated. The residue was purified by flash chromatography on silica gel (*n*‐hexane/EtOAc, 10:1→5:1 v/v) to give a 1.2:1 inseparable diastereomeric mixture of epoxide **34 a** (138 mg, 0.840 mmol, 84 %). Colorless oil; ^1^H NMR (400 MHz, CDCl_3_): *δ=*7.41–7.34 (m, 4 H), 7.32–7.26 (m, 1 H), 4.95 (m, 1 H), 3.18 (m, 0.55 H), 3.02 (m, 0.45 H), 2.84 (t, *J=*4.4 Hz, 0.55 H), 2.76 (t, *J=*4.4 Hz, 0.45 H), 2.62 (m, 0.55 H), 2.51 (m, 0.45 H), 2.15 (m, 0.55 H), 2.06 (m, 0.45 H), 1.90 (dt, *J=*14, 8.0 Hz, 0.45 H), 1.81 ppm (m, 0.55 H); ^13^C NMR (75 MHz, CDCl_3_): *δ=*144.2, 143.9 (minor), 128.58 (minor), 128.54, 127.79 (minor), 127.65, 125.87 (minor), 125.66, 72.7 (minor), 71.7, 50.3 (minor), 50.0, 47.2, 46.9 (minor), 41.8, 41.5 ppm (minor).

**((3a*R*,4*S*,5*S*,6*S*,7*R*,7a*R*)‐4‐Hydroxy‐2,2‐dimethyl‐7‐((triisopropylsilyl)oxy)tetrahydro‐4*H*‐spiro[benzo[*d*][1,3]dioxole‐5,2′‐oxiran]‐6‐yl)methyl pivalate (34 b)**.^59^ VO(acac)_2_ (1.7 mg, 6.4 μmol) and *t*BuOOH (70 % in water, 17 μL, 0.12 mmol) were sequentially added to a solution of allylic alcohol **33 b** 59 (49.6 mg, 0.105 mmol) in 4‐MeTHP (2 mL). The mixture was warmed to 40 °C and stirred for 27 h. The reaction mixture was quenched by the addition of saturated aqueous Na_2_S_2_O_3_ (2 mL) and extracted with EtOAc (10 mL). The organic layer was washed with brine, dried over anhydrous MgSO_4_, filtered and concentrated. The residue was purified by flash chromatography on silica gel (*n*‐hexane/EtOAc, 10:1 v/v) to give epoxide **34 b** (35.9 mg, 0.0738 mmol, 70 %). Pale yellow oil; ${\left[\alpha \right]}_{D}^{27}$ =+31.1 (*c*=1.01, CHCl_3_); ^1^H NMR (400 MHz, CDCl_3_): *δ=*4.58 (dd, *J=*6.9, 3.2 Hz, 1 H), 4.35 (m, 1 H), 4.28 (dd, *J=*7.0, 3.3 Hz, 1 H), 4.15 (t, *J=*2.8 Hz, 1 H), 4.07 (dd, *J=*11, 6.2 Hz, 1 H), 4.01 (dd, *J=*11, 8.5 Hz, 1 H), 2.79 (d, *J=*4.4 Hz, 1 H), 2.75 (d, *J=*4.4 Hz, 1 H), 2.60 (ddd, *J=*8.3, 6.2, 2.2 Hz, 1 H), 2.48 (br d, *J=*11 Hz, 1 H, OH), 1.51 (s, 3 H), 1.37 (s, 3 H), 1.18 (s, 9 H), 1.11–1.03 ppm (m, 21 H); ^13^C NMR (75 MHz, CDCl_3_): *δ=*178.2, 109.9, 77.0, 76.0, 70.1, 64.7, 61.2, 57.0, 46.2, 39.3, 38.8, 27.3, 26.9, 24.7, 18.2, 12.7 ppm; IR (film on ZnSe): $\tilde{\nu }$ =3485, 2943, 2868, 1732, 1464, 1385, 1283, 1211 cm^−1^; HRMS (APCI+): *m*/*z*: calcd for C_25_H_47_O_7_Si: 487.3086 [*M*+H]^+^; found: 487.3084.

**2‐(Cyclopent‐2‐en−1‐yl)‐*N*‐phenethylacetamide (36)**. EDCI (119 mg, 0.620 mmol), DMAP (74.8 mg, 0.612 mmol) and a solution of 2‐phenylethylamine (86.7 mg, 0.715 mmol) in 4‐MeTHP (2 mL) were successively added to a solution of carboxylic acid **35** (61.8 mg, 0.490 mmol) in 4‐MeTHP (3 mL). After 26 h at room temperature, the reaction mixture was quenched by the addition of 33 wt % aqueous citric acid. The resulting mixture was extracted with EtOAc (2×5 mL). The combined organic layer was washed with brine, dried over anhydrous MgSO_4_, filtered and concentrated. The residue was purified by flash chromatography on silica gel (*n*‐hexane/EtOAc, 7:1→5:1 v/v) to give amide **36** (80.5 mg, 0.351 mmol, 72 %). The NMR indicates that **36** exists as a mixture of two rotamers (3:1) by amide group. Colorless solid; ^1^H NMR (400 MHz, CDCl_3_): *δ=*7.34–7.29 (m, 2 H), 7.26–7.21 (m, 1 H), 7.21–7.19 (m, 2 H), 5.74 (dq, 1 H, *J=*5.8, 2.2 Hz, 0.75 H), 5.64–5.60 (m, 1.25 H), 5.44 (br s, 1 H), 3.54 (q, *J=*6.8 Hz, 2 H), 3.08 (m, 0.75 H), 2.82 (t, *J=*6.8 Hz, 2 H), 2.72–1.94 (m, 5.5 H), 1.40 ppm (m, 0.75 H); ^13^C NMR (100 MHz, CDCl_3_): *δ=*172.6 (minor), 172.3, 138.93 (minor), 138.92, 133.9, 131.3, 129.5 (minor), 128.7, 128.5, 126.4, 43.0 (minor), 42.7, 42.5, 40.6, 38.6 (minor), 35.7, 34.1 (minor), 31.8, 29.5 ppm; IR (film on ZnSe): $\tilde{\nu }$ =3292, 3062, 3028, 2932, 2849, 1651, 1557, 1454, 1362, 1294, 1275, 1198 cm^−1^; HRMS (APCI+): *m*/*z*: calcd for C_15_H_20_ON: 230.1539 [*M*+H]^+^; found: 230.1537.

**3,5‐Dimethoxybenzyl palmitate (37)**. Alcohol **15** (4.65 g, 27.7 mmol) and DMAP (169 mg, 1.38 mmol) were placed in a well‐dried 300 mL three‐necked round bottom flask, which was dissolved in 4‐MeTHP (70 mL). Pyridine (6.7 mL, 83 mmol) and palmitoyl chloride (9.2 mL, 30 mmol) were successively added and the mixture was stirred at room temperature for 30 min. The reaction mixture was quenched by the addition of saturated aqueous NaHCO_3_ and extracted with EtOAc (2×25 mL). The combined organic layer was washed with brine, dried over anhydrous MgSO_4_, filtered and concentrated. The residual solid was recrystallized from *n*‐hexane to give ester **37** (10.8 g, 26.6 mmol, 96 %) as a colorless solid. The mother liquid was further purified by flash chromatography on silica gel (*n‐*hexane→*n‐*hexane/EtOAc, 30:1 v/v) to give **37** (447 mg, 1.10 mmol, 4 %). ^1^H NMR (400 MHz, CDCl_3_): *δ=*6.49 (d, *J=*2.4 Hz, 2 H), 6.41 (t, *J=*2.4 Hz, 1 H), 5.05 (s, 2 H), 3.79 (s, 3 H), 2.36 (t, *J=*7.6 Hz, 2 H), 1.68–1.60 (m, 2 H), 1.33–1.21 (m, 24 H), 0.88 ppm (t, *J=*6.8 Hz, 3 H); ^13^C NMR (100 MHz, CDCl_3_): *δ=*173.8, 161.0, 138.5, 106.0, 100.2, 66.1, 55.5, 34.5, 32.1, 29.84, 29.82, 29.80, 29.75, 29.6, 29.5, 29.4, 29.3, 25.1, 22.8, 14.3 ppm; IR (film on ZnSe): $\tilde{\nu }$ =3000, 2925, 2853, 1739, 1610, 1599, 1466, 1430, 1350, 1322, 1298, 1206, 1157, 1071 cm^−1^; HRMS (ESI+): *m*/*z*: calcd for C_25_H_43_O_4_: 407.3156 [*M*+H]^+^; found 407.3141.

**Diethyl cyclopent‐3‐ene‐1,1‐dicarboxylate (39).^[74]^** The Grubbs catalyst^®^ 1^st^ generation (12.3 mg, 0.0149 mmol) was added to a solution of diene **38** (240 mg, 0.997 mmol) in 4‐MeTHP (20 mL). The mixture was stirred at room temperature for 2 h and quenched by the addition of Et_3_N (275 μL, 1.97 mmol). After concentration, the residue was purified by flash chromatography on silica gel (*n*‐hexane/EtOAc, 100:1 v/v) to give ester **39** (195 mg, 0.918 mmol, 92 %). Brownish oil; ^1^H NMR (400 MHz, CDCl_3_): *δ=*5.61 (s, 2 H), 4.20 (q, *J=*7.0 Hz, 4 H), 3.01 (s, 4 H), 1.25 ppm (t, *J=*7.0 Hz, 6 H); ^13^C NMR (75 MHz, CDCl_3_): *δ=*172.2, 127.8, 61.5, 58.9, 40.9, 14.1 ppm.

## Supporting information

As a service to our authors and readers, this journal provides supporting information supplied by the authors. Such materials are peer reviewed and may be re‐organized for online delivery, but are not copy‐edited or typeset. Technical support issues arising from supporting information (other than missing files) should be addressed to the authors.

Supplementary
