# Supplementary material for: 4‐Methyltetrahydropyran (4‐MeTHP): Application as an Organic Reaction Solvent
Source: Chem Asian J. 2019 Oct 16;14(21):3921–37. doi: 10.1002/asia.201901169 (PMC6916367; doi:10.1002/asia.201901169)

CHEMISTRY
AN ASIAN JOURNAL

Supporting Information

4-Methyltetrahydropyran (4-MeTHP): Application as an Organic Reaction Solvent

Shoji Kobayashi,* Tomoki Tamura, Saki Yoshimoto, Takashi Kawakami, and Araki Masuyama[a]

asia_201901169_sm_miscellaneous_information.pdf

1. A PRICE LIST OF COMMERCIALLY AVAILABLE SOLVENTS. S2

2. SOLVENT RECOVERY EXPERIMENTS AND GC-MS ANALYSES. S3

2.1. ANALYTICAL CONDITIONS S3

2.2. RADICAL ADDITION IN 4-METHP S4

2.3. RADICAL ADDITION IN THP S7

2.4. RADICAL ADDITION IN 2-METHF. S10

3. ^{1}H AND ^{13}C NMR SPECTRA OF SYNTHETIC COMPOUNDS. S13

1. A price list of commercially available solvents

Table S1. Price of commercially available solvents[a]

Supplier	Solvent	Grade	Content	Price	
				JPY	USD
Sigma-Aldrich[b]	4-MeTHP	- (unavailable)	-	-	-
	THP	99% (anhydrous)	100 mL	22,400	178.00
	CPME	≧99.90%[e] (ReagentPlus®)	500 mL	11,100	82.40
	2-MeTHF	≧99.5%[e] (ReagentPlus®)	500 mL	20,600	143.00
	THF	≧99.0%[e] (ACS reagent)	500 mL	10,600	84.90
TCI[c]	4-MeTHP	> 99.0% (GC)[e]	500 mL	3,500	-
	THP	> 98.0% (GC)	500 mL	22,300	126.00
	CPME	> 99.5% (GC)[e]	500 mL	3,600	33.00
	2-MeTHF	> 98.0% (GC)[e]	500 mL	10,900	70.00
	THF	> 99.5% (GC)[e]	500 mL	3,100	24.00
Fujifilm Wako[d]	4-MeTHP	> 99.0% (Capillary GC)[e]	500 mL	3,500	39.00
	THP	> 98.0% (Capillary GC)	500 mL	35,000	385.00
	CPME	> 98.0% (Capillary GC)[e]	500 mL	3,600	40.00
	2-MeTHF	> 98.0% (Capillary GC)[e]	500 mL	18,000	198.00
	THF	> 99.5% (Capillary GC)	500 mL	1,950	22.00

[a] The data were collected on September 11, 2019. A safety data sheet (SDS) of solvent is available from the supplier's web page.
[b] https://www.sigmaaldrich.com/united-states.html https://www.sigmaaldrich.com/japan.html
[c] https://www.tcichemicals.com/en/us/index.html https://www.tcichemicals.com/en/jp/index.html
[d] https://labchem-wako.fujifilm.com/us/index.html https://labchem-wako.fujifilm.com/jp/index.html
[e] Stabilized with BHT.

2. Solvent recovery experiments and GC-MS analyses

2.1. Analytical conditions

GC conditions

Gas-phase chromatography was performed on a GC-2014 instrument using a DB-WAX column (30 m × 0.25 mm × 0.25 μm film thickness). A temperature of FID detector was adjusted to 250 °C. The oven was heated at 50 °C for 5 min followed by a temperature gradient of 10 °C/min to 200 °C and, finally, at 200 °C for 5 min. Inlet temperature and pressure were 200 °C and 83 kPa, respectively, with a split ratio of 100:1. Helium was used as the carrier gas at a flow rate of 1.0 mL/min.

GC-MS conditions

The gas chromatograph-mass spectrometry model GCMS-QP2010 Ultra (SHIMADZU CORPORATON, Japan), equipped with DB-WAX capillary column with dimensions 30 m × 0.25 mm × 0.25 μm, was used. The oven was maintained at 50 °C for 5 min followed by a temperature gradient of 10 °C/min to 200 °C and, finally, at 200 °C for 5 min. The injection volume of the sample was 0.2 μL with a split ratio of 100:1, using helium as the carrier gas at a flow rate of 1.0 ml/min. Injector temperature was maintained at 200°C. Detector temperature was maintained at 230 °C. The percentage composition was calculated using peak normalization method assuming equal detector response. The samples were analyzed with an electron impact ionization at 70 eV. The compounds separated were characterized from their mass spectral data using the NIST14 mass spectral library.

2.2. Radical addition in 4-MeTHP

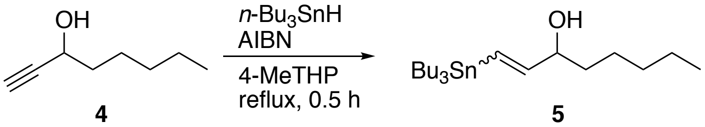

4 5

Experimental procedure

Commercially available 4-MeTHP (stabilized with BHT) was stored with molecular sieves 4Å and used for the reaction (sample 1). Oct-1-yn-3-ol (**4**) (126 mg, 0.996 mmol), *n*-Bu_3SnH (378 mg, 1.30 mmol), and AIBN (32.8 mg, 0.200 mmol) were placed in a 100 mL two-necked round bottom flask equipped with a reflux condenser. The flask was flushed with argon and 4-MeTHP (20 mL) was added. After complete dissolution of AIBN at room temperature, the solution was heated at reflux for 30 min. The solution was stood at room temperature and, after attachment of the distillation apparatus, the solvent was distilled under reduced pressure (bp 38-40 °C/8-10 kPa) (sample 2). The residual oil was purified by flash chromatography on silica gel (*n*-hexane/EtOAc/Et_3N = 50/1/0.5) to give stannane **5** (288 mg, 0.691 mmol, 69%, *E/Z*=16:1) as a pale yellow oil. The purity of the recovered 4-MeTHP was determined to be 99.94% by the GC analysis. The same experiment was performed with the recovered 4-MeTHP, providing the product **5** (64% yield, *E/Z*=8:1) with a recovery of 4-MeTHP (purity: 99.88%) (sample 3).

Compound characterization list analyzed by GC-MS[a]

**t (min)**	2.2	2.5	2.9	3.6	4.1	4.5
**name**	acetone[b]	unknown	unknown	4-MeTHP	isobutyronitrile	unknown
**structure**	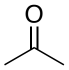			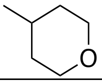	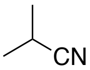	
**#**					**A**	
**sample 1**	-	0.03%	0.19%	99.21%	-	0.05%
**sample 2**	0.01%	0.06%	0.15%	98.54%	0.37%	0.05%
**sample 3**	0.02%	0.14%	0.13%	98.20%	0.62%	0.05%
**t (min)**	5.3	5.8	7.6	9.0	16.0	19.3
**name**	water	isopentyl formate	1-butanol	isopentanol	AIBN	BHT
**structure**	H_2O	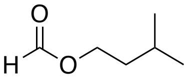	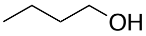	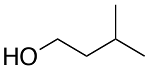	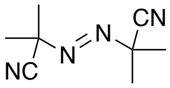	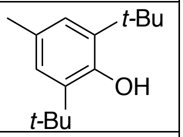
**#**	**B**	**C**	**D**	**E**		
**sample 1**	0.27%	0.06%	-	-	-	0.09%
**sample 2**	0.42%	0.09%	0.09%	0.03%	0.06%	-
**sample 3**	0.38%	0.13%	0.17%	0.06%	0.04%	-
**t (min)**						
**name**						
**structure**						
**#**						
**sample 1**						
**sample 2**						
**sample 3**						

[a] Percentage in the table refers to the peak area percentage in GC-MS.
[b] Derived from washing of GC-MS.

GC spectra

(a) sample 1

C-R8A CHROMATOPAC CH=1 DATA=1:@CHRM1.C00 ATTEN= 4 SPEED= 5.0

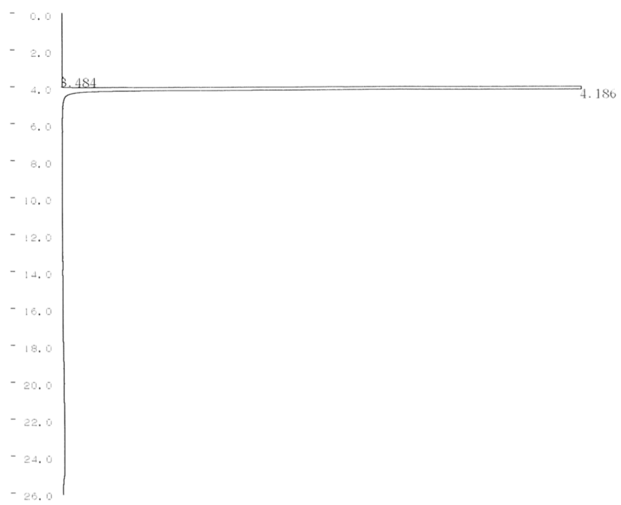

C-R8A CHROMATOPAC CH=1 Report No.=70 DATA=1:@CHRM1.C00 17/09/29 08:50:30

** CALCULATION REPORT **

CH	PKNO	TIME	AREA	HEIGHT	MK	IDNO	CONC	NAME
1	1	3.484	109	41			0.012	
	2	4.186	910963	197676			99.988	
	TOTAL		911072	197717			100	

(b) sample 2

C-R8A CHROMATOPAC CH=1 Report No.=72 DATA=1:@CHRM1.C00 17/09/29 09:27:46

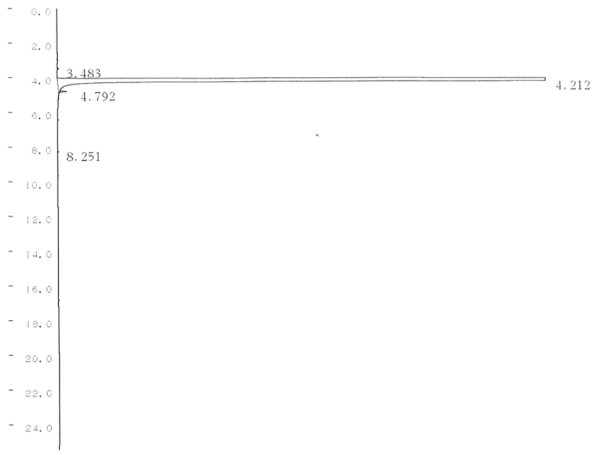

** CALCULATION REPORT **

CH	PKNO	TIME	AREA	HEIGHT	MK	IDNO	CONC	NAME
1	2	3.483	180	60			0.012	
	3	4.212	1502166	255984	S		99.9425	
	4	4.792	583	225	T		0.0388	
	5	8.251	100	39			0.0067	
	TOTAL		1503030	256308			100	

(c) sample 3

C-R8A CHROMATOPAC CH=1 DATA=1:@CHRM1.C00 ATTEN= 4 SPEED= 5.0

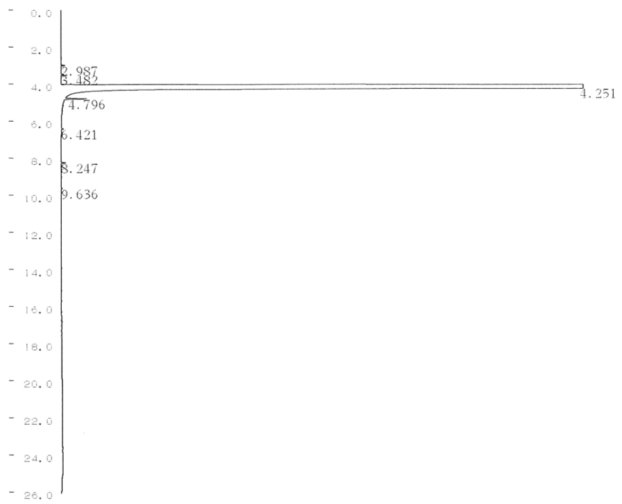

C-R8A CHROMATOPAC CH=1 Report No.=74 DATA=1:@CHRM1.C00 17/09/29 10:36:20

** CALCULATION REPORT **

CH	PKNO	TIME	AREA	HEIGHT	MK	IDNO	CONC	NAME
1	1	2.987	304	136			0.0129	
	2	3.482	287	90			0.0122	
	3	4.251	2344774	330156	S		99.8784	
	4	4.796	1619	660	T		0.069	
	5	6.421	211	76			0.009	
	6	8.247	332	134			0.0141	
	7	9.636	103	41			0.0044	
	TOTAL		2347629	331293			100	

GC-MS spectra

(a) sample 1

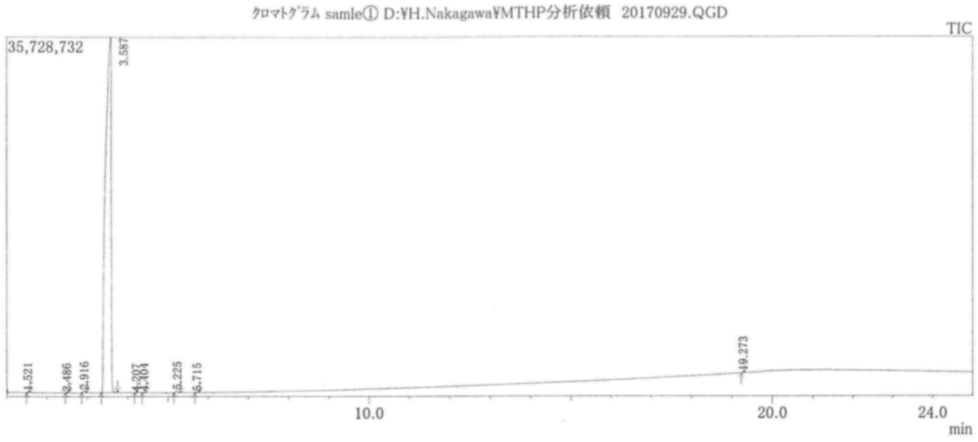

(b) sample 2

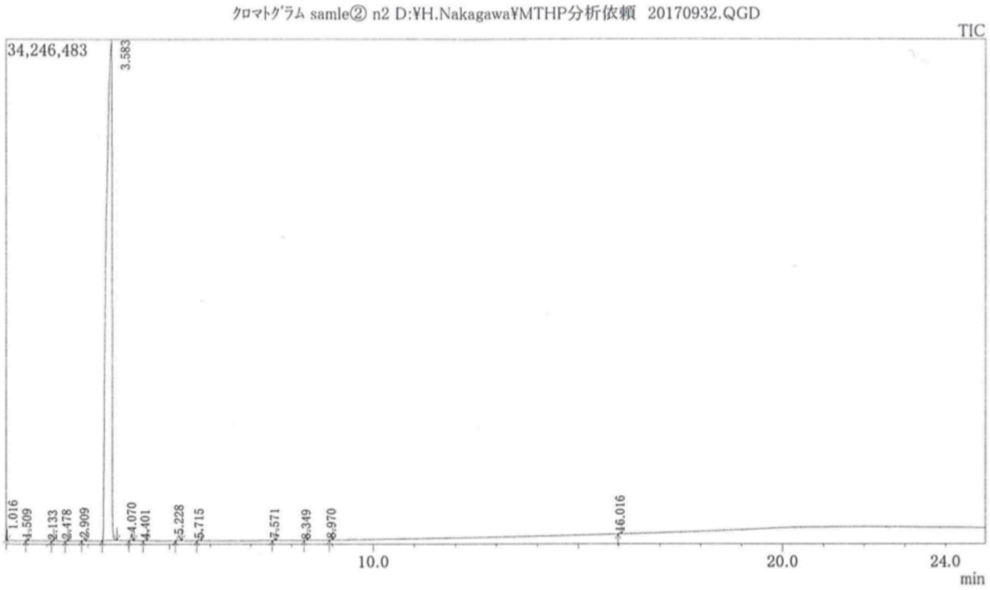

(c) sample 3

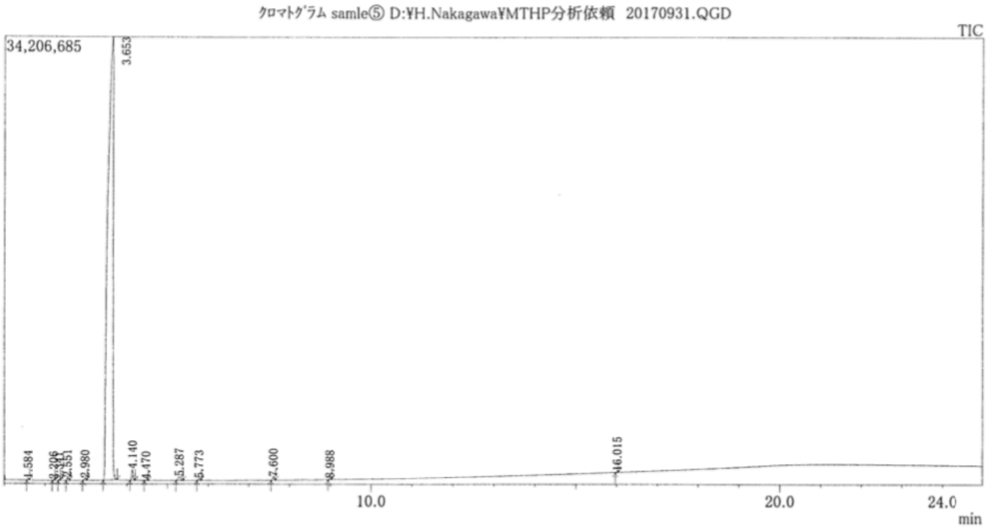

2.3. Radical addition in THP

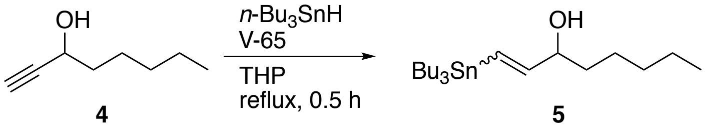

Experimental procedure

Commercially available THP (without stabilizer) was used for the reaction without any pretreatment (sample 4). Oct-1-yn-3-ol (**4**) (126 mg, 0.997 mmol), *n*-Bu_3SnH (380 mg, 1.31 mmol), and V-65 (50.1 mg, 0.202 mmol) were placed in a 100 mL two-necked round bottom flask equipped with a reflux condenser. The flask was flushed with argon and THP (20 mL) was added. After complete dissolution of AIBN at room temperature, the solution was heated at reflux for 30 min. The solution was stood at room temperature and, after attachment of the distillation apparatus, the solvent was distillated under reduced pressure (bp 35-39 °C/17-21 kPa) (sample 5). The residual oil was purified by flash chromatography on silica gel (*n*-hexane/EtOAc/Et_3N = 100/1/1) to give stannane **5** (325 mg, 0.779 mmol, 78%, *E/Z*=10:1) as a pale yellow oil. The purity of the recovered THP was determined to be 99.75% by the GC analysis. The same experiment was performed with the recovered THP, providing the product **5** (79% yield, *E/Z*=10:1) with a recovery of THP (purity: 99.77%) (sample 6).

Compound characterization list analyzed by GC-MS[a]

**t (min)**	2.1	2.5	2.96	3.03	3.5	4.4
**name**	acetone[b]	2-MeTHF	THP	2-MeTHP	3-MeTHP	*n*-butyl formate
**structure**	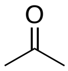	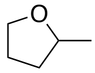	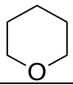	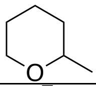	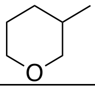	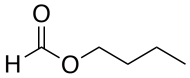
**#**				**F**	**G**	**H**
**sample 4**	0.64%	0.34%	93.60%	1.67%	1.51%	0.46%
**sample 5**	0.07%	0.44%	94.89%	1.56%	1.22%	0.44%
**sample 6**	0.02%	0.40%	96.07%	1.20%	0.85%	0.28%
**t (min)**	5.2	7.6	8.8	14.4	15.6	18.2
**name**	water	1-butanol	2,4-dimethylpentanenitrile	unknown	unknown	δ-lactone
**structure**	H_2O	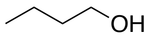	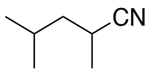			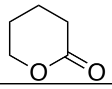
**#**	**B**	**D**	**I**			
**sample 4**	0.54%	0.16%	0.36%	0.16%	0.36%	0.11%
**sample 5**	0.60%	0.05%	0.10%	-	-	-
**sample 6**	0.39%	0.05%	0.13%	-	-	-
**t (min)**						
**name**						
**structure**						
**#**						
**sample 4**						
**sample 5**						
**sample 6**						

[a] Percentage in the table refers to the peak area percentage in GC-MS.
[b] Derived from washing of GC-MS.

GC spectra

(d) sample 4

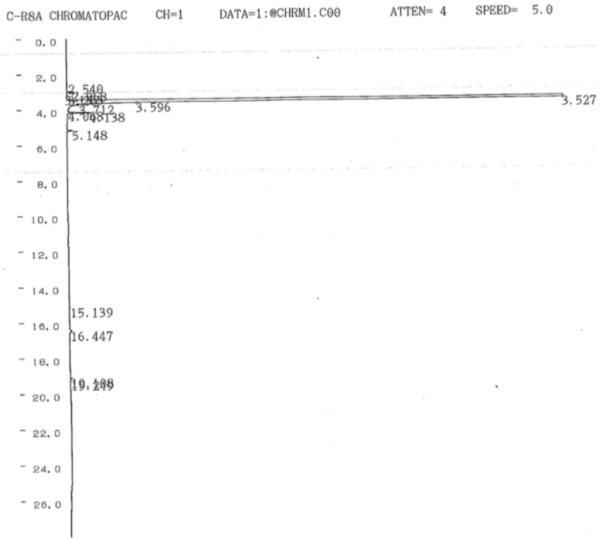

C-R8A CHROMATOPAC CH=1 Report No.=222 DATA=1:@CHRM1.C00 17/12/11 10:59:12

** CALCULATION REPORT **

CH	PKNO	TIME	AREA	HEIGHT	MK	IDNO	CONC	NAME
1	1	2.54	106	50			0.0081	
	2	2.968	491	227			0.0376	
	3	3.137	33	14			0.0025	
	4	3.203	139	63	V		0.0106	
	5	3.527	1301279	354011	S		99.6302	
	6	3.596	530	393	T		0.0406	
	7	3.712	257	137	T		0.0197	
	8	4.068	86	40	T		0.0065	
	9	4.138	1973	767	TV		0.1511	
	10	5.148	544	184			0.0417	
	11	15.139	72	33			0.0055	
	12	16.447	414	68			0.0317	
	13	19.108	148	64			0.0113	
	14	19.249	39	19			0.003	
	TOTAL		1306109	356069			100	

(e) sample 5

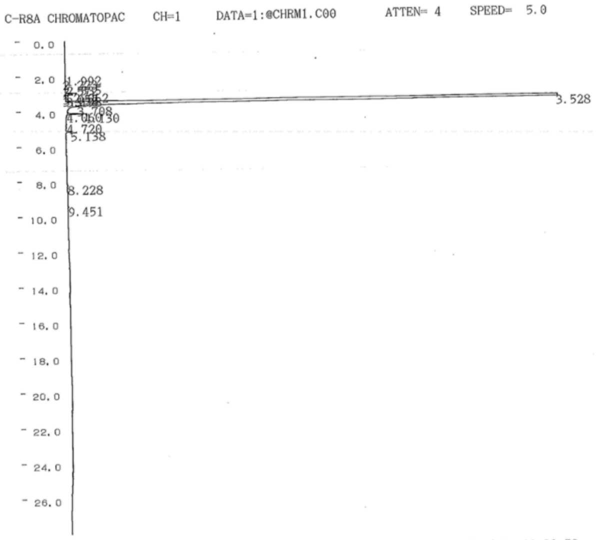

C-R8A CHROMATOPAC CH=1 Report No.=223 DATA=1:@CHRM1.C00 17/12/11 12:30:58

** CALCULATION REPORT **

CH	PKNO	TIME	AREA	HEIGHT	MK	IDNO	CONC	NAME
1	1	1.992	37	29			0.0025	
	3	2.535	98	62			0.0067	
	4	2.572	27	17	V		0.0019	
	5	2.962	700	365			0.0484	
	6	3.052	25	13	V		0.0017	
	7	3.138	28	13			0.0019	
	8	3.198	67	35	V		0.0046	
	9	3.528	1443828	377256	S		99.7453	
	10	3.708	292	166	T		0.0202	
	11	4.06	77	36	T		0.0053	
	12	4.13	1594	658	TV		0.1101	
	13	4.72	36	15			0.0025	
	14	5.138	539	195			0.0372	
	15	8.228	56	24			0.0039	
	16	9.451	114	45			0.0078	
	TOTAL		1447516	378928			100	

(f) sample 6

C-R8A CHROMATOPAC CH=1 Report No.=226 DATA=1:@CHRM1.C00 17/12/11 13:48:40

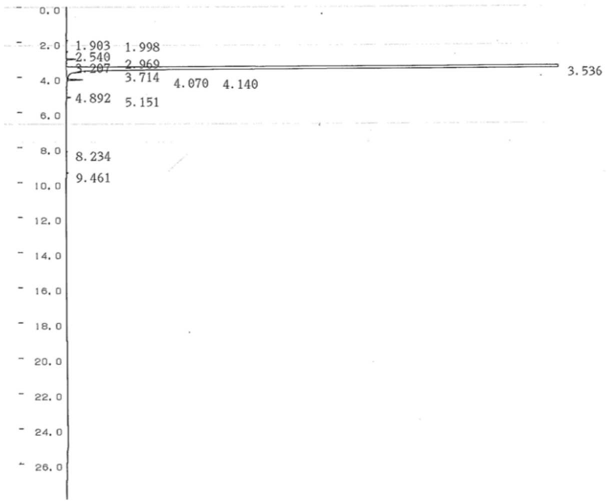

** CALCULATION REPORT **

CH	PKNO	TIME	AREA	HEIGHT	MK	IDNO	CONC	NAME
1	1	1.903	81	42			0.0052	
	2	1.998	31	18			0.002	
	3	2.54	140	50			0.0091	
	4	2.969	743	304			0.0482	
	5	3.207	48	21			0.0031	
	6	3.536	1537225	389583	S		99.7747	
	7	3.714	310	164	T		0.0201	
	8	4.07	59	27	T		0.0038	
	9	4.14	1297	491	TV		0.0842	
	10	4.892	40	15			0.0026	
	11	5.151	422	142			0.0274	
	12	8.234	100	41			0.0065	
	13	9.461	200	75			0.013	
	TOTAL		1540696	390972			100	

GC-MS spectra

(d) sample 4

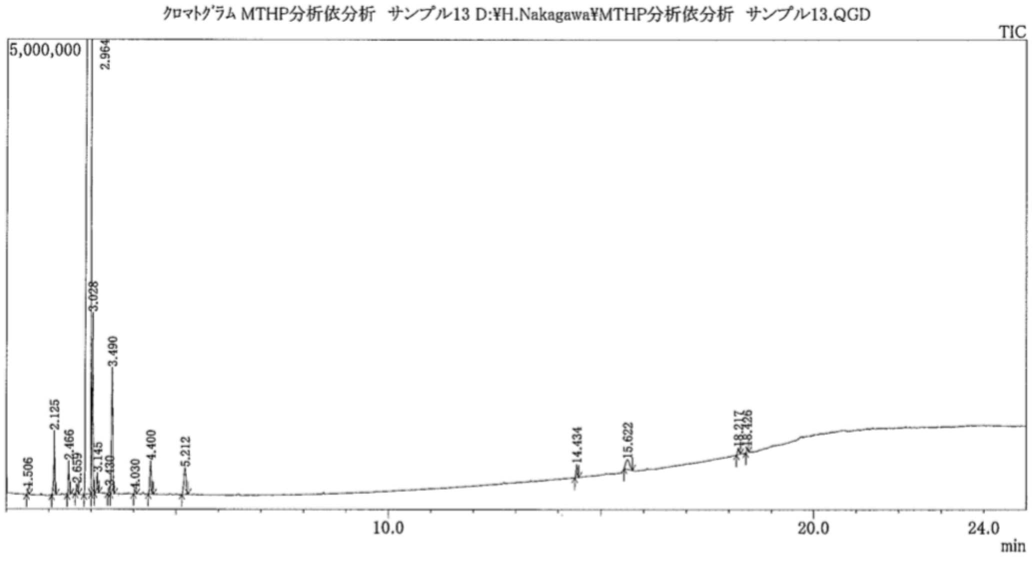

(e) sample 5

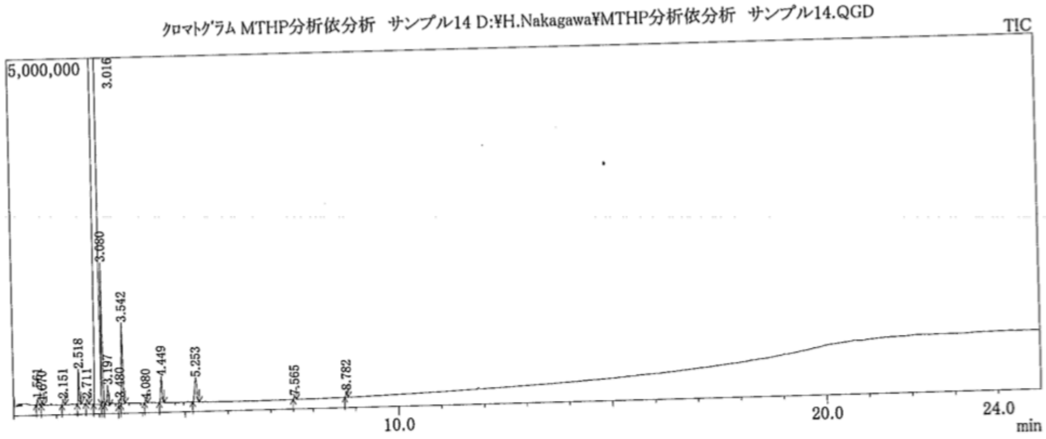

(f) sample 6

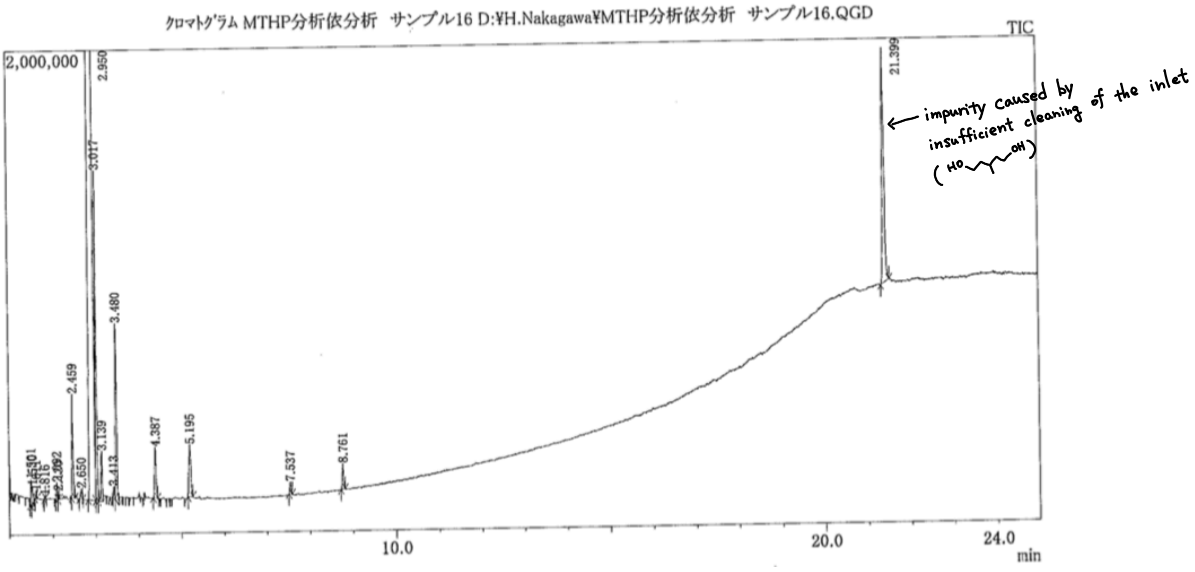

2.4. Radical addition in 2-MeTHF

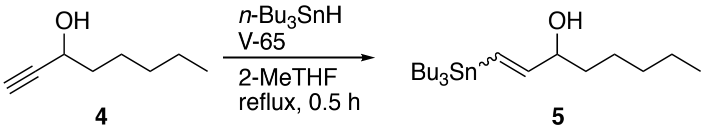

Experimental procedure

Commercially available 2-MeTHF (stabilized with BHT) was stored with molecular sieves 4Å and used for the reaction (sample 7). Oct-1-yn-3-ol (**4**) (126 mg, 0.998 mmol), *n*-Bu_3SnH (379 mg, 1.30 mmol), and V-65 (49.8 mg, 0.201 mmol) were placed in a 100 mL two-necked round bottom flask equipped with a reflux condenser. The flask was flushed with argon and 2-MeTHF (20 mL) was added. After complete dissolution of V-65 at room temperature, the solution was heated at reflux for 30 min. The solution was stood at room temperature and, after attachment of the distillation apparatus, the solvent was distilled under reduced pressure (bp 40-42 °C/25-28 kPa) (sample 8). The residual oil was purified by flash chromatography on silica gel (*n*-hexane/EtOAc/Et_3N = 30/1/0.3) to give stannane **5** (324 mg, 0.821 mmol, 82%, *E/Z*=9:1) as a pale yellow oil. The purity of the recovered 2-MeTHF was determined to be 99.96% by the GC analysis. The same experiment was performed with the recovered 2-MeTHF, providing the product **5** (84% yield, *E/Z*=10:1) with a recovery of 2-MeTHF (purity: 99.59%) (sample 9).

Compound characterization list analyzed by GC-MS[a]

t (min)	2.5	3.2	3.6	4.2	4.6
name	2-MeTHF	*sec*-butyl formate	*n*-propyl acetate	unknown	2-butanol
structure	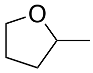	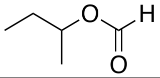	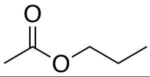		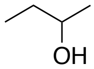
#		J	K	L	M
sample 7	96.20%	-	-	-	0.24%
sample 8	96.79%	0.11%	0.33%	-	0.20%
sample 9	88.46%	1.55%	3.28%	0.09%	0.09%

t (min)	5.2	8.8	13.5	15.7	16.9
name	water	2,4-dimethylpentanenitrile	5-methyltetrahydrofuran-2-ol (+5-methyl-2,3-dihydrofuran)	γ-valerolactone	5-hydroxypentan-2-one
structure	H_2O	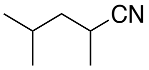	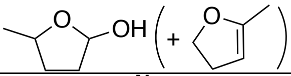	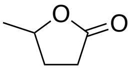	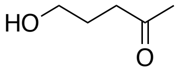
#	B	I	N	O	P
sample 7	1.10%	-	-	-	-
sample 8	1.43%	0.42%	0.04%	0.11%	-
sample 9	0.66%	0.19%	1.91%	0.17%	2.84%

t (min)	19.3	21.4			
name	BHT				
structure	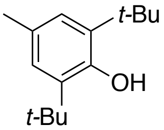	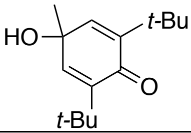			
#					
sample 7	1.40%	0.41%			
sample 8	-	-			
sample 9	-	-			

[a] Percentage in the table refers to the peak area percentage in GC-MS.

GC spectra

(g) sample 7

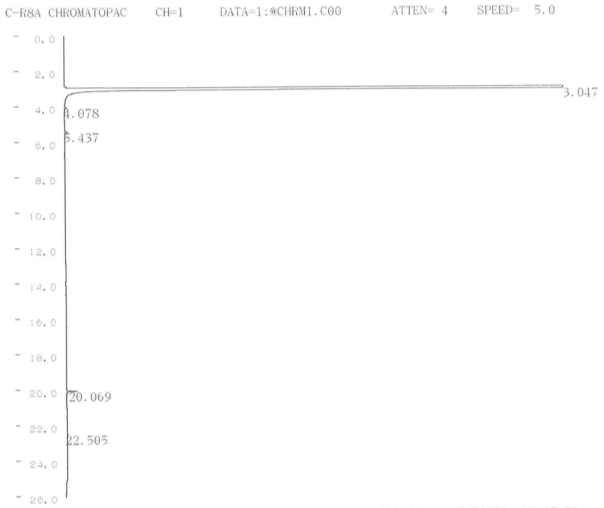

C-R8A CHROMATOPAC CH=1 Report No.=75 DATA=1:@CHRM1.C00 17/09/29 11:17:58

** CALCULATION REPORT **

CH	PKNO	TIME	AREA	HEIGHT	MK	IDNO	CONC	NAME
1	1	3.047	2147333	629114			99.9321	
	2	4.078	215	81			0.01	
	3	5.437	277	100			0.0129	
	4	20.069	867	351			0.0404	
	5	22.505	100	35			0.0047	
		TOTAL	2148793	629681			100	

(h) sample 8

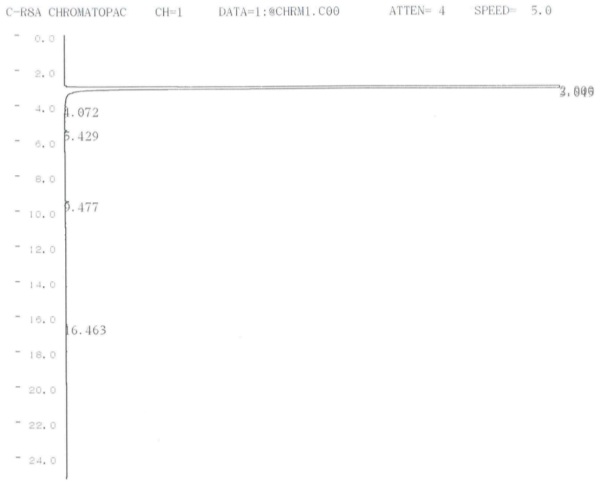

C-R8A CHROMATOPAC CH=1 Report No.=76 DATA=1:@CHRM1.C00 17/09/29 12:16:10

** CALCULATION REPORT **

CH	PKNO	TIME	AREA	HEIGHT	MK	IDNO	CONC	NAME
1	1	2.9	299	77			0.0132	
	2	3.045	2259035	661781	V		99.9576	
	3	4.072	120	45			0.0053	
	4	5.429	203	74			0.009	
	5	9.477	337	123			0.0149	
		TOTAL	2259994	662099			100	

(i) sample 9

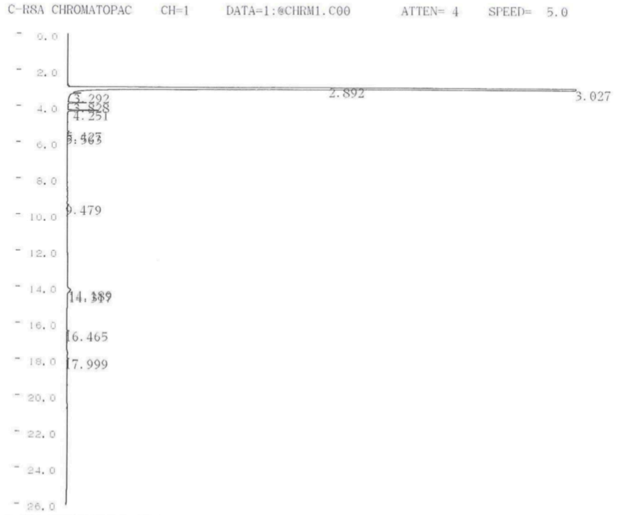

C-R8A CHROMATOPAC CH=1 Report No.=77 DATA=1:@CHRM1.C00 17/09/29 12:50:40

** CALCULATION REPORT **

CH	PKNO	TIME	AREA	HEIGHT	MK	IDNO	CONC	NAME
1	1	2.892	176	65			0.0114	
	2	3.027	1539656	528535	SV		99.5878	
	3	3.292	493	263	T		0.0319	
	4	3.828	1338	579			0.0866	
	5	4.251	2471	976			0.1598	
	6	5.427	172	63			0.0111	
	8	9.479	148	55			0.0096	
	9	14.189	964	103			0.0624	
	10	14.317	256	79	V		0.0166	
	11	16.465	190	80			0.0123	
	12	17.999	165	70			0.0106	
		TOTAL	1546028	530866			100	

GC-MS spectra

(g) sample 7

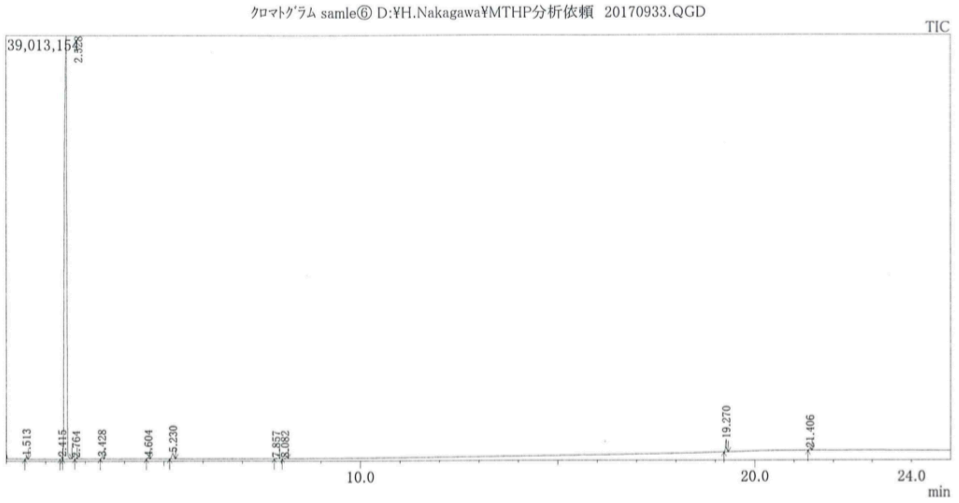

(h) sample 8

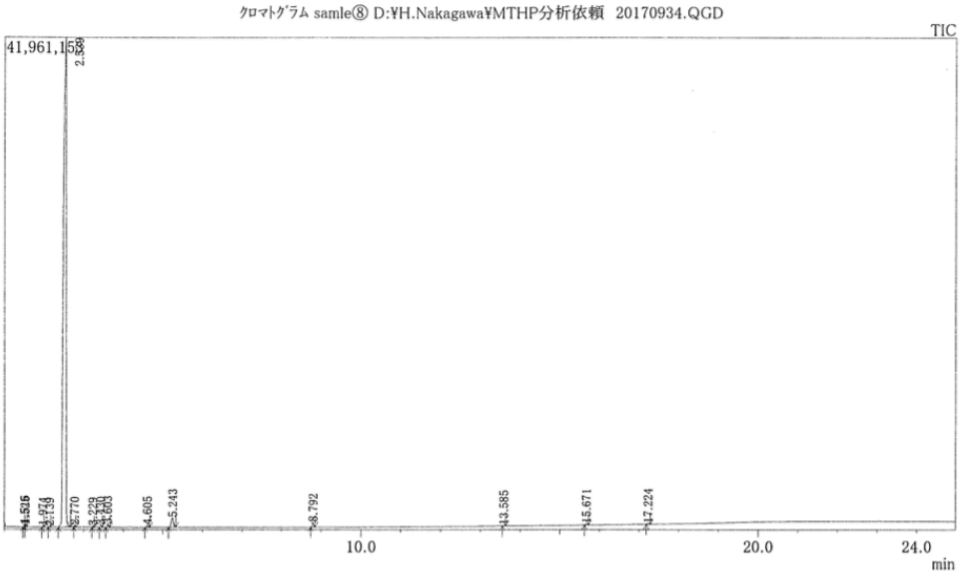

(i) sample 9

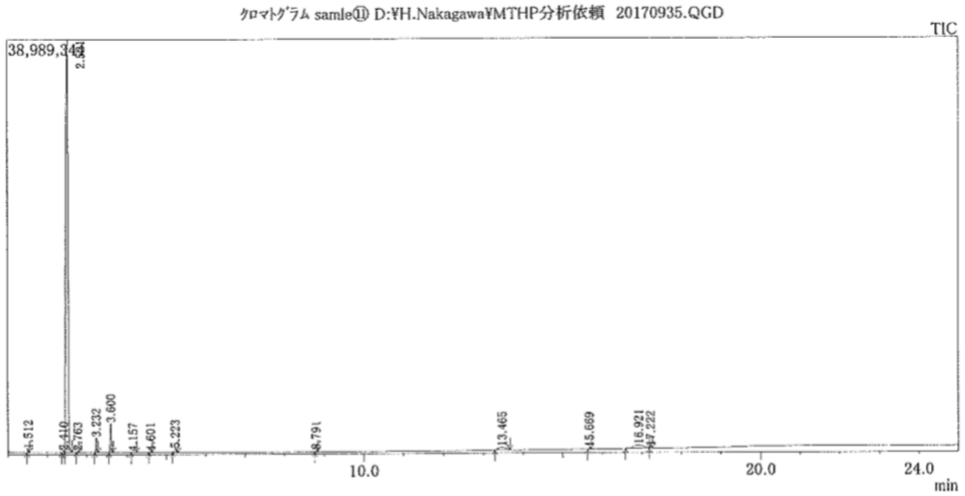

3. ^{1}H and ^{13}C NMR spectra of synthetic compounds

2019/02/28 17:29:39

Acquisition Time (sec)	3.5561	*Comment*	STANDARD 1H OBSERVE			*Date*	Jul 6 2017
Date Stamp	Jul 6 2017	*File Name*	¥¥Mac¥iCloud¥[illegible]NMR¥[illegible]¥2017NMR_K¥2017NMR(agilent)¥tamura¥TT17070602.fid¥fid				
Frequency (MHz)	300.05	*Nucleus*	1H	*Number of Transients*	16	*Original Points Count*	17080
Points Count	65536	*Pulse Sequence*	s2pul	*Receiver Gain*	28.00	*Solvent*	CHLOROFORM-d
Spectrum Offset (Hz)	1698.4518	*Spectrum Type*	STANDARD	*Sweep Width (Hz)*	4803.07	*Temperature (degree C)*	AMBIENT TEMPERATURE

TT17070602

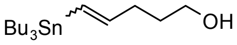

2aa

E/Z = 4:1

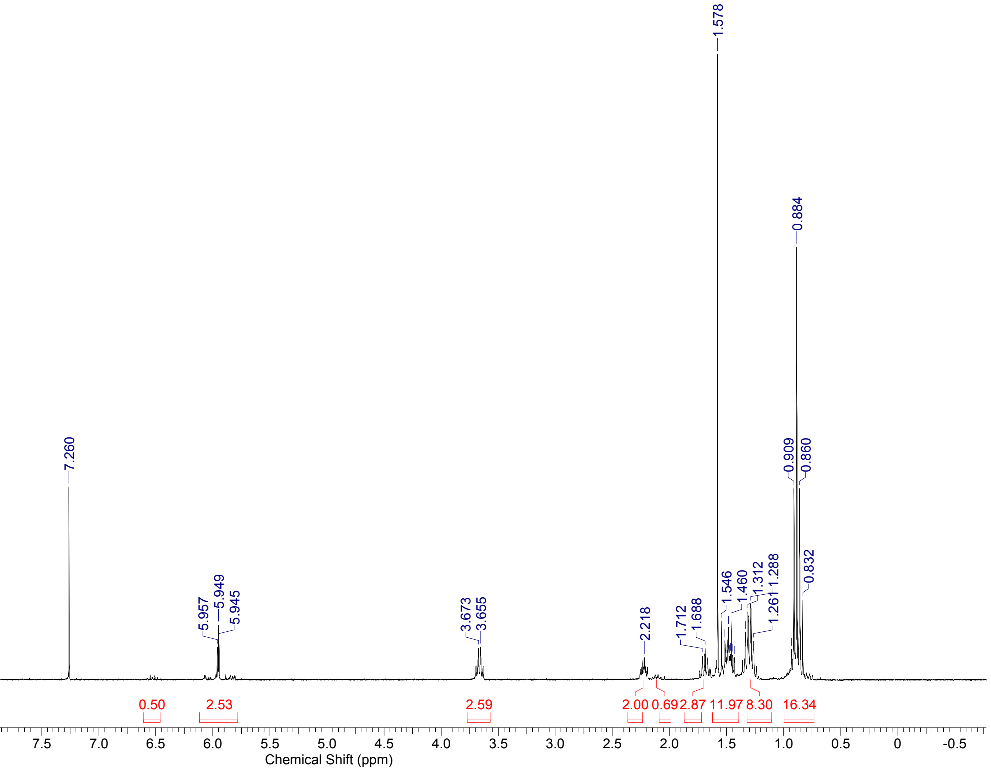

2019/03/14 16:36:06

Acquisition Time (sec)	0.8527	*Comment*	c13-NMR	*Date*	Mar 14 2019	*Date Stamp*	Mar 14 2019
File Name	¥¥Mac¥iCloud¥[illegible]NMR¥[illegible]¥2019NMR_K¥koba¥SK19031401_C.fid¥fid					*Frequency (MHz)*	75.46
Nucleus	13C	*Number of Transients*	128	*Original Points Count*	19335	*Points Count*	65536
Pulse Sequence	s2pul	*Receiver Gain*	30.00	*Solvent*	CHLOROFORM-d	*Spectrum Offset (Hz)*	8956.2578
Spectrum Type	STANDARD	*Sweep Width (Hz)*	22675.74	*Temperature (degree C)*	AMBIENT TEMPERATURE		

SK19031401_C

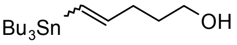

2aa

E/Z = 4:1

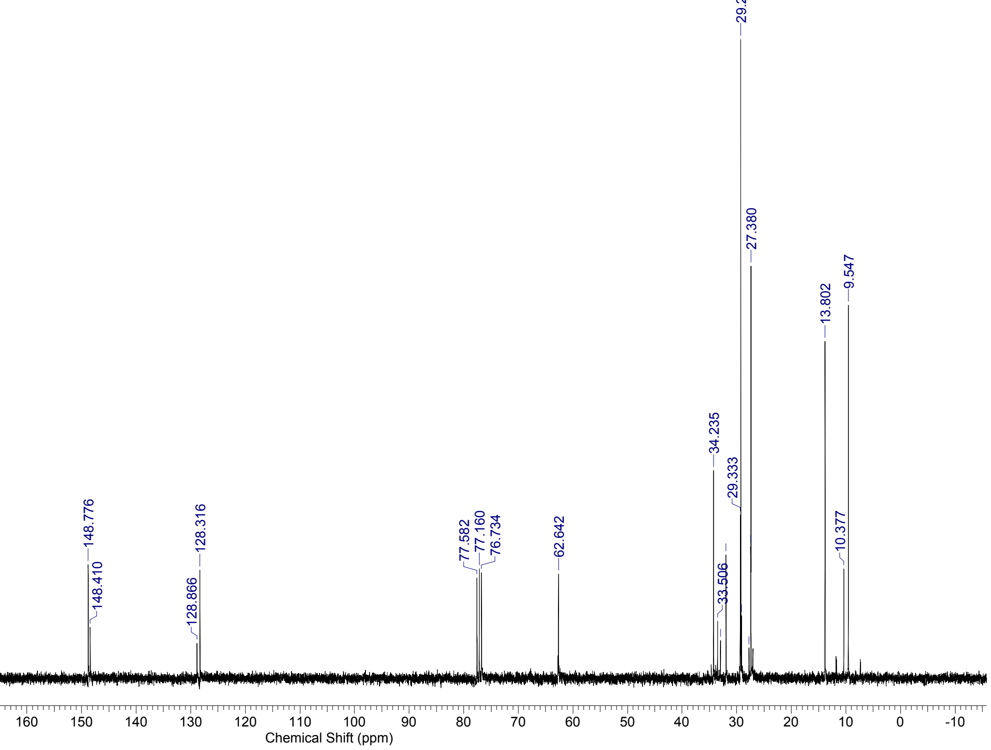

2019/03/01 10:58:30

Acquisition Time (sec)	2.1863	*Comment*	single_pulse	*Date*	23 Jan 2018 12:28:31		
Date Stamp	23 Jan 2018 12:27:40						
File Name	¥¥Mac¥iCloud¥[illegible]NMR[illegible]¥2017NMR_K¥2017NMR(JEOL)¥[illegible]¥TT18012302_Proton-1-1.jdf					*Frequency (MHz)*	399.78
Nucleus	1H	*Number of Transients*	8	*Origin*	ECA	*Original Points Count*	16384
Owner	delta	*Points Count*	32768	*Pulse Sequence*	proton.jxp	*Solvent*	CHLOROFORM-d
Spectrum Offset (Hz)	2003.4902	*Sweep Width (Hz)*	7494.00	*Temperature (degree C)*	18.400		

TT18012302_Proton-1-1.jdf

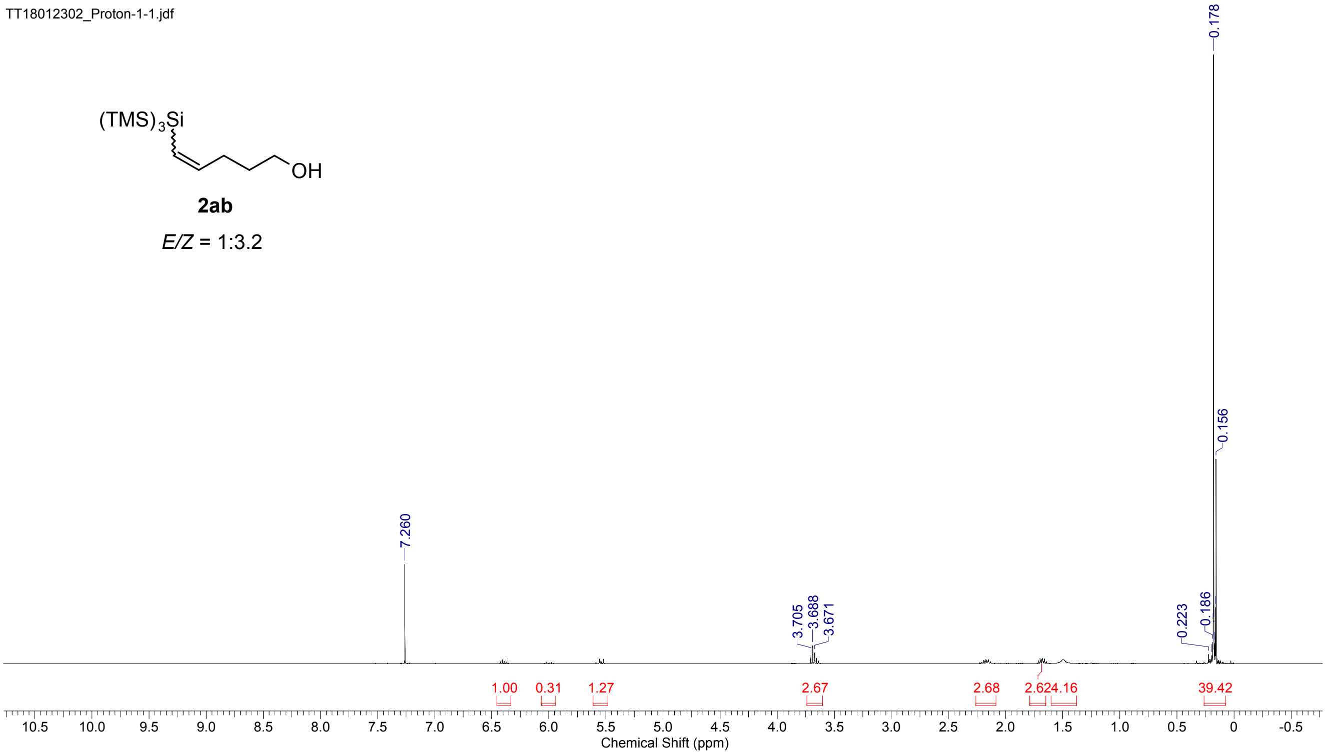

2019/03/13 11:21:27

Acquisition Time (sec)	0.8527	*Comment*	c13-NMR	*Date*	Mar 12 2019	*Date Stamp*	Mar 12 2019		
File Name	¥¥Mac¥iCloud¥[illegible]NMR[illegible]¥2019NMR_K¥koba¥SK19031202_C.fid¥fid					*Frequency (MHz)*	75.46	*Nucleus*	13C
Number of Transients	128	*Original Points Count*	19335	*Points Count*	65536	*Pulse Sequence*	s2pul	*Receiver Gain*	30.00
Solvent	CHLOROFORM-d			*Spectrum Offset (Hz)*	8956.9492	*Spectrum Type*	STANDARD	*Sweep Width (Hz)*	22675.74
Temperature (degree C)	AMBIENT TEMPERATURE								

SK19031202_C

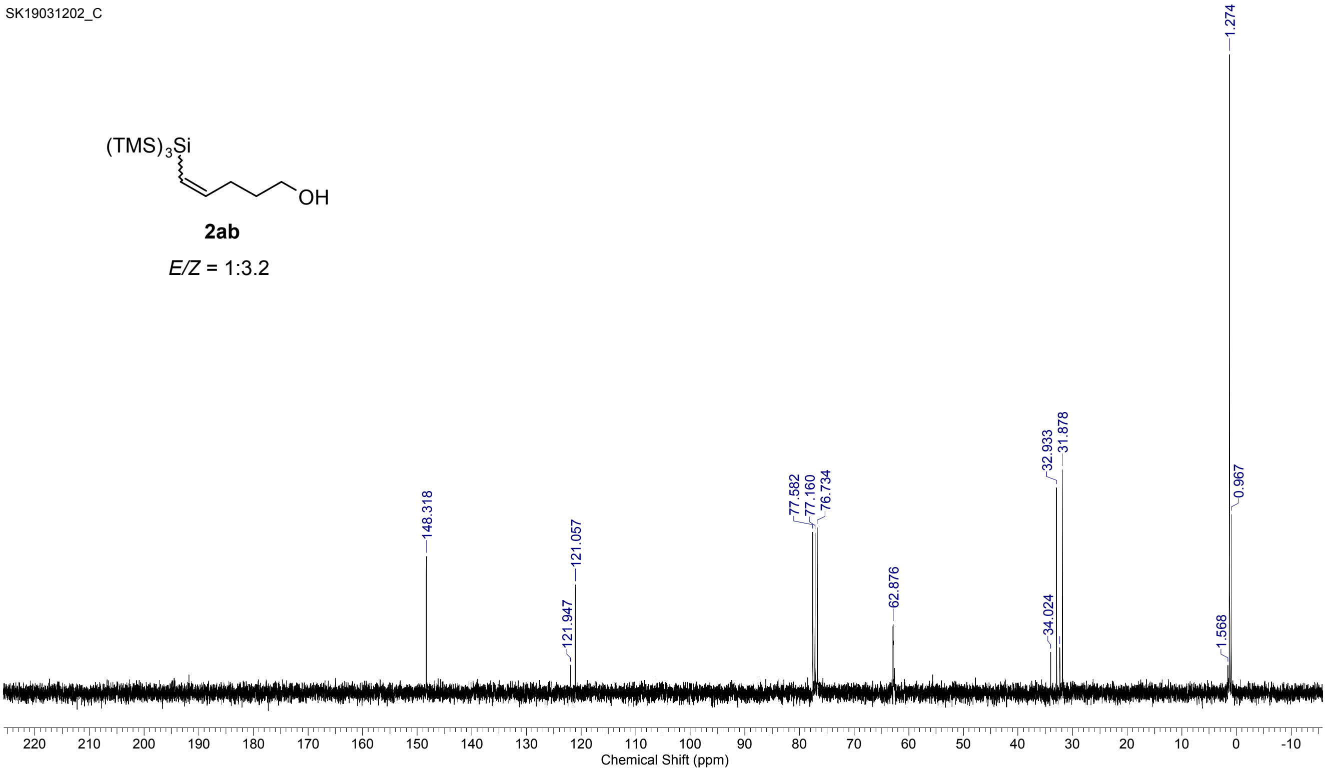

2019/03/01 11:58:58

**Acquisition Time (sec)**	2.1863	**Comment**	single_pulse	**Date**	13 Nov 2017 13:07:39		
**Date Stamp**	13 Nov 2017 13:06:48						
**File Name**	¥¥Mac¥iCloud¥[illegible]NMR[illegible]¥2017NMR_K¥2017NMR(JEOL)¥[illegible]¥TT17111301_Proton-1-1.jdf					**Frequency (MHz)**	399.78
**Nucleus**	1H	**Number of Transients**	8	**Origin**	ECA	**Original Points Count**	16384
**Owner**	delta	**Points Count**	32768	**Pulse Sequence**	proton.jxp	**Solvent**	CHLOROFORM-d
**Spectrum Offset (Hz)**	2003.4905	**Sweep Width (Hz)**	7494.00	**Temperature (degree C)**	17.900		

TT17111301_Proton-1-1.jdf

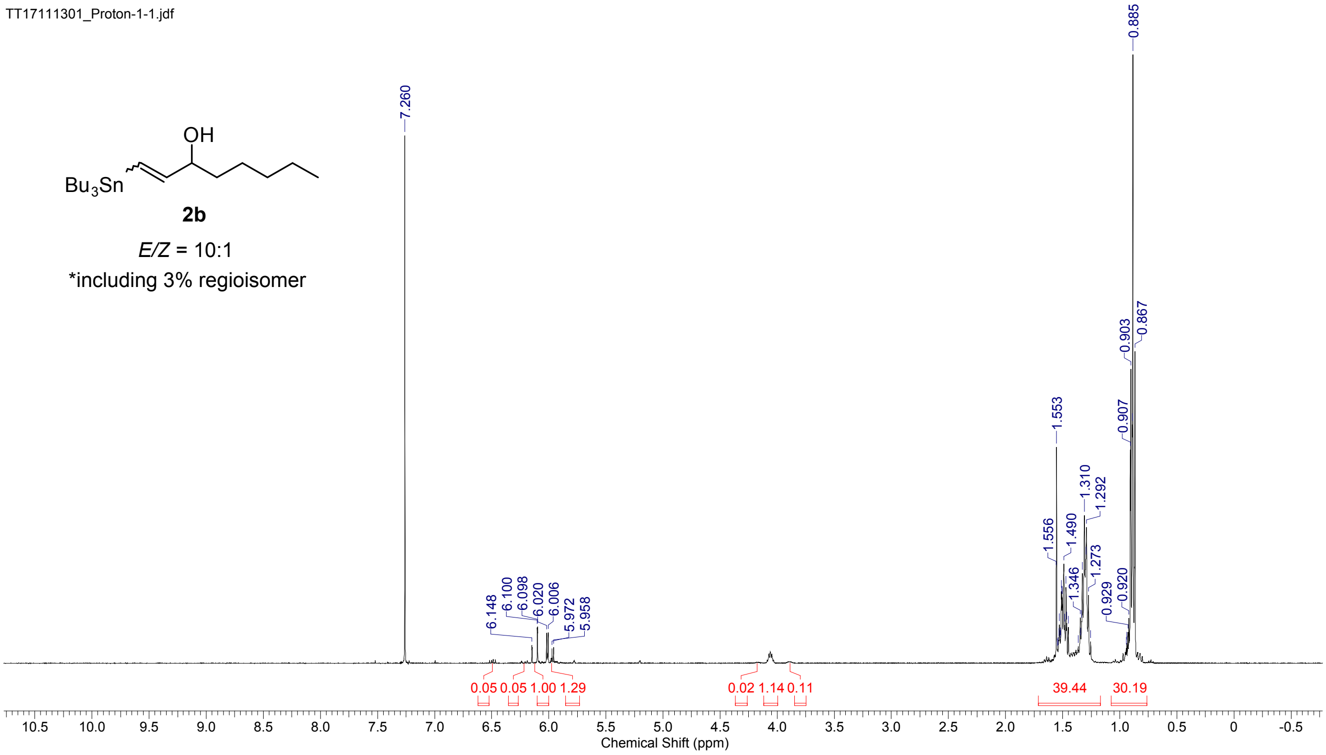

2019/03/13 11:39:36

**Acquisition Time (sec)**	0.8527	**Comment**	c13-NMR	**Date**	Mar 12 2019	**Date Stamp**	Mar 12 2019
**File Name**	¥¥Mac¥iCloud¥[illegible]NMR[illegible]¥2019NMR_K¥koba¥SK19031203_C.fid¥fid			**Frequency (MHz)**	75.46	**Nucleus**	13C
**Number of Transients**	128	**Original Points Count**	19335	**Points Count**	65536	**Pulse Sequence**	s2pul
**Receiver Gain**	30.00	**Solvent**	CHLOROFORM-d	**Spectrum Offset (Hz)**	8956.9492	**Spectrum Type**	STANDARD
**Sweep Width (Hz)**	22675.74	**Temperature (degree C)**	AMBIENT TEMPERATURE				

SK19031203_C

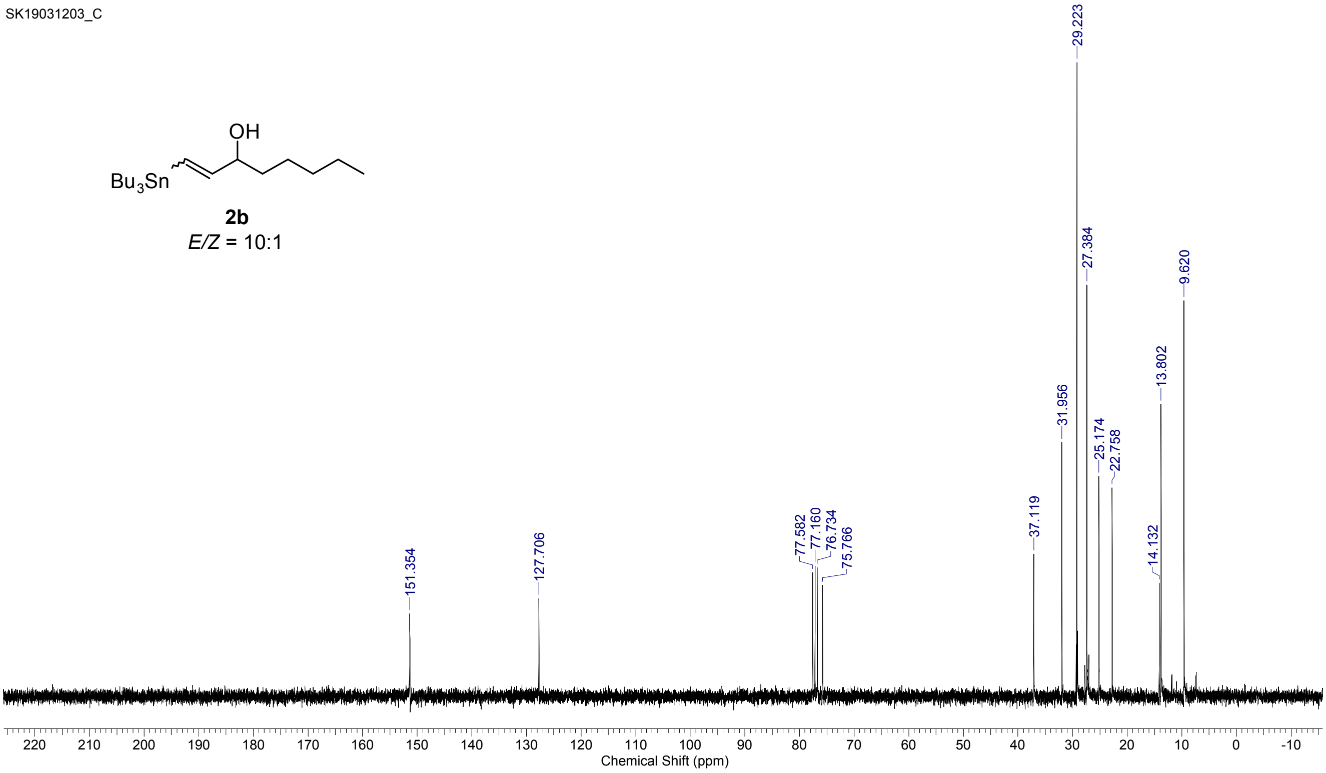

2019/03/01 14:14:08

**Acquisition Time (sec)**	2.1863	**Comment**	single_pulse	**Date**	16 Jan 2018 07:48:07		
**Date Stamp**	16 Jan 2018 07:47:16						
**File Name**	¥¥Mac¥iCloud¥[illegible]NMR[illegible]¥2017NMR_K¥2017NMR(JEOL)¥[illegible]TT18011601_Proton-1-1.jdf					**Frequency (MHz)**	399.78
**Nucleus**	1H	**Number of Transients**	8	**Origin**	ECA	**Original Points Count**	16384
**Owner**	delta	**Points Count**	32768	**Pulse Sequence**	proton.jxp	**Solvent**	CHLOROFORM-d
**Spectrum Offset (Hz)**	2003.4905	**Sweep Width (Hz)**	7494.00	**Temperature (degree C)**	16.500		

TT18011601_Proton-1-1.jdf

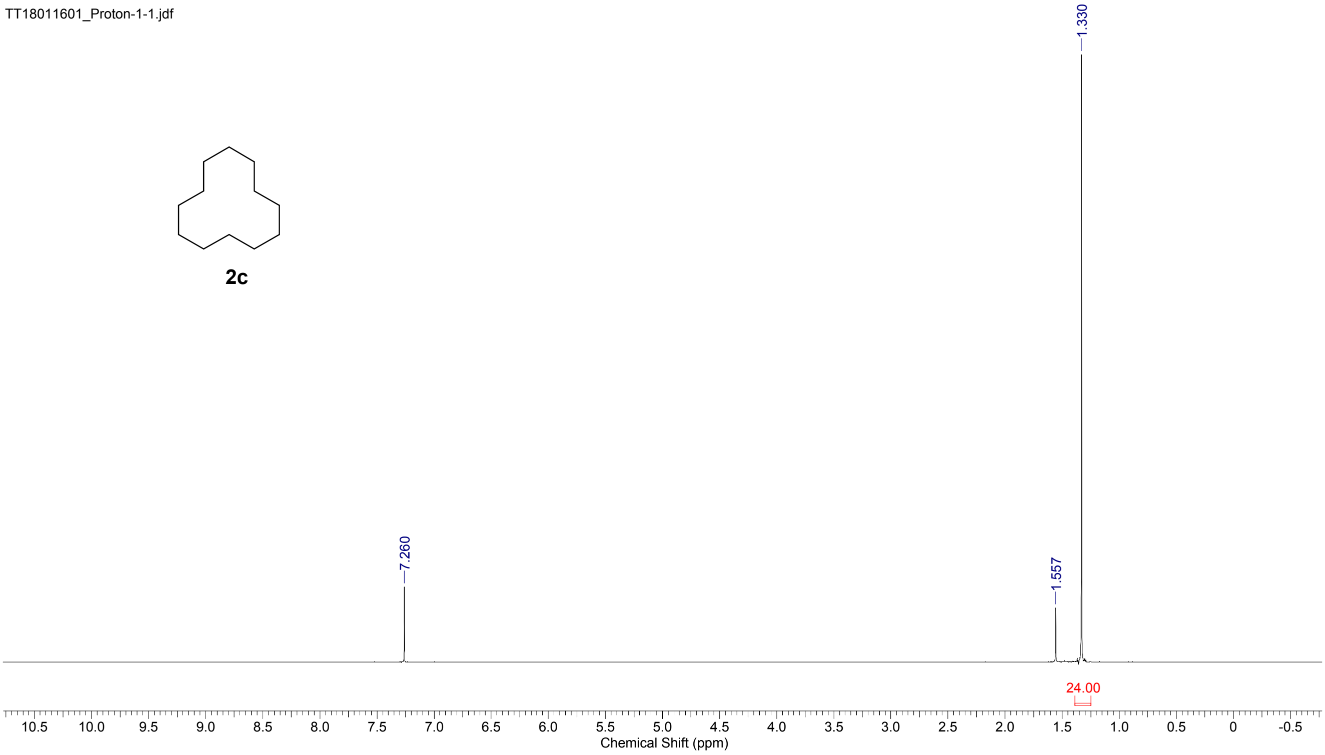

2019/05/28 19:47:53

**Acquisition Time (sec)**	0.8527	**Comment**	c13-NMR	**Date**	May 28 2019	**Date Stamp**	May 28 2019
**File Name**	¥¥Mac¥iCloud¥[illegible]NMR[illegible]¥2019NMR_K¥2019NMR(agilent)¥koba¥SK19052801_C.fid¥fid					**Frequency (MHz)**	75.46
**Nucleus**	13C	**Number of Transients**	32	**Original Points Count**	19335	**Points Count**	65536
**Pulse Sequence**	s2pul	**Receiver Gain**	30.00	**Solvent**	CHLOROFORM-d	**Spectrum Offset (Hz)**	8957.2949
**Spectrum Type**	STANDARD	**Sweep Width (Hz)**	22675.74	**Temperature (degree C)**	AMBIENT TEMPERATURE		

2c-C(SK19052801_C).esp

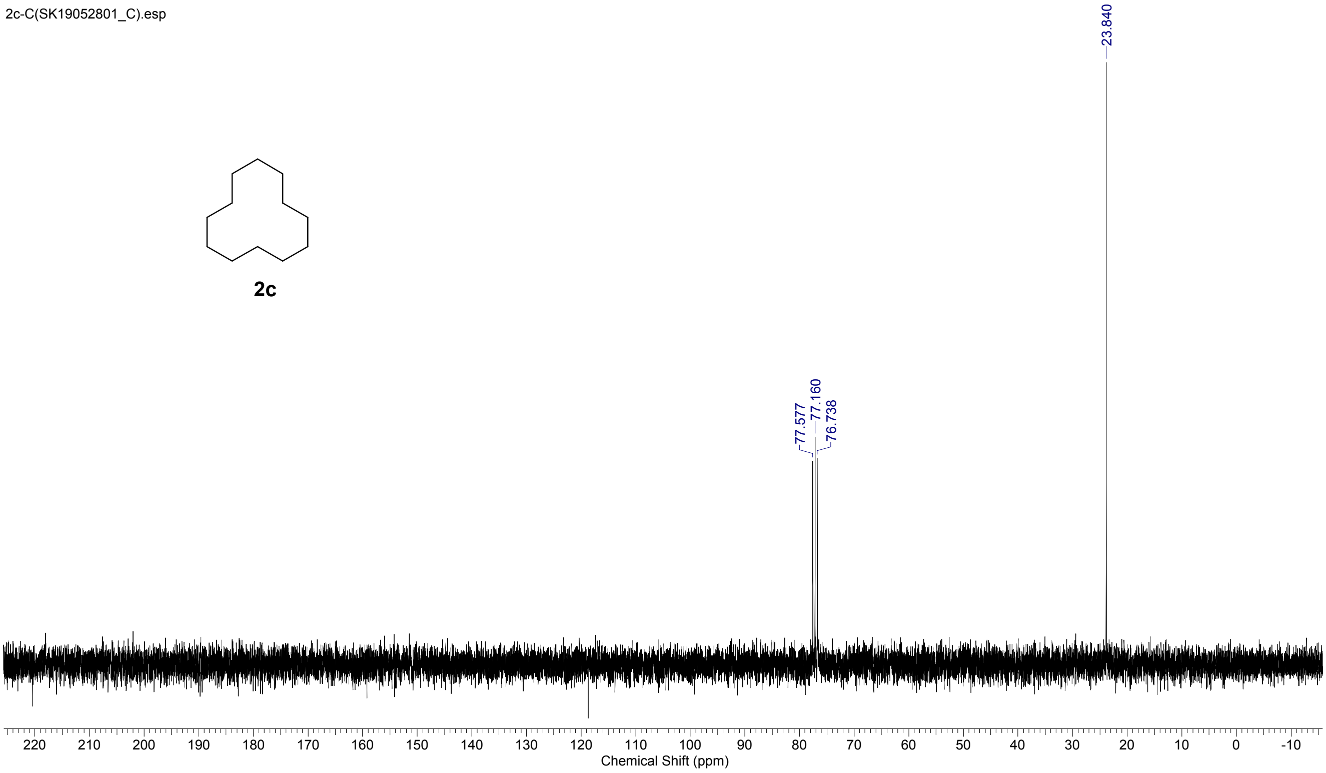

2019/03/01 14:24:18

Acquisition Time (sec)	2.1863	*Comment*	single_pulse	*Date*	17 Jan 2018 10:40:49		
Date Stamp	17 Jan 2018 10:39:58						
File Name	¥¥Mac¥iCloud¥[illegible]NMR[illegible]¥2017NMR_K¥2017NMR(JEOL)¥[illegible]TT18011701_Proton-1-1.jdf					*Frequency (MHz)*	399.78
Nucleus	1H	*Number of Transients*	8	*Origin*	ECA	*Original Points Count*	16384
Owner	delta	*Points Count*	32768	*Pulse Sequence*	proton.jxp	*Solvent*	CHLOROFORM-d
Spectrum Offset (Hz)	2003.4905	*Sweep Width (Hz)*	7494.00	*Temperature (degree C)*	20.800		

TT18011701_Proton-1-1.jdf

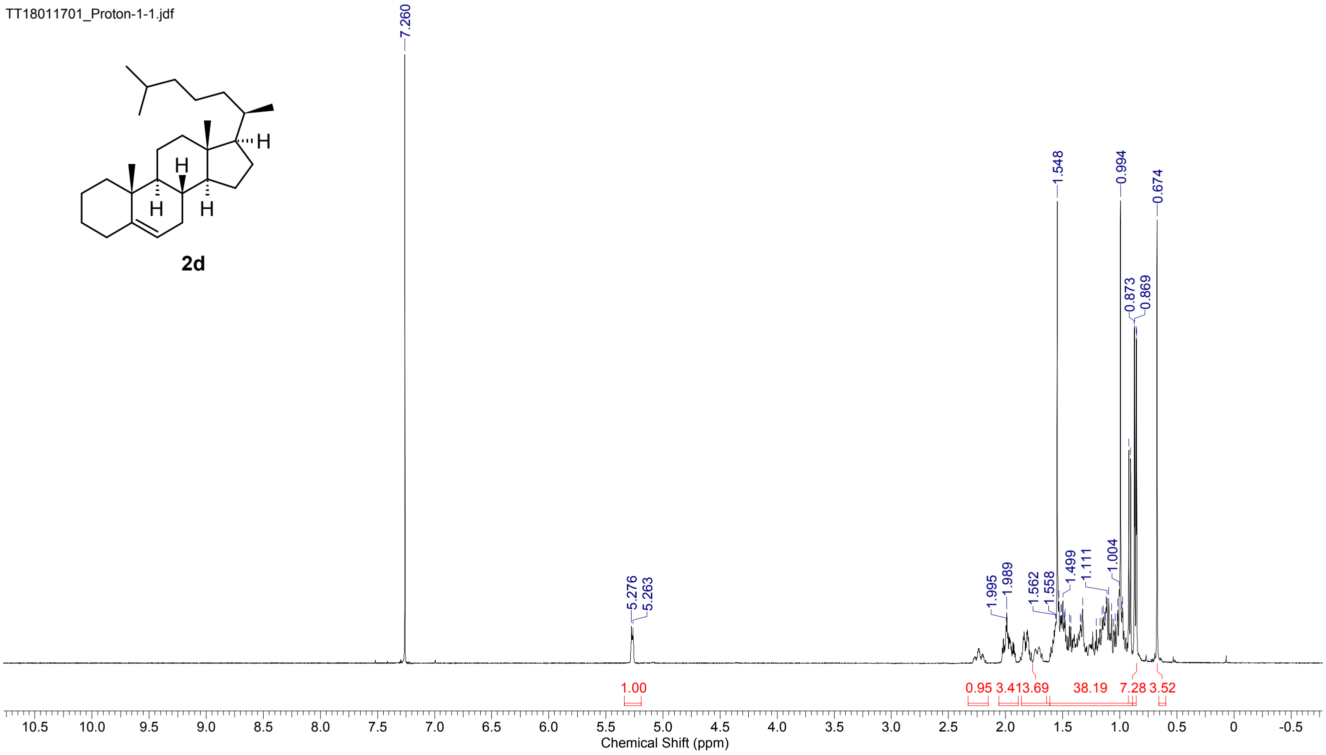

2019/03/14 17:39:06

Acquisition Time (sec)	0.8527	*Comment*	c13-NMR	*Date*	Mar 14 2019	*Date Stamp*	Mar 14 2019		
File Name	¥¥Mac¥iCloud¥[illegible]NMR[illegible]¥2019NMR_K¥koba¥SK19031402_C.fid¥fid					*Frequency (MHz)*	75.46	*Nucleus*	13C
Number of Transients	128	*Original Points Count*	19335	*Points Count*	65536	*Pulse Sequence*	s2pul	*Receiver Gain*	30.00
Solvent	CHLOROFORM-d			*Spectrum Offset (Hz)*	8957.2949	*Spectrum Type*	STANDARD	*Sweep Width (Hz)*	22675.74
Temperature (degree C)	AMBIENT TEMPERATURE								

SK19031402_C

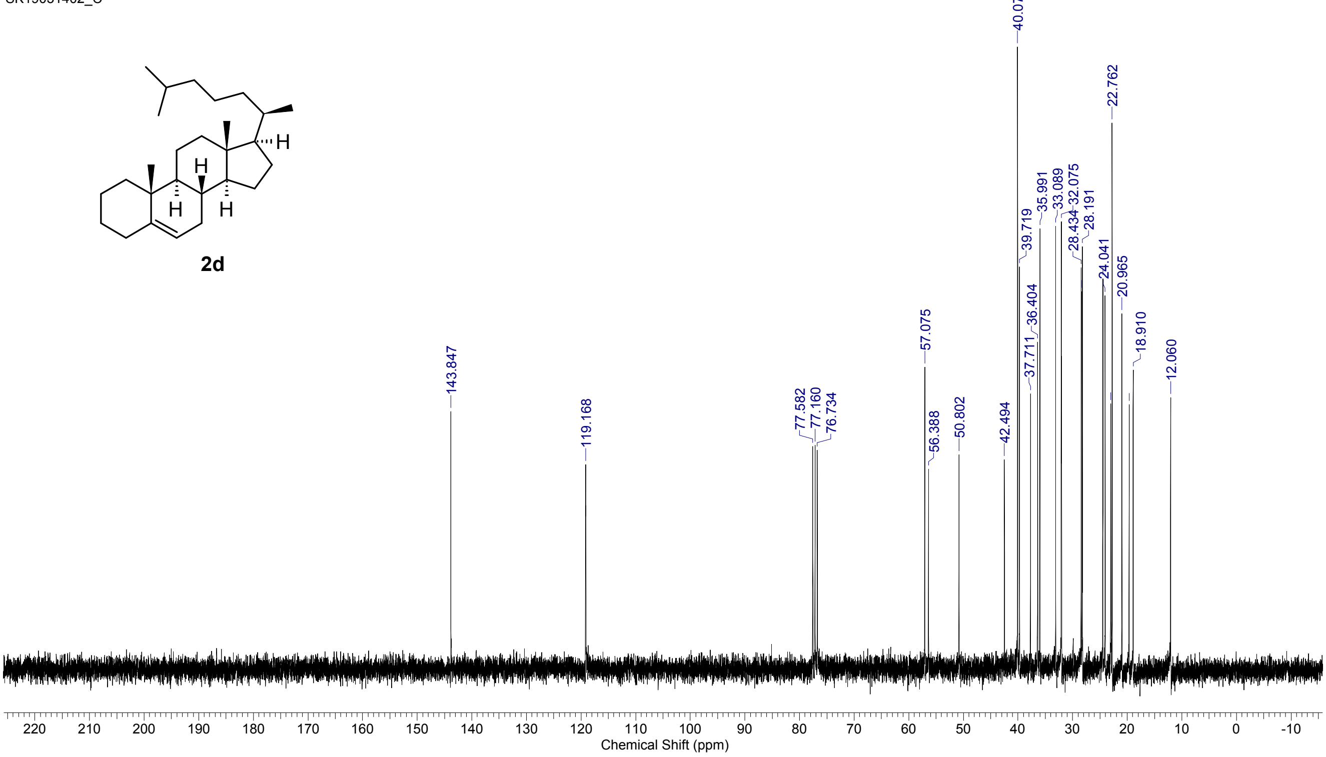

2019/06/27 16:18:55

Acquisition Time (sec)	3.5561	Comment	STANDARD 1H OBSERVE			Date	Jun 27 2019
Date Stamp	Jun 27 2019	File Name	¥¥Mac¥iCloud¥[illegible]NMR[illegible]¥2019NMR_K¥2019NMR(agilent)¥kuroda¥YK19062701.fid¥fid				
Frequency (MHz)	300.05	Nucleus	1H	Number of Transients	16	Original Points Count	17080
Points Count	65536	Pulse Sequence	s2pul	Receiver Gain	28.00	Solvent	CHLOROFORM-d
Spectrum Offset (Hz)	1698.7449	Spectrum Type	STANDARD	Sweep Width (Hz)	4803.07	Temperature (degree C)	AMBIENT TEMPERATURE

YK19062701

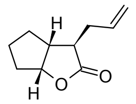

2e

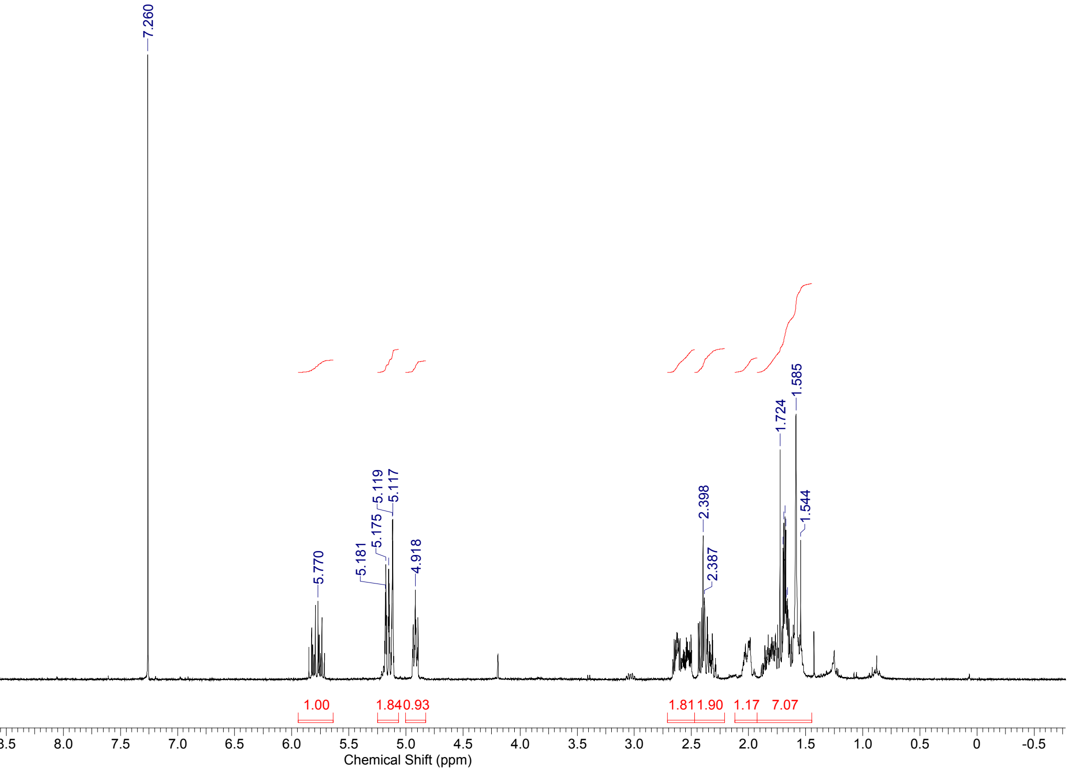

2019/06/27 16:37:17

Acquisition Time (sec)	0.8527	Comment	c13-NMR	Date	Jun 27 2019	Date Stamp	Jun 27 2019
File Name	¥¥Mac¥iCloud¥[illegible]NMR[illegible]¥2019NMR_K¥2019NMR(agilent)¥kuroda¥YK19062701_C.fid¥fid					Frequency (MHz)	75.46
Nucleus	13C	Number of Transients	1024	Original Points Count	19335	Points Count	65536
Pulse Sequence	s2pul	Receiver Gain	30.00	Solvent	CHLOROFORM-d	Spectrum Offset (Hz)	8957.2959
Spectrum Type	STANDARD	Sweep Width (Hz)	22675.74	Temperature (degree C)	AMBIENT TEMPERATURE		

YK19062701_C

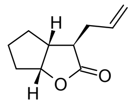

2e

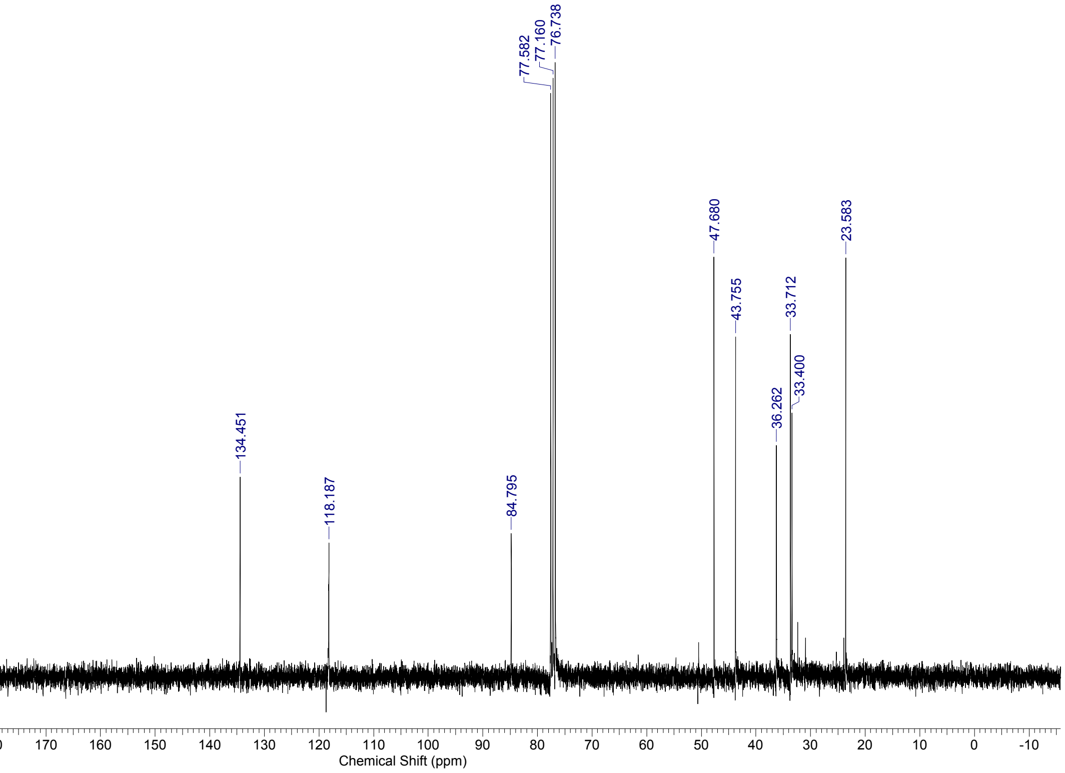

2019/05/28 14:23:29

Acquisition Time (sec)	3.5561	***Comment***	STANDARD 1H OBSERVE			***Date***	May 25 2019
Date Stamp	May 25 2019	***File Name***	¥¥Mac¥iCloud¥[illegible]NMR[illegible]¥2019NMR_K¥2019NMR(agilent)¥koba¥SK19052506.fid¥fid				
Frequency (MHz)	300.05	***Nucleus***	1H	***Number of Transients***	16	***Original Points Count***	17080
Points Count	65536	***Pulse Sequence***	s2pul	***Receiver Gain***	30.00	***Solvent***	CHLOROFORM-d
Spectrum Offset (Hz)	1698.7450	***Spectrum Type***	STANDARD	***Sweep Width (Hz)***	4803.07	***Temperature (degree C)***	AMBIENT TEMPERATURE

SK19052506

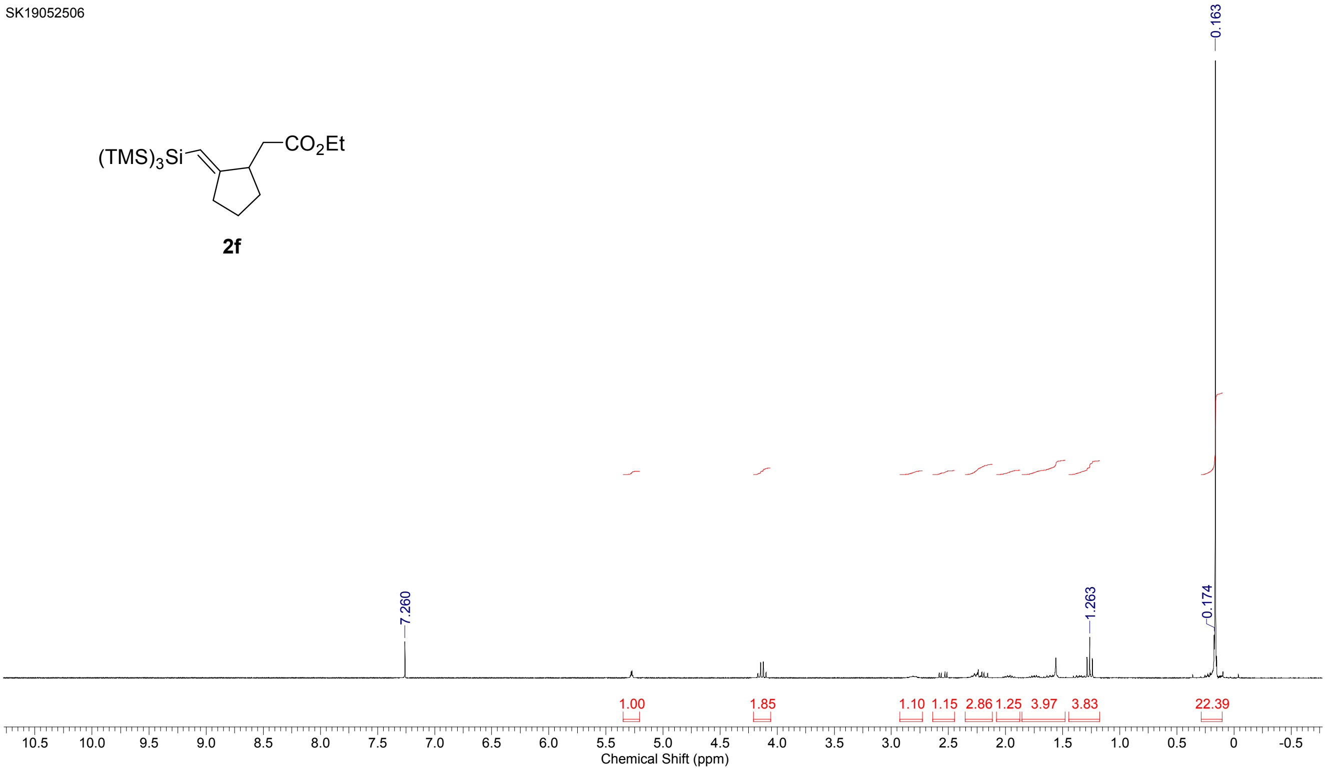

2019/05/28 14:42:54

Acquisition Time (sec)	1.0381	***Comment***	single pulse decoupled gated NOE			***Date***	26 Apr 2019 13:16:47
Date Stamp	26 Apr 2019 12:37:16						
File Name	¥¥Mac¥iCloud¥[illegible]NMR[illegible]¥2019NMR_K¥2019NMR(JEOL)¥[illegible]NMR2019¥TT19042601_Carbon-1-1.jdf						
Frequency (MHz)	100.53	***Nucleus***	13C	***Number of Transients***	1024	***Origin***	ECA
Original Points Count	32768	***Owner***	delta	***Points Count***	65536	***Pulse Sequence***	carbon.jxp
Solvent	CHLOROFORM-d	***Spectrum Offset (Hz)***	10058.1504	***Sweep Width (Hz)***	31565.66	***Temperature (degree C)***	18.600

2f-C(TT19042601_Carbon-1-1).esp

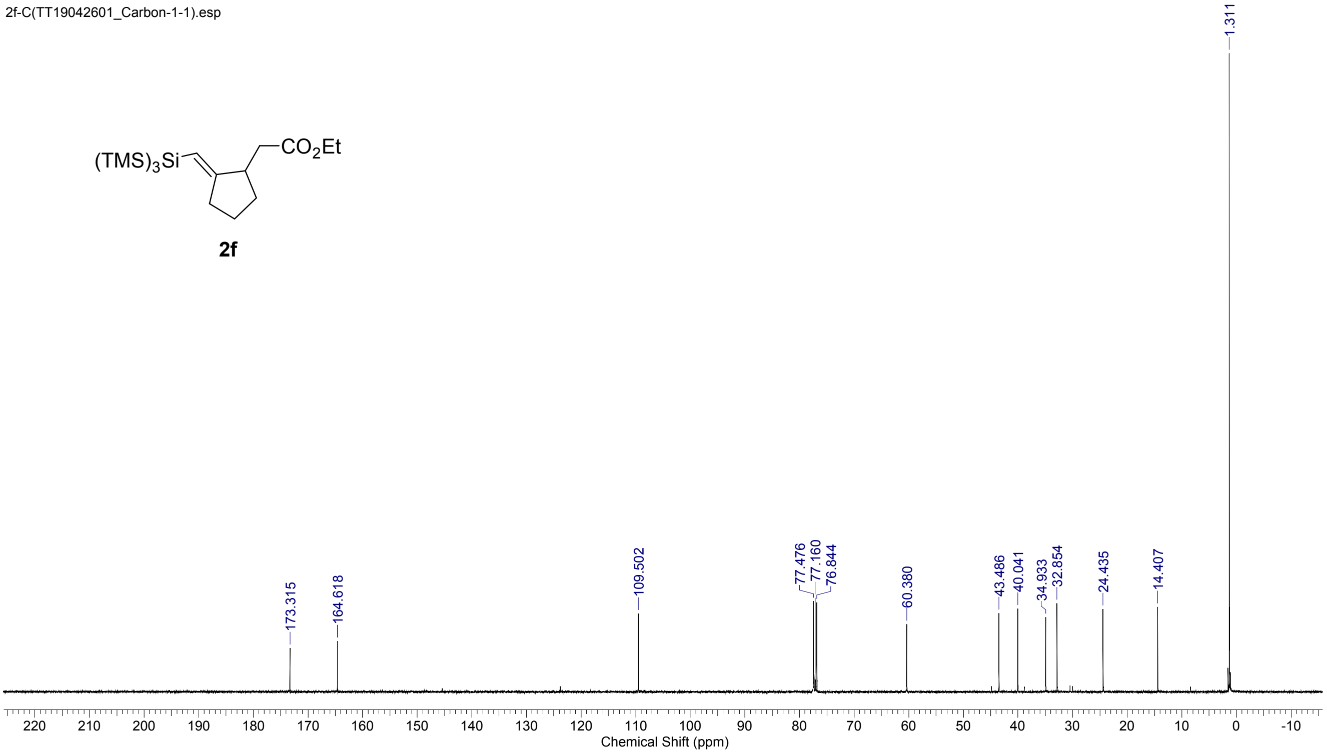

2019/06/25 12:36:12

Acquisition Time (sec)	2.1863	*Comment*	single pulse	*Date*	10 Jun 2019 13:42:09			*Date Stamp*	10 Jun 2019 13:41:18
File Name	¥¥WS¥data¥[illegible]TT19061003_Proton-1-1.jdf			*Frequency (MHz)*	399.78	*Nucleus*	1H	*Number of Transients*	8
Origin	ECA	*Original Points Count*	16384	*Owner*	delta	*Points Count*	32768	*Pulse Sequence*	proton.jxp
Solvent	CHLOROFORM-d			*Spectrum Offset (Hz)*	2003.9476	*Sweep Width (Hz)*	7494.00	*Temperature (degree C)*	19.800

TT19061003_Proton-1-1.esp

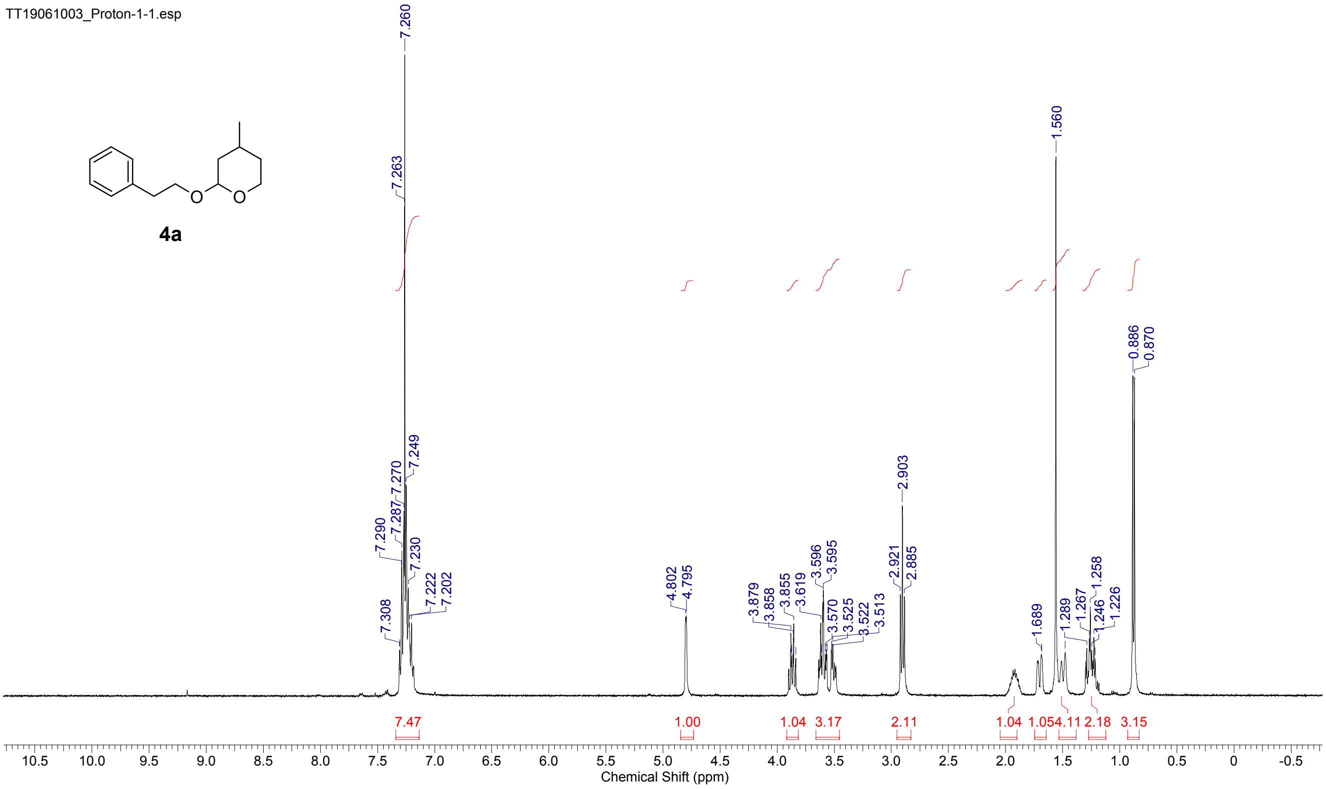

2019/06/25 12:38:25

Acquisition Time (sec)	1.0381	*Comment*	single pulse decoupled gated NOE			*Date*	14 Jun 2019 08:52:38		
Date Stamp	14 Jun 2019 08:13:06			*File Name*	¥¥WS¥data¥[illegible]TT19061003_Carbon-1-1.jdf			*Frequency (MHz)*	100.53
Nucleus	13C	*Number of Transients*	1024	*Origin*	ECA	*Original Points Count*	32768	*Owner*	delta
Points Count	65536	*Pulse Sequence*	carbon.jxp	*Solvent*	CHLOROFORM-d			*Spectrum Offset (Hz)*	10058.1504
Sweep Width (Hz)	31565.66	*Temperature (degree C)*	21.100						

TT19061003_Carbon-1-1.esp

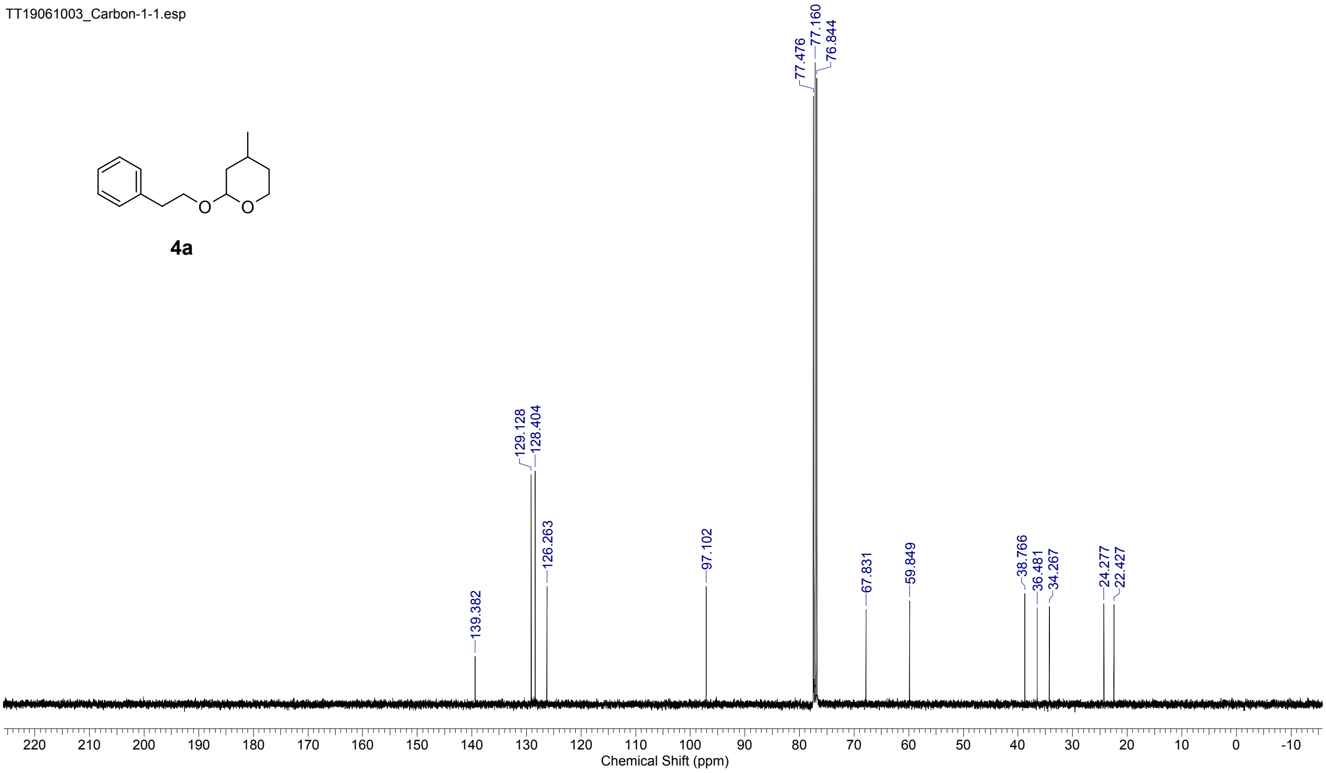

2019/06/25 12:40:24

Acquisition Time (sec)	2.1863	*Comment*	single pulse	*Date*	10 Jun 2019 13:30:16			*Date Stamp*	10 Jun 2019 13:29:24
File Name	¥¥WS¥data¥[illegible]TT19061001_Proton-2-1.jdf			*Frequency (MHz)*	399.78	*Nucleus*	1H	*Number of Transients*	8
Origin	ECA	*Original Points Count*	16384	*Owner*	delta	*Points Count*	32768	*Pulse Sequence*	proton.jxp
Solvent	CHLOROFORM-d			*Spectrum Offset (Hz)*	2003.2618	*Sweep Width (Hz)*	7494.00	*Temperature (degree C)*	20.000

TT19061001_Proton-2-1.esp

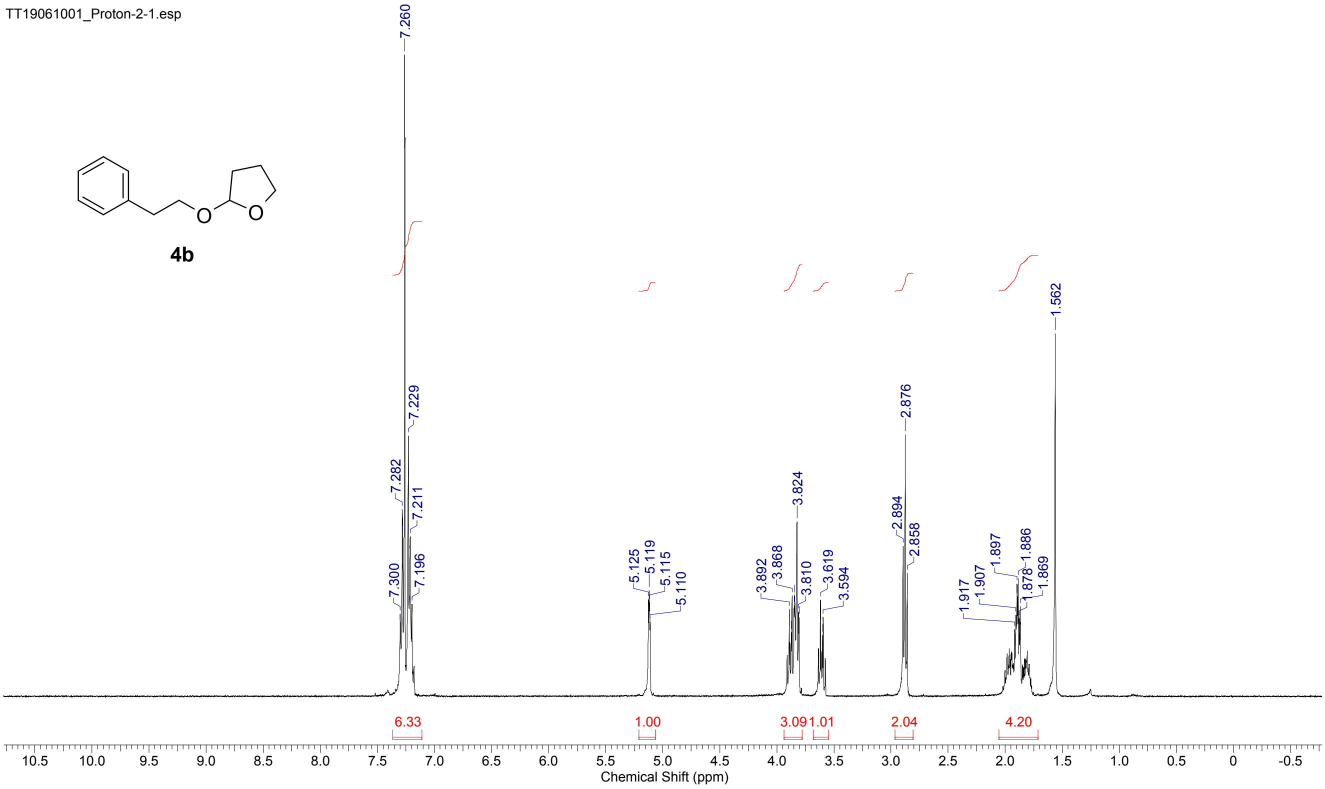

2019/06/25 12:41:24

Acquisition Time (sec)	1.0381	*Comment*	single pulse decoupled gated NOE			*Date*	14 Jun 2019 13:24:13		
Date Stamp	14 Jun 2019 12:44:42			*File Name*	¥¥WS¥data¥[illegible]TT19061001_Carbon-1-1.jdf			*Frequency (MHz)*	100.53
Nucleus	13C	*Number of Transients*	1024	*Origin*	ECA	*Original Points Count*	32768	*Owner*	delta
Points Count	65536	*Pulse Sequence*	carbon.jxp	*Solvent*	CHLOROFORM-d			*Spectrum Offset (Hz)*	10056.7051
Sweep Width (Hz)	31565.66	*Temperature (degree C)*	20.400						

TT19061001_Carbon-1-1.esp

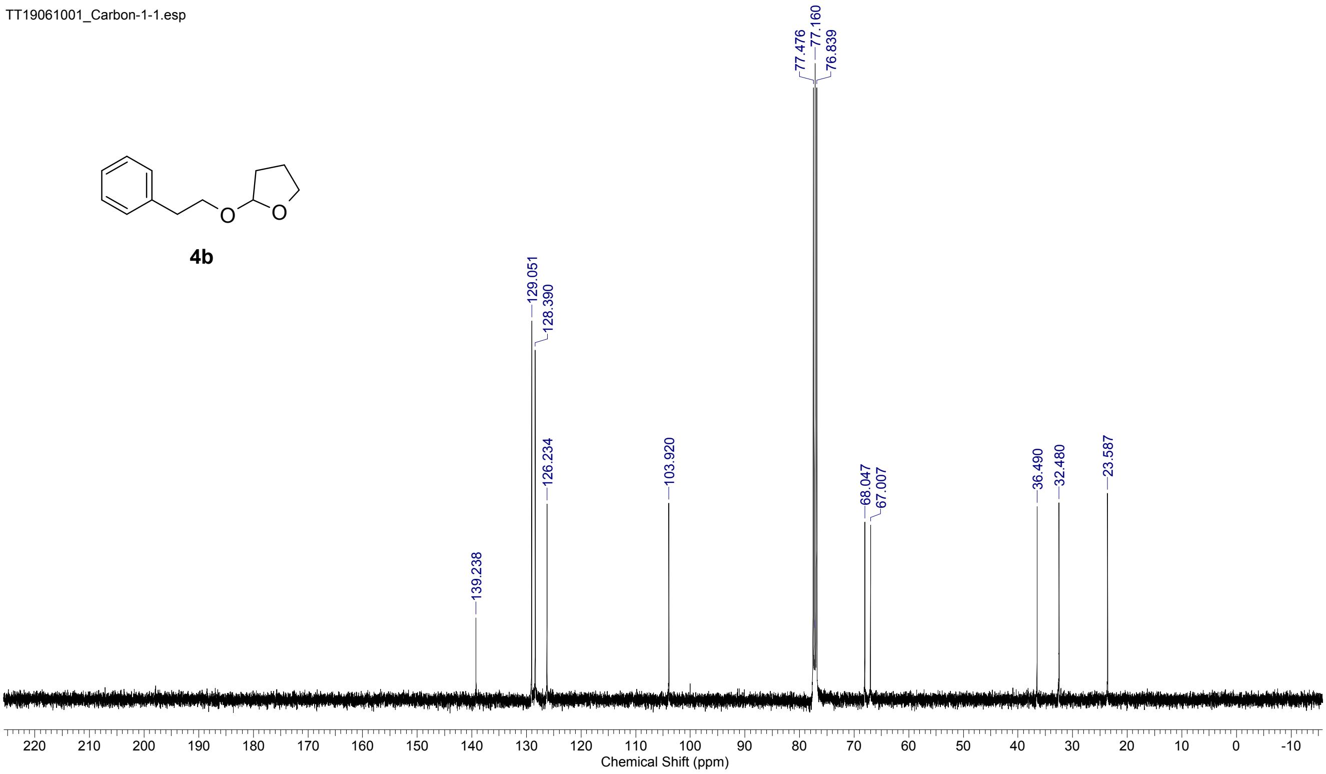

2019/06/25 12:44:12

Parameter	Value
Acquisition Time (sec)	2.1863
Comment	single_pulse
Date	15 Jun 2019 11:56:09
Date Stamp	15 Jun 2019 11:55:18
File Name	¥¥WS¥data¥[illegible]TT19061507_Proton-1-1.jdf
Frequency (MHz)	399.78
Nucleus	1H
Number of Transients	8
Origin	ECA
Original Points Count	16384
Owner	delta
Points Count	32768
Pulse Sequence	proton.jxp
Solvent	CHLOROFORM-d
Spectrum Offset (Hz)	2003.4905
Sweep Width (Hz)	7494.00
Temperature (degree C)	20.500

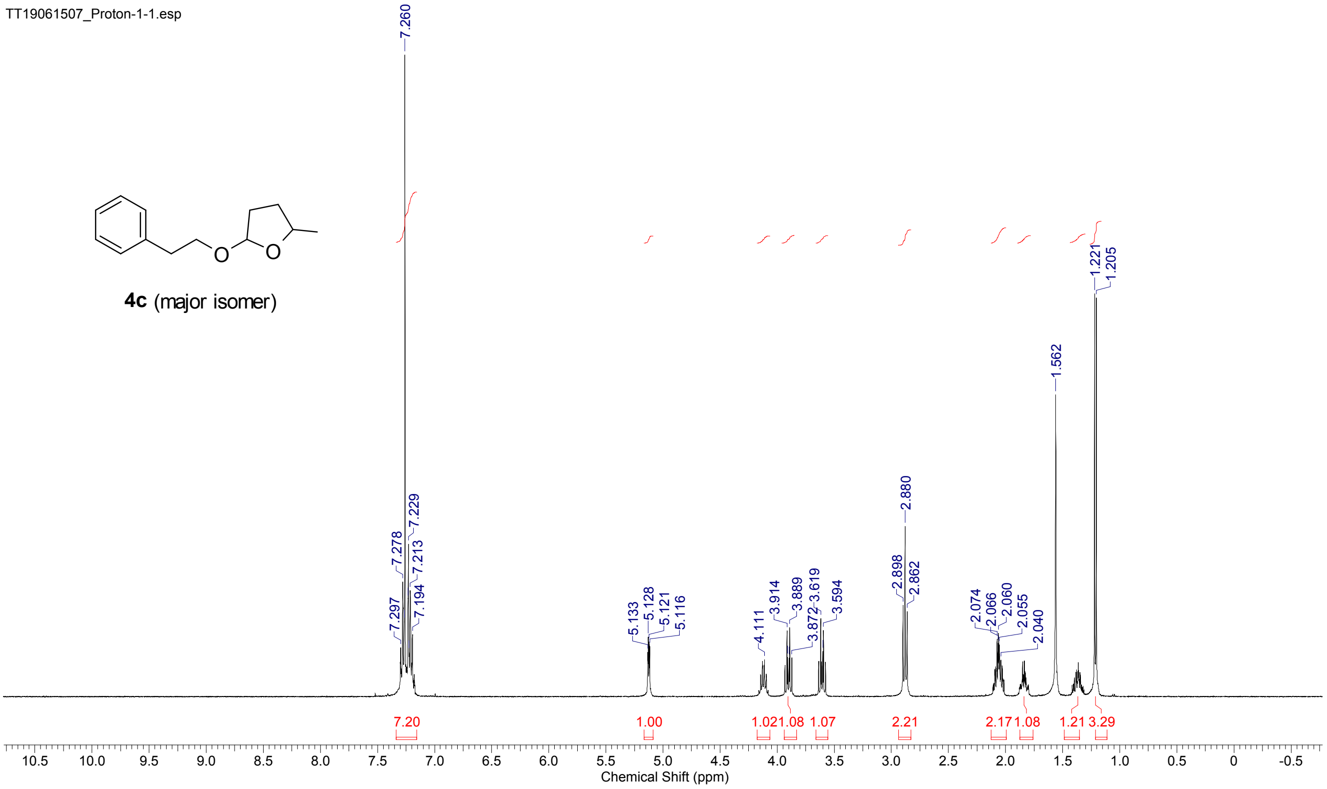

2019/06/25 12:46:31

Parameter	Value
Acquisition Time (sec)	1.0381
Comment	single pulse decoupled gated NOE
Date	17 Jun 2019 08:12:18
Date Stamp	17 Jun 2019 07:32:47
File Name	¥¥WS¥data¥[illegible]TT19061507_Carbon-1-1.jdf
Frequency (MHz)	100.53
Nucleus	13C
Number of Transients	1024
Origin	ECA
Original Points Count	32768
Owner	delta
Points Count	65536
Pulse Sequence	carbon.jxp
Solvent	CHLOROFORM-d
Spectrum Offset (Hz)	10056.7051
Sweep Width (Hz)	31565.66
Temperature (degree C)	18.700

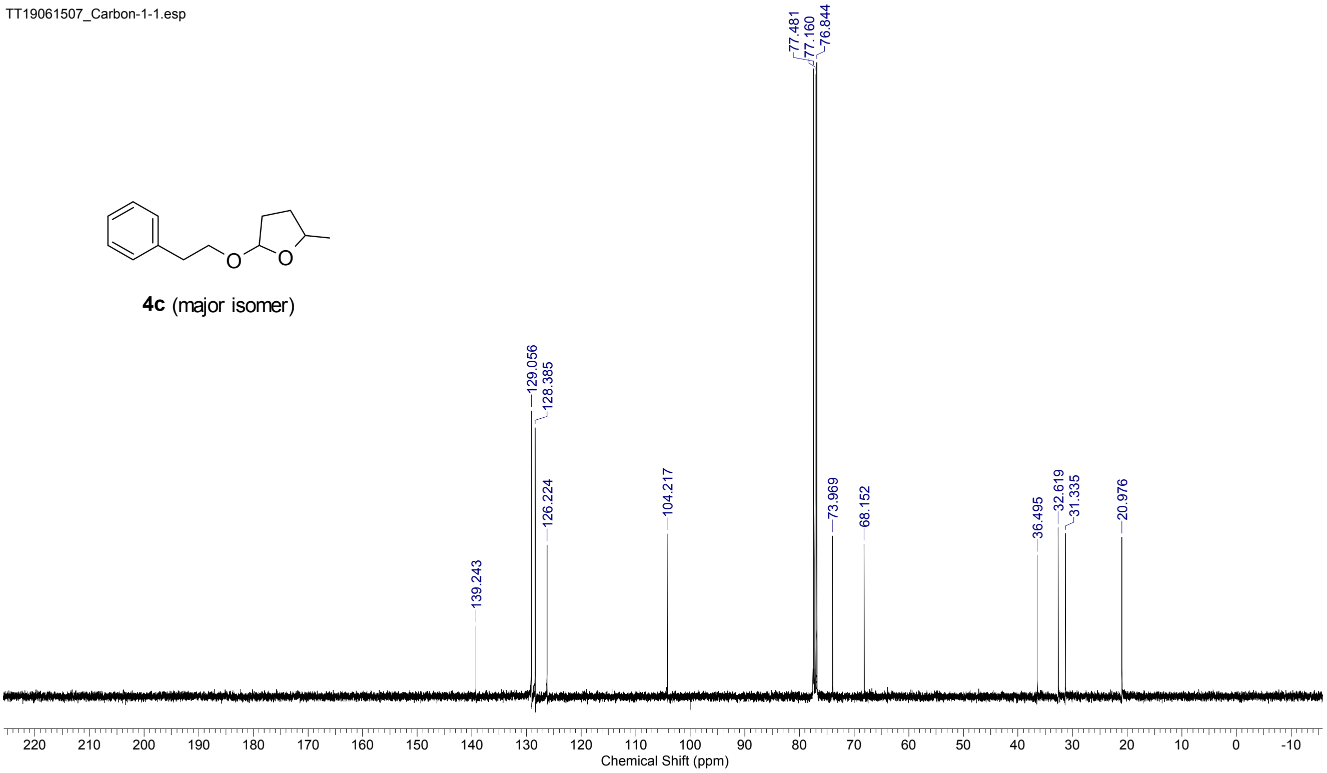

2019/06/25 15:11:46

Acquisition Time (sec)	2.1863	Comment	single pulse	Date	17 Jun 2019 13:07:34	Date Stamp	17 Jun 2019 13:06:43
File Name	¥¥WS¥data¥[illegible]TT19061701_Proton-2-1.jdf			Frequency (MHz)	399.78	Nucleus	1H
Number of Transients	8	Origin	ECA	Original Points Count	16384	Owner	delta
Points Count	32768	Pulse Sequence	proton.jxp	Solvent	CHLOROFORM-d	Spectrum Offset (Hz)	2003.7192
Sweep Width (Hz)	7494.00	Temperature (degree C)	20.400				

4c-Hminor_TT19061701_Proton-2-1.esp

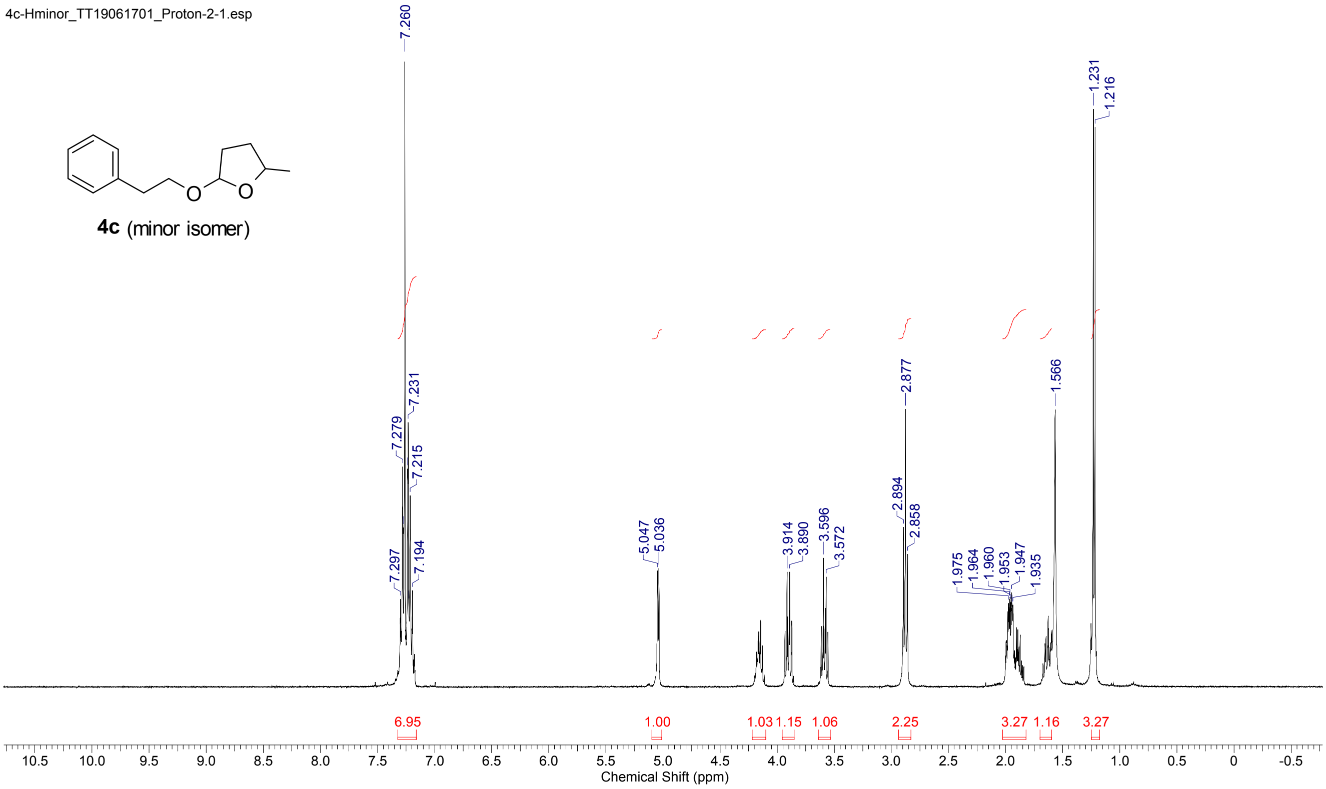

2019/06/25 12:53:13

Acquisition Time (sec)	1.0381	Comment	single pulse decoupled gated NOE	Date	17 Jun 2019 11:17:36		
Date Stamp	17 Jun 2019 11:01:27	File Name	¥¥WS¥data¥[illegible]TT19061701_Carbon-1-1.jdf			Frequency (MHz)	100.53
Nucleus	13C	Number of Transients	756	Origin	ECA	Original Points Count	32768
Owner	delta	Points Count	65536	Pulse Sequence	carbon.jxp	Solvent	CHLOROFORM-d
Spectrum Offset (Hz)	10057.6689	Sweep Width (Hz)	31565.66	Temperature (degree C)	19.600		

TT19061701_Carbon-1-1.esp

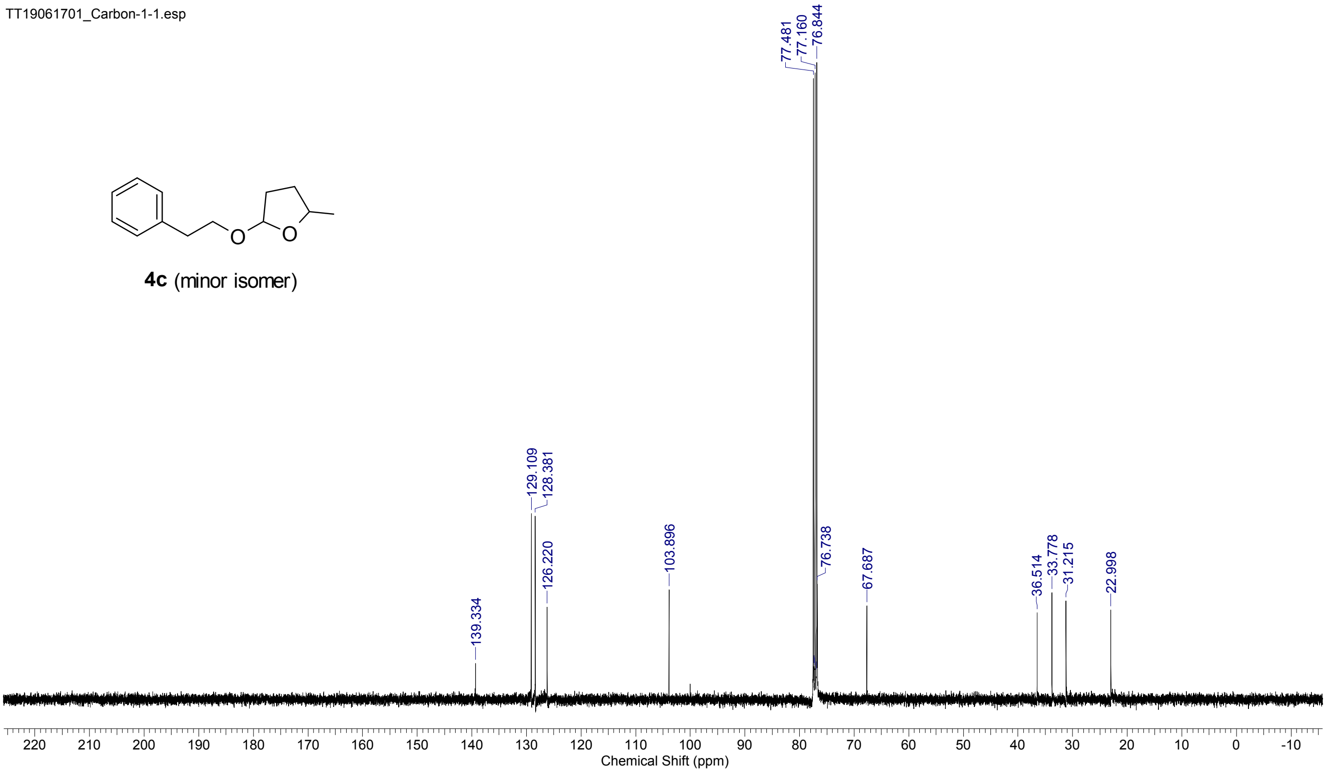

2019/03/02 14:09:19

Acquisition Time (sec)	2.1863	*Comment*	single pulse	*Date*	23 Feb 2017 13:05:05		
Date Stamp	23 Feb 2017 13:04:14						
File Name	¥¥Mac¥iCloud¥[illegible]NMR[illegible]¥2016NMR_K¥2016NMR(JEOL)¥[illegible]¥SY17022301_Proton-2-1.jdf					*Frequency (MHz)*	399.78
Nucleus	1H	*Number of Transients*	8	*Origin*	ECA	*Original Points Count*	16384
Owner	delta	*Points Count*	32768	*Pulse Sequence*	proton.jxp	*Solvent*	CHLOROFORM-d
Spectrum Offset (Hz)	2003.2618	*Sweep Width (Hz)*	7494.00	*Temperature (degree C)*	21.300		

SY17022301_Proton-2-1.jdf

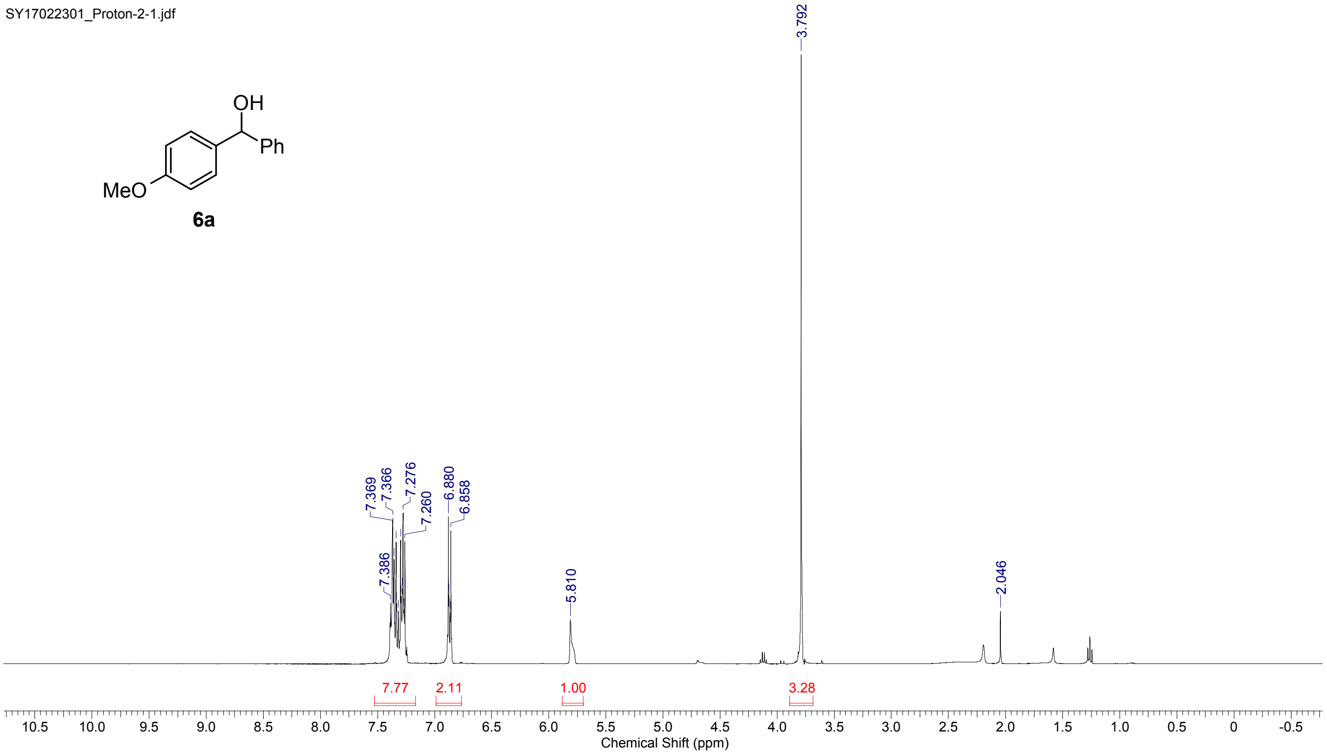

2019/03/02 14:15:45

Acquisition Time (sec)	1.0381	*Comment*	single pulse decoupled gated NOE	*Date*	23 Feb 2017 13:17:03
Date Stamp	23 Feb 2017 13:07:01				
File Name	¥¥Mac¥iCloud¥[illegible]NMR[illegible]¥2016NMR_K¥2016NMR(JEOL)¥[illegible]¥SY17022301_Carbon-1-1.jdf			*Frequency (MHz)*	100.53
Nucleus	13C	*Number of Transients*	256	*Origin*	ECA
Original Points Count	32768				
Owner	delta	*Points Count*	65536	*Pulse Sequence*	carbon.jxp
Solvent	CHLOROFORM-d				
Spectrum Offset (Hz)	10056.7051	*Sweep Width (Hz)*	31565.66	*Temperature (degree C)*	21.200

SY17022301_Carbon-1-1.jdf

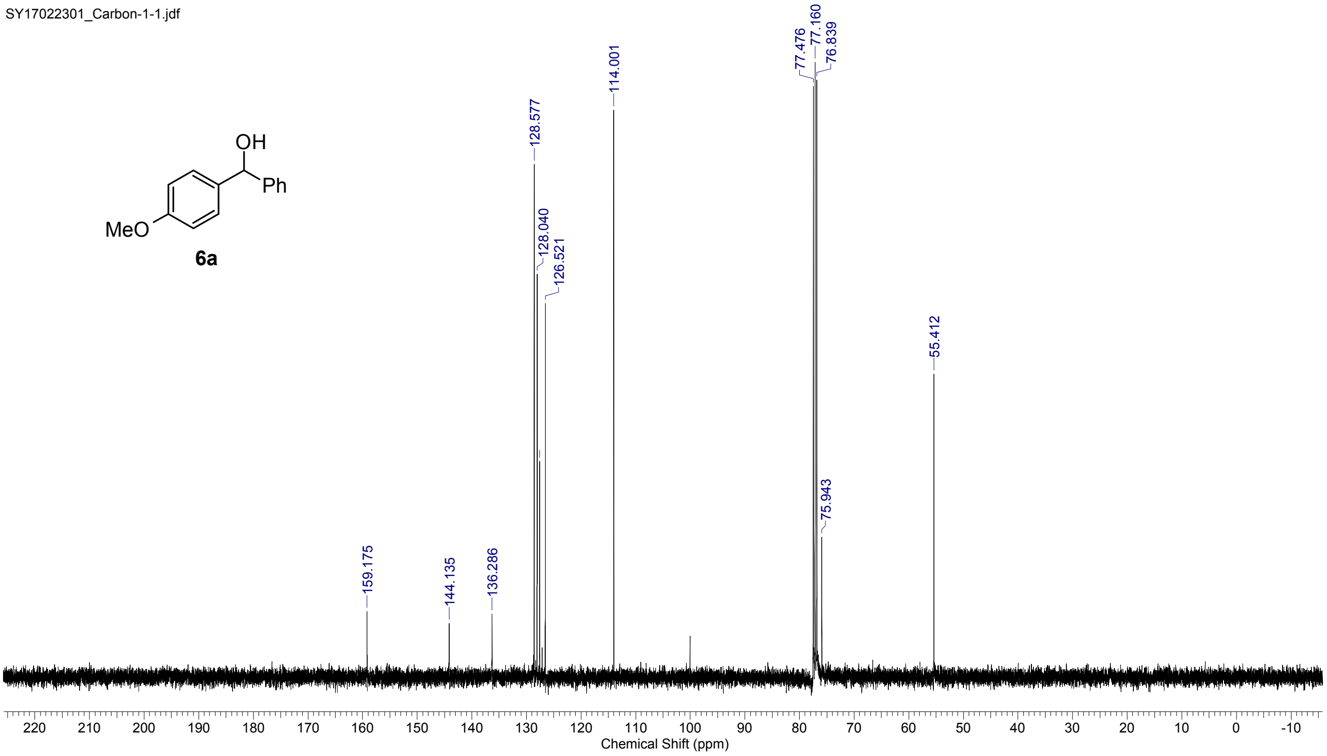

2019/03/02 14:46:39

Acquisition Time (sec)	2.1863	***Comment***	single_pulse	***Date***	05 Jun 2017 12:10:22		
Date Stamp	05 Jun 2017 12:09:31						
File Name	¥¥Mac¥iCloud¥[illegible]NMR[illegible]¥2017NMR_K¥2017NMR(JEOL)¥[illegible]¥TT17060503_Proton-1-1.jdf					***Frequency (MHz)***	399.78
Nucleus	1H	***Number of Transients***	8	***Origin***	ECA	***Original Points Count***	16384
Owner	delta	***Points Count***	32768	***Pulse Sequence***	proton.jxp	***Solvent***	CHLOROFORM-d
Spectrum Offset (Hz)	2003.4905	***Sweep Width (Hz)***	7494.00	***Temperature (degree C)***	20.500		

TT17060503_Proton-1-1.jdf

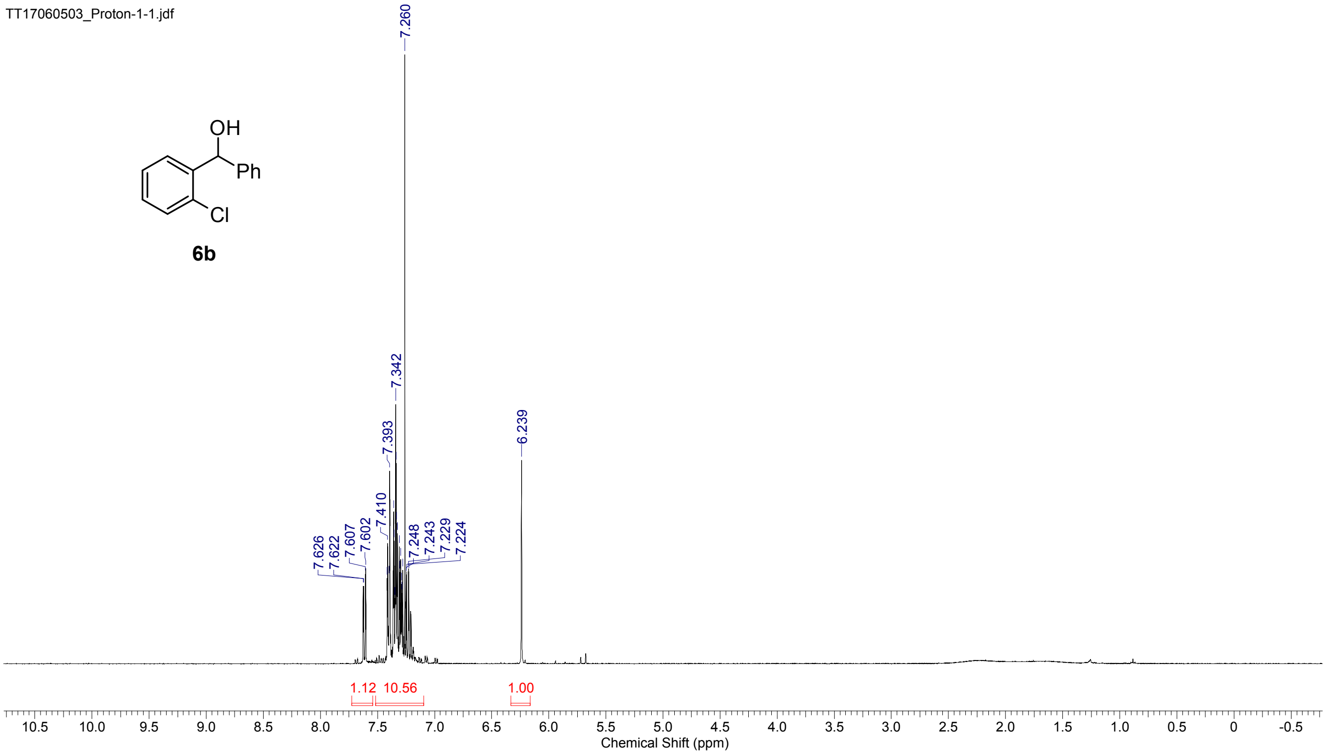

2019/03/02 14:53:35

Acquisition Time (sec)	1.0381	***Comment***	single pulse decoupled gated NOE			***Date***	22 Feb 2017 14:27:47
Date Stamp	22 Feb 2017 14:22:40						
File Name	¥¥Mac¥iCloud¥[illegible]NMR[illegible]¥2016NMR_K¥2016NMR(JEOL)¥[illegible]¥SY17022209_Carbon-1-1.jdf					***Frequency (MHz)***	100.53
Nucleus	13C	***Number of Transients***	128	***Origin***	ECA	***Original Points Count***	32768
Owner	delta	***Points Count***	65536	***Pulse Sequence***	carbon.jxp	***Solvent***	CHLOROFORM-d
Spectrum Offset (Hz)	10049.9629	***Sweep Width (Hz)***	31565.66	***Temperature (degree C)***	19.400		

SY17022209_Carbon-1-1.jdf

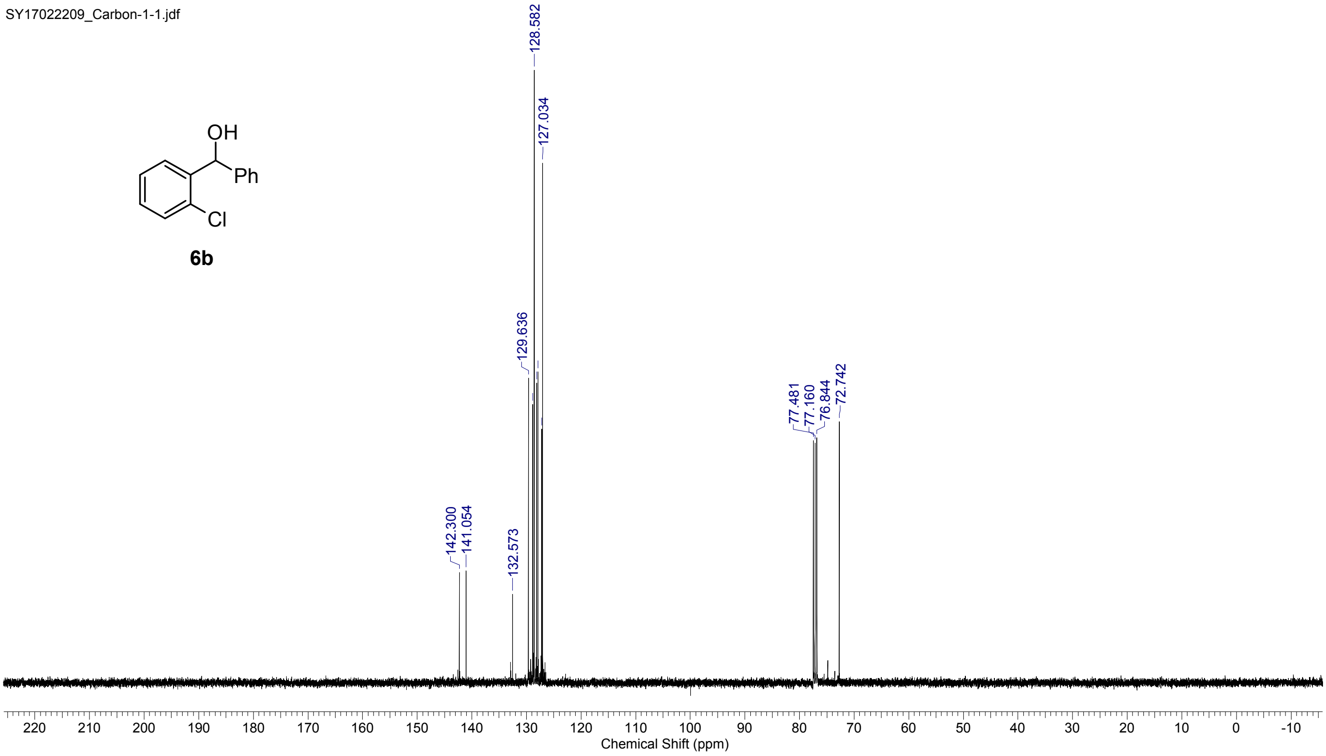

2019/06/03 10:19:52

Acquisition Time (sec)	3.5561	*Comment*	STANDARD 1H OBSERVE			*Date*	Jun 1 2019
Date Stamp	Jun 1 2019	*File Name*	¥¥Mac¥iCloud¥[illegible]¥NMR[illegible]¥2019NMR_K¥2019NMR(agilent)¥koba¥SK19060104.fid¥fid				
Frequency (MHz)	300.05	*Nucleus*	1H	*Number of Transients*	16	*Original Points Count*	17080
Points Count	65536	*Pulse Sequence*	s2pul	*Receiver Gain*	24.00	*Solvent*	CHLOROFORM-d
Spectrum Offset (Hz)	1698.8915	*Spectrum Type*	STANDARD	*Sweep Width (Hz)*	4803.07	*Temperature (degree C)*	AMBIENT TEMPERATURE

SK19060104

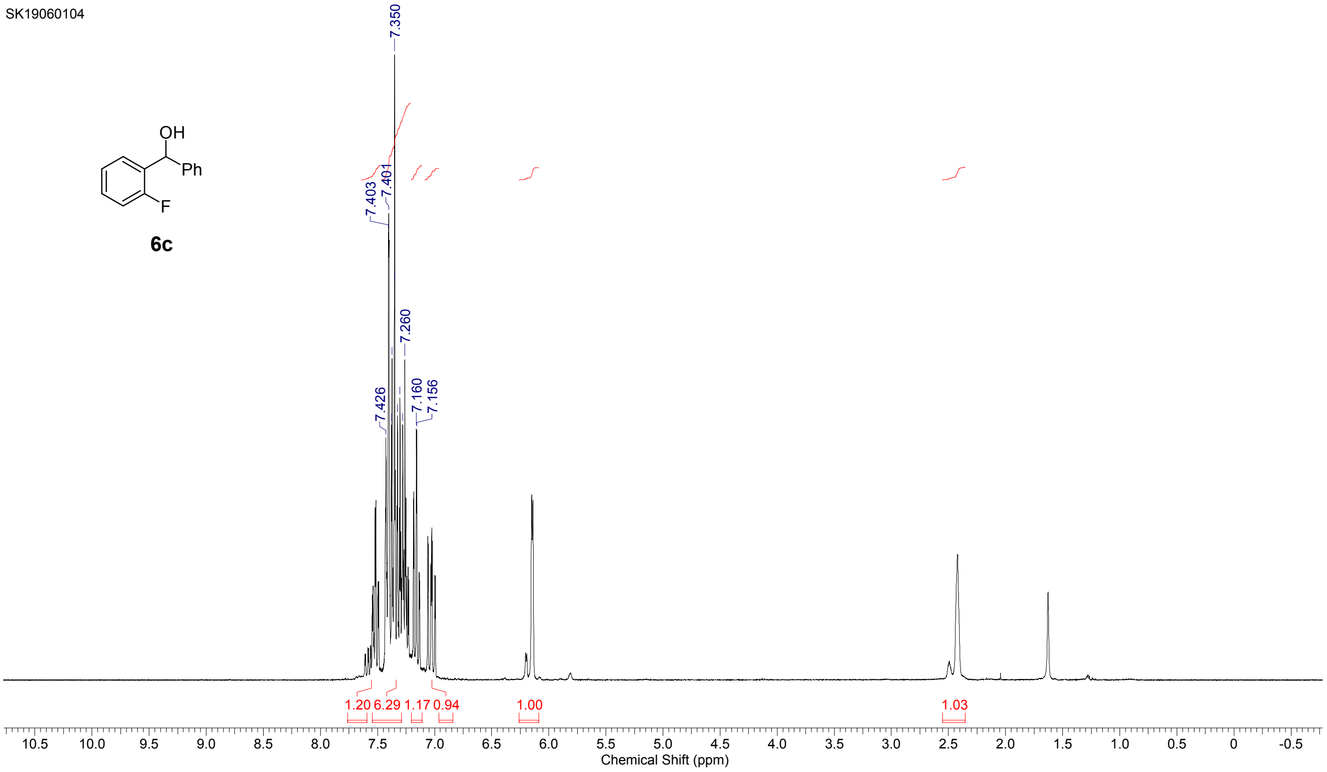

2019/06/03 13:14:37

Acquisition Time (sec)	0.8527	*Comment*	c13-NMR	*Date*	Jun 1 2019	*Date Stamp*	Jun 1 2019
File Name	¥¥Mac¥iCloud¥[illegible]¥NMR[illegible]¥2019NMR_K¥2019NMR(agilent)¥koba¥SK19060104_C.fid¥fid					*Frequency (MHz)*	75.46
Nucleus	13C	*Number of Transients*	1024	*Original Points Count*	19335	*Points Count*	65536
Pulse Sequence	s2pul	*Receiver Gain*	30.00	*Solvent*	CHLOROFORM-d	*Spectrum Offset (Hz)*	8955.5654
Spectrum Type	STANDARD	*Sweep Width (Hz)*	22675.74	*Temperature (degree C)*	AMBIENT TEMPERATURE		

6c-C_SK19060104_C.esp

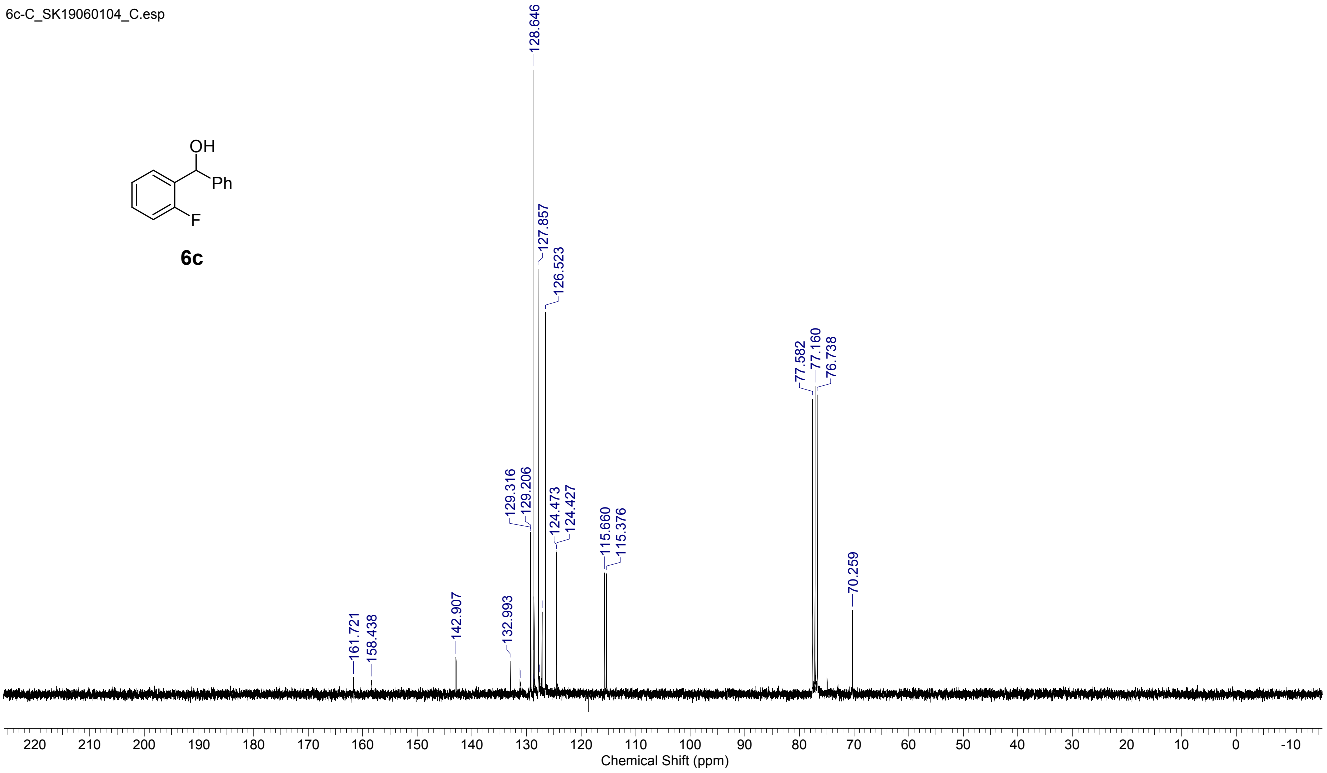

2019/03/02 15:40:19

**Acquisition Time (sec)**	2.1863	**Comment**	single_pulse	**Date**	22 Feb 2017 14:10:24		
**Date Stamp**	22 Feb 2017 14:09:33						
**File Name**	¥¥Mac¥iCloud¥[illegible]NMR[illegible]¥2016NMR_K¥2016NMR(JEOL)¥[illegible]¥SY17022208_Proton-1-1.jdf					**Frequency (MHz)**	399.78
**Nucleus**	1H	**Number of Transients**	8	**Origin**	ECA	**Original Points Count**	16384
**Owner**	delta	**Points Count**	32768	**Pulse Sequence**	proton.jxp	**Solvent**	CHLOROFORM-d
**Spectrum Offset (Hz)**	2003.9476	**Sweep Width (Hz)**	7494.00	**Temperature (degree C)**	19.300		

SY17022208_Proton-1-1.jdf

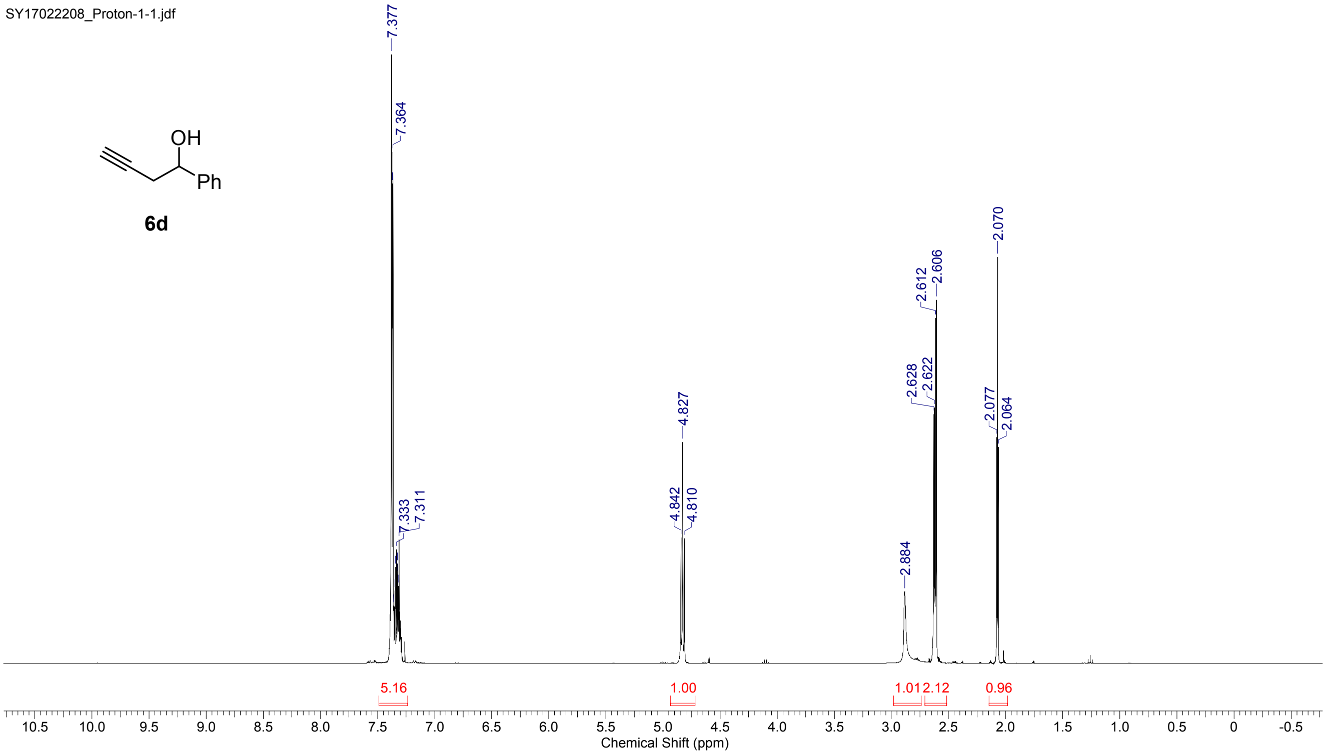

2019/03/02 15:43:06

**Acquisition Time (sec)**	1.0381	**Comment**	single pulse decoupled gated NOE			**Date**	22 Feb 2017 14:17:31
**Date Stamp**	22 Feb 2017 14:12:24						
**File Name**	¥¥Mac¥iCloud¥[illegible]NMR[illegible]¥2016NMR_K¥2016NMR(JEOL)¥[illegible]¥SY17022208_Carbon-1-1.jdf					**Frequency (MHz)**	100.53
**Nucleus**	13C	**Number of Transients**	128	**Origin**	ECA	**Original Points Count**	32768
**Owner**	delta	**Points Count**	65536	**Pulse Sequence**	carbon.jxp	**Solvent**	CHLOROFORM-d
**Spectrum Offset (Hz)**	10036.4756	**Sweep Width (Hz)**	31565.66	**Temperature (degree C)**	19.300		

SY17022208_Carbon-1-1.jdf

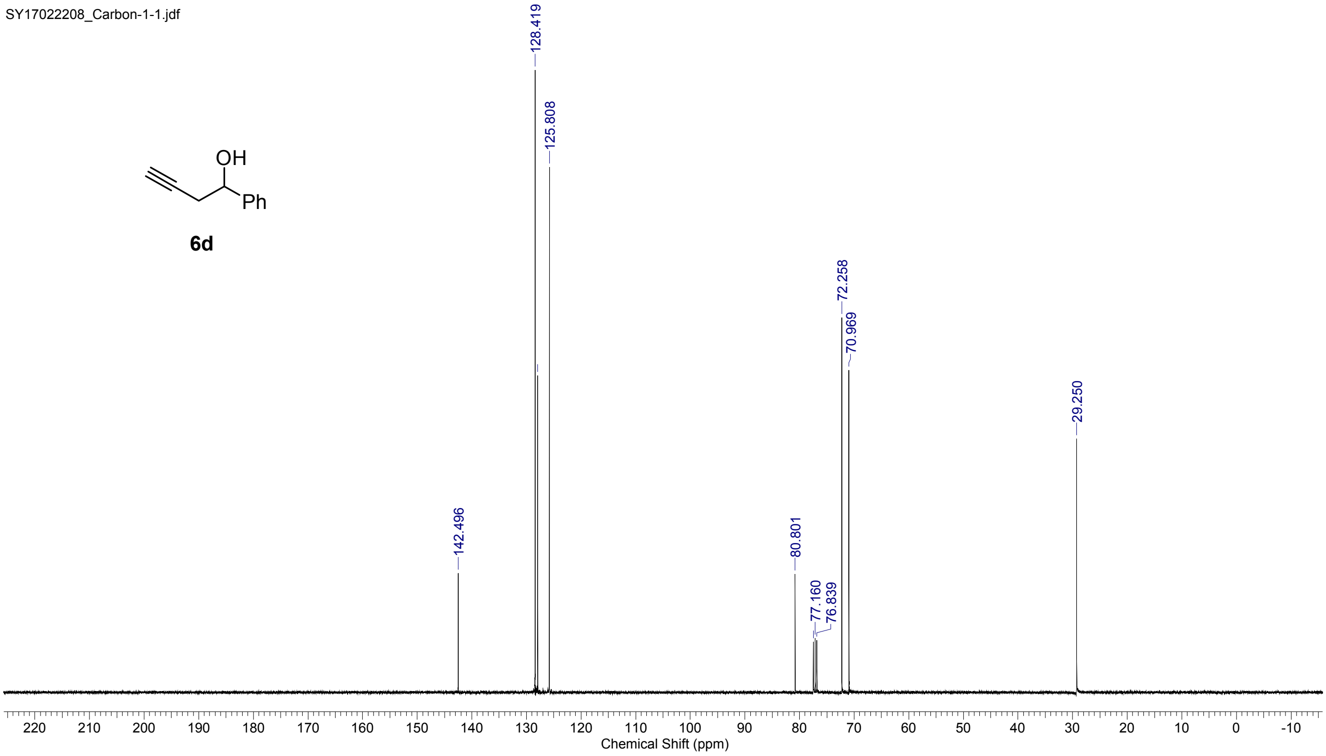

2019/03/02 16:00:03

Acquisition Time (sec)	2.1863	***Comment***	single pulse	***Date***	24 Nov 2016 08:16:25		
Date Stamp	24 Nov 2016 08:15:34						
File Name	¥¥Mac¥iCloud¥[illegible]¥NMR[illegible]¥2016NMR_K¥2016NMR(JEOL)¥[illegible]¥SY16112401_Proton-1-1.jdf					***Frequency (MHz)***	399.78
Nucleus	1H	***Number of Transients***	8	***Origin***	ECA	***Original Points Count***	16384
Owner	delta	***Points Count***	32768	***Pulse Sequence***	proton.jxp	***Solvent***	CHLOROFORM-d
Spectrum Offset (Hz)	2003.2618	***Sweep Width (Hz)***	7494.00	***Temperature (degree C)***	17.200		

SY16112401_Proton-1-1.jdf

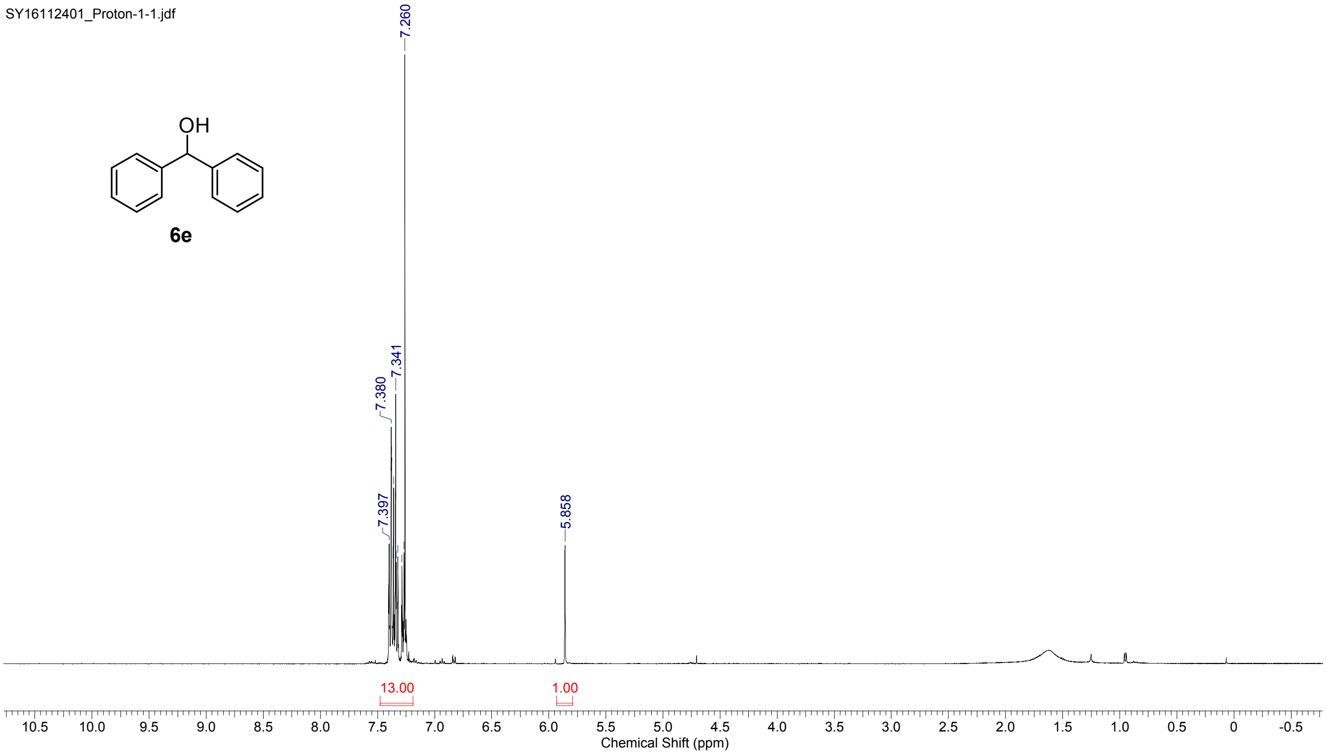

2019/03/02 16:09:08

Acquisition Time (sec)	1.0381	***Comment***	single pulse decoupled gated NOE			***Date***	22 Feb 2017 13:10:04
Date Stamp	22 Feb 2017 13:04:52						
File Name	¥¥Mac¥iCloud¥[illegible]¥NMR[illegible]¥2016NMR_K¥2016NMR(JEOL)¥[illegible]¥SY17022207_Carbon-1-1.jdf					***Frequency (MHz)***	100.53
Nucleus	13C	***Number of Transients***	130	***Origin***	ECA	***Original Points Count***	32768
Owner	delta	***Points Count***	65536	***Pulse Sequence***	carbon.jxp	***Solvent***	CHLOROFORM-d
Spectrum Offset (Hz)	10049.4805	***Sweep Width (Hz)***	31565.66	***Temperature (degree C)***	19.100		

SY17022207_Carbon-1-1.jdf

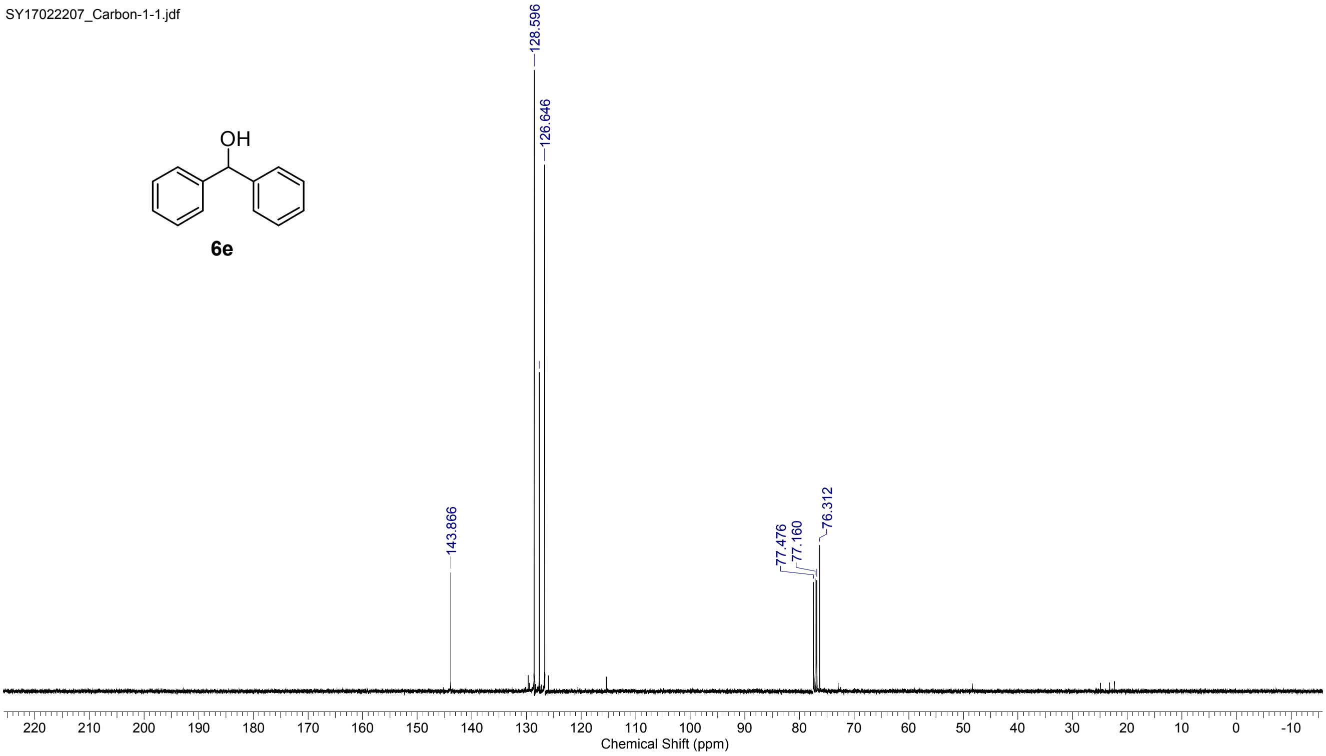

2019/05/27 11:34:09

Acquisition Time (sec)	3.5561	*Comment*	STANDARD 1H OBSERVE			*Date*	May 27 2019
Date Stamp	May 27 2019	*File Name*	¥¥Mac¥iCloud¥[illegible]NMR[illegible]¥2019NMR_K¥2019NMR(agilent)¥koba¥SK19052701.fid¥fid				
Frequency (MHz)	300.05	*Nucleus*	1H	*Number of Transients*	16	*Original Points Count*	17080
Points Count	65536	*Pulse Sequence*	s2pul	*Receiver Gain*	34.00	*Solvent*	CHLOROFORM-d
Spectrum Offset (Hz)	1698.8181	*Spectrum Type*	STANDARD	*Sweep Width (Hz)*	4803.07	*Temperature (degree C)*	AMBIENT TEMPERATURE

6f-H(SK19052701).esp

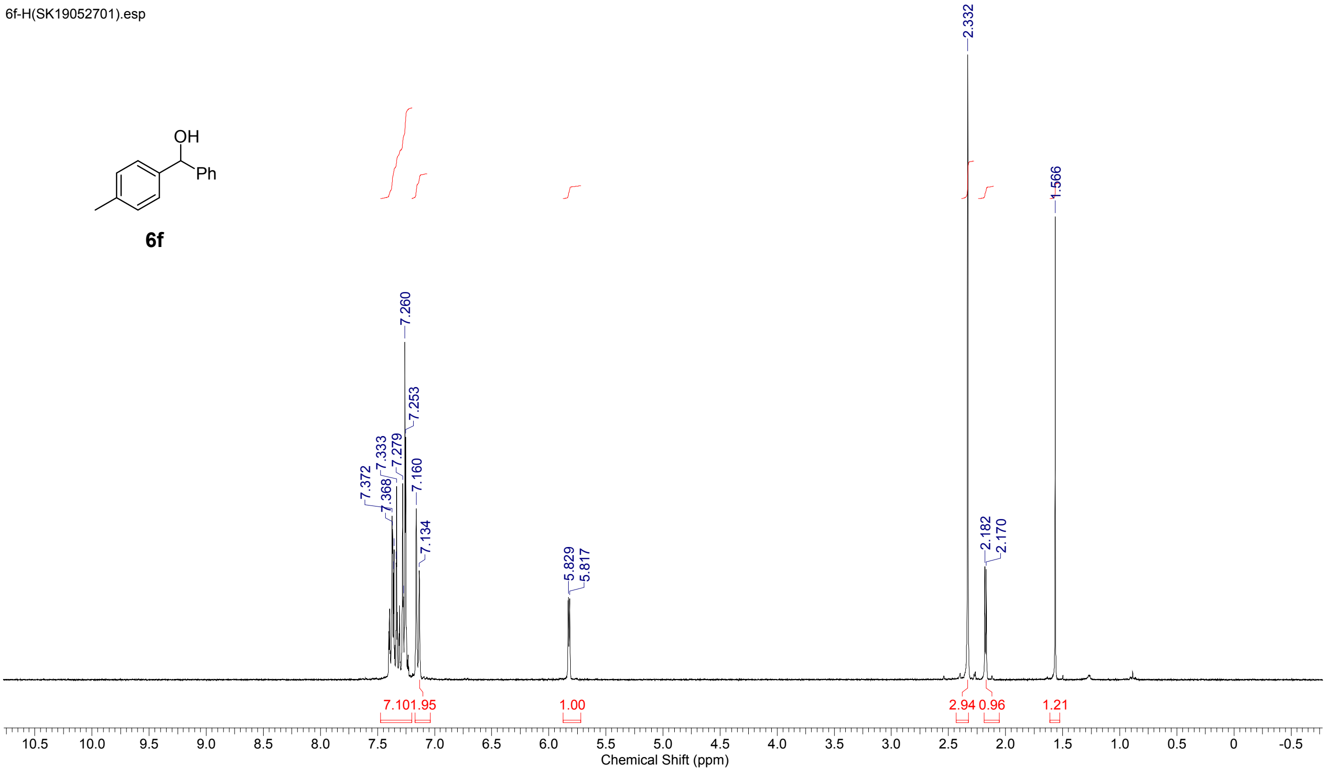

2019/05/27 11:50:14

Acquisition Time (sec)	0.8527	*Comment*	c13-NMR	*Date*	May 27 2019	*Date Stamp*	May 27 2019
File Name	¥¥Mac¥iCloud¥[illegible]NMR[illegible]¥2019NMR_K¥2019NMR(agilent)¥koba¥SK19052701_C.fid¥fid					*Frequency (MHz)*	75.46
Nucleus	13C	*Number of Transients*	1024	*Original Points Count*	19335	*Points Count*	65536
Pulse Sequence	s2pul	*Receiver Gain*	30.00	*Solvent*	CHLOROFORM-d	*Spectrum Offset (Hz)*	8957.2959
Spectrum Type	STANDARD	*Sweep Width (Hz)*	22675.74	*Temperature (degree C)*	AMBIENT TEMPERATURE		

SK19052701_C

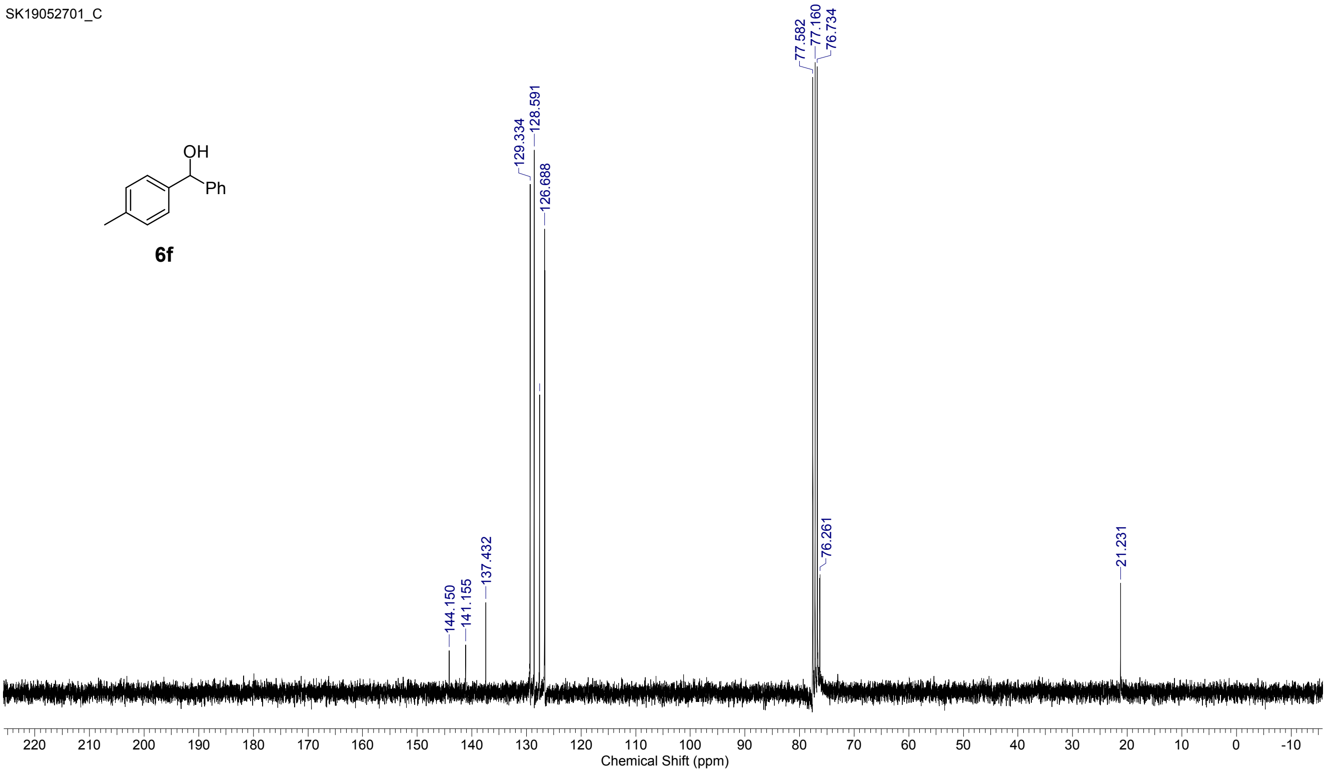

2019/06/04 8:51:12

Acquisition Time (sec)	3.5561	*Comment*	STANDARD 1H OBSERVE			*Date*	Jun 1 2019
Date Stamp	Jun 1 2019	*File Name*	¥¥Mac¥iCloud¥[illegible]NMR[illegible]¥2019NMR_K¥2019NMR(agilent)¥koba¥SK19060101.fid¥fid				
Frequency (MHz)	300.05	*Nucleus*	1H	*Number of Transients*	16	*Original Points Count*	17080
Points Count	65536	*Pulse Sequence*	s2pul	*Receiver Gain*	34.00	*Solvent*	CHLOROFORM-d
Spectrum Offset (Hz)	1698.8915	*Spectrum Type*	STANDARD	*Sweep Width (Hz)*	4803.07	*Temperature (degree C)*	AMBIENT TEMPERATURE

SK19060101

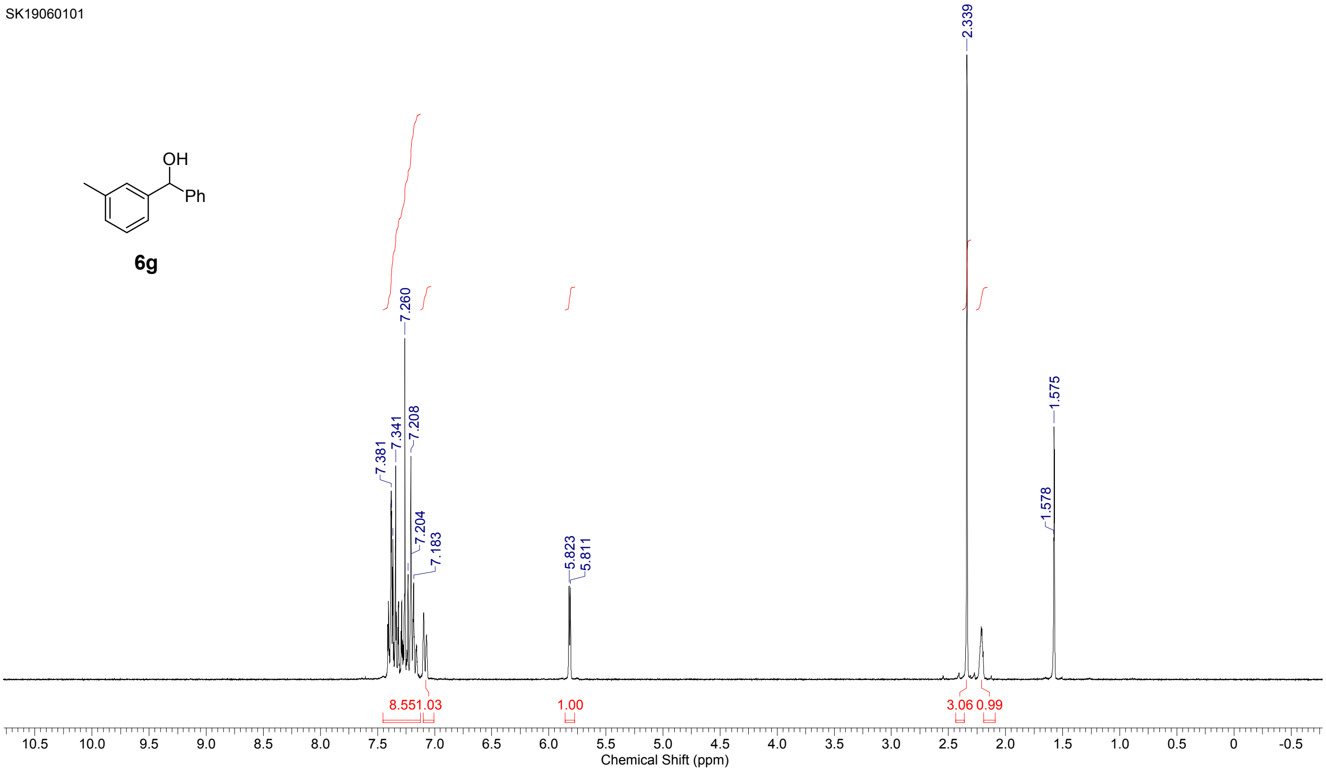

2019/06/04 8:58:21

Acquisition Time (sec)	0.8527	*Comment*	c13-NMR	*Date*	Jun 1 2019	*Date Stamp*	Jun 1 2019
File Name	¥¥Mac¥iCloud¥[illegible]NMR[illegible]¥2019NMR_K¥2019NMR(agilent)¥koba¥SK19060101_C.fid¥fid					*Frequency (MHz)*	75.46
Nucleus	13C	*Number of Transients*	512	*Original Points Count*	19335	*Points Count*	65536
Pulse Sequence	s2pul	*Receiver Gain*	30.00	*Solvent*	CHLOROFORM-d	*Spectrum Offset (Hz)*	8955.9121
Spectrum Type	STANDARD	*Sweep Width (Hz)*	22675.74	*Temperature (degree C)*	AMBIENT TEMPERATURE		

SK19060101_C

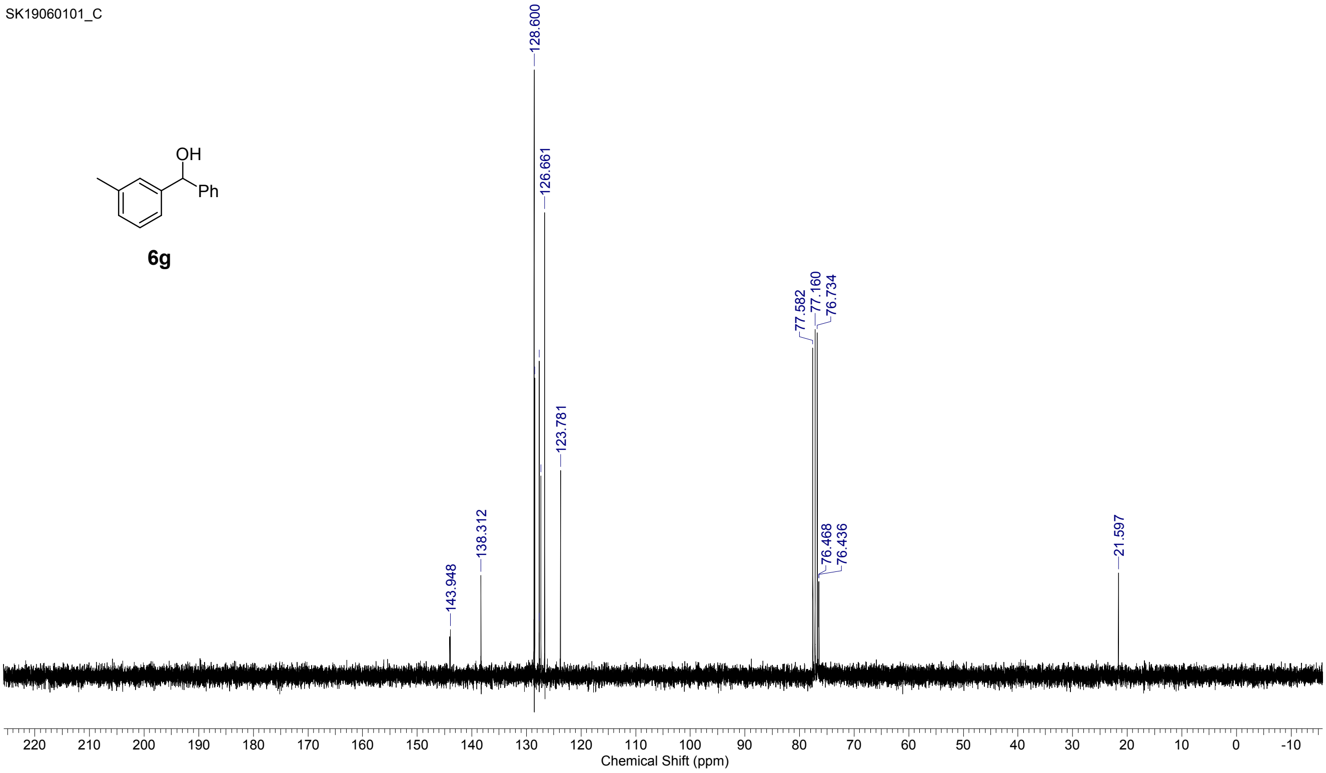

2019/06/04 9:17:20

Acquisition Time (sec)	3.5561	Comment	STANDARD 1H OBSERVE			Date	Jun 1 2019
Date Stamp	Jun 1 2019	File Name	¥¥Mac¥iCloud¥[illegible]¥NMR[illegible]¥2019NMR_K¥2019NMR(agilent)¥koba¥SK19060102.fid¥fid				
Frequency (MHz)	300.05	Nucleus	1H	Number of Transients	16	Original Points Count	17080
Points Count	65536	Pulse Sequence	s2pul	Receiver Gain	24.00	Solvent	CHLOROFORM-d
Spectrum Offset (Hz)	1678.7367	Spectrum Type	STANDARD	Sweep Width (Hz)	4803.07	Temperature (degree C)	AMBIENT TEMPERATURE

SK19060102

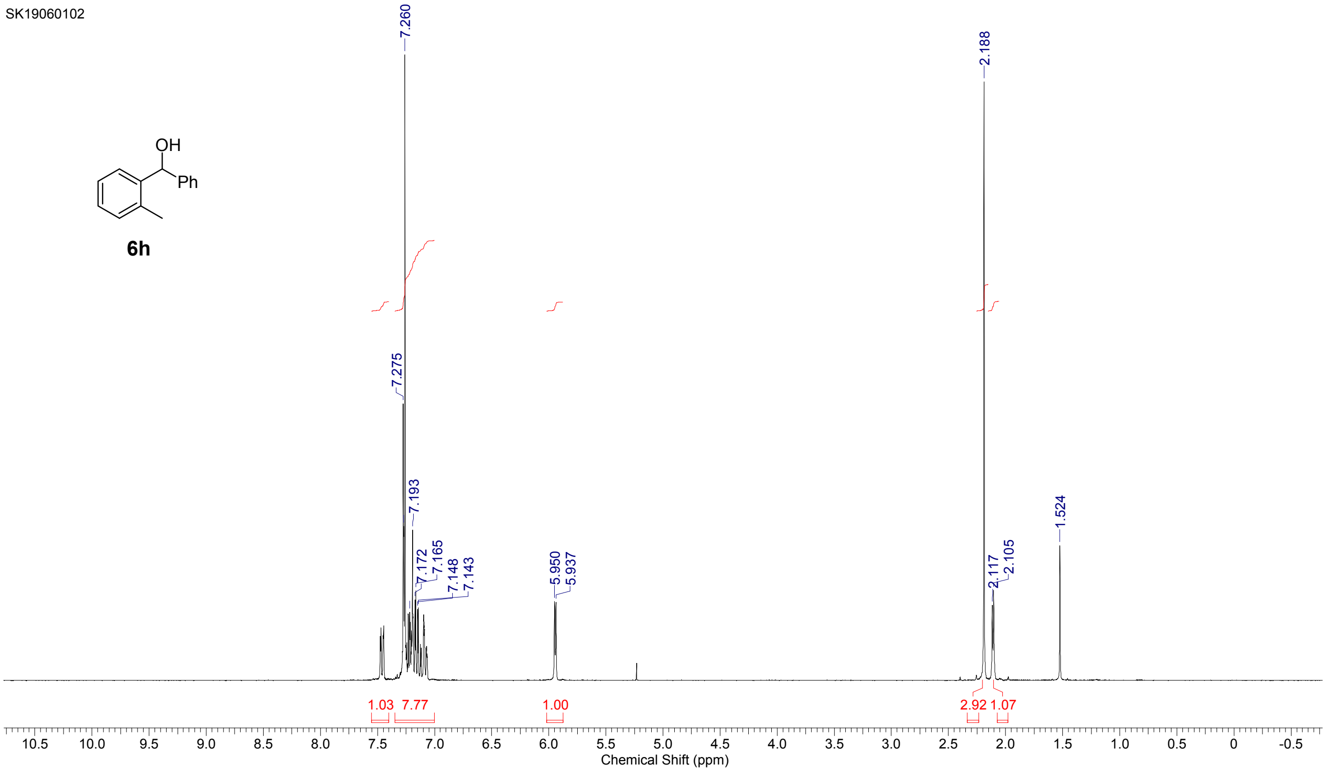

2019/06/04 9:28:56

Acquisition Time (sec)	1.0381	Comment	single pulse decoupled gated NOE			Date	22 Feb 2017 10:18:29
Date Stamp	22 Feb 2017 10:11:22						
File Name	¥¥Mac¥iCloud¥[illegible]¥NMR[illegible]¥2016NMR_K¥2016NMR(JEOL)¥[illegible]NMR2016¥SY17022203_Carbon-1-1.jdf						
Frequency (MHz)	100.53	Nucleus	13C	Number of Transients	180	Origin	ECA
Original Points Count	32768	Owner	delta	Points Count	65536	Pulse Sequence	carbon.jxp
Solvent	CHLOROFORM-d	Spectrum Offset (Hz)	10052.8525	Sweep Width (Hz)	31565.66	Temperature (degree C)	18.100

SY17022203_Carbon-1-1.jdf

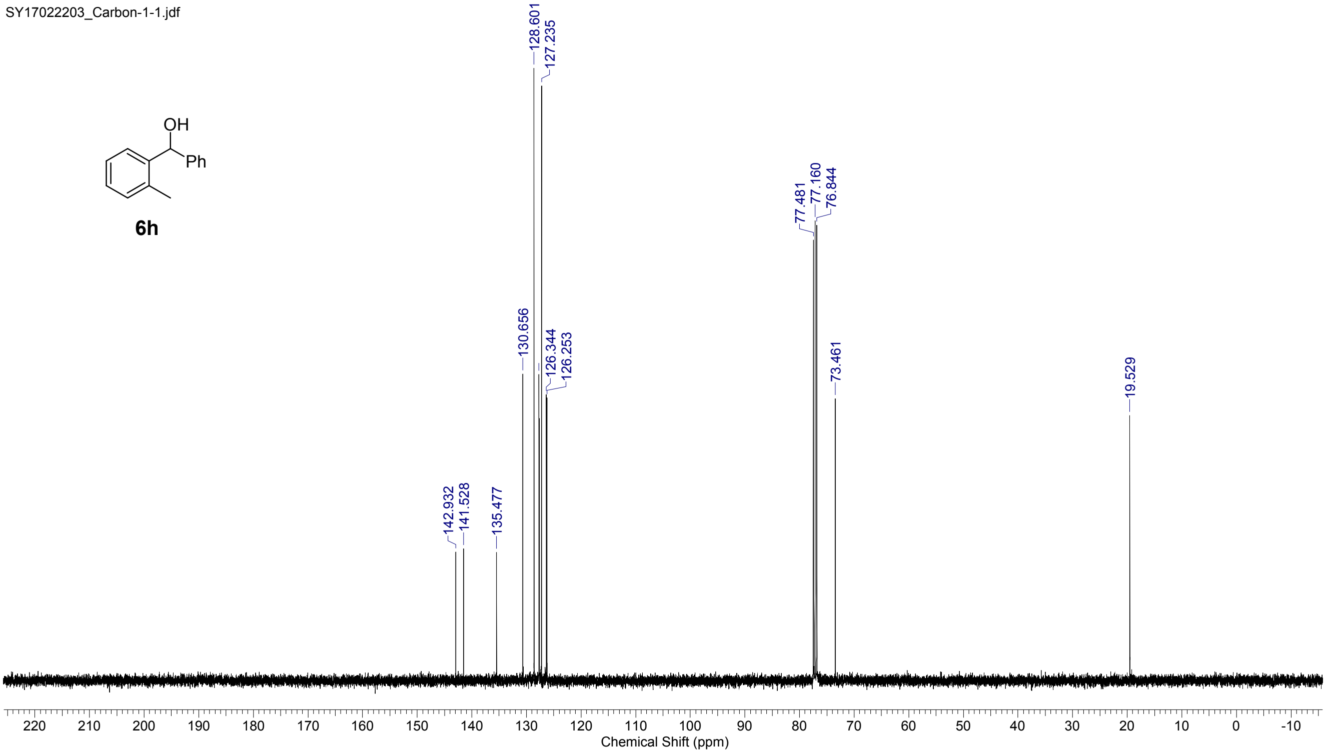

2019/03/02 16:52:32

**Acquisition Time (sec)**	2.1863	**Comment**	single_pulse	**Date**	21 Jun 2017 13:01:19		
**Date Stamp**	21 Jun 2017 13:00:28						
**File Name**	¥¥Mac¥iCloud¥[illegible]NMR[illegible]¥2017NMR_K¥2017NMR(JEOL)¥[illegible]¥TT17062101_Proton-1-1.jdf					**Frequency (MHz)**	399.78
**Nucleus**	1H	**Number of Transients**	8	**Origin**	ECA	**Original Points Count**	16384
**Owner**	delta	**Points Count**	32768	**Pulse Sequence**	proton.jxp	**Solvent**	CHLOROFORM-d
**Spectrum Offset (Hz)**	2003.4905	**Sweep Width (Hz)**	7494.00	**Temperature (degree C)**	20.900		

TT17062101_Proton-1-1.jdf

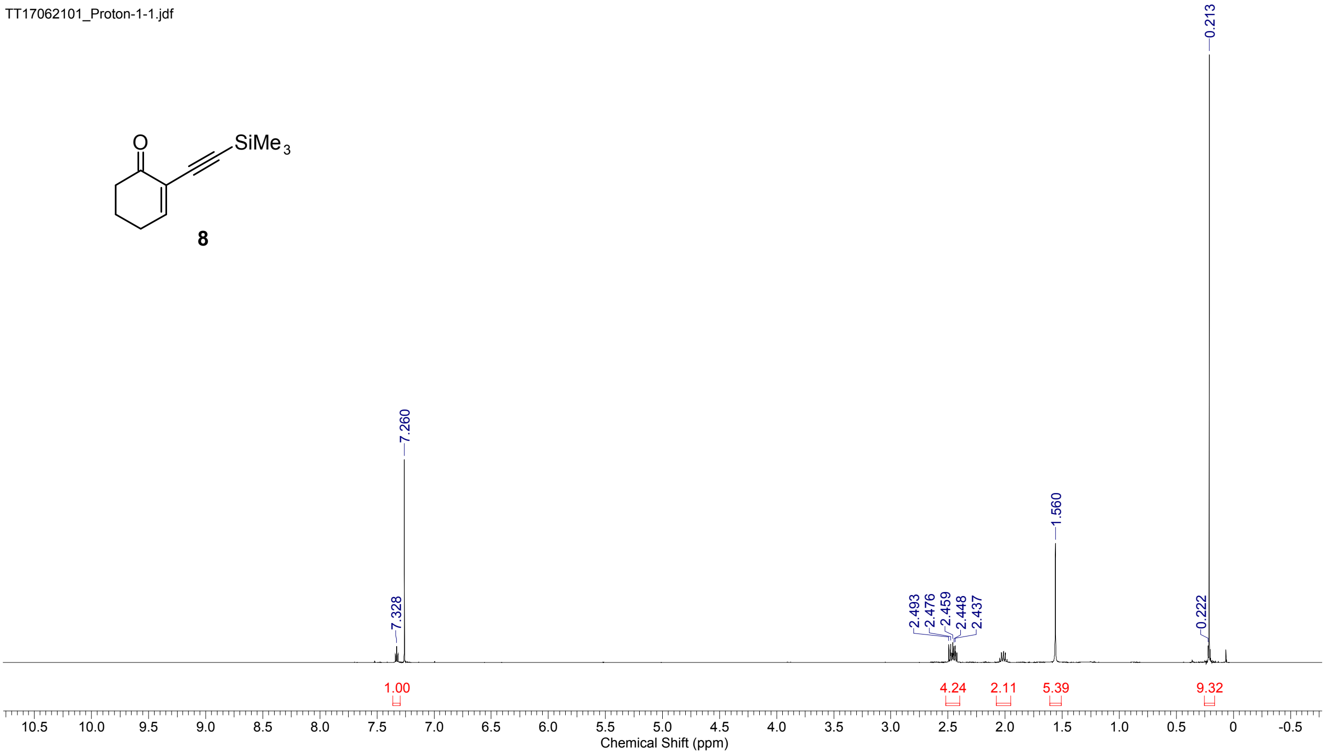

2019/03/07 19:44:44

**Acquisition Time (sec)**	1.0381	**Comment**	single pulse decoupled gated NOE			**Date**	26 Jan 2019 09:34:46
**Date Stamp**	26 Jan 2019 08:55:14						
**File Name**	¥¥Mac¥iCloud¥[illegible]NMR[illegible]¥2018NMR_K¥2018NMR(JEOL)¥[illegible]NMR2018¥TT19012601_Carbon-1-1.jdf						
**Frequency (MHz)**	100.53	**Nucleus**	13C	**Number of Transients**	1024	**Origin**	ECA
**Original Points Count**	32768	**Owner**	delta	**Points Count**	65536	**Pulse Sequence**	carbon.jxp
**Solvent**	CHLOROFORM-d	**Spectrum Offset (Hz)**	10053.8154	**Sweep Width (Hz)**	31565.66	**Temperature (degree C)**	17.300

TT19012601_Carbon-1-1.jdf

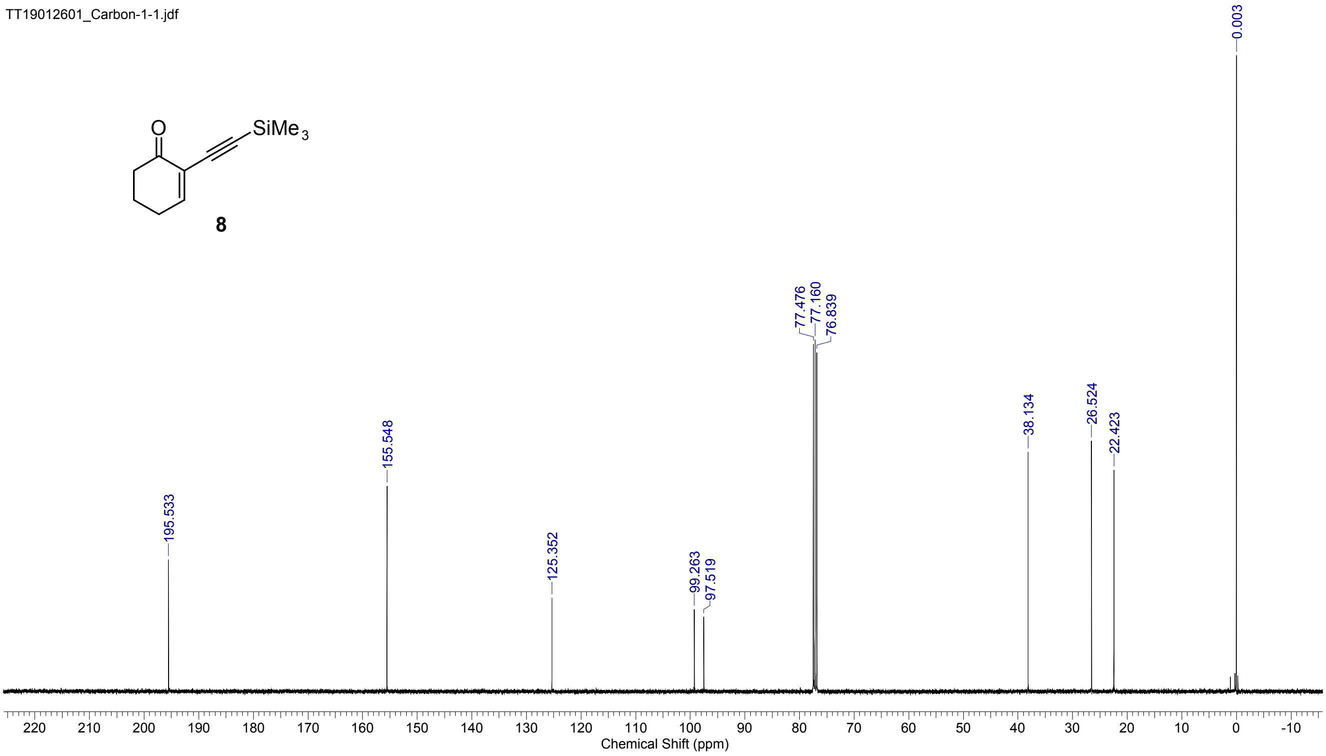

2019/03/04 10:16:00

**Acquisition Time (sec)**	2.1863	**Comment**	single pulse	**Date**	07 Dec 2017 11:41:15		
**Date Stamp**	07 Dec 2017 11:40:24						
**File Name**	¥¥Mac¥iCloud¥[illegible]NMR[illegible]¥2017NMR_K¥2017NMR(JEOL)¥[illegible]¥TT17120702_Proton-1-1.jdf					**Frequency (MHz)**	399.78
**Nucleus**	1H	**Number of Transients**	8	**Origin**	ECA	**Original Points Count**	16384
**Owner**	delta	**Points Count**	32768	**Pulse Sequence**	proton.jxp	**Solvent**	CHLOROFORM-d
**Spectrum Offset (Hz)**	2003.4905	**Sweep Width (Hz)**	7494.00	**Temperature (degree C)**	20.500		

TT17120702_Proton-1-1.jdf

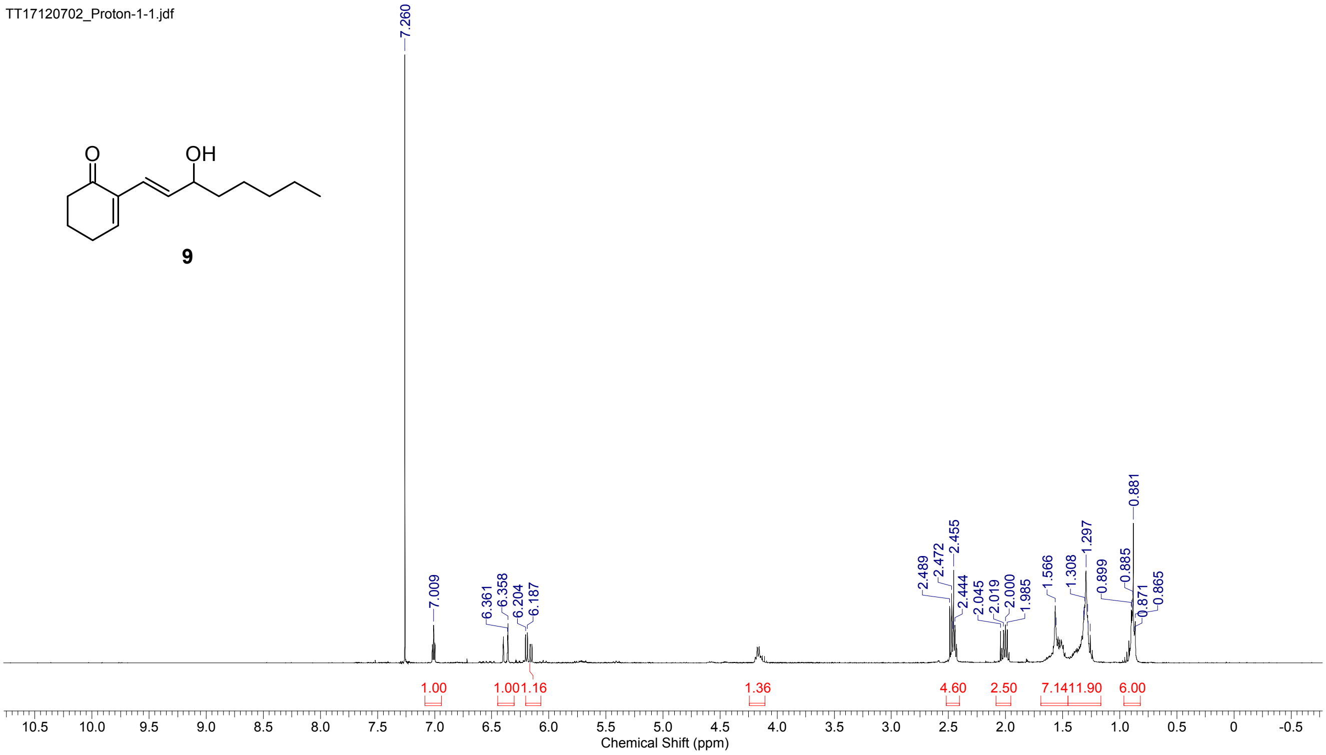

2019/05/18 14:12:29

**Acquisition Time (sec)**	1.0381	**Comment**	single pulse decoupled gated NOE			**Date**	18 May 2019 09:41:10
**Date Stamp**	18 May 2019 09:01:38						
**File Name**	¥¥Mac¥iCloud¥[illegible]NMR[illegible]¥2019NMR_K¥2019NMR(JEOL)¥[illegible]NMR2019¥SK19051801_Carbon-1-1.jdf						
**Frequency (MHz)**	100.53	**Nucleus**	13C	**Number of Transients**	1024	**Origin**	ECA
**Original Points Count**	32768	**Owner**	delta	**Points Count**	65536	**Pulse Sequence**	carbon.jxp
**Solvent**	CHLOROFORM-d	**Spectrum Offset (Hz)**	10056.2236	**Sweep Width (Hz)**	31565.66	**Temperature (degree C)**	19.500

SK19051801_Carbon-1-1.jdf

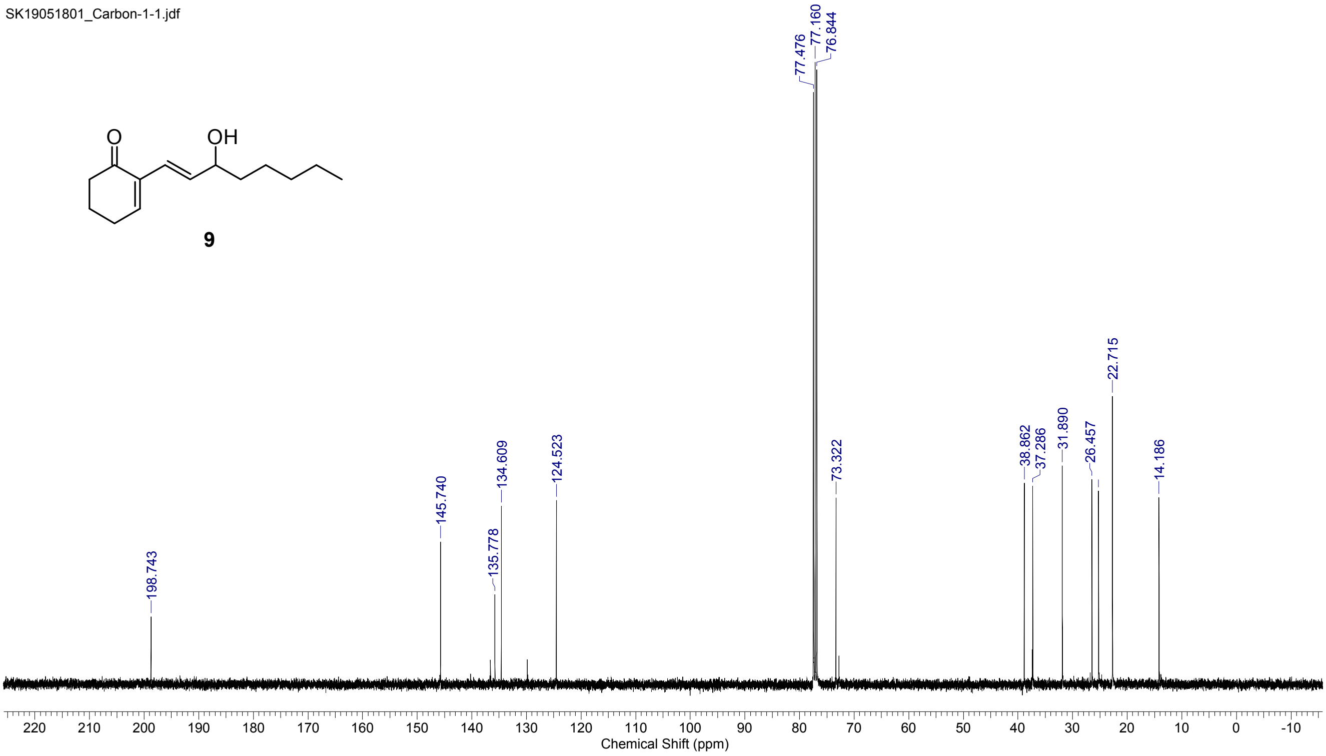

2019/03/04 11:47:02

Acquisition Time (sec)	2.1863	*Comment*	single_pulse	*Date*	13 Sep 2017 12:04:48		
Date Stamp	13 Sep 2017 12:03:57						
File Name	¥¥Mac¥iCloud¥[illegible]NMR[illegible]¥2017NMR_K¥2017NMR(JEOL)¥[illegible]¥TT17091301_Proton-1-1.jdf					*Frequency (MHz)*	399.78
Nucleus	1H	*Number of Transients*	8	*Origin*	ECA	*Original Points Count*	16384
Owner	delta	*Points Count*	32768	*Pulse Sequence*	proton.jxp	*Solvent*	CHLOROFORM-d
Spectrum Offset (Hz)	2003.4905	*Sweep Width (Hz)*	7494.00	*Temperature (degree C)*	22.900		

15-H.esp

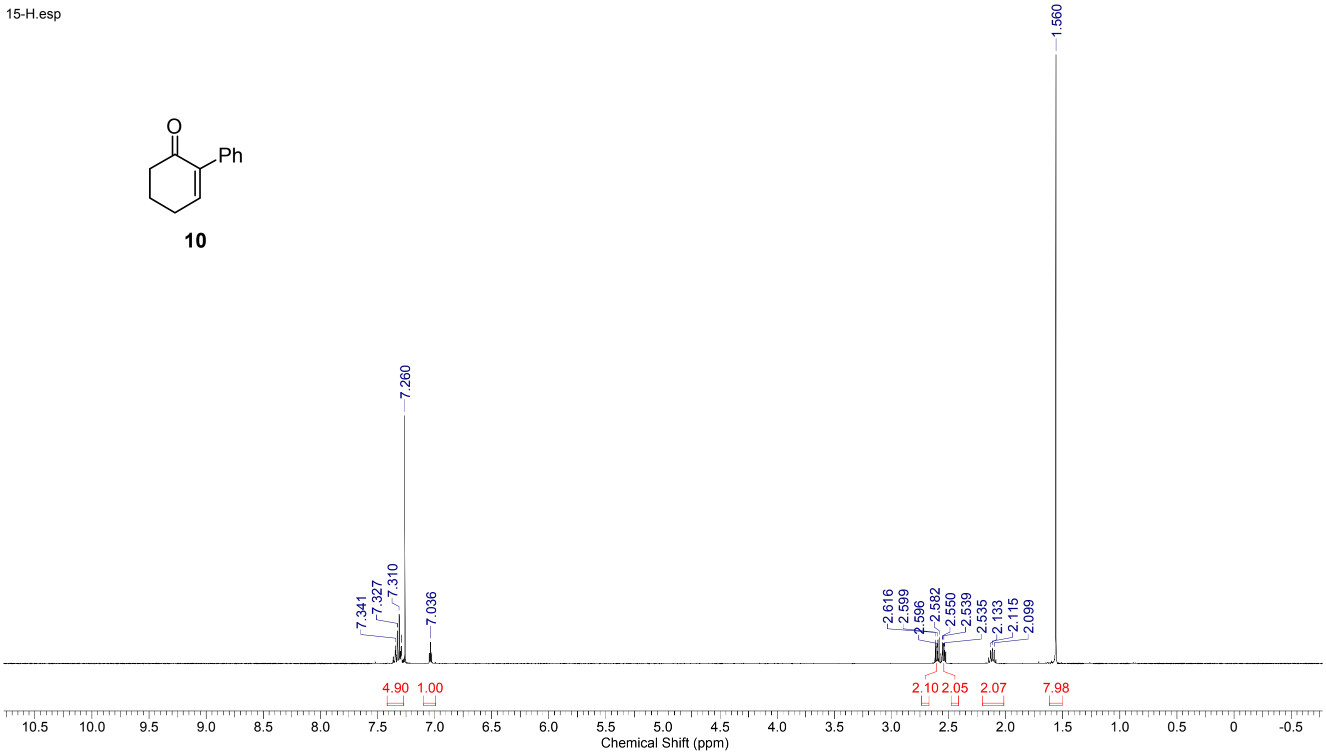

2019/03/07 19:49:19

Acquisition Time (sec)	1.0381	*Comment*	single pulse decoupled gated NOE			*Date*	19 Feb 2019 11:26:54
Date Stamp	19 Feb 2019 10:47:22						
File Name	¥¥Mac¥iCloud¥[illegible]NMR[illegible]¥2018NMR_K¥2018NMR(JEOL)¥[illegible]NMR2018¥TT19021901_Carbon-1-1.jdf						
Frequency (MHz)	100.53	*Nucleus*	13C	*Number of Transients*	1024	*Origin*	ECA
Original Points Count	32768	*Owner*	delta	*Points Count*	65536	*Pulse Sequence*	carbon.jxp
Solvent	CHLOROFORM-d	*Spectrum Offset (Hz)*	10053.8154	*Sweep Width (Hz)*	31565.66	*Temperature (degree C)*	19.700

TT19021901_Carbon-1-1.jdf

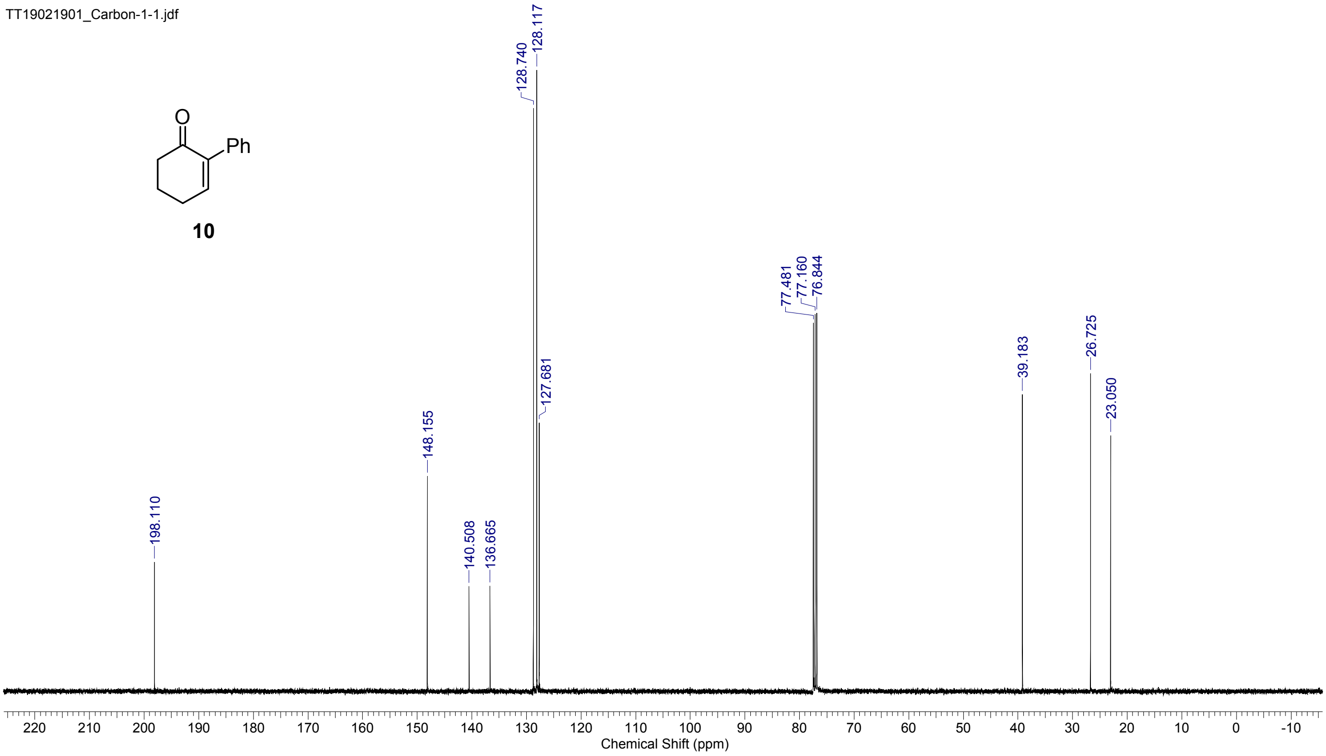

2019/03/04 13:05:30

Acquisition Time (sec)	3.5561	*Comment*	STANDARD 1H OBSERVE			*Date*	Jun 30 2017
Date Stamp	Jun 30 2017	*File Name*	¥¥Mac¥iCloud¥[illegible]NMR[illegible]¥2017NMR_K¥2017NMR(agilent)¥tamura¥TT17063002.fid¥fid				
Frequency (MHz)	300.05	*Nucleus*	1H	*Number of Transients*	4	*Original Points Count*	17080
Points Count	65536	*Pulse Sequence*	s2pul	*Receiver Gain*	18.00	*Solvent*	CHLOROFORM-d
Spectrum Offset (Hz)	1698.5983	*Spectrum Type*	STANDARD	*Sweep Width (Hz)*	4803.07	*Temperature (degree C)*	AMBIENT TEMPERATURE

16-H.esp

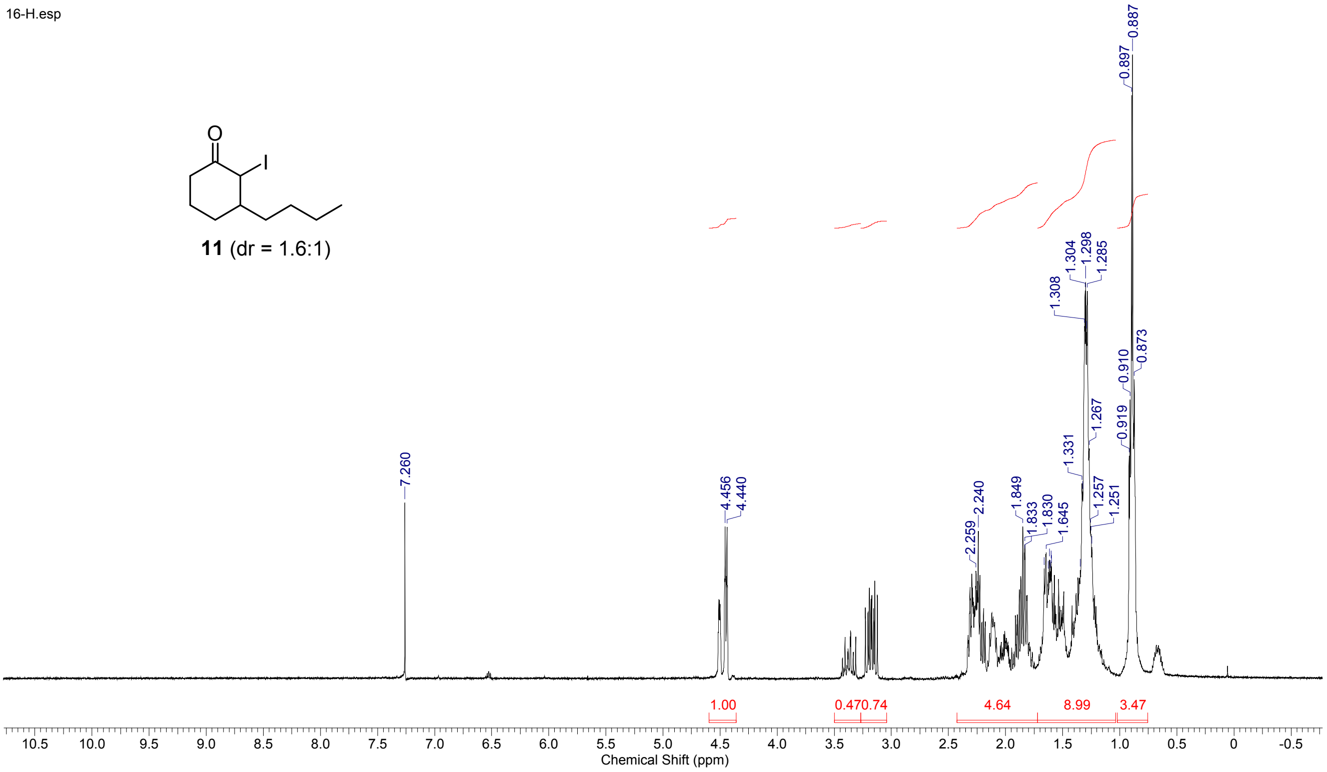

2019/03/04 12:38:13

Acquisition Time (sec)	0.8527	*Comment*	c13-NMR	*Date*	Jun 30 2017	*Date Stamp*	Jun 30 2017
File Name	¥¥Mac¥iCloud¥[illegible]NMR[illegible]¥2017NMR_K¥2017NMR(agilent)¥tamura¥TT17063002_C.fid¥fid					*Frequency (MHz)*	75.46
Nucleus	13C	*Number of Transients*	672	*Original Points Count*	19335	*Points Count*	65536
Pulse Sequence	s2pul	*Receiver Gain*	30.00	*Solvent*	CHLOROFORM-d	*Spectrum Offset (Hz)*	8954.8730
Spectrum Type	STANDARD	*Sweep Width (Hz)*	22675.74	*Temperature (degree C)*	AMBIENT TEMPERATURE		

16-C.esp

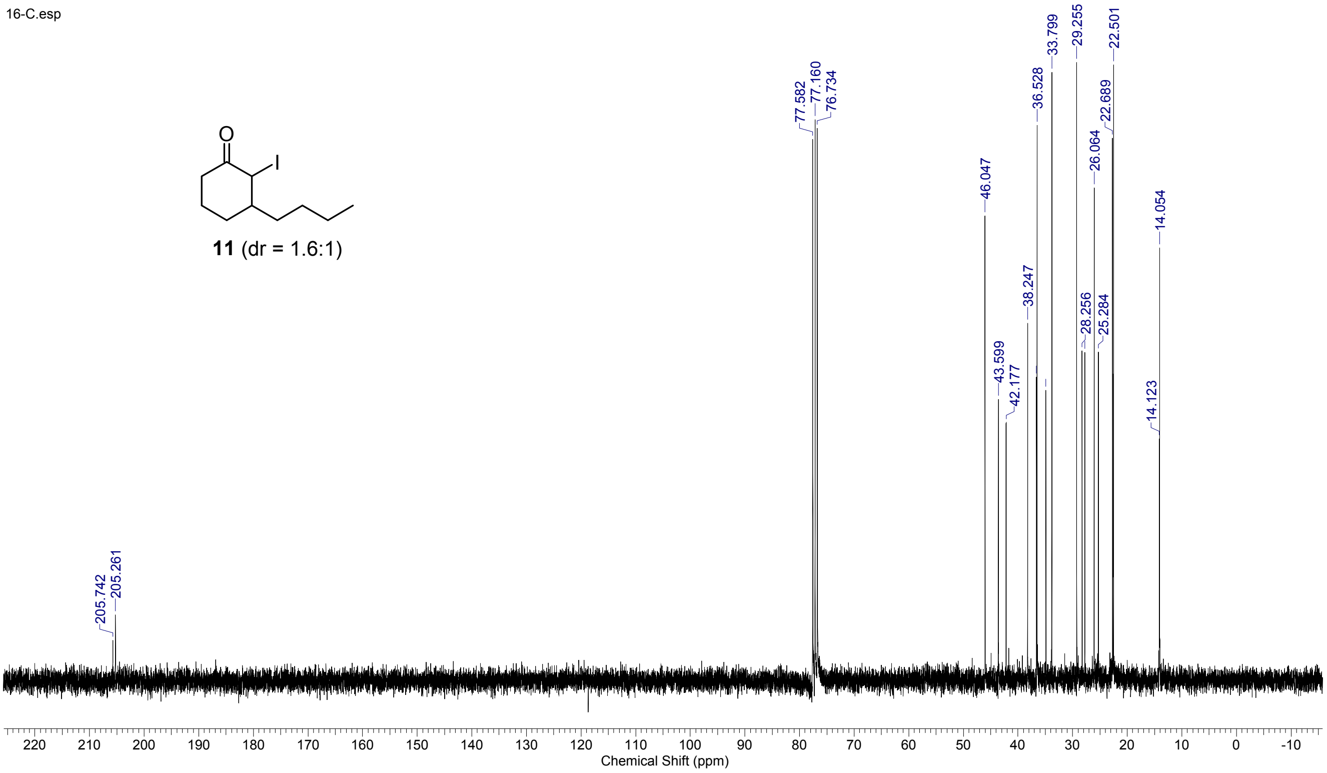

2019/05/18 16:59:14

Acquisition Time (sec)	2.1863	Comment	single_pulse	Date	18 May 2019 12:01:26		
Date Stamp	18 May 2019 12:00:35						
File Name	¥¥Mac¥iCloud¥[illegible]NMR[illegible]¥2019NMR_K¥2019NMR(JEOL)¥[illegible]NMR2019¥SK19051803_Proton-1-1.jdf						
Frequency (MHz)	399.78	Nucleus	1H	Number of Transients	8	Origin	ECA
Original Points Count	16384	Owner	delta	Points Count	32768	Pulse Sequence	proton.jxp
Solvent	CHLOROFORM-d	Spectrum Offset (Hz)	1999.8311	Sweep Width (Hz)	7494.00	Temperature (degree C)	19.800

SK19051803_Proton-1-1.jdf

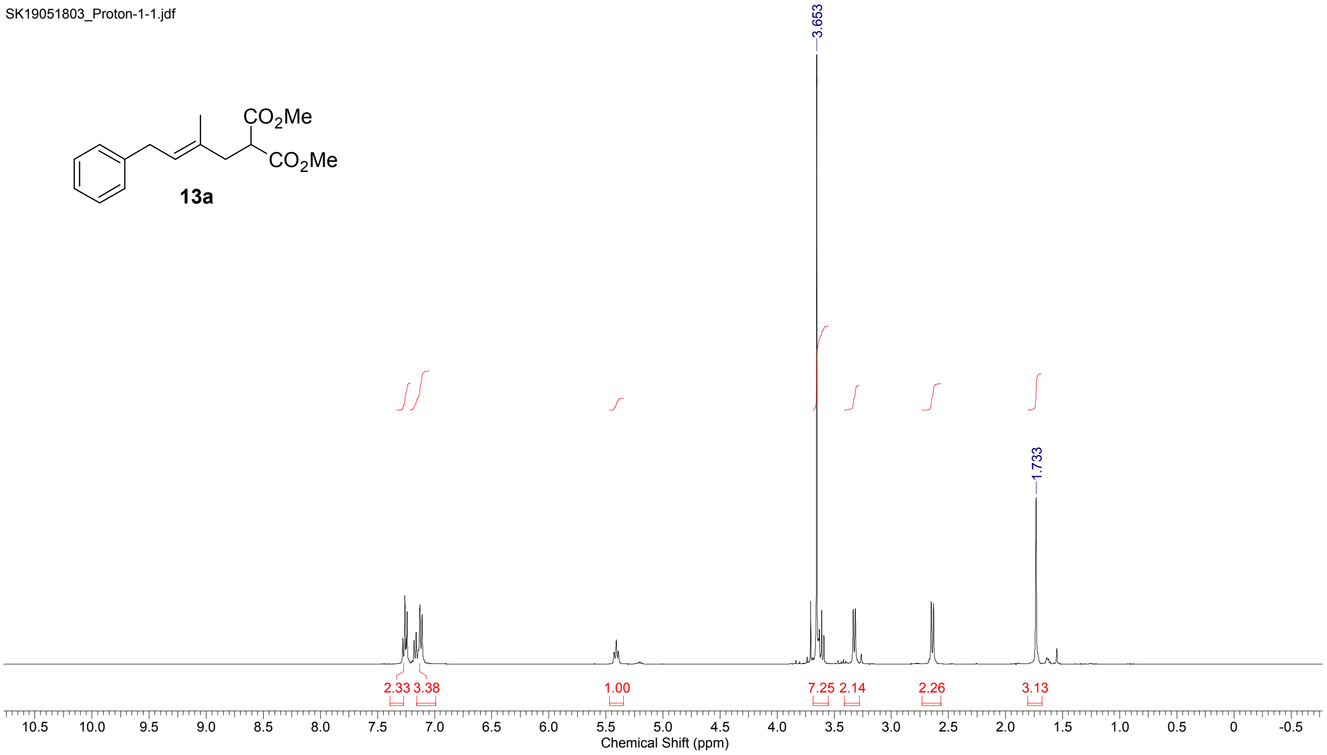

2019/05/18 17:10:38

Acquisition Time (sec)	1.0381	Comment	single pulse decoupled gated NOE	Date	18 May 2019 12:43:20		
Date Stamp	18 May 2019 12:03:48						
File Name	¥¥Mac¥iCloud¥[illegible]NMR[illegible]¥2019NMR_K¥2019NMR(JEOL)¥[illegible]NMR2019¥SK19051803_Carbon-1-1.jdf						
Frequency (MHz)	100.53	Nucleus	13C	Number of Transients	1024	Origin	ECA
Original Points Count	32768	Owner	delta	Points Count	65536	Pulse Sequence	carbon.jxp
Solvent	CHLOROFORM-d	Spectrum Offset (Hz)	10053.8154	Sweep Width (Hz)	31565.66	Temperature (degree C)	19.600

SK19051803_Carbon-1-1.jdf

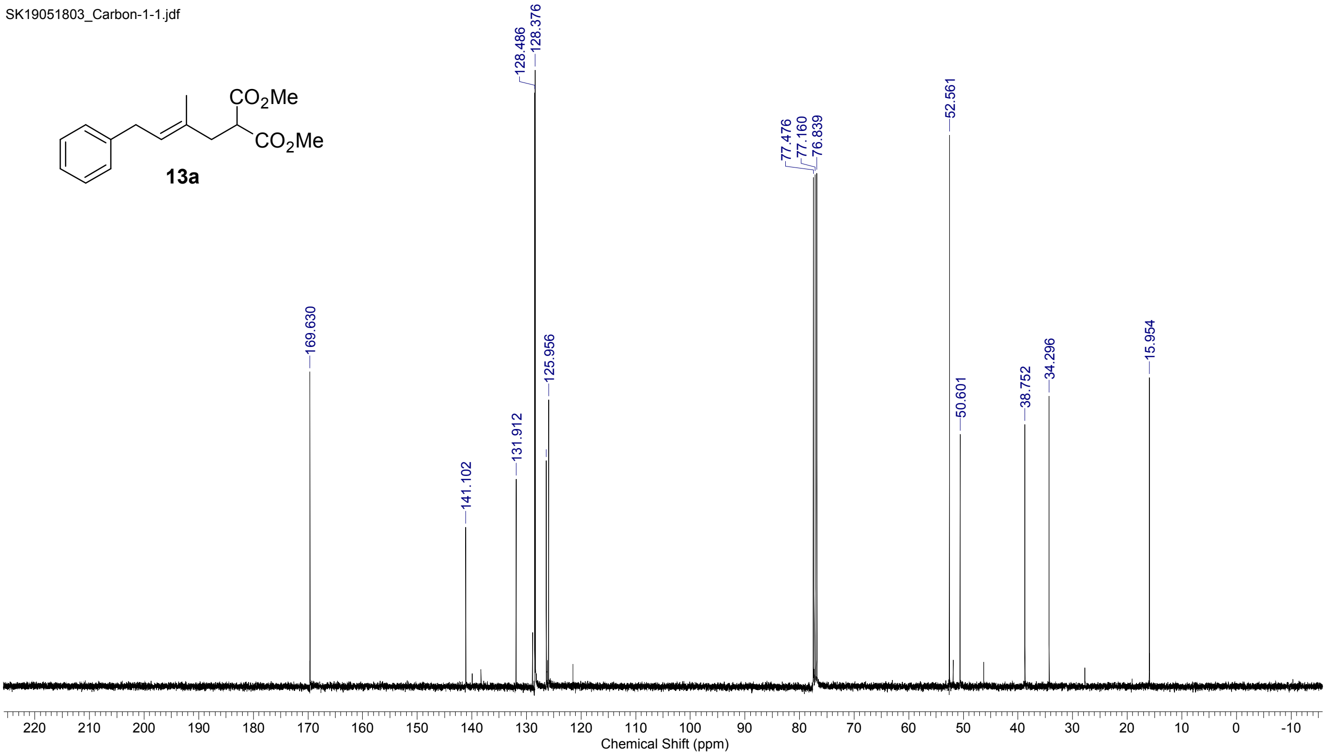

2019/03/04 15:03:16

Acquisition Time (sec)	2.1863	*Comment*	single_pulse	*Date*	27 Oct 2017 13:42:46		
Date Stamp	27 Oct 2017 13:41:55						
File Name	¥¥Mac¥iCloud¥[illegible]NMR[illegible]¥2017NMR_K¥2017NMR(JEOL)¥[illegible]¥TT17102701_Proton-1-1.jdf					*Frequency (MHz)*	399.78
Nucleus	1H	*Number of Transients*	8	*Origin*	ECA	*Original Points Count*	16384
Owner	delta	*Points Count*	32768	*Pulse Sequence*	proton.jxp	*Solvent*	CHLOROFORM-d
Spectrum Offset (Hz)	2003.4905	*Sweep Width (Hz)*	7494.00	*Temperature (degree C)*	19.200		

TT17102701_Proton-1-1.jdf

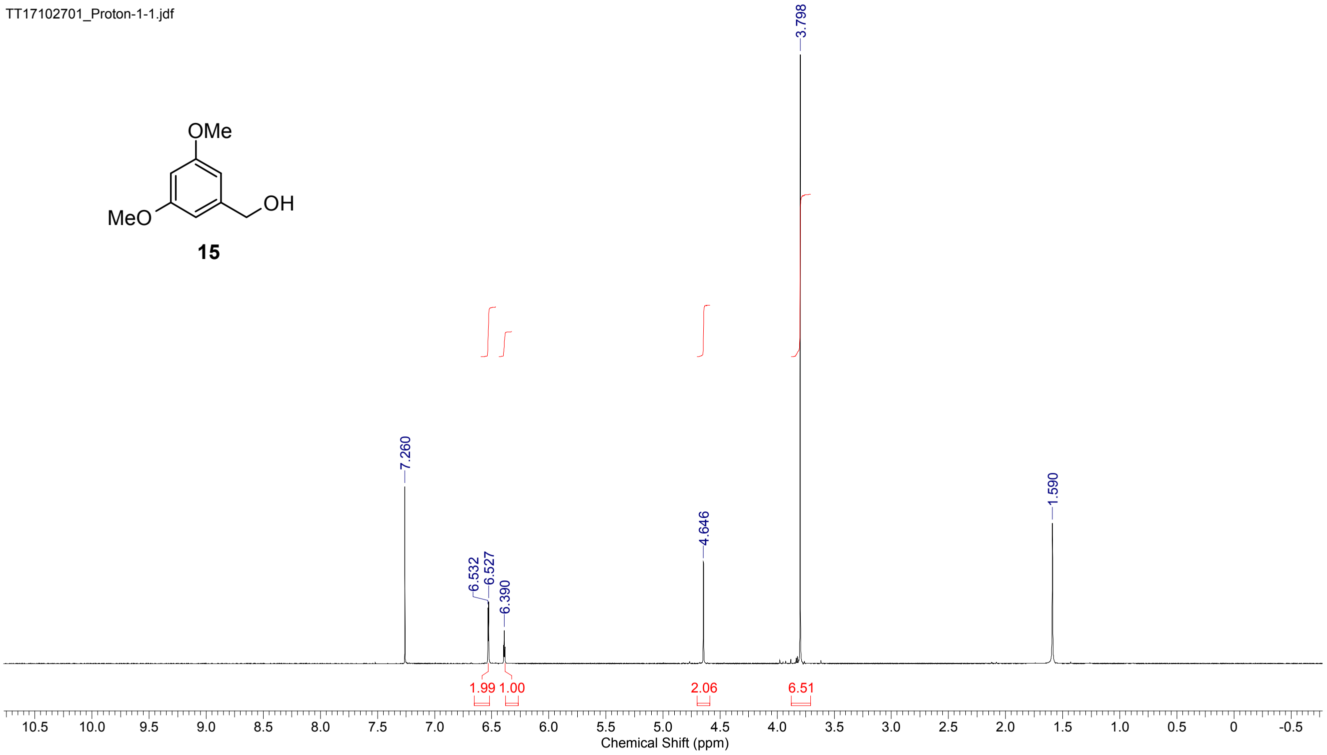

2019/03/13 20:07:24

Acquisition Time (sec)	0.8527	*Comment*	c13-NMR	*Date*	Mar 13 2019	*Date Stamp*	Mar 13 2019		
File Name	¥¥Mac¥iCloud¥[illegible]NMR[illegible]¥2019NMR_K¥koba¥SK19031302_C.fid¥fid					*Frequency (MHz)*	75.46	*Nucleus*	13C
Number of Transients	512	*Original Points Count*	19335	*Points Count*	65536	*Pulse Sequence*	s2pul	*Receiver Gain*	30.00
Solvent	CHLOROFORM-d			*Spectrum Offset (Hz)*	8956.6035	*Spectrum Type*	STANDARD	*Sweep Width (Hz)*	22675.74
Temperature (degree C)	AMBIENT TEMPERATURE								

SK19031302_C

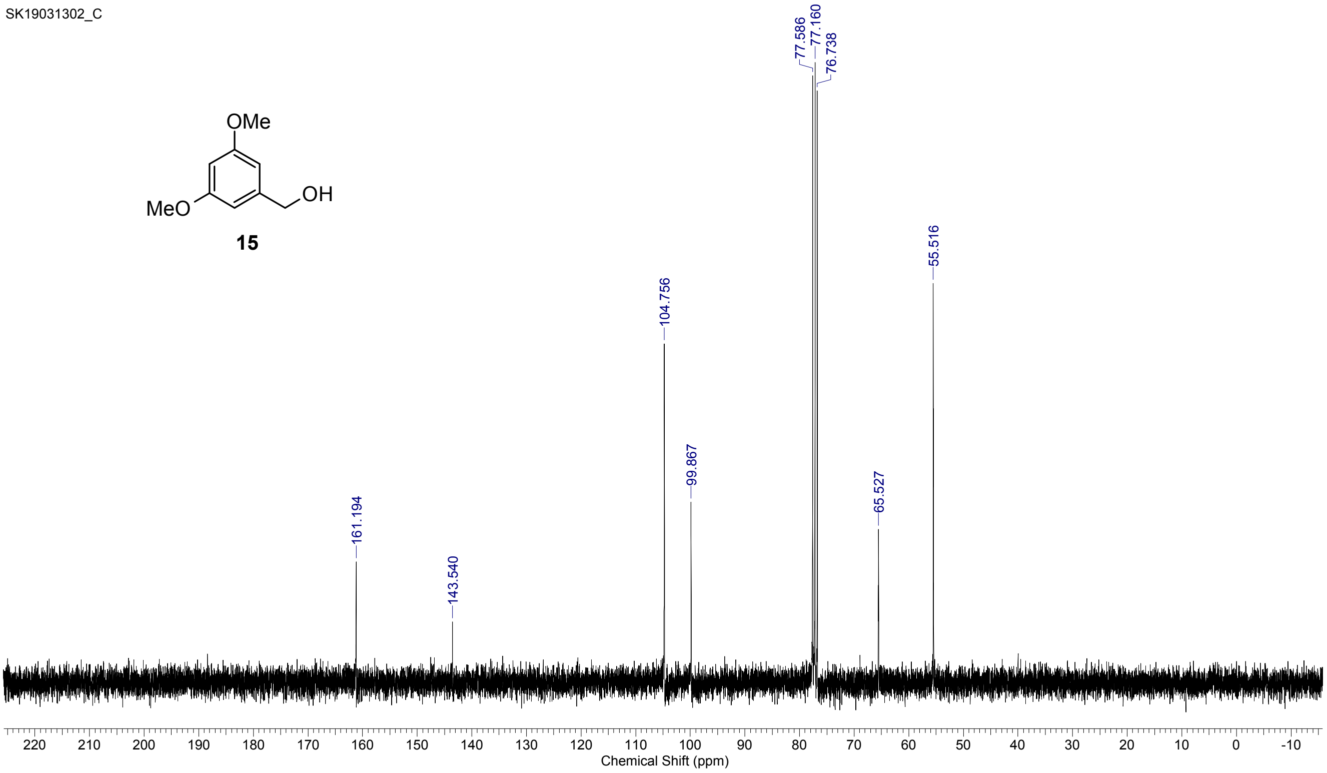

2019/03/13 16:53:34

Acquisition Time (sec)	3.5561	Comment	STANDARD 1H OBSERVE			Date	Mar 13 2019
Date Stamp	Mar 13 2019	File Name	¥¥Mac¥iCloud¥[illegible]NMR[illegible]¥2019NMR_K¥koba¥SK19031303.fid¥fid				
Frequency (MHz)	300.05	Nucleus	1H	Number of Transients	16	Original Points Count	17080
Points Count	65536	Pulse Sequence	s2pul	Receiver Gain	16.00	Solvent	CHLOROFORM-d
Spectrum Offset (Hz)	1699.2579	Spectrum Type	STANDARD	Sweep Width (Hz)	4803.07	Temperature (degree C)	AMBIENT TEMPERATURE

SK19031303

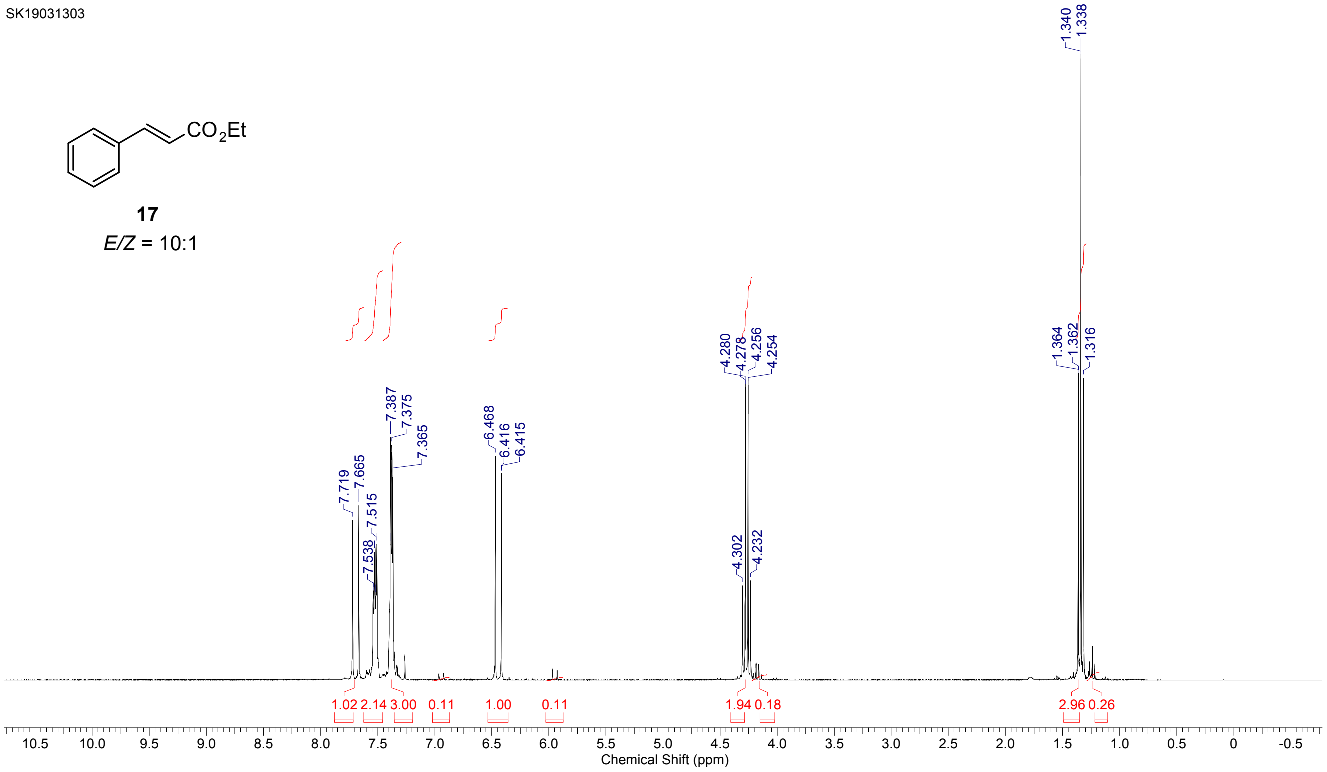

2019/03/13 17:28:42

Acquisition Time (sec)	0.8527	Comment	c13-NMR	Date	Mar 13 2019	Date Stamp	Mar 13 2019		
File Name	¥¥Mac¥iCloud¥[illegible]NMR[illegible]¥2019NMR_K¥koba¥SK19031303_C.fid¥fid					Frequency (MHz)	75.46	Nucleus	13C
Number of Transients	128	Original Points Count	19335	Points Count	65536	Pulse Sequence	s2pul	Receiver Gain	30.00
Solvent	CHLOROFORM-d			Spectrum Offset (Hz)	8952.7979	Spectrum Type	STANDARD	Sweep Width (Hz)	22675.74
Temperature (degree C)	AMBIENT TEMPERATURE								

SK19031303_C

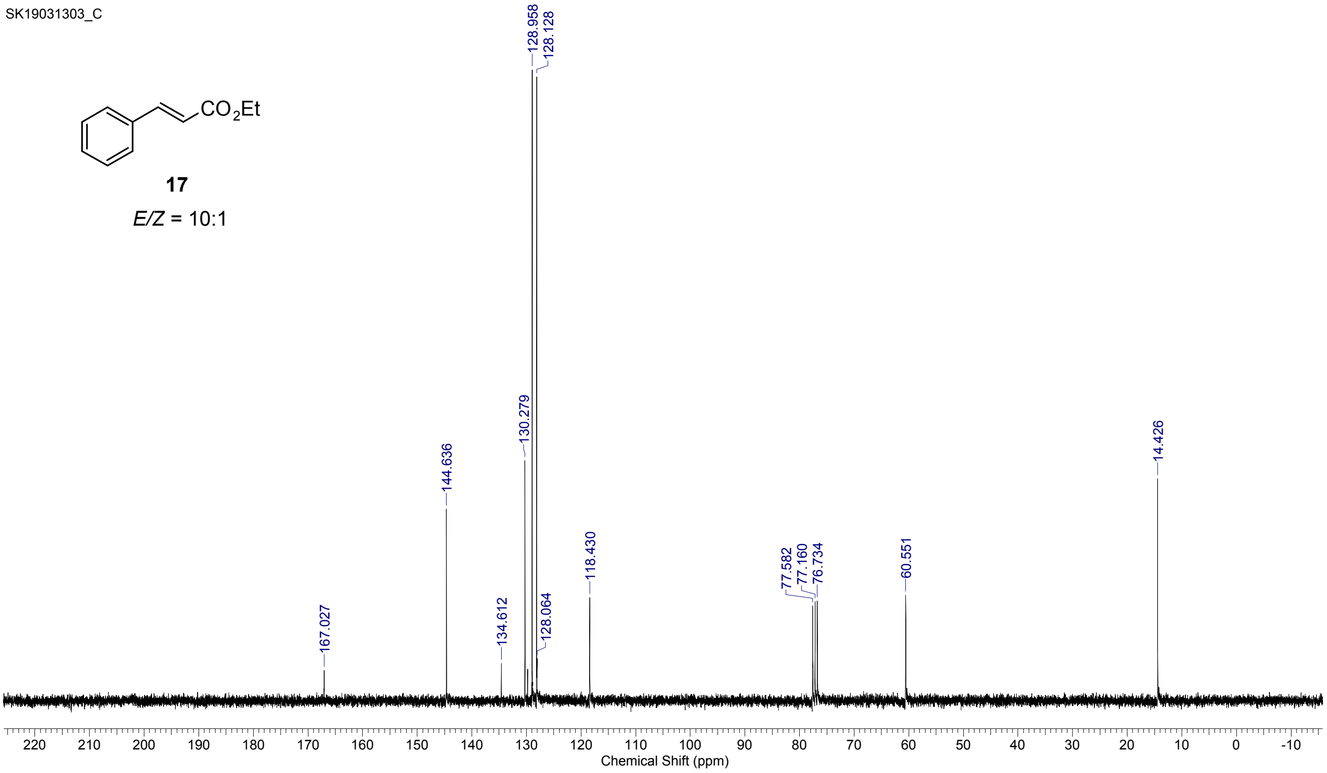

2019/03/04 16:06:39

Acquisition Time (sec)	2.1863	***Comment***	single_pulse	***Date***	16_Jun_2017 09:17:57		
Date Stamp	16_Jun_2017 09:17:05						
File Name	¥¥Mac¥iCloud¥[illegible]¥NMR[illegible]¥2017NMR_K¥2017NMR(JEOL)¥[illegible]¥TT17061601_Proton-1-1.jdf					***Frequency (MHz)***	399.78
Nucleus	1H	***Number of Transients***	8	***Origin***	ECA	***Original Points Count***	16384
Owner	delta	***Points Count***	32768	***Pulse Sequence***	proton.jxp	***Solvent***	CHLOROFORM-d
Spectrum Offset (Hz)	2003.4905	***Sweep Width (Hz)***	7494.00	***Temperature (degree C)***	21.300		

TT17061601_Proton-1-1.jdf

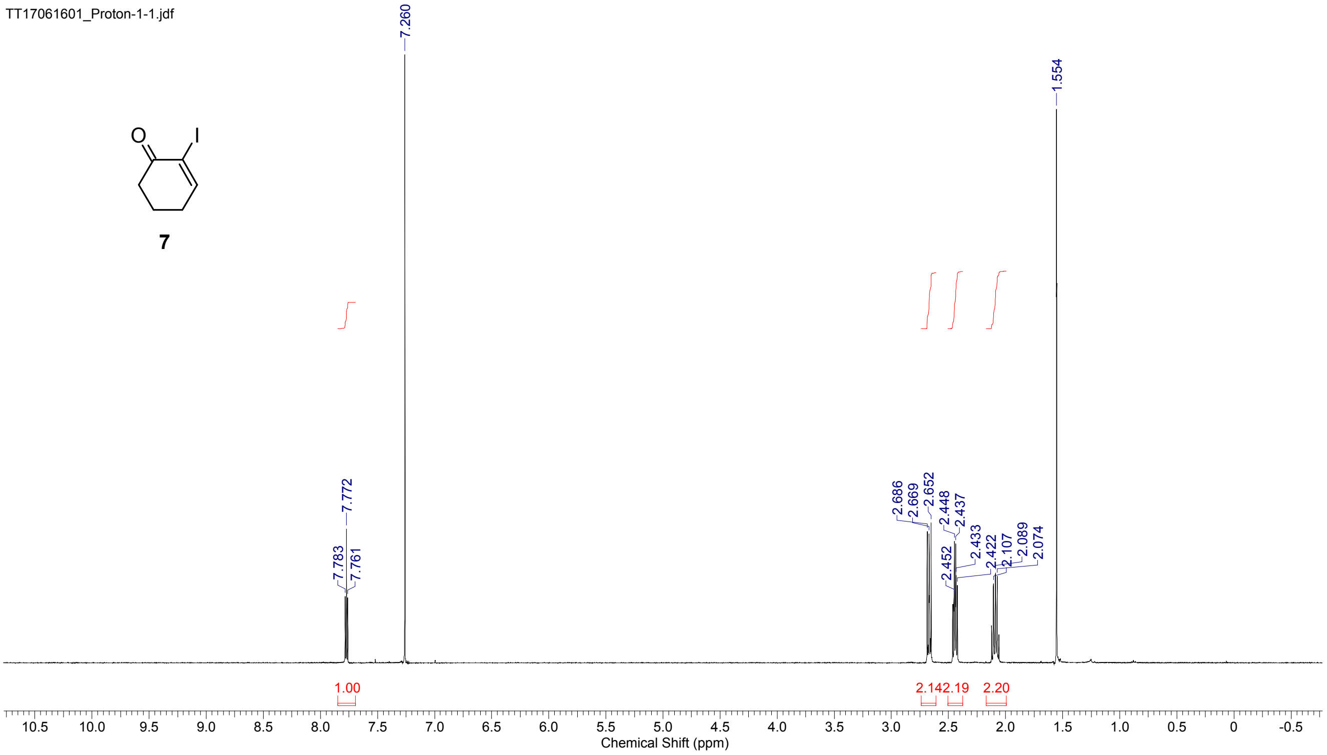

2019/03/07 19:55:27

Acquisition Time (sec)	1.0381	***Comment***	single pulse decoupled gated NOE			***Date***	22_Jan_2019 12:38:09
Date Stamp	22_Jan_2019 11:58:38						
File Name	¥¥Mac¥iCloud¥[illegible]¥NMR[illegible]¥2018NMR_K¥2018NMR(JEOL)¥[illegible]NMR2018¥TT19012204_Carbon-1-1.jdf						
Frequency (MHz)	100.53	***Nucleus***	13C	***Number of Transients***	1024	***Origin***	ECA
Original Points Count	32768	***Owner***	delta	***Points Count***	65536	***Pulse Sequence***	carbon.jxp
Solvent	CHLOROFORM-d	***Spectrum Offset (Hz)***	10054.2969	***Sweep Width (Hz)***	31565.66	***Temperature (degree C)***	20.000

TT19012204_Carbon-1-1.jdf

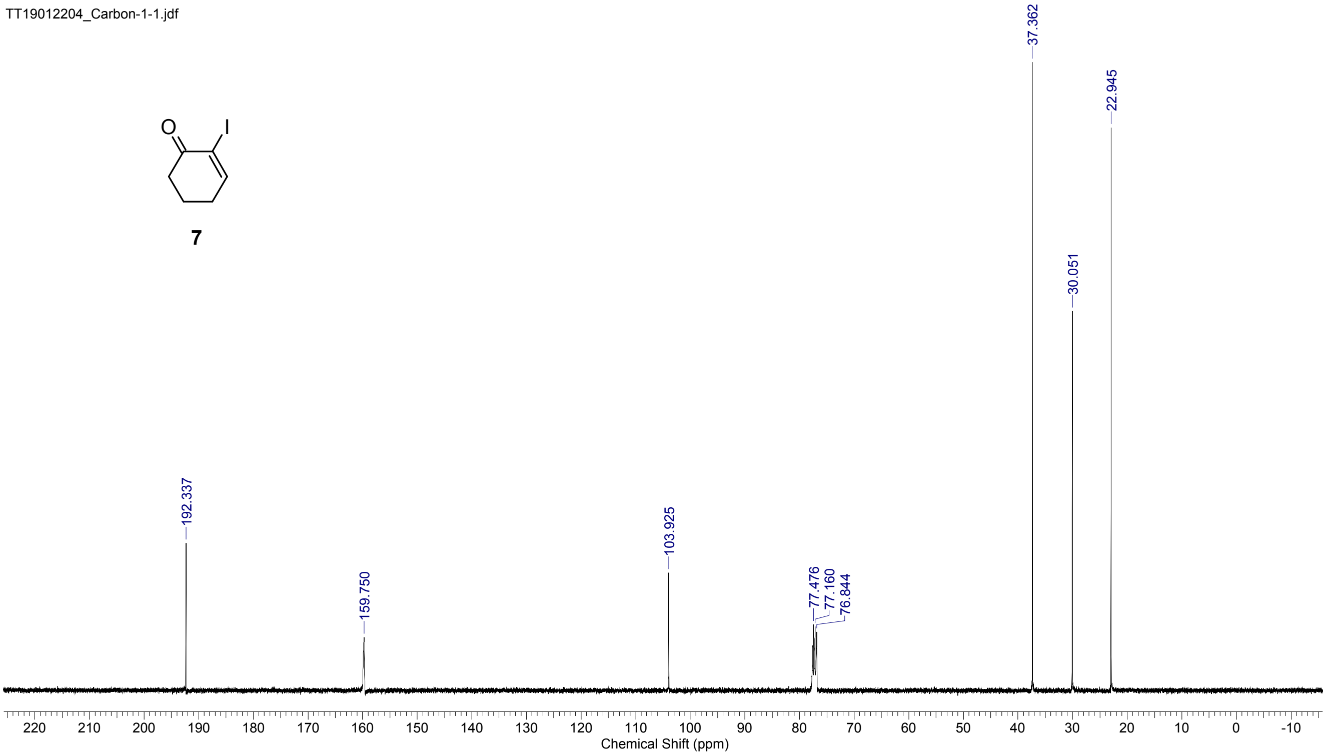

2019/03/06 18:12:27

Acquisition Time (sec)	2.1863	Comment	single pulse	Date	15 Feb 2019 08:46:58		
Date Stamp	15 Feb 2019 08:46:07						
File Name	¥¥Mac¥iCloud¥ [illegible] NMR [illegible] ¥2018NMR_K¥2018NMR(JEOL)¥ [illegible] NMR2018¥TK19021501_Proton-1-1.jdf						
Frequency (MHz)	399.78	Nucleus	1H	Number of Transients	8	Origin	ECA
Original Points Count	16384	Owner	delta	Points Count	32768	Pulse Sequence	proton.jxp
Solvent	CHLOROFORM-d	Spectrum Offset (Hz)	2003.2618	Sweep Width (Hz)	7494.00	Temperature (degree C)	16.700

TK19021501_Proton-1-1.jdf

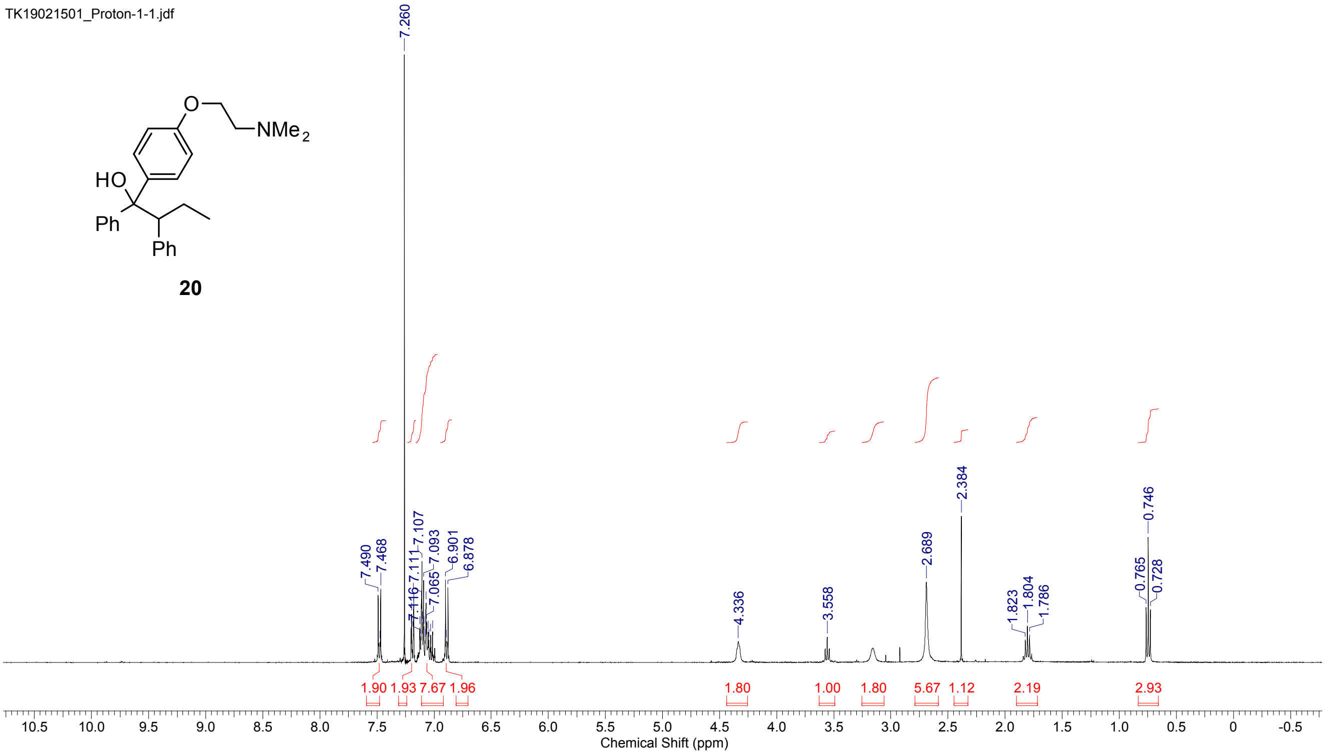

2019/03/06 18:34:43

Acquisition Time (sec)	1.0381	Comment	single pulse decoupled gated NOE	Date	22 Feb 2019 11:32:42		
Date Stamp	22 Feb 2019 11:12:50						
File Name	¥¥Mac¥iCloud¥ [illegible] NMR [illegible] ¥2018NMR_K¥2018NMR(JEOL)¥ [illegible] NMR2018¥TK19022202_Carbon-1-1.jdf						
Frequency (MHz)	100.53	Nucleus	13C	Number of Transients	512	Origin	ECA
Original Points Count	32768	Owner	delta	Points Count	65536	Pulse Sequence	carbon.jxp
Solvent	CHLOROFORM-d	Spectrum Offset (Hz)	10052.8525	Sweep Width (Hz)	31565.66	Temperature (degree C)	21.600

TK19022202_Carbon-1-1.jdf

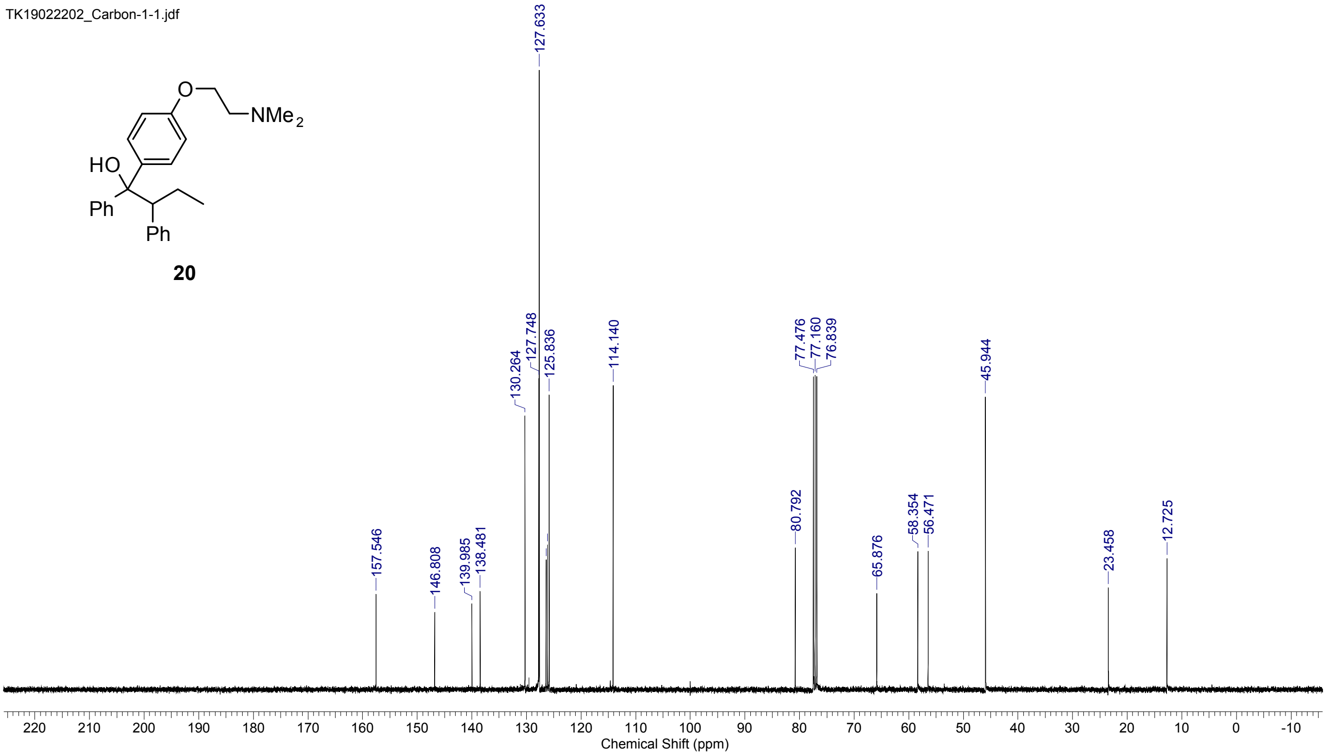

2019/06/28 15:42:33

Acquisition Time (sec)	3.5561	Comment	STANDARD 1H OBSERVE			Date	Jun 28 2019
Date Stamp	Jun 28 2019	File Name	¥¥Mac¥iCloud¥ [illegible]¥NMR_[illegible]¥2019NMR_K¥2019NMR(agilent)¥koba¥SK19062802.fid¥fid				
Frequency (MHz)	300.05	Nucleus	1H	Number of Transients	2	Original Points Count	17080
Points Count	65536	Pulse Sequence	s2pul	Receiver Gain	16.00	Solvent	CHLOROFORM-d
Spectrum Offset (Hz)	1698.8182	Spectrum Type	STANDARD	Sweep Width (Hz)	4803.07	Temperature (degree C)	AMBIENT TEMPERATURE

SK19062802

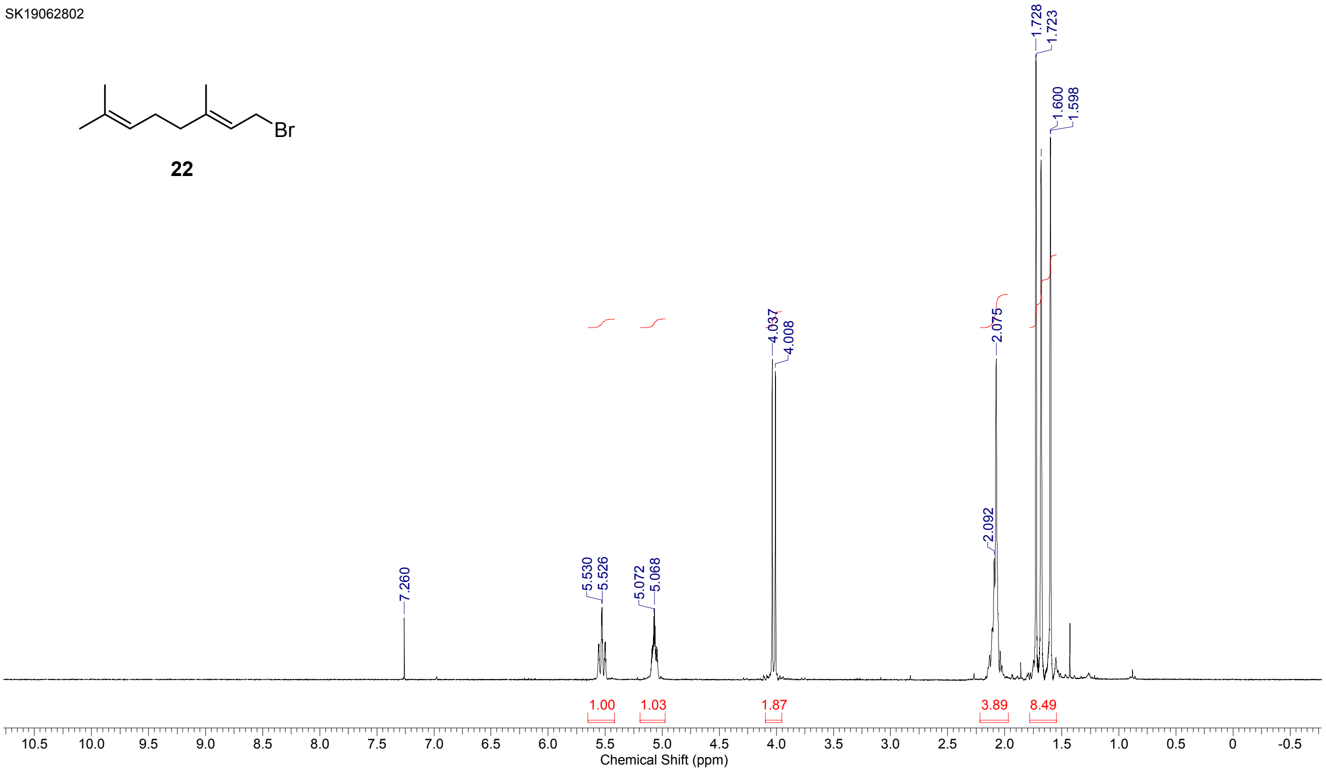

2019/06/28 15:45:50

Acquisition Time (sec)	0.8527	Comment	c13-NMR	Date	Jun 28 2019	Date Stamp	Jun 28 2019
File Name	¥¥Mac¥iCloud¥ [illegible]¥NMR_[illegible]¥2019NMR_K¥2019NMR(agilent)¥koba¥SK19062802_C.fid¥fid					Frequency (MHz)	75.46
Nucleus	13C	Number of Transients	256	Original Points Count	19335	Points Count	65536
Pulse Sequence	s2pul	Receiver Gain	30.00	Solvent	CHLOROFORM-d	Spectrum Offset (Hz)	8957.2949
Spectrum Type	STANDARD	Sweep Width (Hz)	22675.74	Temperature (degree C)	AMBIENT TEMPERATURE		

SK19062802_C

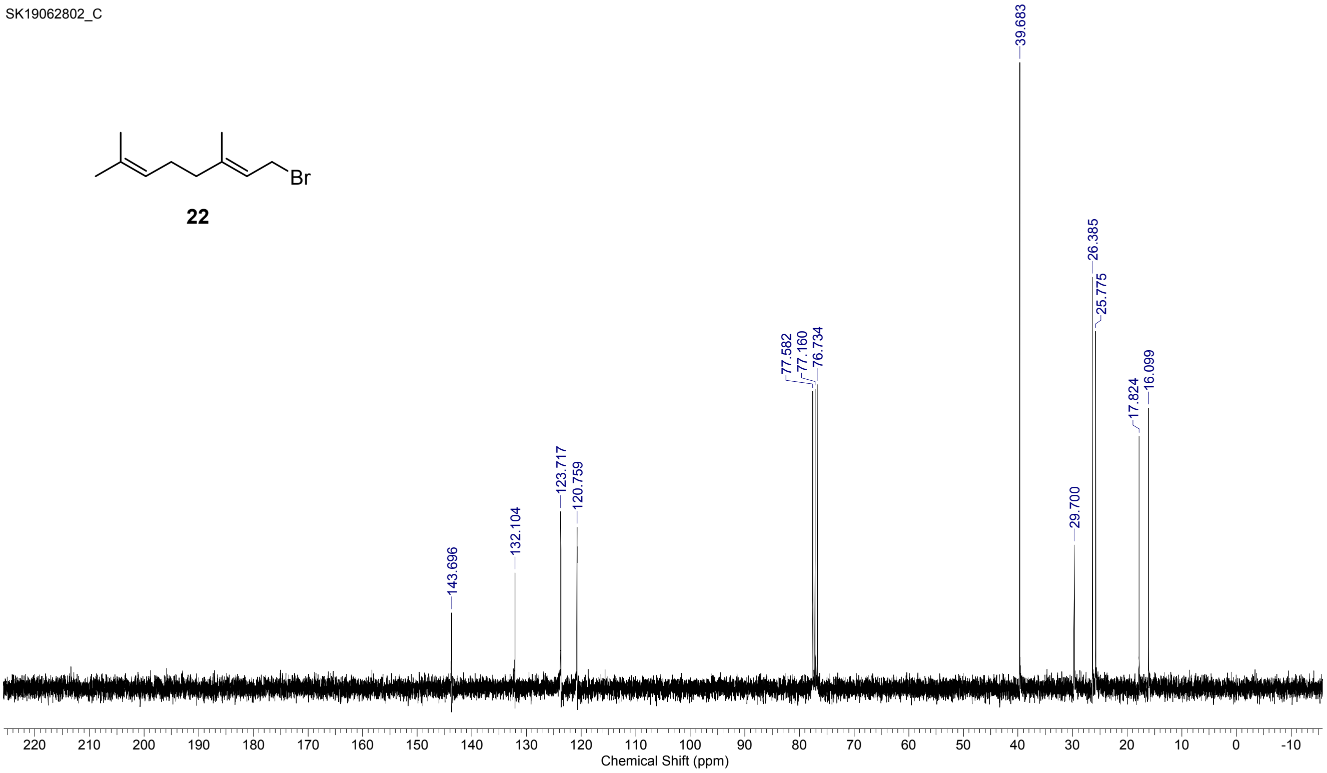

2019/03/06 19:08:41

Acquisition Time (sec)	2.1863	*Comment*	single_pulse	*Date*	17_Jul_2018 08:14:45		
Date Stamp	17_Jul_2018 08:13:54						
File Name	¥¥Mac¥iCloud¥[illegible]¥NMR [illegible]¥2018NMR_K¥2018NMR(JEOL)¥[illegible]NMR2018¥TK18071701_Proton−1−1.jdf						
Frequency (MHz)	399.78	*Nucleus*	1H	*Number of Transients*	8	*Origin*	ECA
Original Points Count	16384	*Owner*	delta	*Points Count*	32768	*Pulse Sequence*	proton.jxp
Solvent	CHLOROFORM-d	*Spectrum Offset (Hz)*	2003.7192	*Sweep Width (Hz)*	7494.00	*Temperature (degree C)*	24.300

TK18071701_Proton-1-1.jdf

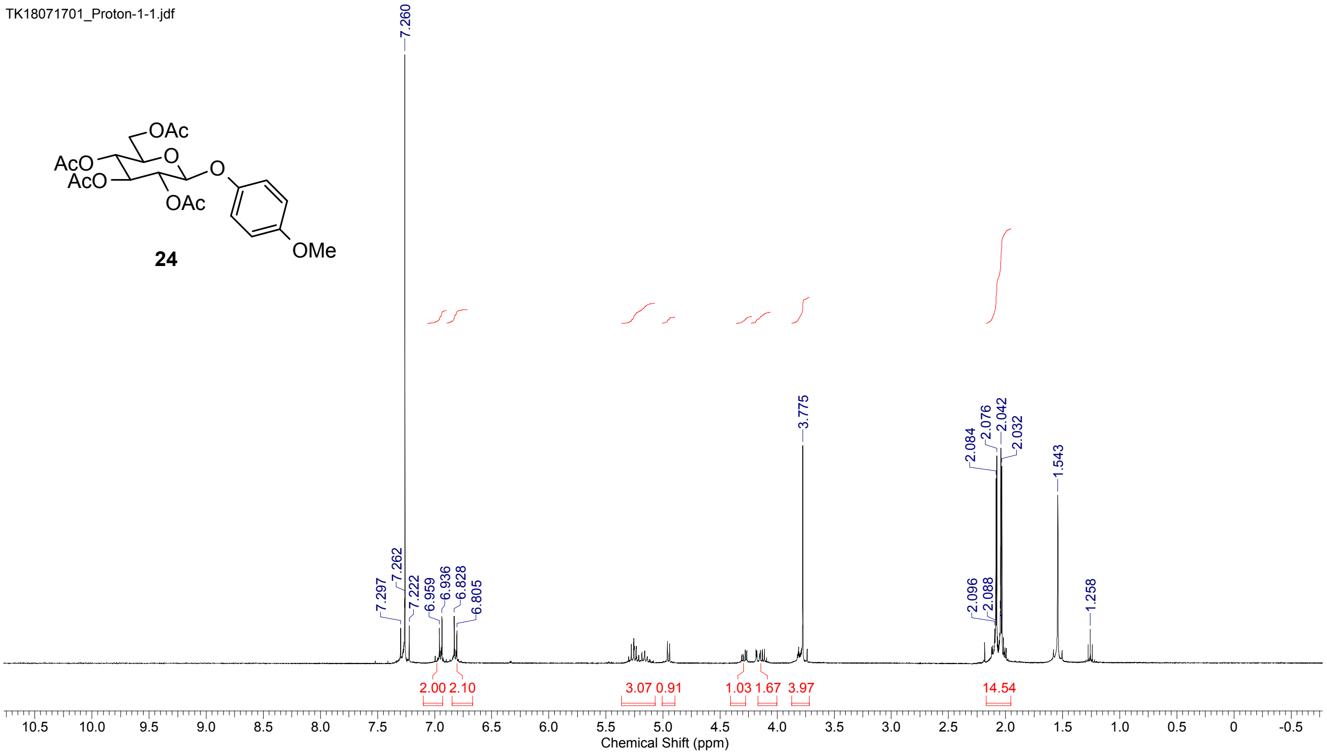

2019/05/14 17:09:50

Acquisition Time (sec)	0.8527	*Comment*	c13-NMR	*Date*	May 14 2019	*Date Stamp*	May 14 2019
File Name	¥¥Mac¥iCloud¥[illegible]¥NMR [illegible]¥2019NMR_K¥2019NMR(agilent)¥koshishiba¥MK19041103_C.fid¥fid					*Frequency (MHz)*	75.46
Nucleus	13C	*Number of Transients*	256	*Original Points Count*	19335	*Points Count*	65536
Pulse Sequence	s2pul	*Receiver Gain*	30.00	*Solvent*	CHLOROFORM-d	*Spectrum Offset (Hz)*	8954.8730
Spectrum Type	STANDARD	*Sweep Width (Hz)*	22675.74	*Temperature (degree C)*	AMBIENT TEMPERATURE		

MK19041103_C

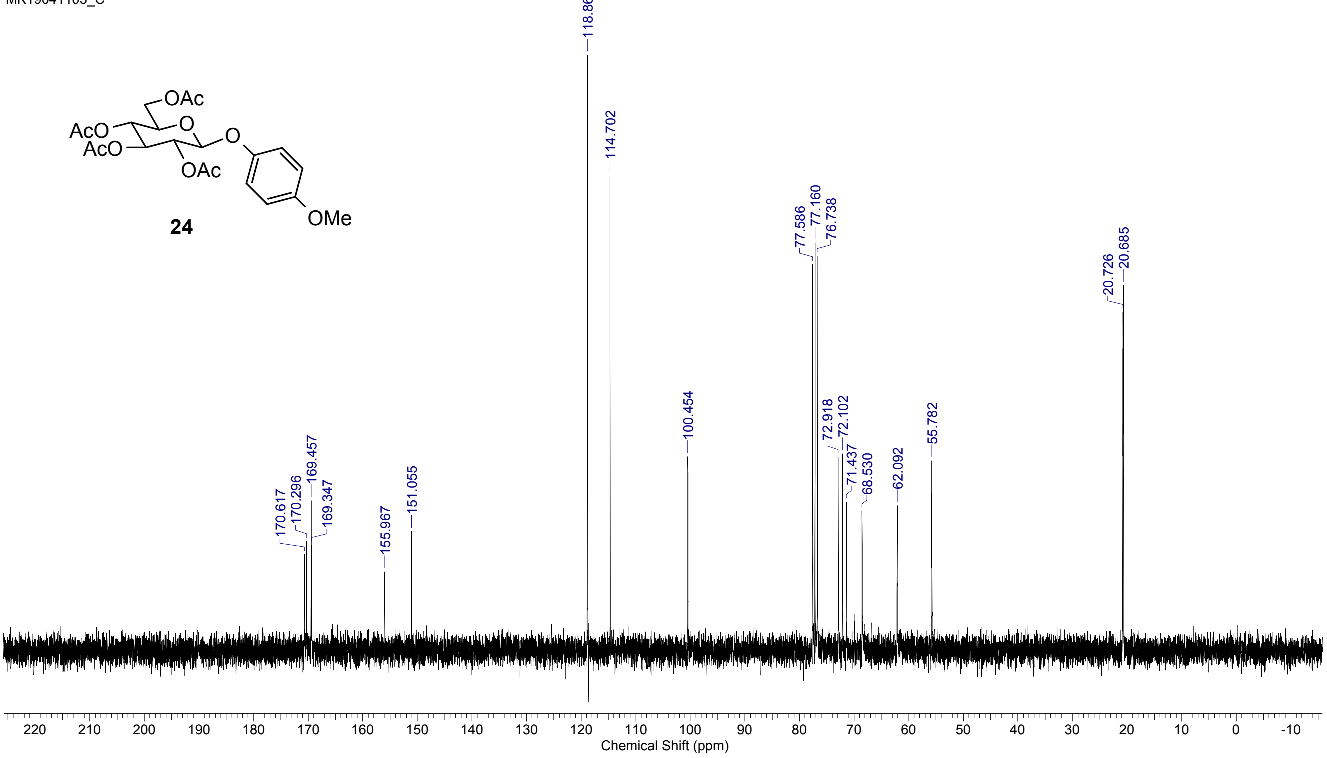

2019/03/04 19:50:01

Acquisition Time (sec)	3.5561	Comment	STANDARD 1H OBSERVE			Date	Jul 12 2014
Date Stamp	Jul 12 2014	File Name	¥¥.PSF¥.Home¥Documents¥[illegible]¥NMR[illegible]¥2014NMR¥yasumoto¥14071201.fid¥fid				
Frequency (MHz)	300.06	Nucleus	1H	Number of Transients	16	Original Points Count	17080
Points Count	65536	Pulse Sequence	s2pul	Receiver Gain	16.00	Solvent	CHLOROFORM-d
Spectrum Offset (Hz)	1698.6199	Spectrum Type	STANDARD	Sweep Width (Hz)	4803.07	Temperature (degree C)	AMBIENT TEMPERATURE

14071201.esp

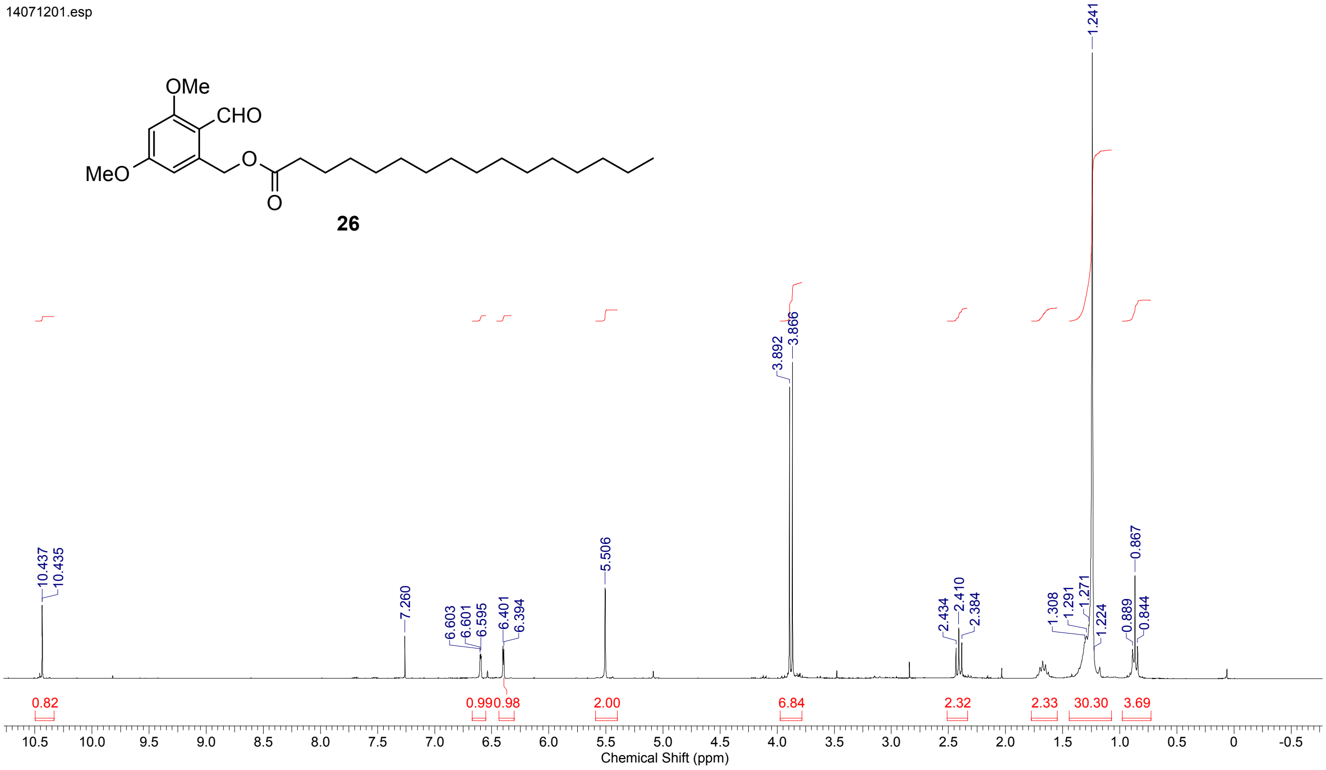

2019/03/04 19:54:04

Acquisition Time (sec)	0.8527	Comment	c13-NMR	Date	Jul 12 2014	Date Stamp	Jul 12 2014
File Name	¥¥.PSF¥.Home¥Documents¥[illegible]¥NMR[illegible]¥2014NMR¥yasumoto¥14071201_C.fid¥fid					Frequency (MHz)	75.46
Nucleus	13C	Number of Transients	736	Original Points Count	19335	Points Count	65536
Pulse Sequence	s2pul	Receiver Gain	30.00	Solvent	CHLOROFORM-d		
Spectrum Offset (Hz)	8954.5850	Spectrum Type	STANDARD	Sweep Width (Hz)	22675.74	Temperature (degree C)	AMBIENT TEMPERATURE

14071201_C.esp

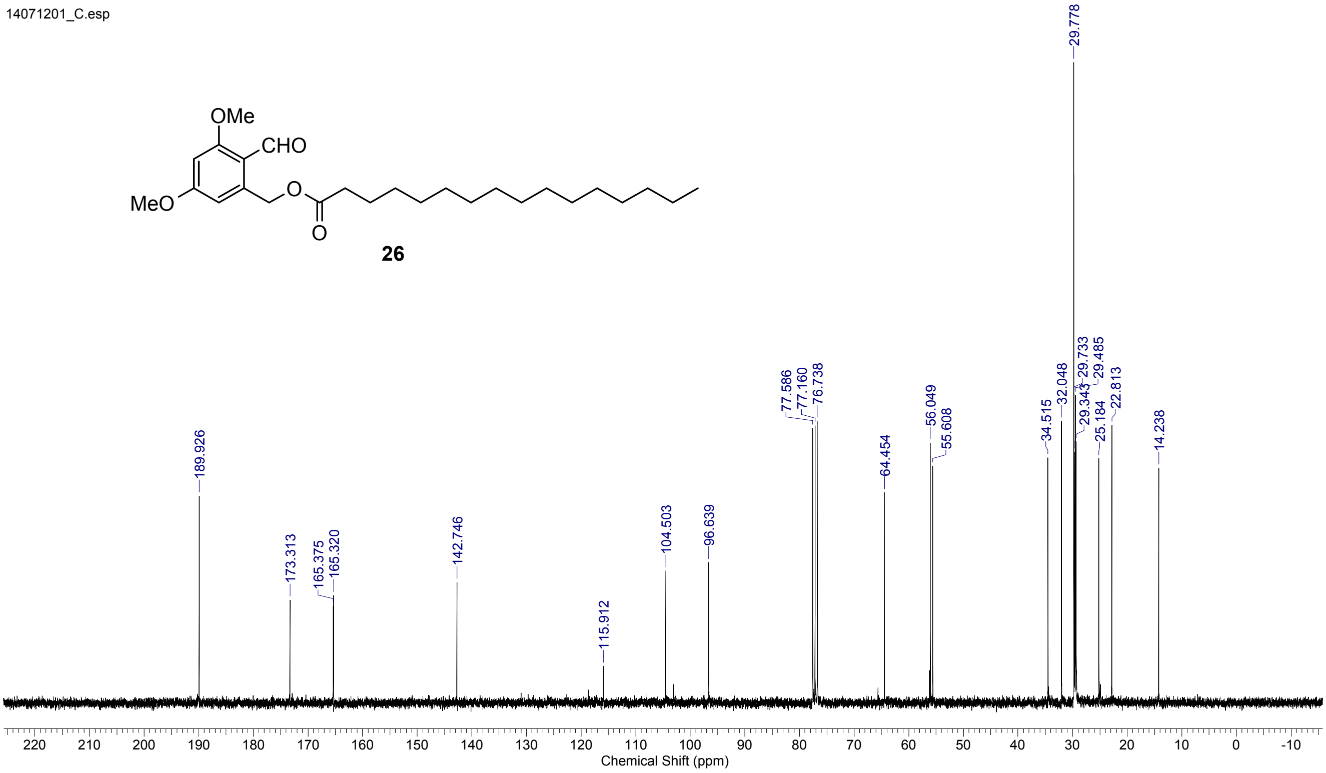

2019/03/06 20:14:04

Acquisition Time (sec)	2.1863	Comment	single pulse	Date	10 Dec 2018 08:00:26		
Date Stamp	10 Dec 2018 07:59:35						
File Name	¥¥Mac¥iCloud¥[illegible]NMR[illegible]¥2018NMR_K¥2018NMR(JEOL)¥[illegible]NMR2018¥TTK18121001_Proton-1-1.jdf						
Frequency (MHz)	399.78	Nucleus	1H	Number of Transients	8	Origin	ECA
Original Points Count	16384	Owner	delta	Points Count	32768	Pulse Sequence	proton.jxp
Solvent	CHLOROFORM-d	Spectrum Offset (Hz)	2003.9476	Sweep Width (Hz)	7494.00	Temperature (degree C)	13.800

TTK18121001_Proton-1-1.jdf

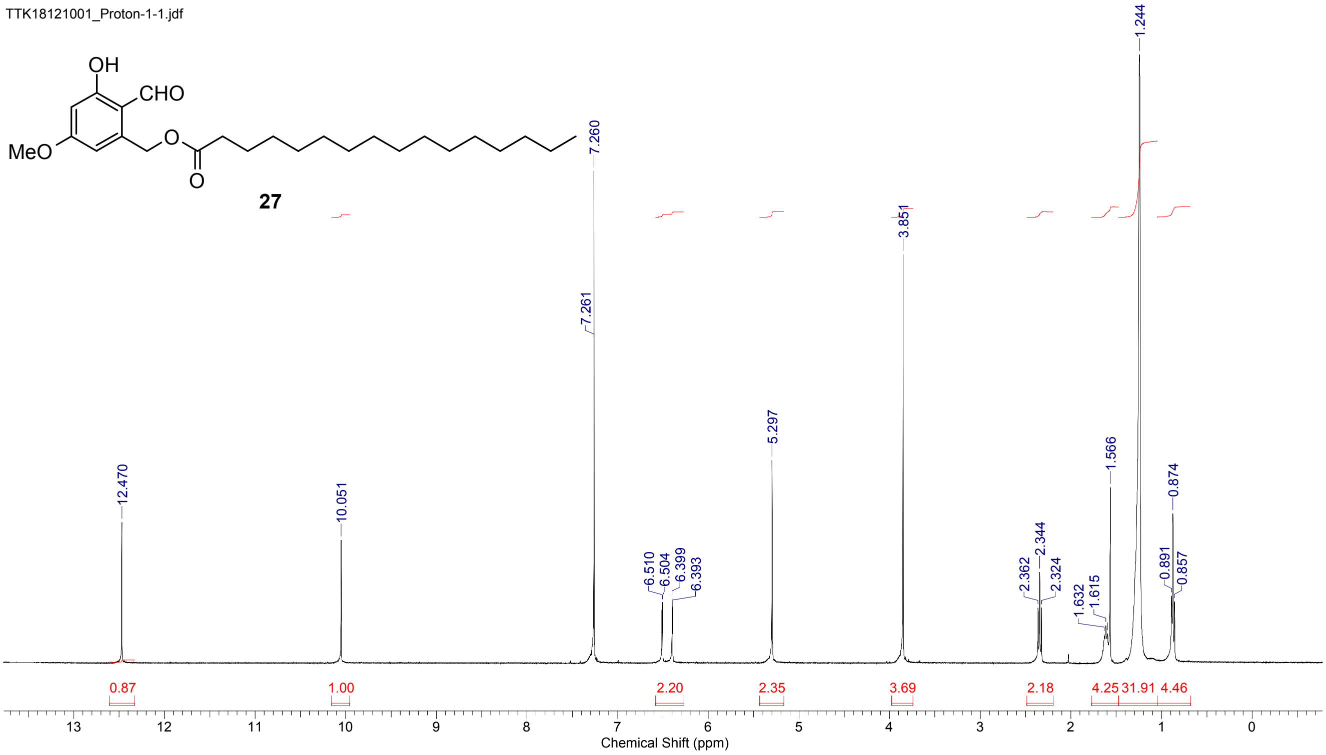

2019/06/28 15:52:20

Acquisition Time (sec)	0.8527	Comment	c13-NMR	Date	Jun 28 2019	Date Stamp	Jun 28 2019
File Name	¥¥Mac¥iCloud¥[illegible]NMR[illegible]¥2019NMR_K¥2019NMR(agilent)¥koba¥SK19062801_C.fid¥fid					Frequency (MHz)	75.46
Nucleus	13C	Number of Transients	832	Original Points Count	19335	Points Count	65536
Pulse Sequence	s2pul	Receiver Gain	30.00	Solvent	CHLOROFORM-d	Spectrum Offset (Hz)	8958.3340
Spectrum Type	STANDARD	Sweep Width (Hz)	22675.74	Temperature (degree C)	AMBIENT TEMPERATURE		

SK19062801_C

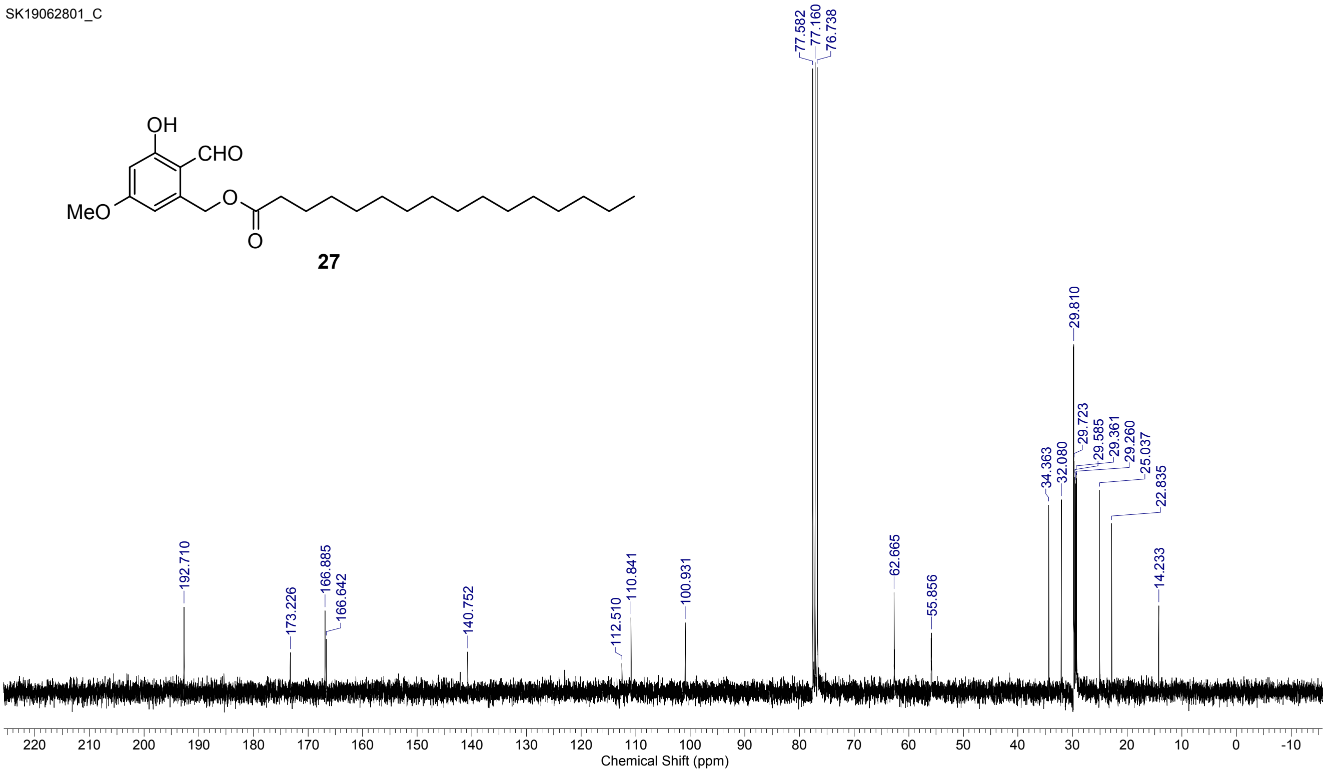

2019/03/05 18:55:51

Acquisition Time (sec)	2.1863	Comment	single_pulse	Date	02 Nov 2017 12:17:42		
Date Stamp	02 Nov 2017 12:16:51						
File Name	¥¥Mac¥iCloud¥[illegible]NMR[illegible]¥2017NMR_K¥2017NMR(JEOL)¥[illegible]¥TT17110201_Proton-1-1.jdf					Frequency (MHz)	399.78
Nucleus	1H	Number of Transients	8	Origin	ECA	Original Points Count	16384
Owner	delta	Points Count	32768	Pulse Sequence	proton.jxp	Solvent	CHLOROFORM-d
Spectrum Offset (Hz)	2003.4905	Sweep Width (Hz)	7494.00	Temperature (degree C)	18.700		

TT17110201_Proton-1-1.jdf

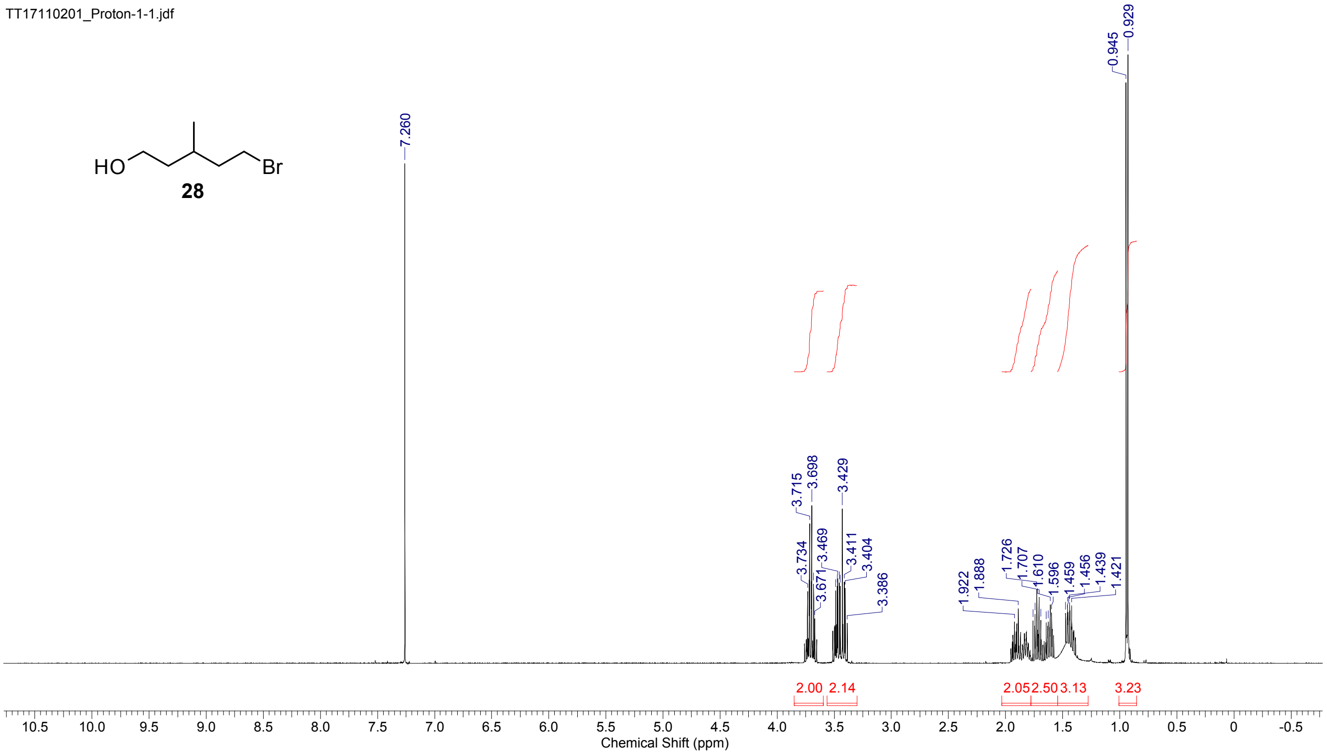

2019/03/12 12:14:16

Acquisition Time (sec)	0.8527	Comment	c13-NMR	Date	Mar 12 2019	Date Stamp	Mar 12 2019		
File Name	¥¥Mac¥iCloud¥[illegible]NMR[illegible]¥2019NMR_K¥koba¥SK19031201_C.fid¥fid					Frequency (MHz)	75.46	Nucleus	13C
Number of Transients	192	Original Points Count	19335	Points Count	65536	Pulse Sequence	s2pul	Receiver Gain	30.00
Solvent	CHLOROFORM-d			Spectrum Offset (Hz)	8953.8359	Spectrum Type	STANDARD	Sweep Width (Hz)	22675.74
Temperature (degree C)	AMBIENT TEMPERATURE								

SK19031201_C

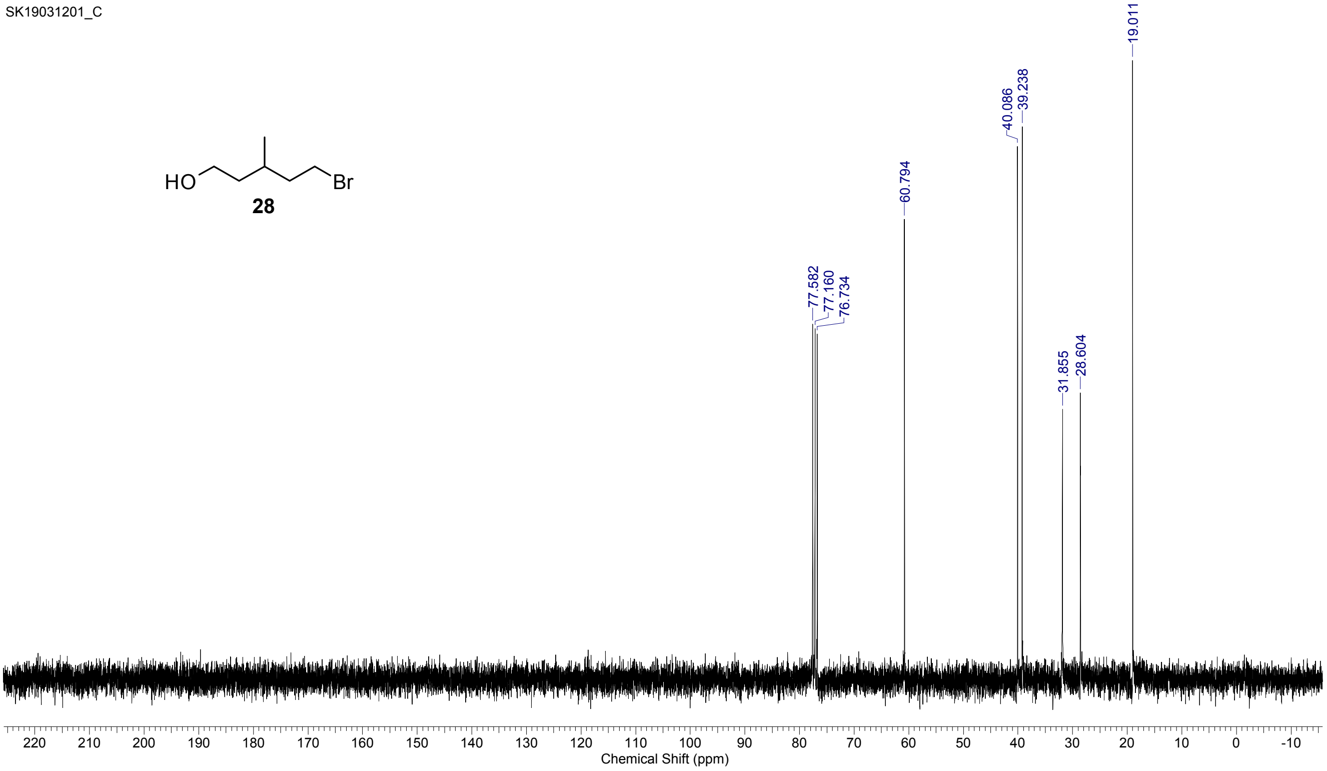

Acquisition Time (sec)	3.5561	*Comment*	STANDARD 1H OBSERVE			*Date*	Jul 31 2019
Date Stamp	Jul 31 2019	*File Name*	¥¥Mac¥iCloud¥[illegible]¥NMR [illegible]¥2019NMR_K¥2019NMR(agilent)¥kuroda¥YK19073101.fid¥fid				
Frequency (MHz)	300.05	*Nucleus*	1H	*Number of Transients*	16	*Original Points Count*	17080
Points Count	65536	*Pulse Sequence*	s2pul	*Receiver Gain*	34.00	*Solvent*	pyridine
Spectrum Offset (Hz)	1605.9590	*Spectrum Type*	STANDARD	*Sweep Width (Hz)*	4803.07	*Temperature (degree C)*	AMBIENT TEMPERATURE

YK19073101

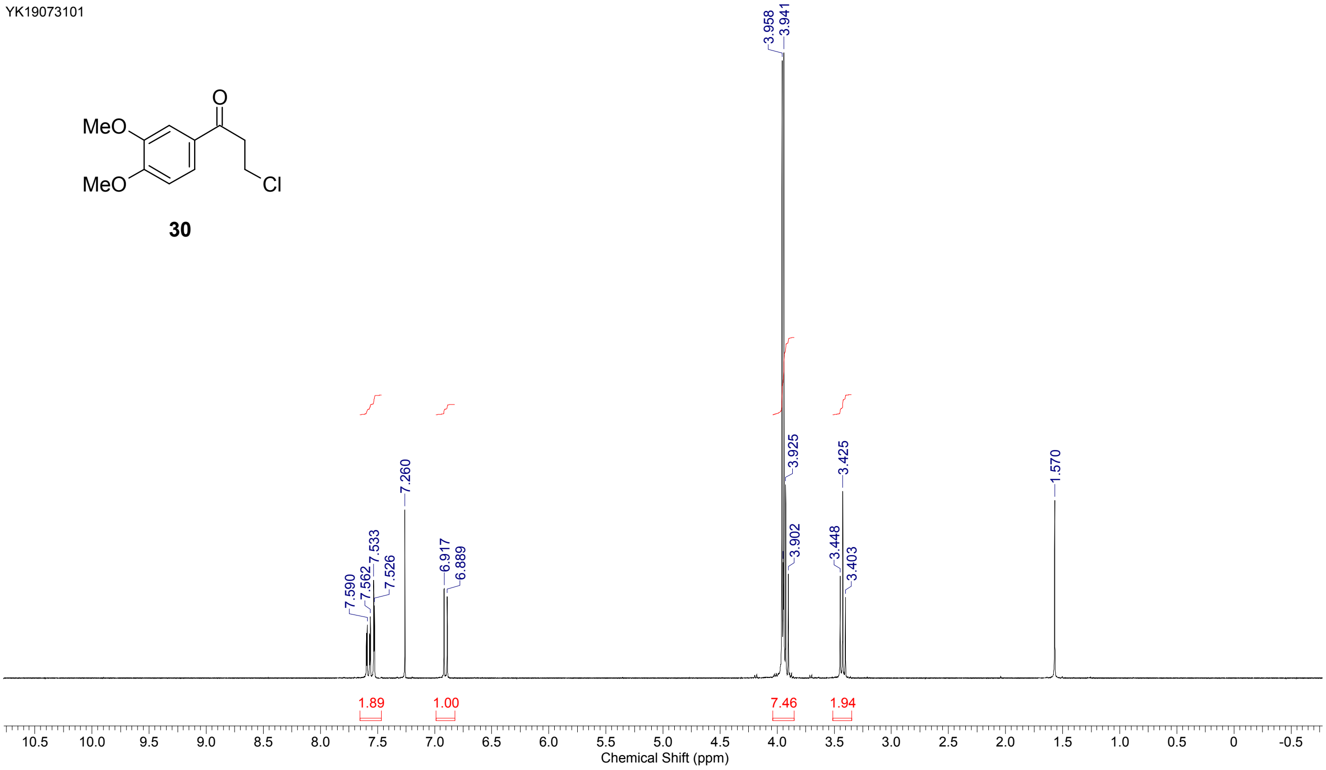

Acquisition Time (sec)	0.8527	*Comment*	c13-NMR	*Date*	Aug 1 2019	*Date Stamp*	Aug 1 2019
File Name	¥¥Mac¥iCloud¥[illegible]¥NMR [illegible]¥2019NMR_K¥2019NMR(agilent)¥kuroda¥YK19080101_C.fid¥fid					*Frequency (MHz)*	75.46
Nucleus	13C	*Number of Transients*	640	*Original Points Count*	19335	*Points Count*	65536
Pulse Sequence	s2pul	*Receiver Gain*	30.00	*Solvent*	CHLOROFORM-d	*Spectrum Offset (Hz)*	8955.5654
Spectrum Type	STANDARD	*Sweep Width (Hz)*	22675.74	*Temperature (degree C)*	AMBIENT TEMPERATURE		

YK19080101_C

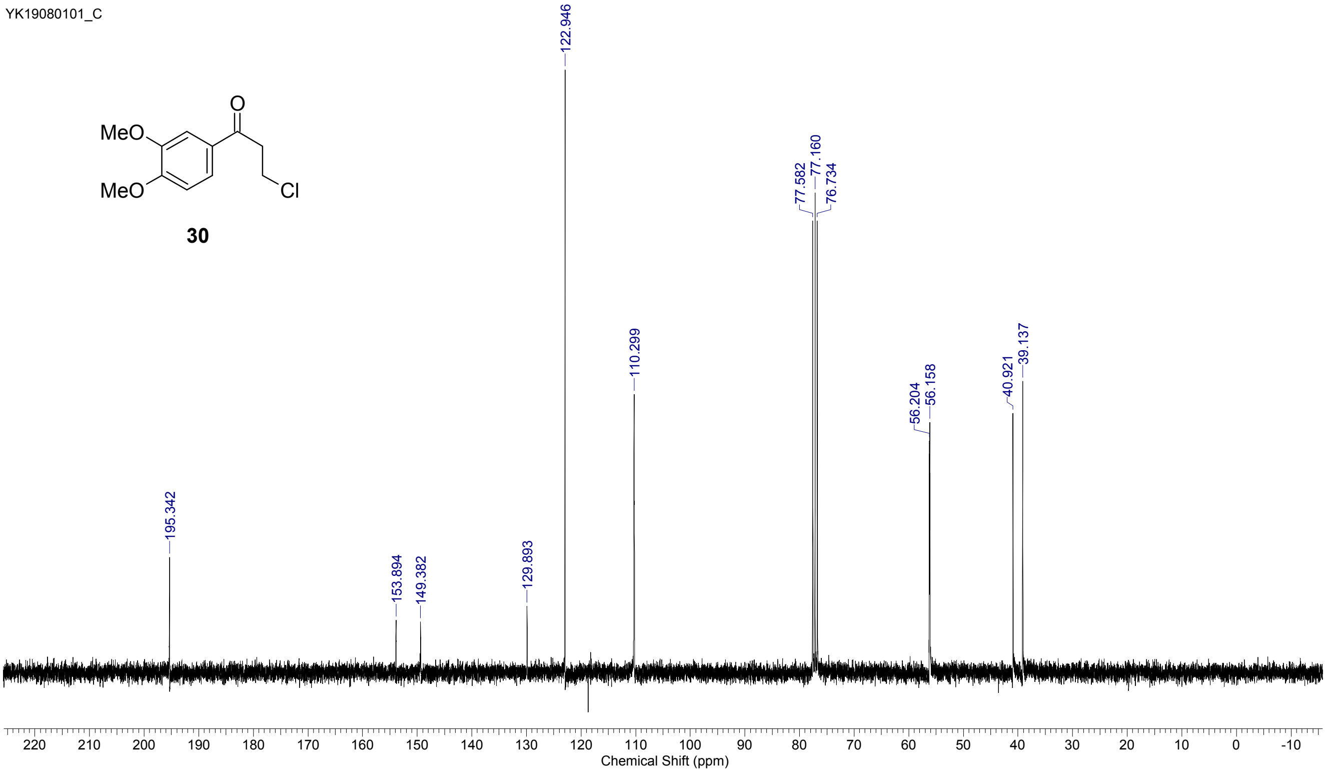

2019/03/06 20:23:46

Acquisition Time (sec)	2.1863	Comment	single pulse	Date	24 Oct 2018 09:43:15		
Date Stamp	24 Oct 2018 09:42:23						
File Name	¥¥Mac¥iCloud¥ [illegible]NMR [illegible]¥2018NMR_K¥2018NMR(JEOL)¥ [illegible]NMR2018¥TK18102402_Proton-1-1.jdf						
Frequency (MHz)	399.78	Nucleus	1H	Number of Transients	8	Origin	ECA
Original Points Count	16384	Owner	delta	Points Count	32768	Pulse Sequence	proton.jxp
Solvent	CHLOROFORM-d	Spectrum Offset (Hz)	2003.4905	Sweep Width (Hz)	7494.00	Temperature (degree C)	18.900

TK18102402_Proton-1-1.jdf

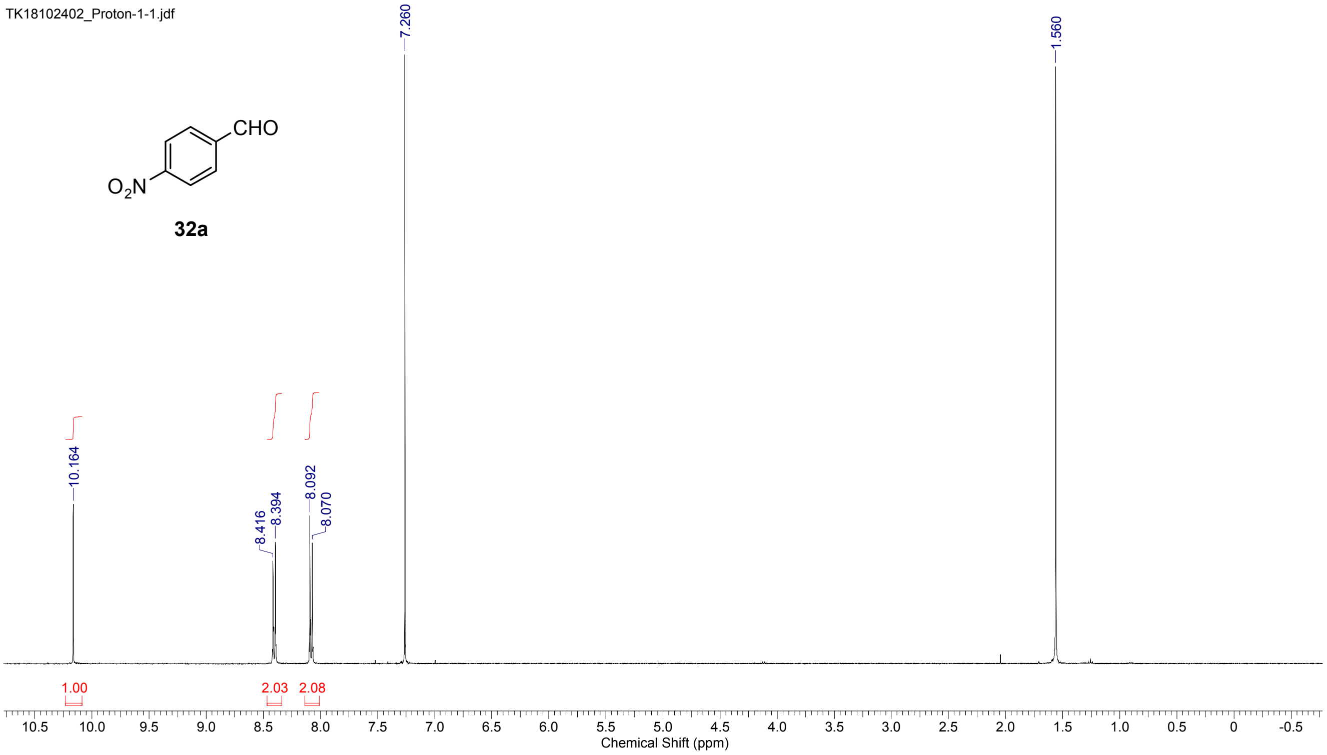

2019/03/13 20:13:54

Acquisition Time (sec)	0.8527	Comment	c13-NMR	Date	Mar 13 2019	Date Stamp	Mar 13 2019		
File Name	¥¥Mac¥iCloud¥ [illegible]NMR [illegible]¥2019NMR_K¥koba¥SK19031301_C.fid¥fid					Frequency (MHz)	75.46	Nucleus	13C
Number of Transients	672	Original Points Count	19335	Points Count	65536	Pulse Sequence	s2pul	Receiver Gain	30.00
Solvent	CHLOROFORM-d			Spectrum Offset (Hz)	8956.6035	Spectrum Type	STANDARD	Sweep Width (Hz)	22675.74
Temperature (degree C)	AMBIENT TEMPERATURE								

SK19031301_C

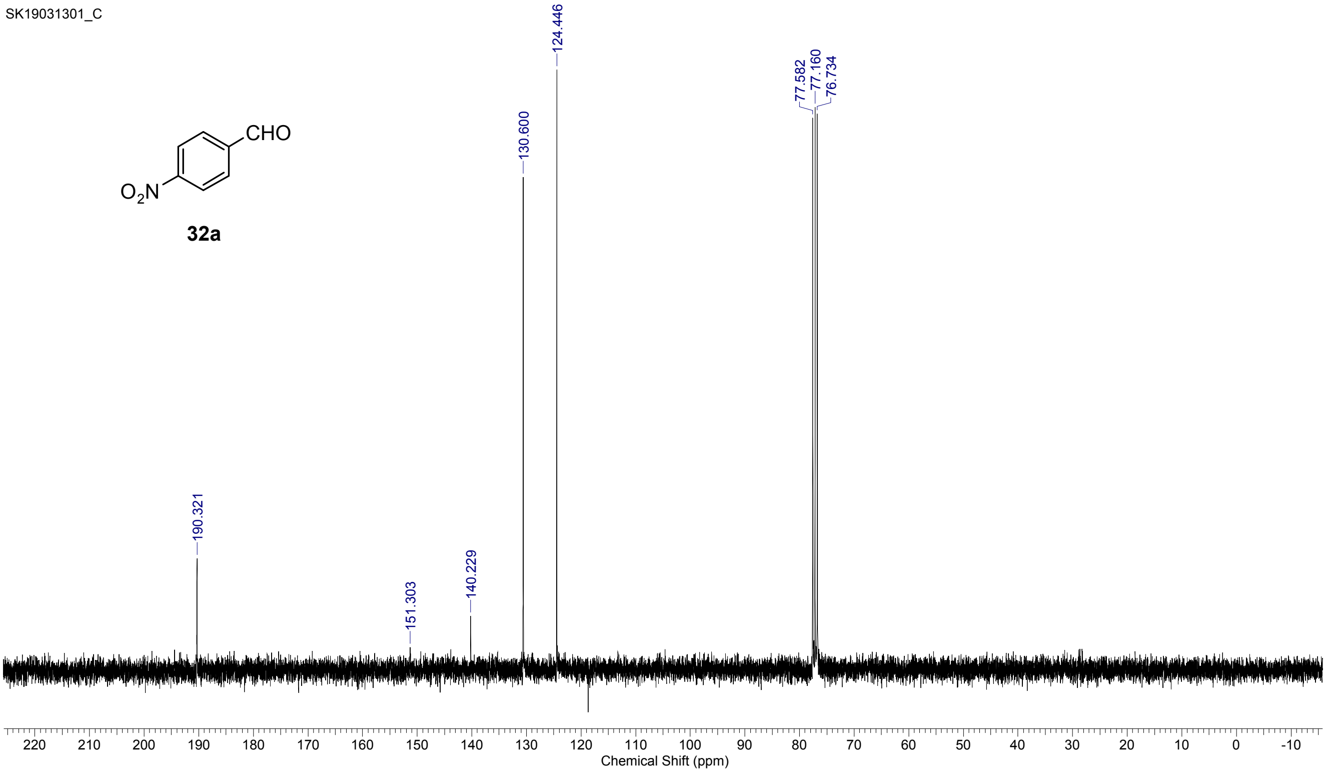

2019/03/07 14:48:08

Acquisition Time (sec)	2.1863	***Comment***	single pulse	***Date***	16 Oct 2018 08:24:46		
Date Stamp	16 Oct 2018 08:23:55						
File Name	¥¥Mac¥iCloud¥ [illegible]¥NMR [illegible]¥2018NMR K¥2018NMR(JEOL)¥ [illegible]NMR2018¥TK18101603_Proton-1-1.jdf						
Frequency (MHz)	399.78	***Nucleus***	1H	***Number of Transients***	8	***Origin***	ECA
Original Points Count	16384	***Owner***	delta	***Points Count***	32768	***Pulse Sequence***	proton.jxp
Solvent	CHLOROFORM-d	***Spectrum Offset (Hz)***	2003.4905	***Sweep Width (Hz)***	7494.00	***Temperature (degree C)***	17.700

TK18101603_Proton-1-1.jdf

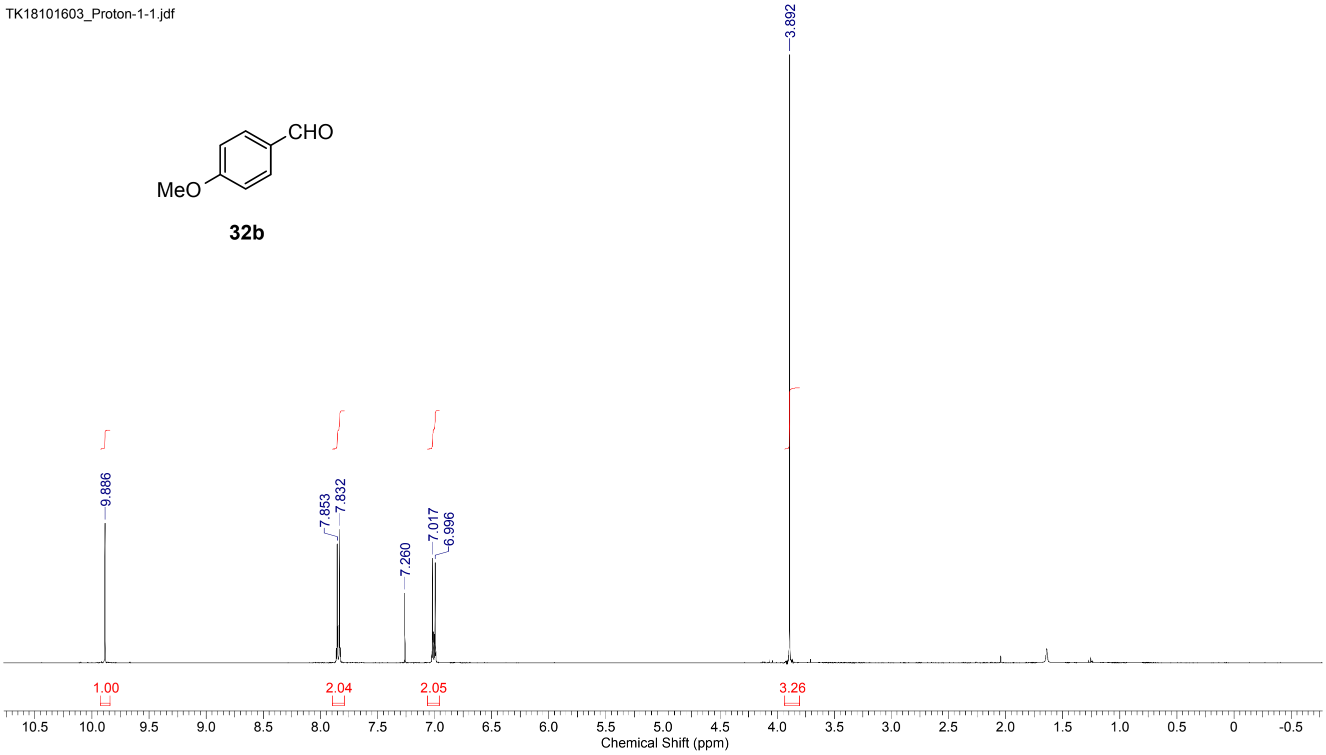

2019/03/07 14:50:46

Acquisition Time (sec)	1.0381	***Comment***	single pulse decoupled gated NOE	***Date***	01 Mar 2019 09:33:38		
Date Stamp	01 Mar 2019 09:23:36						
File Name	¥¥Mac¥iCloud¥ [illegible]¥NMR [illegible]¥2018NMR K¥2018NMR(JEOL)¥ [illegible]NMR2018¥TK19022802_Carbon-1-1.jdf						
Frequency (MHz)	100.53	***Nucleus***	13C	***Number of Transients***	256	***Origin***	ECA
Original Points Count	32768	***Owner***	delta	***Points Count***	65536	***Pulse Sequence***	carbon.jxp
Solvent	CHLOROFORM-d	***Spectrum Offset (Hz)***	10047.0723	***Sweep Width (Hz)***	31565.66	***Temperature (degree C)***	21.400

TK19022802_Carbon-1-1.jdf

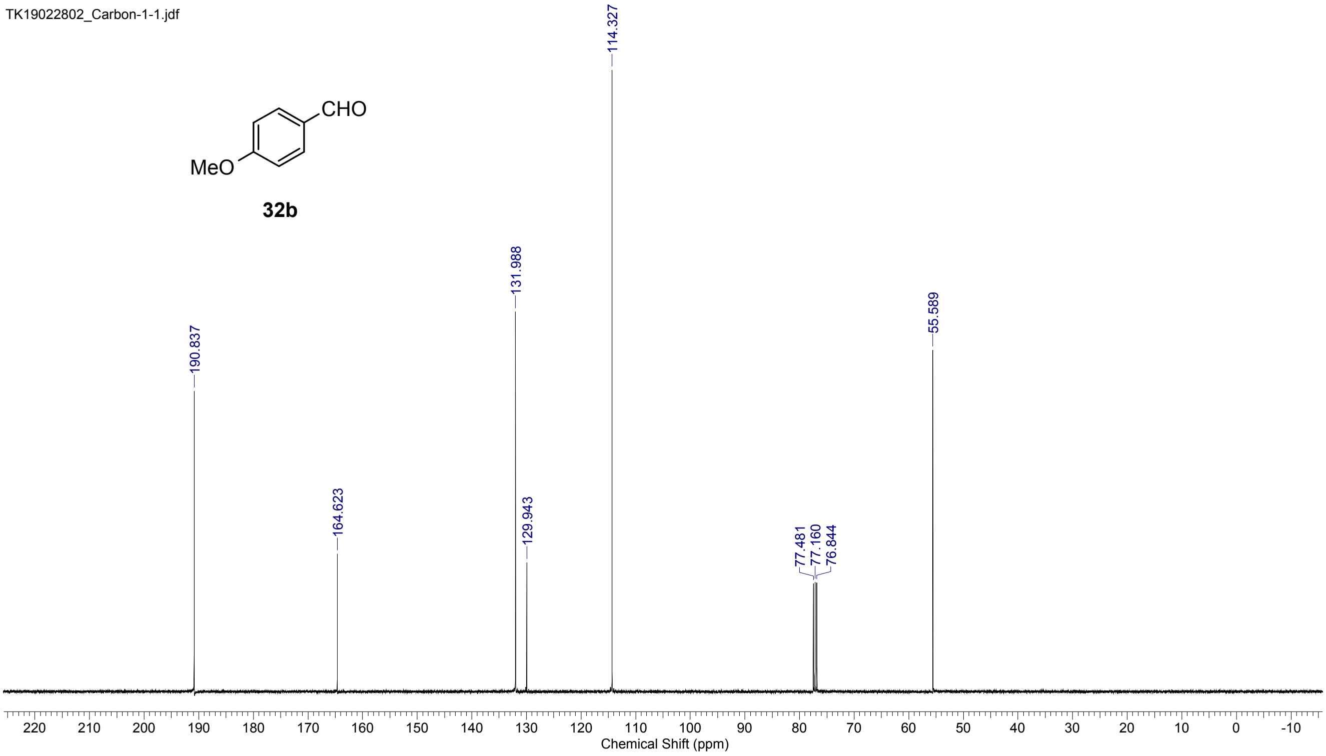

2019/03/07 16:29:29

Acquisition Time (sec)	3.5561	*Comment*	STANDARD 1H OBSERVE			*Date*	Aug 8 2012
Date Stamp	Aug 8 2012	*File Name*	¥¥Mac¥iCloud¥ [illegible]NMR [illegible]¥2012NMR_K¥saka¥12080801.fid¥fid				
Frequency (MHz)	300.06	*Nucleus*	1H	*Number of Transients*	16	*Original Points Count*	17080
Points Count	65536	*Pulse Sequence*	s2pul	*Receiver Gain*	16.00	*Solvent*	CHLOROFORM-d
Spectrum Offset (Hz)	1752.1255	*Spectrum Type*	STANDARD	*Sweep Width (Hz)*	4803.07	*Temperature (degree C)*	AMBIENT TEMPERATURE

36-H(12080801).esp

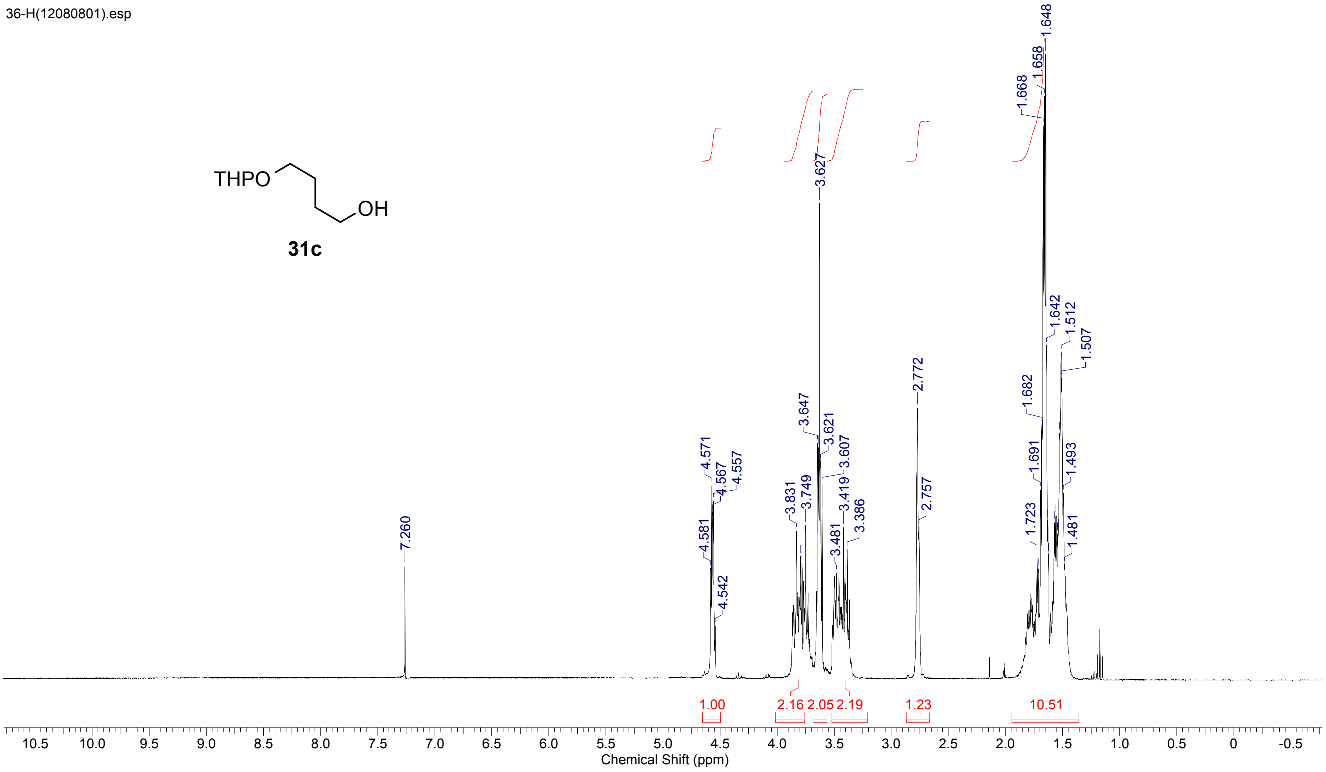

2019/03/07 16:38:11

Acquisition Time (sec)	0.8527	*Comment*	c13-NMR	*Date*	Aug 8 2012	*Date Stamp*	Aug 8 2012		
File Name	¥¥Mac¥iCloud¥ [illegible]NMR [illegible]¥2012NMR_K¥saka¥12080801_C.fid¥fid					*Frequency (MHz)*	75.46	*Nucleus*	13C
Number of Transients	96	*Original Points Count*	19335	*Points Count*	65536	*Pulse Sequence*	s2pul	*Receiver Gain*	30.00
Solvent	CHLOROFORM-d			*Spectrum Offset (Hz)*	8966.3594	*Spectrum Type*	STANDARD	*Sweep Width (Hz)*	22675.74
Temperature (degree C)	AMBIENT TEMPERATURE								

12080801_C

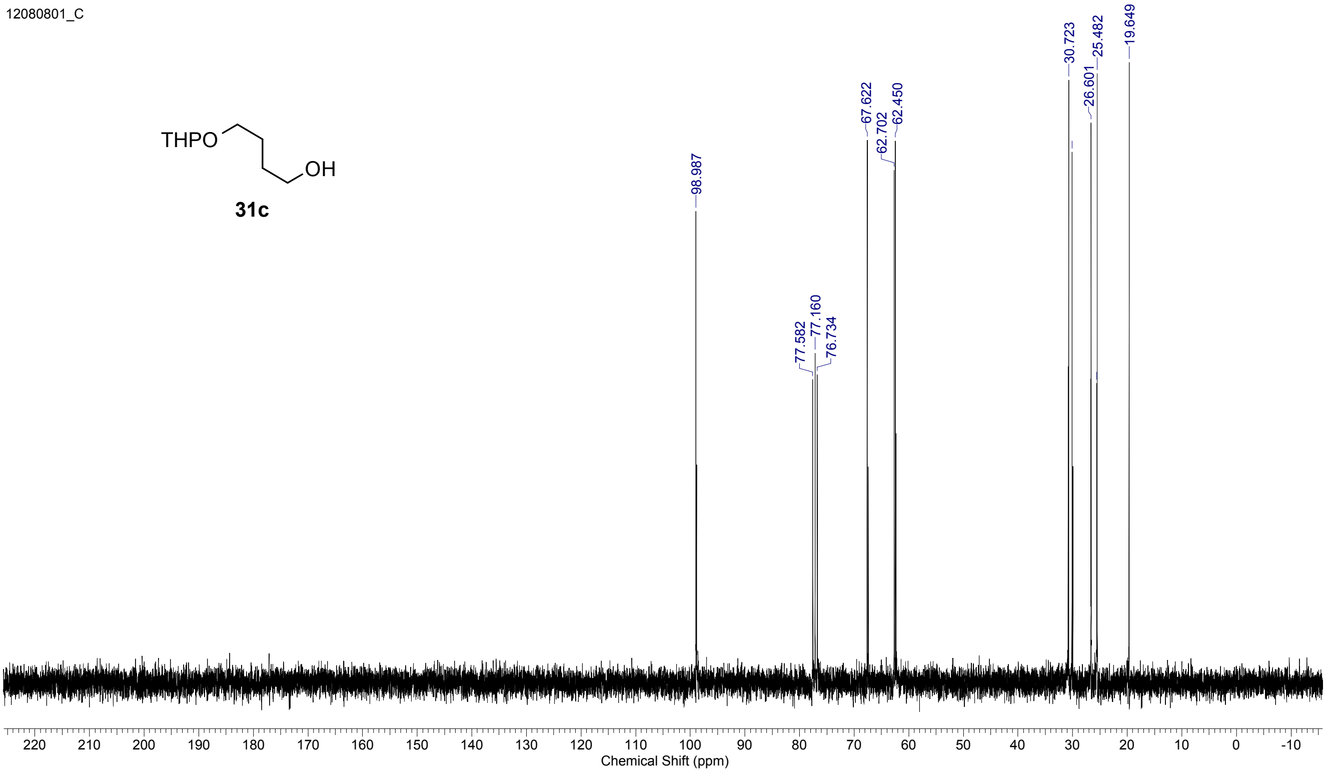

2019/03/07 15:02:26

Acquisition Time (sec)	3.5561	Comment	STANDARD 1H OBSERVE			Date	Apr 23 2012
Date Stamp	Apr 23 2012	File Name	¥¥Mac¥iCloud¥[illegible]NMR[illegible]¥2012NMR_K¥saka¥12042301.fid¥fid				
Frequency (MHz)	300.06	Nucleus	1H	Number of Transients	16	Original Points Count	17080
Points Count	65536	Pulse Sequence	s2pul	Receiver Gain	24.00	Solvent	CHLOROFORM-d
Spectrum Offset (Hz)	1767.8837	Spectrum Type	STANDARD	Sweep Width (Hz)	4803.07	Temperature (degree C)	AMBIENT TEMPERATURE

12042301

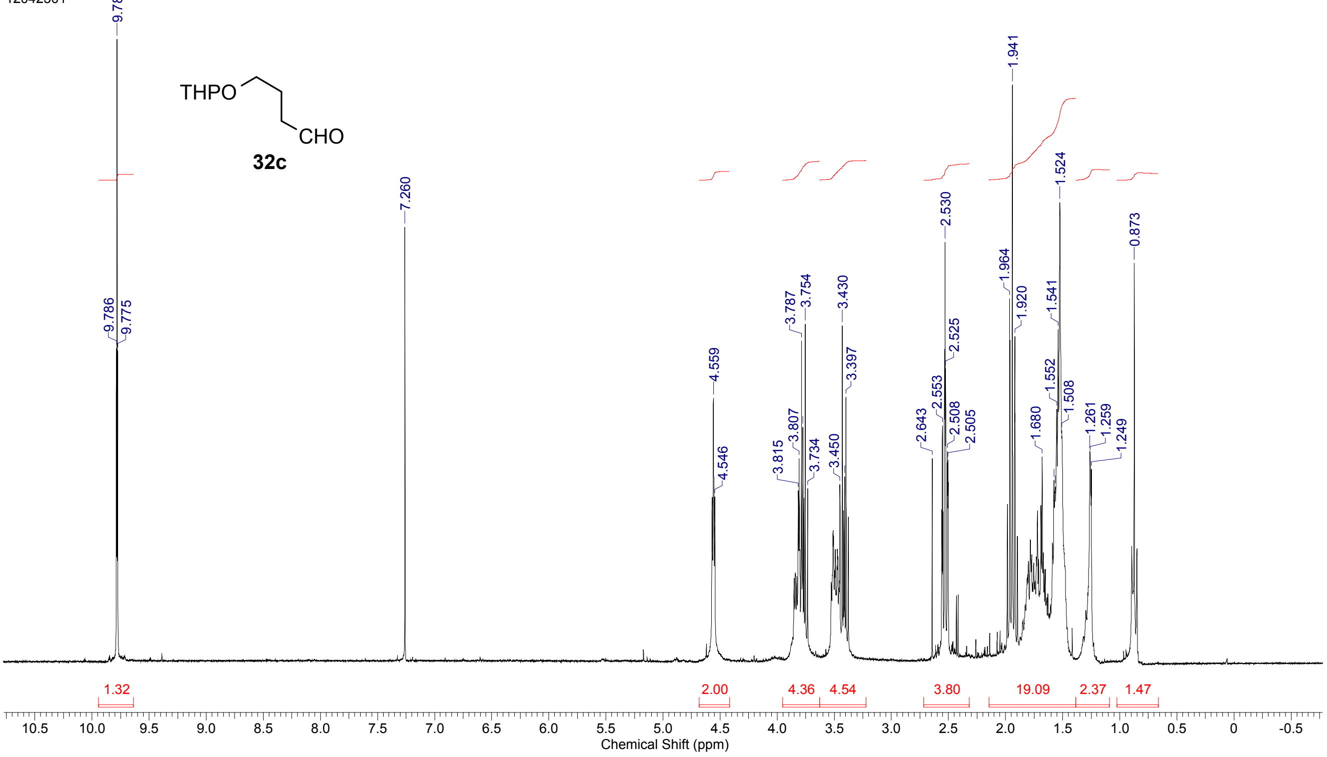

2019/03/07 17:19:56

Acquisition Time (sec)	0.8527	Comment	c13-NMR	Date	Jul 11 2012	Date Stamp	Jul 11 2012		
File Name	¥¥Mac¥iCloud¥[illegible]NMR[illegible]¥2012NMR_K¥saka¥12071102_C.fid¥fid					Frequency (MHz)	75.46	Nucleus	13C
Number of Transients	64	Original Points Count	19335	Points Count	65536	Pulse Sequence	s2pul	Receiver Gain	30.00
Solvent	CHLOROFORM-d			Spectrum Offset (Hz)	8962.2070	Spectrum Type	STANDARD	Sweep Width (Hz)	22675.74
Temperature (degree C)	AMBIENT TEMPERATURE								

12071102_C

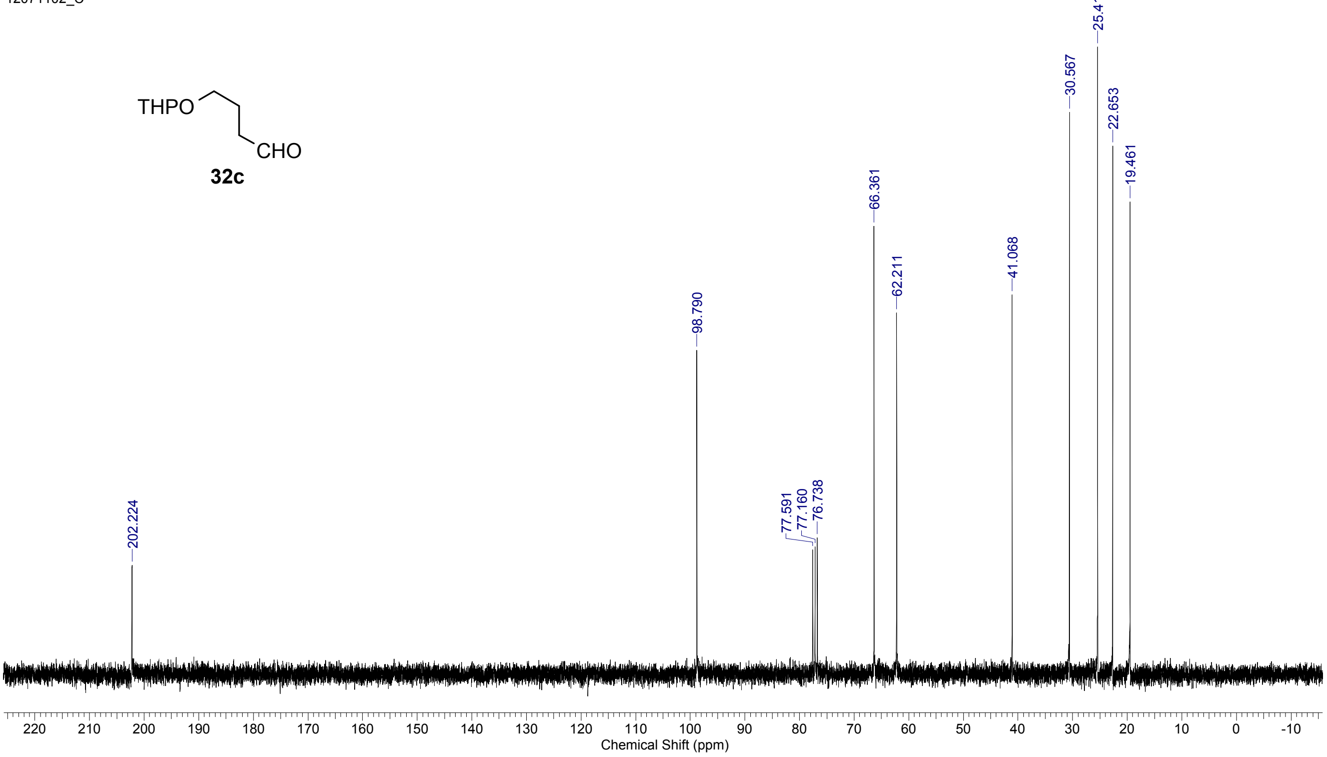

2019/03/07 18:13:26

Acquisition Time (sec)	2.1863	Comment	single_pulse	Date	05 Feb 2019 13:33:59		
Date Stamp	05 Feb 2019 13:33:08						
File Name	¥¥Mac¥iCloud¥[illegible]¥NMR_[illegible]¥2018NMR_K¥2018NMR(JEOL)¥[illegible]NMR2018¥TK19020501_Proton-1-1.jdf						
Frequency (MHz)	399.78	Nucleus	1H	Number of Transients	8	Origin	ECA
Original Points Count	16384	Owner	delta	Points Count	32768	Pulse Sequence	proton.jxp
Solvent	CHLOROFORM-d	Spectrum Offset (Hz)	2003.4905	Sweep Width (Hz)	7494.00	Temperature (degree C)	22.100

39-H(TK19020501_Proton-1-1).esp

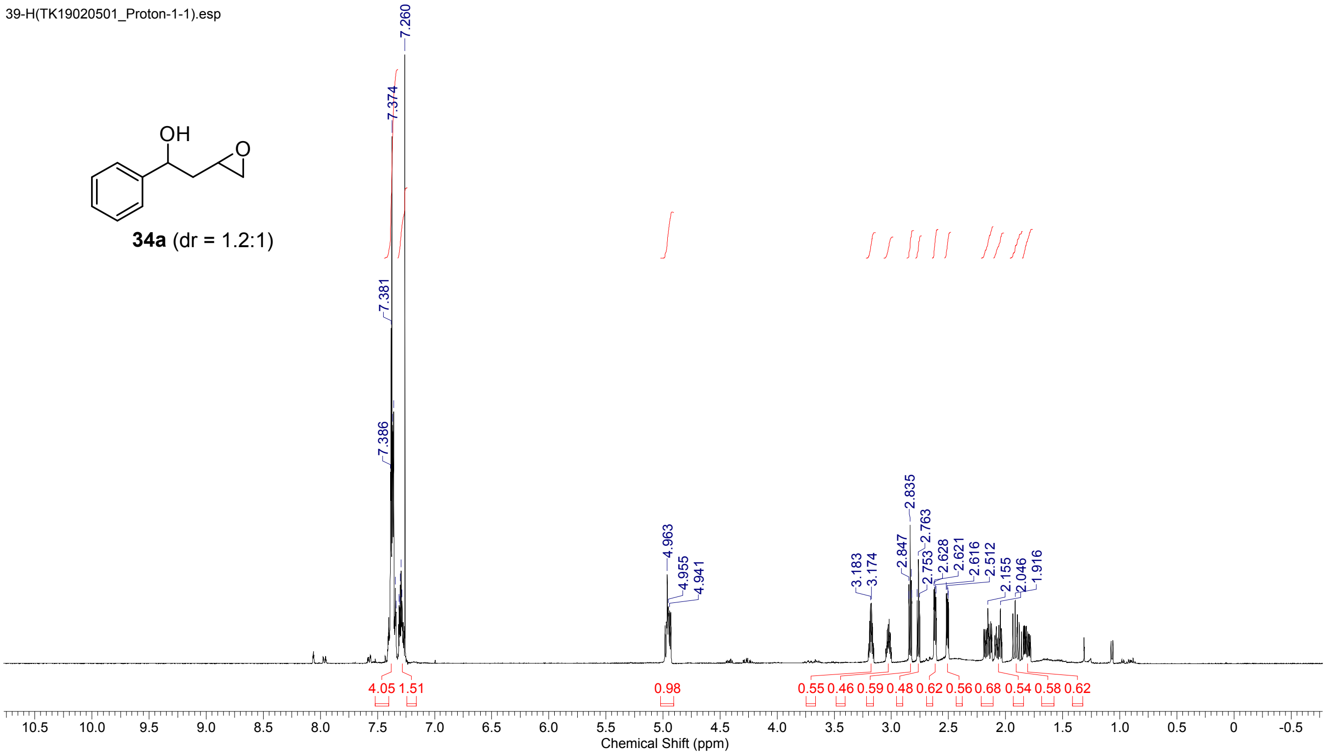

2019/03/07 18:28:29

Acquisition Time (sec)	0.8527	Comment	c13-NMR	Date	Feb 25 2019	Date Stamp	Feb 25 2019
File Name	¥¥Mac¥iCloud¥[illegible]¥NMR_[illegible]¥2018NMR_K¥2018NMR(agilent)¥kawakami¥TK19022503_C.fid¥fid					Frequency (MHz)	75.46
Nucleus	13C	Number of Transients	128	Original Points Count	19335	Points Count	65536
Pulse Sequence	s2pul	Receiver Gain	30.00	Solvent	CHLOROFORM-d	Spectrum Offset (Hz)	8949.3369
Spectrum Type	STANDARD	Sweep Width (Hz)	22675.74	Temperature (degree C)	AMBIENT TEMPERATURE		

TK19022503_C

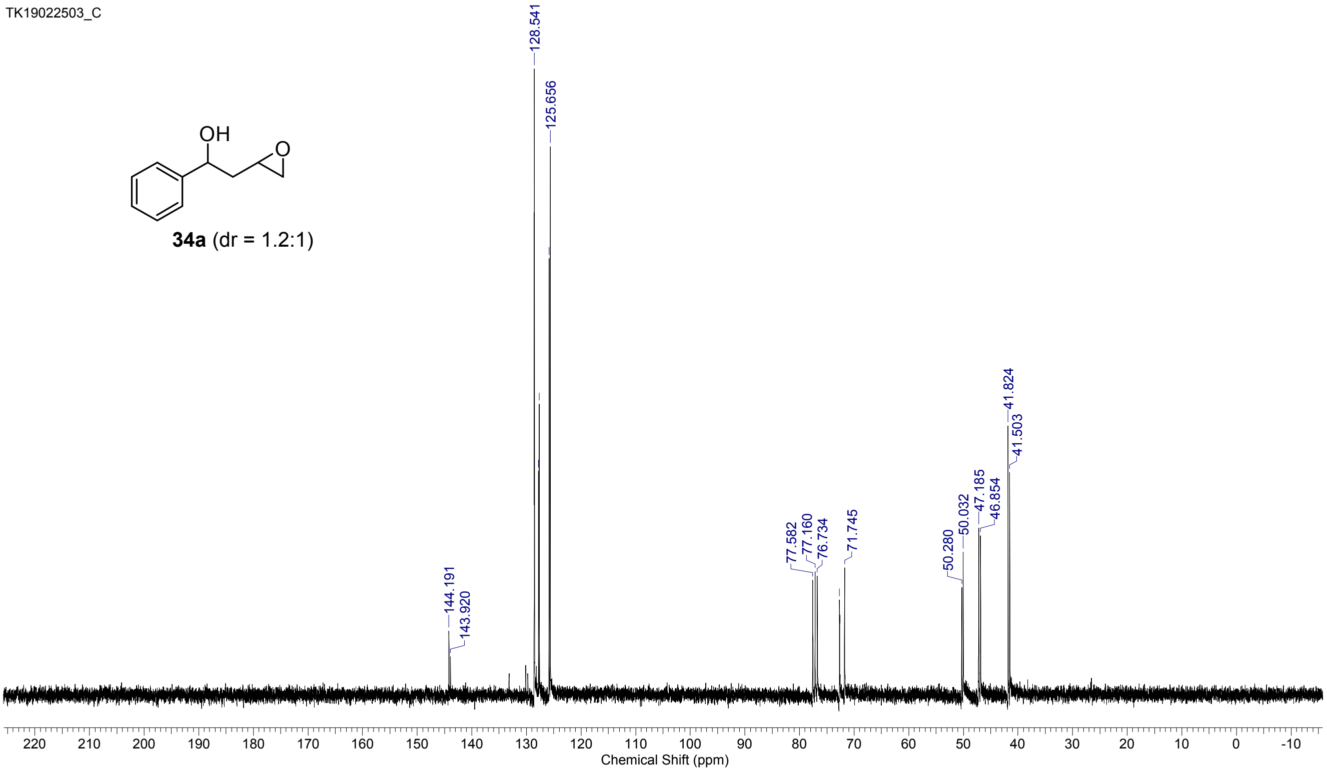

2019/05/20 17:34:35

Acquisition Time (sec)	2.1863	***Comment***	single_pulse	***Date***	11 Apr 2019 14:45:44			***Date Stamp***	11 Apr 2019 14:44:53
File Name	¥¥WS¥data¥[illegible]¥TT19041103_Proton-1-1.jdf			***Frequency (MHz)***	399.78	***Nucleus***	1H	***Number of Transients***	8
Origin	ECA	***Original Points Count***	16384	***Owner***	delta	***Points Count***	32768	***Pulse Sequence***	proton.jxp
Solvent	CHLOROFORM-d			***Spectrum Offset (Hz)***	2003.4905	***Sweep Width (Hz)***	7494.00	***Temperature (degree C)***	16.200

TT19041103_Proton-1-1.esp

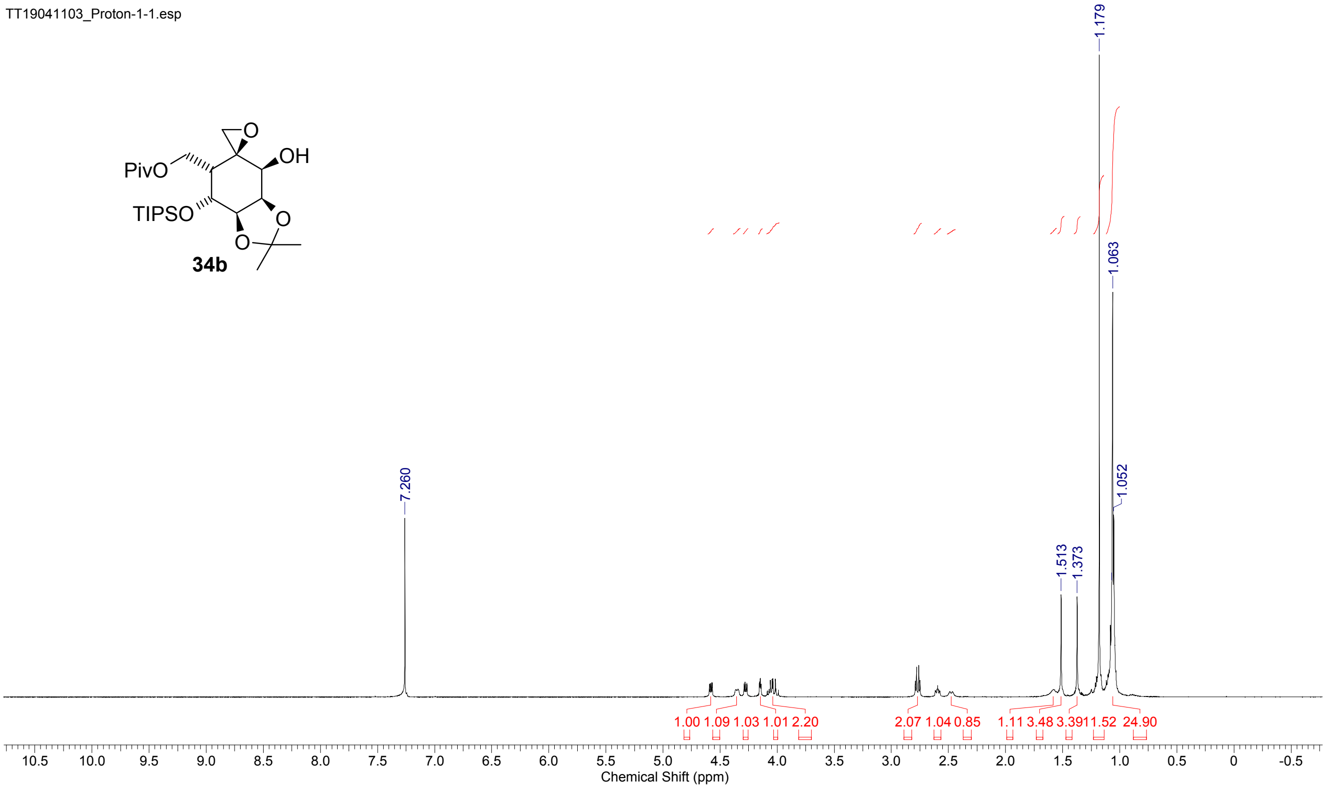

2019/05/20 19:38:10

Acquisition Time (sec)	1.0381	***Comment***	single pulse decoupled gated NOE			***Date***	12 Apr 2019 12:57:55		
Date Stamp	12 Apr 2019 12:18:23			***File Name***	¥¥WS¥data¥[illegible]¥TT19041201_Carbon-1-1.jdf			***Frequency (MHz)***	100.53
Nucleus	13C	***Number of Transients***	1024	***Origin***	ECA	***Original Points Count***	32768	***Owner***	delta
Points Count	65536	***Pulse Sequence***	carbon.jxp	***Solvent***	CHLOROFORM-d			***Spectrum Offset (Hz)***	10056.2236
Sweep Width (Hz)	31565.66	***Temperature (degree C)***	16.600						

TT19041201_Carbon-1-1.esp

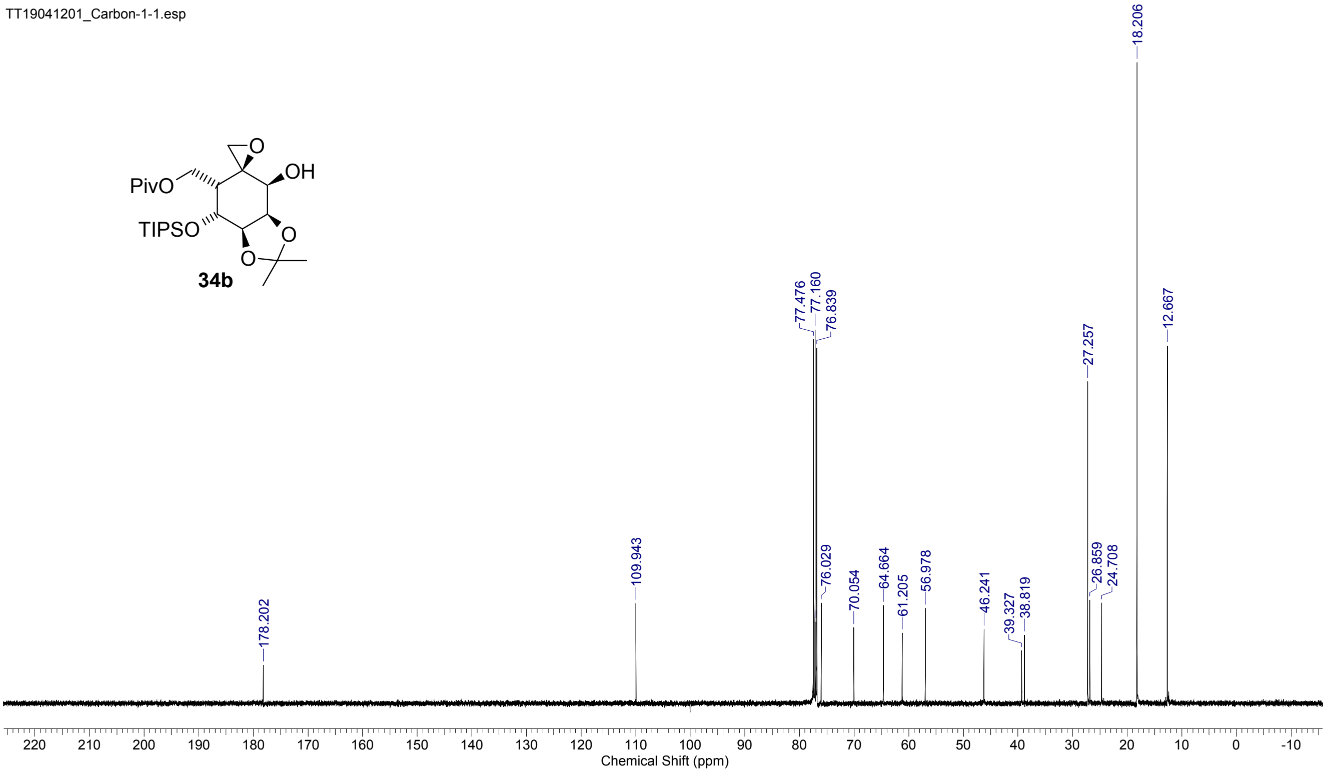

2019/03/11 18:10:06

Acquisition Time (sec)	2.1863	Comment	single pulse	Date	18 Feb 2019 12:01:30		
Date Stamp	18 Feb 2019 12:00:38						
File Name	¥¥Mac¥iCloud¥ [illegible]¥NMR [illegible]¥2018NMR_K¥2018NMR(JEOL)¥ [illegible]NMR2018¥TK19021801_Proton-1-1.jdf						
Frequency (MHz)	399.78	Nucleus	1H	Number of Transients	8	Origin	ECA
Original Points Count	16384	Owner	delta	Points Count	32768	Pulse Sequence	proton.jxp
Solvent	CHLOROFORM-d	Spectrum Offset (Hz)	2003.4905	Sweep Width (Hz)	7494.00	Temperature (degree C)	21.300

TK19021801_Proton-1-1.jdf

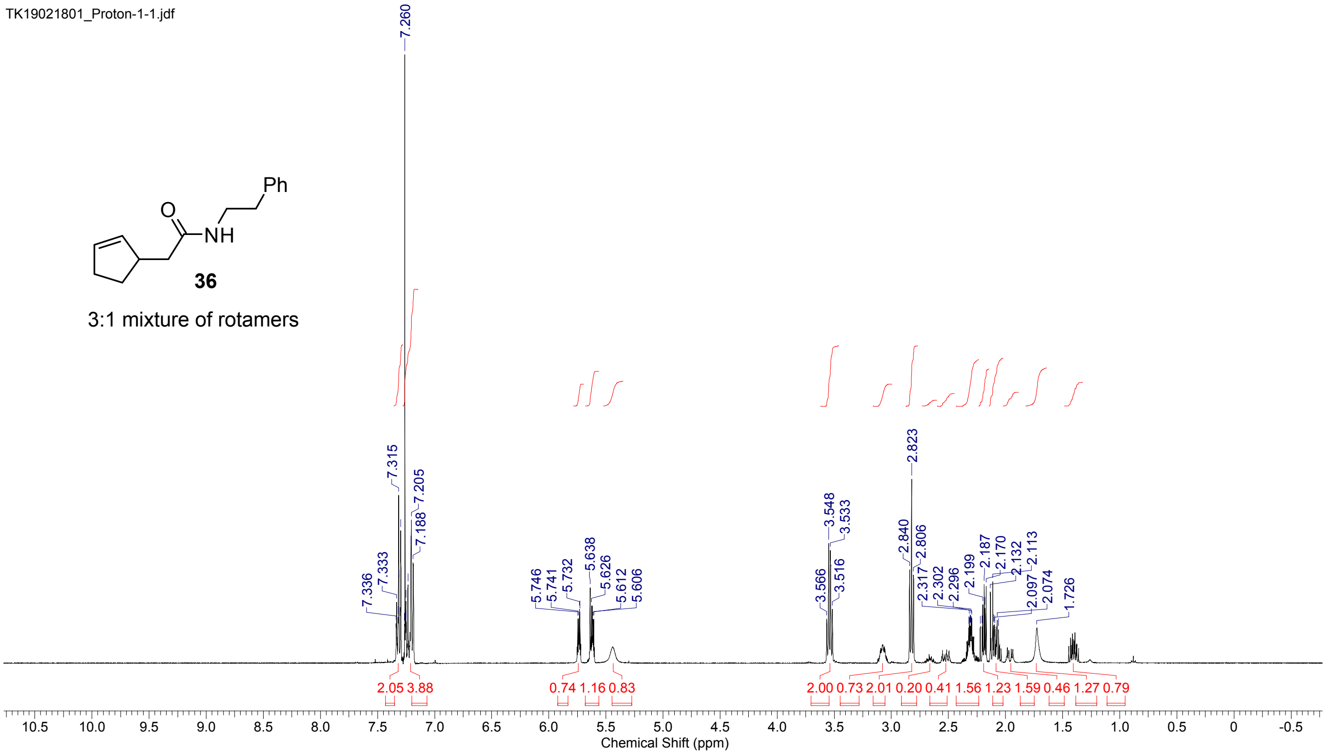

2019/03/11 18:39:28

Acquisition Time (sec)	1.0381	Comment	single pulse decoupled gated NOE	Date	02 Mar 2019 09:12:02		
Date Stamp	02 Mar 2019 08:52:10						
File Name	¥¥Mac¥iCloud¥ [illegible]¥NMR [illegible]¥2018NMR_K¥2018NMR(JEOL)¥ [illegible]NMR2018¥TK19030203_Carbon-1-1.jdf						
Frequency (MHz)	100.53	Nucleus	13C	Number of Transients	512	Origin	ECA
Original Points Count	32768	Owner	delta	Points Count	65536	Pulse Sequence	carbon.jxp
Solvent	CHLOROFORM-d	Spectrum Offset (Hz)	10038.8838	Sweep Width (Hz)	31565.66	Temperature (degree C)	20.500

TK19030203_Carbon-1-1.jdf

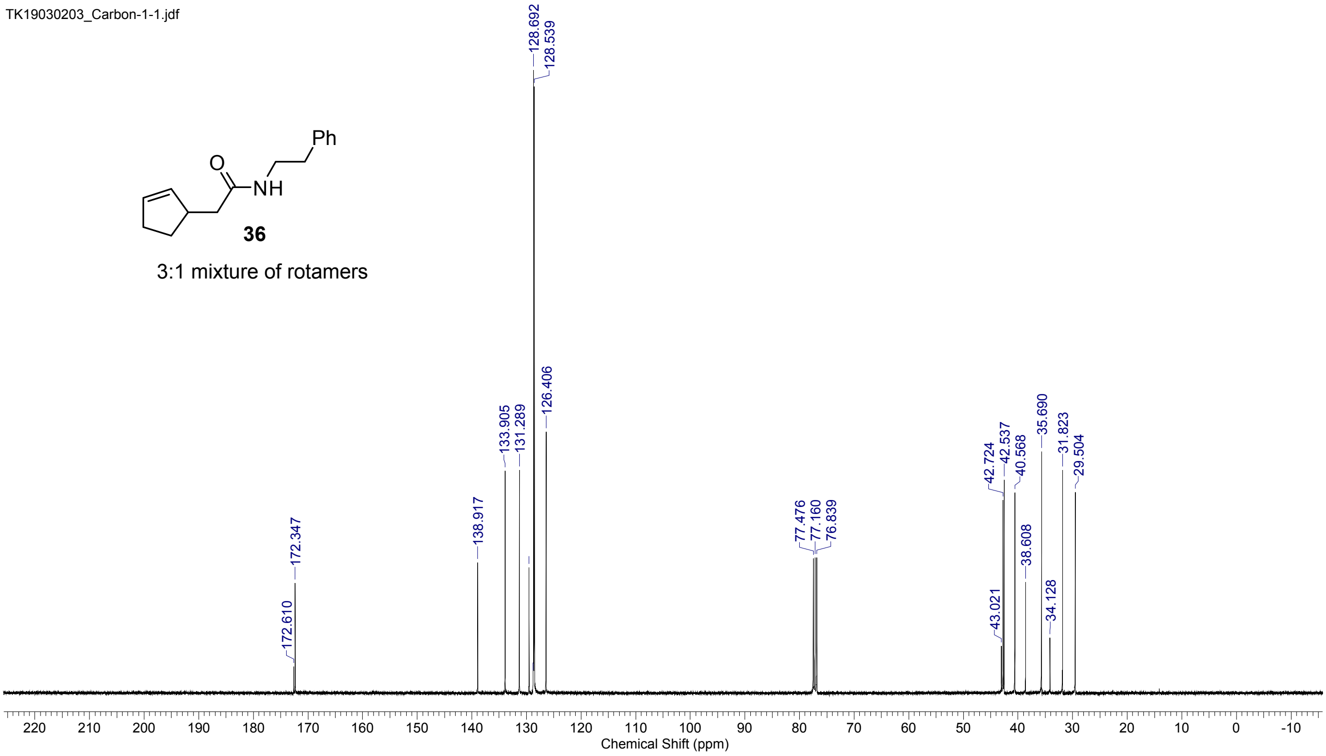

2019/03/07 18:47:57

Acquisition Time (sec)	2.1863	*Comment*	single_pulse	*Date*	07 Nov 2017 09:45:29		
Date Stamp	07 Nov 2017 09:44:38						
File Name	¥¥Mac¥iCloud¥ [illegible]¥NMR [illegible]¥2017NMR K¥2017NMR(JEOL)¥ [illegible]NMR2017¥TT17110702_Proton-1-1.jdf						
Frequency (MHz)	399.78	*Nucleus*	1H	*Number of Transients*	8	*Origin*	ECA
Original Points Count	16384	*Owner*	delta	*Points Count*	32768	*Pulse Sequence*	proton.jxp
Solvent	CHLOROFORM-d	*Spectrum Offset (Hz)*	2003.4905	*Sweep Width (Hz)*	7494.00	*Temperature (degree C)*	18.900

TT17110702_Proton-1-1.jdf

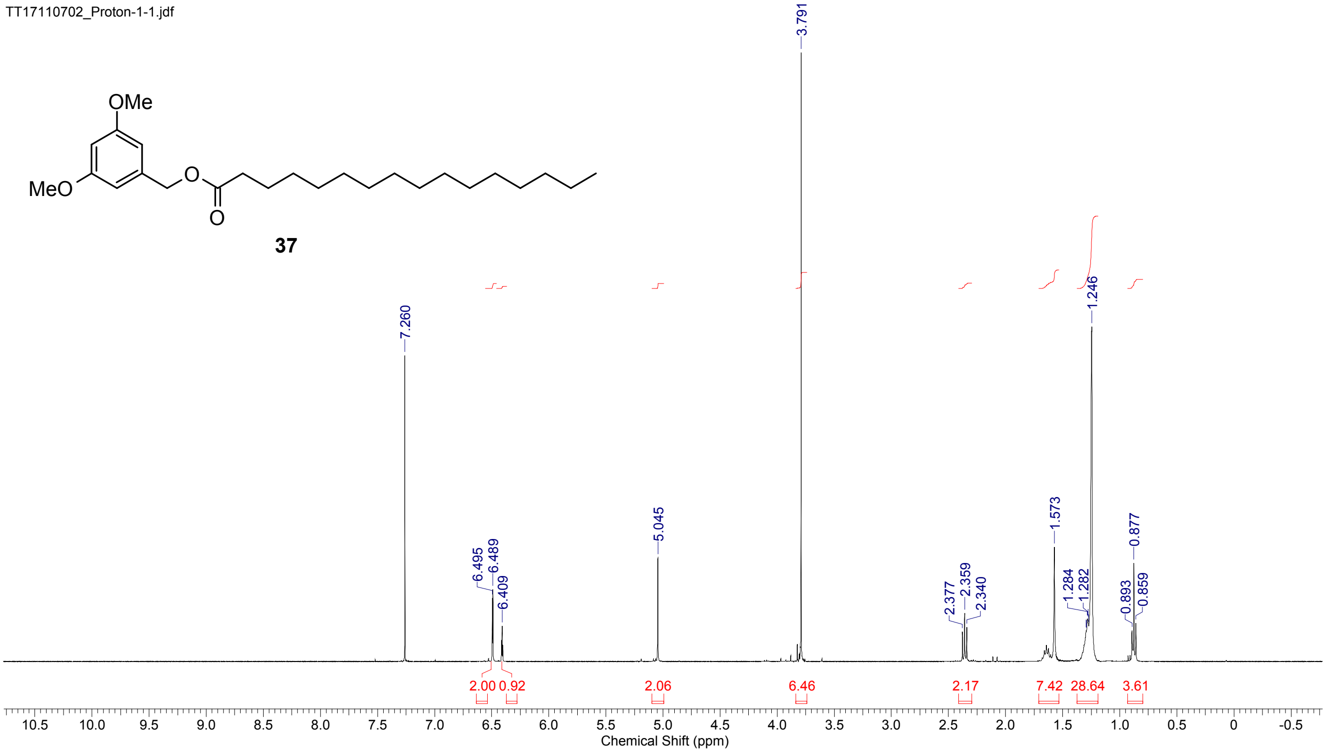

2019/03/07 18:53:59

Acquisition Time (sec)	0.8527	*Comment*	c13-NMR	*Date*	Aug 4 2014	*Date Stamp*	Aug 4 2014
File Name	¥¥Mac¥iCloud¥ [illegible]¥NMR [illegible]¥2014NMR K¥yasumoto¥14080401_C.fid¥fid					*Frequency (MHz)*	75.46
Nucleus	13C	*Number of Transients*	1024	*Original Points Count*	19335	*Points Count*	65536
Pulse Sequence	s2pul	*Receiver Gain*	30.00	*Solvent*	CHLOROFORM-d		
Spectrum Offset (Hz)	8955.6230	*Spectrum Type*	STANDARD	*Sweep Width (Hz)*	22675.74	*Temperature (degree C)*	AMBIENT TEMPERATURE

14080401_C

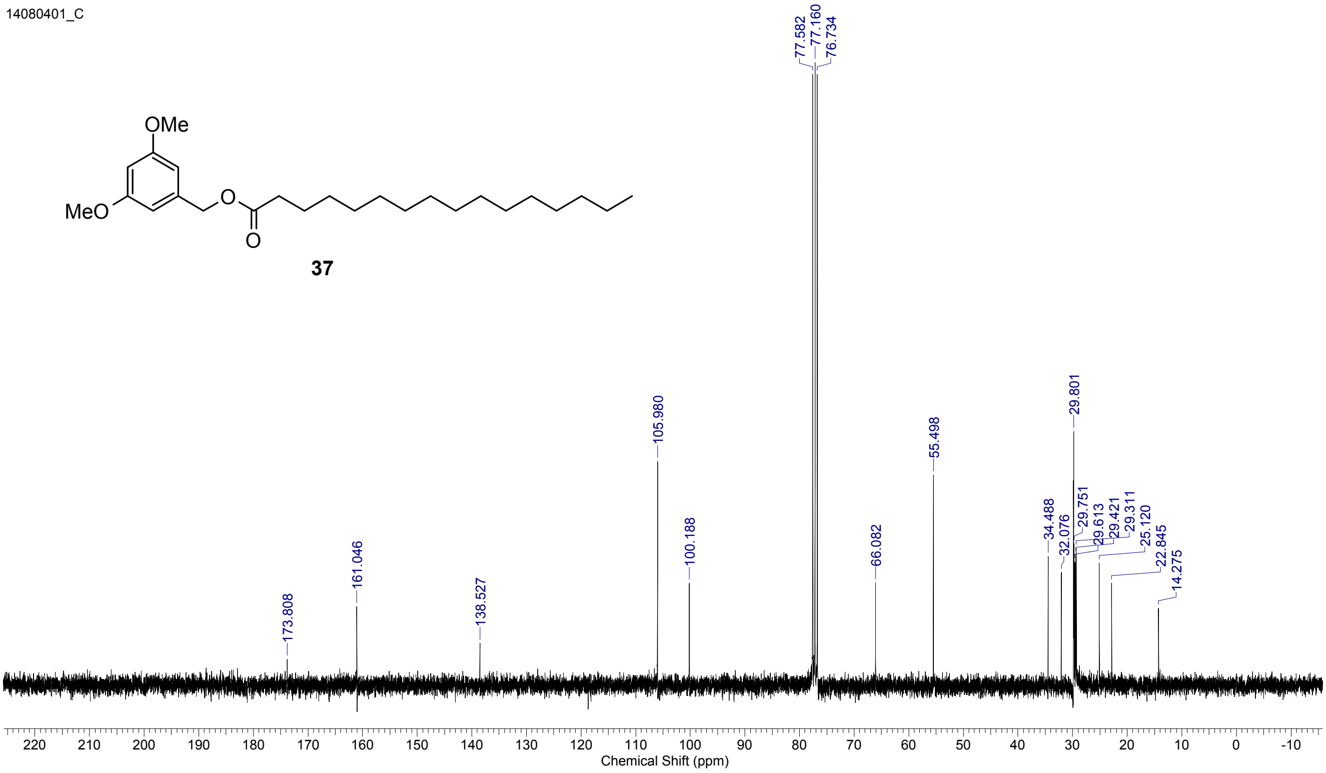

2019/03/07 19:01:23

Acquisition Time (sec)	2.1863	*Comment*	single_pulse	*Date*	21 Jan 2019 11:16:36		
Date Stamp	21 Jan 2019 11:15:45						
File Name	¥¥Mac¥iCloud¥[illegible]¥NMR[illegible]¥2018NMR_K¥2018NMR(JEOL)¥[illegible]NMR2018¥TK19012101_Proton-1-1.jdf						
Frequency (MHz)	399.78	*Nucleus*	1H	*Number of Transients*	8	*Origin*	ECA
Original Points Count	16384	*Owner*	delta	*Points Count*	32768	*Pulse Sequence*	proton.jxp
Solvent	CHLOROFORM-d	*Spectrum Offset (Hz)*	2003.4905	*Sweep Width (Hz)*	7494.00	*Temperature (degree C)*	19.300

TK19012101_Proton-1-1.jdf

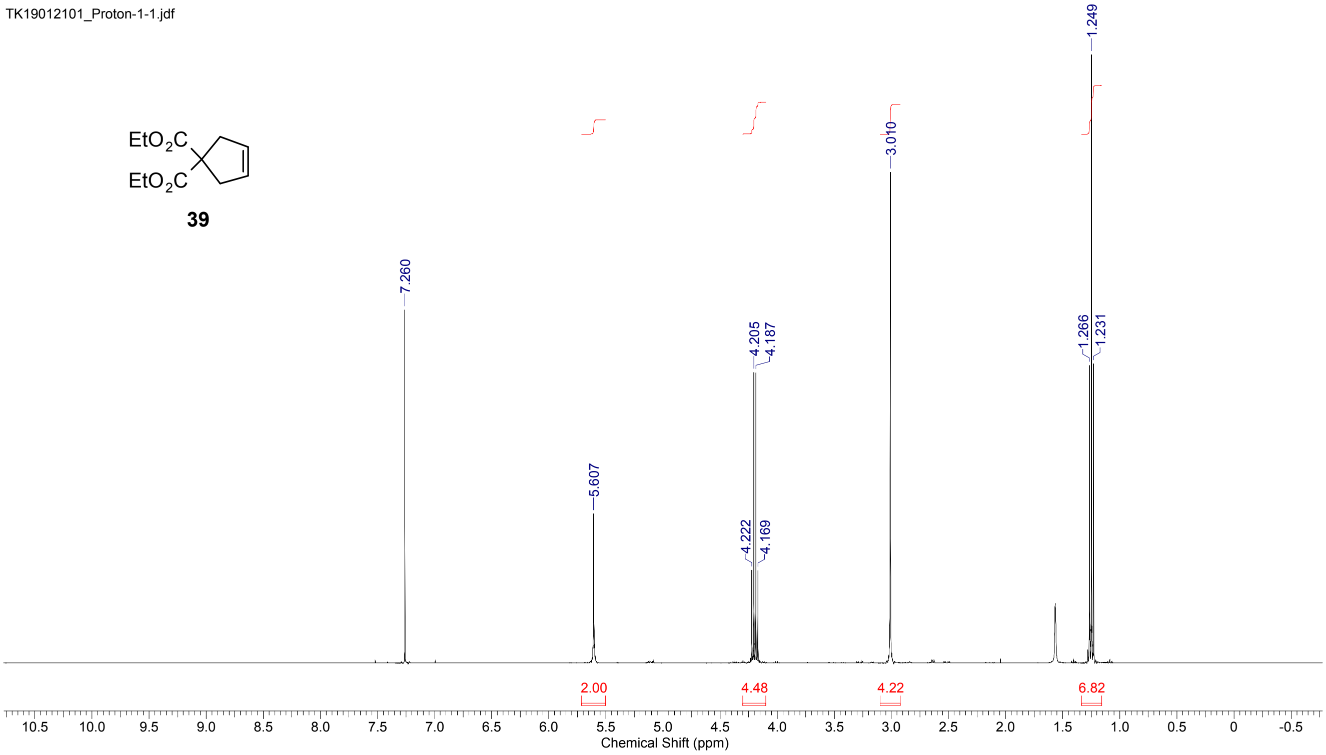

2019/05/27 10:57:10

Acquisition Time (sec)	0.8527	*Comment*	c13-NMR	*Date*	Feb 25 2019	*Date Stamp*	Feb 25 2019
File Name	¥¥Mac¥iCloud¥[illegible]¥NMR[illegible]¥2018NMR_K¥2018NMR(agilent)¥kawakami¥TK19022502_C.fid¥fid					*Frequency (MHz)*	75.46
Nucleus	13C	*Number of Transients*	64	*Original Points Count*	19335	*Points Count*	65536
Pulse Sequence	s2pul	*Receiver Gain*	30.00	*Solvent*	CHLOROFORM-d	*Spectrum Offset (Hz)*	8952.4512
Spectrum Type	STANDARD	*Sweep Width (Hz)*	22675.74	*Temperature (degree C)*	AMBIENT TEMPERATURE		

44-C(TK19022502_C).esp

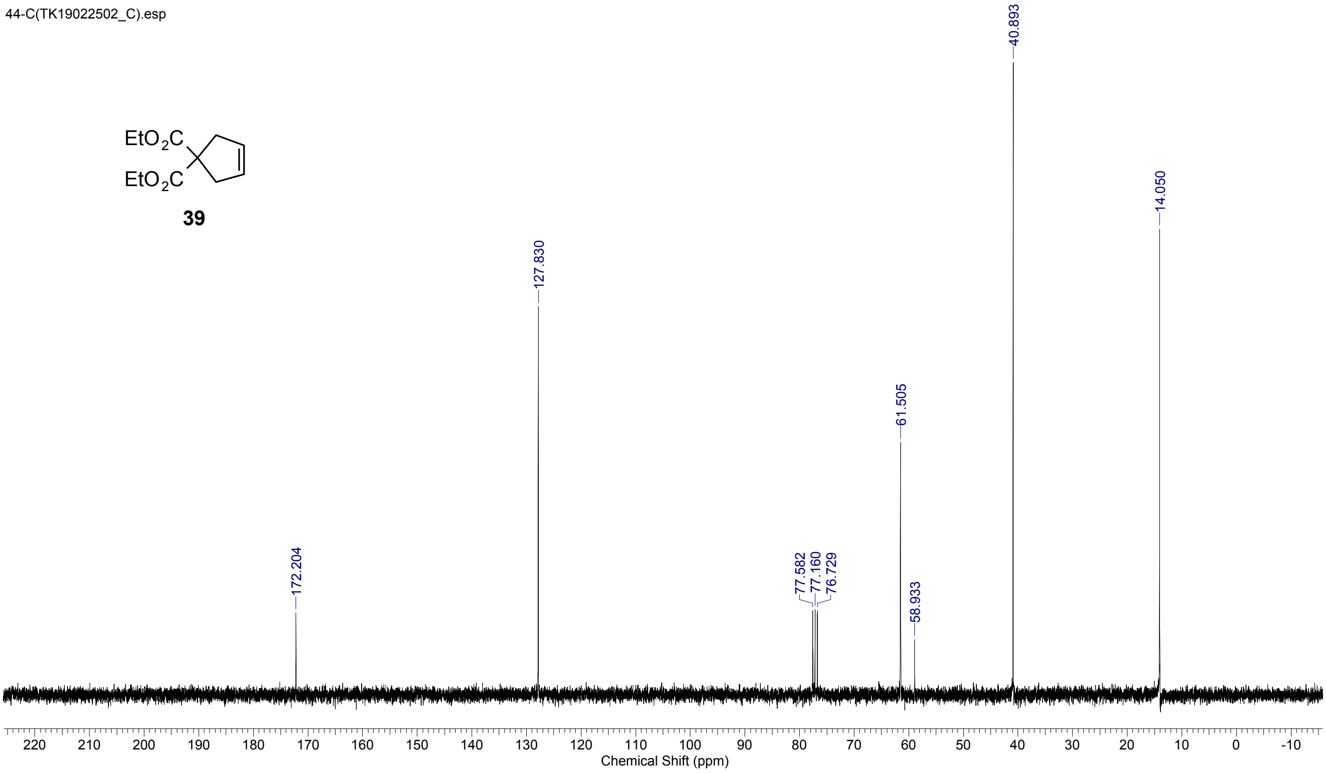

Supplement: Supplementary file 1 — Supplementary [file ASIA-14-3921-s001.pdf]
